# Concordant Metabolite Differences Across Cecal Content, Liver, and Plasma in Mice Fed an Obesogenic Diet Containing Common Bean (*Phaseolus vulgaris* L.): A Multi-Compartment Metabolomics Study

**DOI:** 10.3390/nu18213669

**Published:** 2026-11-05

**Authors:** Henry J. Thompson, Vanessa K. Fitzgerald, Tymofiy Lutsiv, John N. McGinley, Elizabeth S. Neil, Hisham Hussan

**Affiliations:** 1 Cancer Prevention Laboratory, Colorado State University, Fort Collins, CO 80523, USA; vanessa.fitzgerald@colostate.edu (V.K.F.); john.mcginley@colostate.edu (J.N.M.); elizabeth.neil@colostate.edu (E.S.N.); 2 Department of Internal Medicine, University of California, Davis, CA 95817, USA; hhussan@health.ucdavis.edu

**Keywords:** common bean, *Phaseolus vulgaris*, metabolomics, cecal metabolome, gut–liver axis, ceramides, branched-chain amino acids, polyunsaturated fatty acids, hepatic transcripts, adiposity

## Abstract

**Background/Objectives:** How metabolic changes accompanying common bean (*Phaseolus vulgaris* L.) consumption are organized across gut, liver, and blood, and which are separable from lower adiposity, is unclear. **Methods:** Male C57BL/6J mice were fed an obesogenic diet with or without cooked bean powder supplying 70% (nominal) of total dietary protein for 14 weeks; diets were assigned to whole cages (4 per diet). Diets were formulated to nominally match macronutrient proportions (*w*/*w*) from ingredient and supplier values; sources, and hence estimated protein, amino acid, α-linolenate, and fiber supply, differed; intake was not measured. Cecal content, liver, and plasma were profiled by untargeted metabolomics and liver by RNA-seq for selected gene sets. Metabolite classes selected by three-compartment concordance were examined against prior predictions. **Results:** Diet groups separated in every compartment, most strongly in cecal content. In all three, 36 host metabolites differed concordantly and 22 KEGG pathways were significant. Five classes met the selection criteria: branched-chain amino acids and their keto acids accumulated in cecal content despite lower estimated supply; long-chain *n*-3 and *n*-6 fatty acids shifted reciprocally while hepatic *Fads2*, *Elovl5*, and *Fads1* transcripts were 45–61% of control; and glutathione-related metabolites, ceramides, and bile acids differed as predicted. Total depot mass was significantly lower only in the full cohort (−12%). After MANCOVA adjustment for depot redistribution and hepatic lipid, the branched-chain class retained its diet association (q-value = 0.012), the glutathione-related (q-value = 0.047) and *n*-3 (q-value = 0.049) classes did so provisionally, the former and the hepatic component of the latter depending on one cage in cage-aware models, and ceramides (q-value = 0.20) and bile acids (q-value = 0.15) did not. **Conclusions:** Whether these differences drive or follow the leaner phenotype requires time-course, single-constituent designs in both sexes.

## 1. Introduction

Diets rich in pulses, including common bean (*Phaseolus vulgaris* L.), are associated with a lower risk of obesity, type 2 diabetes, and cardiovascular disease [1,2,3,4], and dietary substitution with cooked bean powder (beans cooked, freeze-dried, and milled) attenuates diet-induced obesity and hepatic steatosis in preclinical models [5,6,7,8,9,10,11]. These benefits are widely attributed to the bean’s integrated food matrix, i.e., protein, minerals, dietary fiber (including resistant starch and other fermentable fiber), and phytochemicals acting in concert, rather than to any single isolated constituent [12,13]. However, the metabolic consequences of cooked bean intake at the level of the tissue metabolome and how those effects are organized across the gut–liver–plasma axis remain incompletely defined.

In a series of studies in C57BL/6J mice of both sexes fed an obesogenic diet in which cooked bean replaces part of the protein source, we have previously reported reduced body weight and adiposity [5], attenuated hepatic steatosis with remodeling of the hepatic transcriptome [6], lower hepatic ceramides [14], altered bile-acid metabolism [15], and dose-dependent restructuring of the cecal microbiome [16]. Two of those reports [14,15] drew in part on the same untargeted metabolomics run of liver and plasma that is analyzed again here, and the hepatic RNA-sequencing (RNA-seq) data reported in [6], which showed lower transcripts of lipogenic enzymes, desaturases, and elongases with bean feeding, are re-examined here for selected gene sets in relation to the fatty-acid data. We have not reported the cecal luminal metabolome, the compartment in which diet and microbiome interact, or an integrated analysis of the three compartments. Those two elements, and the relation between the published transcripts and the fatty-acid indices in the same biospecimens, are the contributions of this report. We included sphingolipid and bile-acid results to complete the cross-compartment picture because we had not previously attempted to dissociate diet effects from adiposity effects on these variables. However, the ceramide and bile acid data included here do not constitute an independent replication of the previously reported work.

A recurring problem in dietary metabolomics is that a metabolite that changes with diet may contribute to a protective phenotype or may simply track a change in that phenotype (e.g., fat mass) [17,18,19,20,21,22,23]. Given that common bean feeding lowers adiposity, any metabolite that also covaries with adiposity could be a consequence of lower body fat or a contributor to it. Cross-sectional profiling at a single terminal time point cannot answer that question, and we do not attempt to do so here. Such a design can describe which metabolites differ by diet in each compartment, whether those differences are concordant across compartments, and whether the diet effect on a metabolite class persists after statistical adjustment for adiposity, within the limits of the sample. We consider the last of these exploratory; statistical adjustment can identify metabolite classes whose diet association is not explained by a single adiposity measure, but it cannot establish independence from adiposity or a causal role.

The data-driven investigation reported herein focused on untargeted metabolomic profiling of cecal content, liver, and plasma from the same animals, integrated by cross-validated multivariate modeling, cross-compartment concordance, Kyoto Encyclopedia of Genes and Genomes (KEGG) pathway topology, and exploratory multivariate analysis of covariance of metabolite-class composites on diet and adiposity. The research objectives were: (1) to characterize the metabolomic response to common bean in each compartment, with the cecal metabolome as the new element; (2) to quantify concordance of the response among compartments; and (3) to determine, as an exploratory, hypothesis-generating question, whether the diet association of selected metabolite classes persists after adjustment for adiposity. A fourth objective followed from the fatty-acid result: (4) to determine, from RNA-seq of the same liver pools, whether transcripts of the desaturase–elongase enzymes shared by the *n*-3 and *n*-6 pathways, and the transcripts of the hepatic branched-chain amino acid (BCAA) and glutathione pathways, differed with diet.

## 2. Materials and Methods

Procedural detail is provided in the Supplementary Methods (Sections SM1–SM9). That document begins with a table that locates each reporting item (Table SM1).

### 2.1. Animals and Diets

The animals were the male cohort of a dose-response study of white kidney bean, for which the design, diets, and adiposity, hepatic steatosis, cecal microbiome, ceramide, and bile-acid outcomes have been previously reported [5,6,14,15,16]. We consider only the male control and high bean intake diet groups here. Male C57BL/6J mice (stock 000664; The Jackson Laboratory, Bar Harbor, ME, USA) were received at 20 days of age. On arrival mice were unpacked at random, housed 5 per cage, and adapted to a purified diet until 8 weeks of age. Because group housed male mice can be aggressive if rehoused, diets were assigned to whole cages (4 cages of 5 mice per diet), with individual animals exchanged between cages so that mean body weight and pre-allocation weight gain were balanced across groups (SM1); the cage was therefore the unit of diet assignment and of diet delivery, and the treatment-level replication is 4 cages per diet (Section 2.7). Food intake was not measured (SM1). Necropsy took place in the fed state at 22–23 weeks of age. Plasma was separated from blood collected into ethylenediaminetetraacetic acid (EDTA) tubes, and liver and cecal content were snap-frozen in liquid nitrogen and stored at −70 °C. Tibia length and the wet weights of the inguinal subcutaneous, mesenteric, retroperitoneal, and epididymal fat depots were recorded for every animal; total visceral fat is the sum of the last three. Husbandry and necropsy procedures are given in SM1. The study was conducted under Colorado State University Institutional Animal Care and Use Committee (IACUC) protocol 18-7746A, later renumbered KP1431 and KP5432 (Institutional Review Board Statement).

Diets were prepared in our laboratory and formulated, as reported [5,6,14,15,16], to nominally equal macronutrient proportions by weight from ingredient and supplier values (58.6% total carbohydrate, 20% total protein, 15.5% total fat, and 5.9% micronutrients, on the AIN-93G basis [24]), providing 32.5% of energy from fat. The bean ingredient was white kidney bean, cooked as a whole food with its leachate, then freeze-dried and milled. It was included at 40.8 g/100 g in the bean diet group in place of the corresponding amounts of casein, corn starch, cellulose, and sucrose, and L-tryptophan (0.06 g/100 g) was added. Ingredient-based estimates of the amino acid and fatty-acid composition of each diet were derived from food-composition data [25,26,27,28] (Table S1; Data S5; SM2). The finished diets were not assayed, so the macronutrient match is nominal and the amino acid and fatty-acid values are estimates.

### 2.2. Samples for Analysis

Seven male mouse samples per diet group were evaluated, a number recommended by Metabolon, Inc. (Morrisville, NC, USA), based on their experience in preclinical models and reached in discussions that balanced the number of samples evaluated per group against the cost of analysis. Liver and cecal content samples were pools of approximately equal amounts of tissue from two mice (43–60 mg per donor for cecal content and 50–62 mg for liver), paired by adjacent adiposity rank within diet group without regard to cage; 5 of the 14 profiled pools hold two animals from one cage and 9 span two cages (SM3; Data S3). For plasma, 4 of the 14 samples were two-animal pools, and 10 were from a single mouse because plasma volume was insufficient to pool (SM3).

### 2.3. Metabolomic Profiling

Samples were profiled on the Metabolon Global Discovery (HD4) platform (Metabolon, Inc., Morrisville, NC, USA) [29], which uses four chromatographic methods on an ultra-performance liquid chromatography (UPLC) system (ACQUITY; 范, Milford, MA, USA) coupled to an Orbitrap mass spectrometer (Q Exactive; Thermo Fisher Scientific, Waltham, MA, USA) (SM4). Quality control followed the platform’s standard design: a technical-replicate pool made from every client sample (CMTRX), process blanks, and internal standards, with experimental samples randomized within the run. Median relative standard deviations in the CMTRX replicates were 5–6% for internal standards and 8–10% for endogenous biochemicals across the three matrices.

Compounds were identified by retention index, accurate mass, and tandem mass spectrometry (MS/MS) match to an authenticated standard in the Metabolon library; names with an asterisk indicate identification without a purified standard. Peak areas were batch-normalized by setting each compound’s run-day median to 1.0, and missing values were imputed with the per-compound minimum observed value. Log_2_-transformed, Pareto-scaled matrices were derived for parametric and multivariate analyses (Data S4). Unnamed features were retained in the differential-abundance tables but excluded from host-focused analyses. Features missing in more than half of the samples are flagged in Data S1, and the number of non-detects is reported in the text when a discussed metabolite is affected. The raw peak-area, batch-normalized, and imputed tables for all three matrices are provided in Data S6.

### 2.4. Differential Abundance and Multivariate Analysis

Per-metabolite fold changes (FC; bean/control) were computed as the ratio of group means of the batch-normalized, imputed data. Group differences were tested with Welch’s unequal-variance *t*-test on log_2_-transformed, Pareto-scaled values, with Benjamini–Hochberg false-discovery-rate (FDR) control within each matrix [30]. As a check on the influence of individual pools, every comparison was repeated with the two-sided Mann–Whitney U test (exact in the absence of ties; otherwise normal approximation with tie and continuity correction).

We defined diet-exposure markers as compounds that, in this design, enter the host predominantly from the bean ingredient or its cooking rather than from endogenous mammalian synthesis. The rule was applied by chemical identifier and synonym, and the complete list of excluded compounds, with the rule applied to each, is provided in Data S1 (SM5).

Group separation was assessed by two-component partial least-squares discriminant analysis (PLS-DA) on the host feature set (named, non-exposure, and not constant across the 14 samples: 768, 948, and 895 features in cecal content, liver, and plasma, respectively). Performance was evaluated by leave-one-out cross-validation with Pareto scaling refit inside every fold, and the significance of Q^2^ was assessed against 1000 random permutations of the group labels. Features were ranked by variable importance in projection (VIP). Cross-validation and permutation testing are safeguards against overfitting in a design with 14 observations and several hundred features; they do not exclude it [31,32]. Therefore, we interpret the PLS-DA results only as evidence of group separation, not as a predictive model.

### 2.5. Fatty Acid Ratios

Because the platform scales each metabolite to its own median, summing scaled abundances across different fatty acids does not represent a molar pool, so we did not use a summed *n*-3:*n*-6 ratio. Instead, we report ratios of individual fatty acids. For any two metabolites, the ratio of median-scaled values differs from the molar ratio only by a constant, so the between-group FC in a pairwise ratio is the FC in the molar ratio. Per-sample log_2_ ratios were compared between groups by Welch’s *t*-test with a 95% confidence interval (CI) on the *FC* and checked by Mann–Whitney U. The eight ratios examined were FDR-corrected within matrix (SM6). The platform does not resolve the isomers of 18:3 and 20:3. We refer to them as α-linolenate and dihomo-γ-linolenate for the reasons given in SM6, and the ratios that use them carry this uncertainty.

### 2.6. Enrichment and Pathway Analysis

Pathway analysis followed the quantitative procedure in MetaboAnalyst 6.0 [33] and was implemented in R using the MetaboAnalyst *Mus musculus* KEGG pathway library and compound database, mapping compounds to KEGG via their Human Metabolome Database (HMDB) identifiers [34]. The data were generalized-log_10_-transformed and Pareto-scaled [35] without further sample normalization. Each pathway with at least one matched compound was tested against its KEGG metabolite set [36] by the global test, with FDR across tested pathways within each matrix, and pathway impact was the summed relative betweenness centrality of the matched compounds [37]. The global test was computed with the R package globaltest [38], and the pathway library and compound database were those distributed with MetaboAnalystR [39]. Identical settings were applied to all three matrices (Table S5; SM6). The global test is non-directional; the direction of change was taken from the differential abundance results.

### 2.7. Cross-Compartment Concordance and Multivariate Adjustment for Adiposity

Metabolites were matched across compartments by Metabolon compound identifier, and log_2_FC values of the matched host metabolites were compared by Spearman correlation. Host metabolites differing at *p* < 0.05 and in the same direction in all three compartments were tabulated, with those also meeting FDR < 0.05 in all three identified separately.

For each of six metabolite classes (ceramides, bile acids, BCAA parents or catabolites, *n*-3 polyunsaturated fatty acids, glutathione-related metabolites, and ketone bodies with acylcarnitines), a composite score was computed per sample as the mean Pareto-scaled abundance of directionally coherent member metabolites (membership in Data S3). The adiposity covariates were reduced before modeling. The four fat depot masses linked to each pool (subcutaneous, mesenteric, retroperitoneal, and epididymal fat) were compared between diets as a profile by two-sample Hotelling’s T^2^ and summarized by principal component analysis of their z-scores; hepatic lipid (mg/g dry weight), from the previously reported assay [6], was carried as a separate covariate. For the class-level models, which require one covariate vector per pool across the three compartments, the phenotype is the mean of the two donors of each pool; the donor-correct phenotypes, in which 10 of the 14 plasma composites are linked to their single donor (Section 2.2), are used in the compartment-level and univariate analyses (Table S4C,E,F). Body weight was not used as a covariate in the multivariate models because it combines fat and lean mass and was collinear with the first depot component (r = 0.83).

Adjustment was then tested by multivariate analysis of covariance (MANCOVA) in two orientations. In the primary, class-level analysis, the response for each class was the vector of its three compartment composites (cecal content, liver, plasma; three responses; n = 14 pools) and the predictors were diet group and, in successive models, no covariate; total depot mass; subcutaneous fat; hepatic lipid; the first depot component; the second depot component; the first two components; the first component with hepatic lipid; and total depot mass with hepatic lipid (nine models, M0–M8, all reported in Table S4B; Table 1 reports five of them). In the secondary, compartment-level analysis, the response was the vector of the six composites measured in one compartment with the same predictor sets. The diet term has one degree of freedom, for which the Hotelling–Lawley, Pillai, Wilks, and Roy statistics coincide and yield an exact F; we report the Hotelling–Lawley trace and its F with (m, n − k − m + 1) degrees of freedom, where m is the number of responses and k the number of model parameters. Each model was refitted with each pool omitted in turn, and the largest resulting diet *p*-value is reported as the leave-one-pool-out sensitivity.

The six classes, their composite memberships, and the covariate sets were fixed, from the concordance screen and the prior predictions of Section 3.3, before any adjustment model was fitted. The depots were reduced to principal components, and at most two covariates entered any model, to hold the parameter count at 4 against 14 observations. Collinearity was quantified: in the conservative model the variance inflation factors were 4.8 for diet, 6.0 for the first depot component, and 11.6 for hepatic lipid, because the two covariates are correlated with each other (r = 0.91) and each with diet; collinearity inflates the standard error of the diet term and lowers the power of the adjusted test without changing its nominal error rate, and the single-covariate models (variance inflation factors ≤ 2.4 for the depot covariates and 4.6 for hepatic lipid) and the total-depot-mass model (1.7–6.1) are reported alongside for that reason. Because the exact F assumes multivariate normality of the residuals, which cannot be checked at this sample size, the significance of each class-level diet term was also assessed by permutation (Freedman–Lane residual permutation: the residuals of the covariate-only model were permuted 10,000 times and added to its fitted values, and the full model was refitted to each permuted response; SM7) and, as a one-response check on model stability, by analysis of covariance of the class-mean composite (the mean of the three compartment composites) with the same covariate sets; because the mean cancels where the compartments differ in direction, this check is interpreted together with the per-compartment univariate analyses of Table S4D.

Univariate ordinary least-squares regressions of each composite on diet and one adiposity covariate at a time (subcutaneous, mesenteric, total visceral, or epididymal fat, body weight, or hepatic lipid), with the commonality decomposition [40] of explained variance, are reported as descriptive follow-up (Table S4F; Data S3; SM7). For each composite, they report the fraction of explained variance shared by diet and a covariate, and the diet term under covariates not used in the multivariate models, including body weight. A nominal diet term for one composite in a single-covariate model is not interpreted as retention of its class; the class-level test, which examines the three compartments jointly, is the primary analysis.

We consider these analyses exploratory and associative. Adiposity is a post-allocation variable that may lie on the causal pathway from diet to metabolite, and a model containing diet and adiposity cannot separate diet-driven from adiposity-driven effects; it can show only whether the diet association of a class persists, is attenuated, or disappears when a given adiposity measure is included. The class-level diet tests have 8–10 denominator degrees of freedom, so a non-significant adjusted diet term is a failure to separate the two, not evidence of no effect; the compartment-level models (six responses on 14 observations) are near-saturated and are reported in Table S4C as descriptive only.

Because diet was delivered by cage and pool donors were paired without regard to cage, samples that share a cage are not independent [41,42,43,44], and neither the leave-one-pool-out procedure nor the residual permutation addresses that dependence. Diets were assigned to whole cages, but the allocation was balanced on body weight by exchanging individual animals between cages, so it was not an unrestricted randomization of cages, and a design-based (randomization) test at the cage level is not available. As a model-based sensitivity analysis that accommodates pools containing animals from two cages (SM9; Table S8), each composite and each host metabolite was analyzed by a multiple-membership linear mixed model with a cage random effect [45,46,47,48], in which a pool carries weight one-half on each donor cage (weight one on a single cage for a same-cage pool, and for plasma the cage of the contributing donor); the ratio of cage to residual variance was estimated by restricted maximum likelihood [49], the diet coefficient by generalized least squares at that estimate, and the diet term was tested by an approximate Wald *t* statistic, with a 95% CI, on the 6 between-cage degrees of freedom (8 cages − 2 diets) [50]. With eight cages the variance components are imprecise, so the Wald *p*-values are approximate [51,52], and a cage variance estimated at zero means that the data do not separate cage from residual variance, not that cage effects are absent. Each mixed model, and the class-level MANCOVA of Table 1, was also refitted with every pool holding a donor from one cage omitted in turn (leave-one-cage-out [53,54,55]; 10–12 pools remain, and omitting either of the two cages that contribute to 4 pools leaves 4 denominator degrees of freedom for the diet test under the conservative model), and the largest resulting diet *p*-value, the range of the diet coefficient, and whether its sign was the same in all eight omissions are reported. The mixed models are fitted to one composite at a time; the joint, three-compartment test of a class remains the pool-level MANCOVA of Table 1. Neither analysis adds replication: the design has 4 cages per diet, and the *p*-values reported elsewhere in this paper are conditional on the pool being the unit of analysis.

### 2.8. Liver RNA Sequencing and Transcript Analysis

The liver RNA-seq data were generated for our study of hepatic steatosis in this cohort [6] and are reused in [14]. The gene-set analysis reported here is new, and we do not present the data as an independent replication. RNA was sequenced from the 20 male liver pools (10 per diet), which include the 14 pools profiled by metabolomics with the same two donor animals per pool. Library preparation, sequencing, and mapping were as described [6,14] (SM8). Gene-level counts were normalized by the trimmed mean of M-values [56] and exported as counts per million (CPM); genes with CPM ≥ 1 in at least 5 of the 20 pools were retained (12,116 genes).

The analysis was restricted to five gene sets specified from the metabolite results before the per-gene values were extracted: the polyunsaturated fatty-acid desaturase–elongase module, lipogenic targets of sterol regulatory element-binding protein-1c (SREBP-1c), the peroxisome proliferator-activated receptor-α (PPARα) axis, BCAA catabolism, and glutathione synthesis and turnover (member genes in Table S7 and SM8). Because genome-wide results from these data were reported previously [6], the selection was not blind to that report.

Differential expression was assessed on log_2_(CPM + 1) with an empirical-Bayes moderated t statistic incorporating a mean–variance trend, the limma-trend estimator [57] implemented in the R package limma (Supplementary Code), with Benjamini–Hochberg control of the FDR across all retained genes. This differs from the count-based model applied in [6]. For the 14 pools shared with the metabolomics, log_2_(CPM + 1) was also compared by Welch’s *t*-test and the Mann–Whitney U test. Product-to-precursor fatty-acid indices were formed per pool as in Section 2.5 and related to transcript abundance by Pearson and Spearman correlation. Because the two diets form separate clusters on both axes, the across-pool coefficients restate the group difference and are not interpreted as within-group relations. Transcript abundance was not confirmed by quantitative PCR, and protein abundance and enzyme activity were not measured. The sequencing data are deposited in the Gene Expression Omnibus under accession GSE227120; the gene-set tables, per-pool CPM, fatty-acid indices, and correlations are provided as Data S7.

### 2.9. Statistical Power, Multiplicity, and Software

The design provides approximately 80% power to detect a standardized between-group difference of Cohen’s *d* ≈ 1.6 by two-sample *t*-test at *p* < 0.05, rising to d ≈ 1.9 at the nominal threshold corresponding to FDR < 0.05 (*p* ≈ 0.02). Non-significance for a given metabolite is not interpreted as evidence of no effect. FDR was controlled within each matrix for per-metabolite tests and for the pairwise fatty acid ratios. The *six* class-level MANCOVA diet terms under a given covariate set form one family, and the Benjamini–Hochberg q-value across the *six* is reported with each *p*-value for the conservative model (Table 1); the compartment-level MANCOVA (Table S4C) and the univariate composite regressions of Table S4F are descriptive, the latter with FDR across the 18 composites. Phenotype comparisons between groups were made by Welch’s *t*-test on the 14 pools and confirmed on all 40 animals.

Analyses were performed in R 4.6.1 [58] (limma 3.68.5 [57], globaltest 5.66.0 [38], lmtest 0.9-40, ggplot2 4.0.3), with a fixed random seed (20260924) for permutation tests; the pathway analysis used the MetaboAnalyst 6.0 *Mus musculus* KEGG library and compound database [39]. The analysis code is provided as Supplementary Code (the R Markdown document and its rendered report, whose session information lists exact package versions). Run on the Metabolon data tables (Data S6), the annotation, crosswalk, diet, and transcript files (Data S1, S3, S5, and S7), and the per-animal necropsy and hepatic-lipid files and genome-wide liver CPM export (Data S8), it reproduces the differential-abundance, multivariate, fatty acid-ratio, cross-compartment correlation, composite-regression, MANCOVA, cage-aware sensitivity, and transcript analyses reported here. The multiple-membership mixed model and the leave-one-cage-out refits were implemented in base R (SM9). The depot principal components, Hotelling’s T^2^, and MANCOVA were computed in base R (stats 4.6.1) as general linear hypothesis tests on the multivariate linear model, with the diet-term tests checked against stats::anova.mlm. The per-animal necropsy and hepatic-lipid files and the genome-wide CPM export are provided as Data S8, so that the code can be run from the supplementary archive alone (Data Availability Statement).

## 3. Results

### 3.1. The Diet Groups Separate in Every Compartment, and the Differences Are Largest in Cecal Content

**Figure 1:**
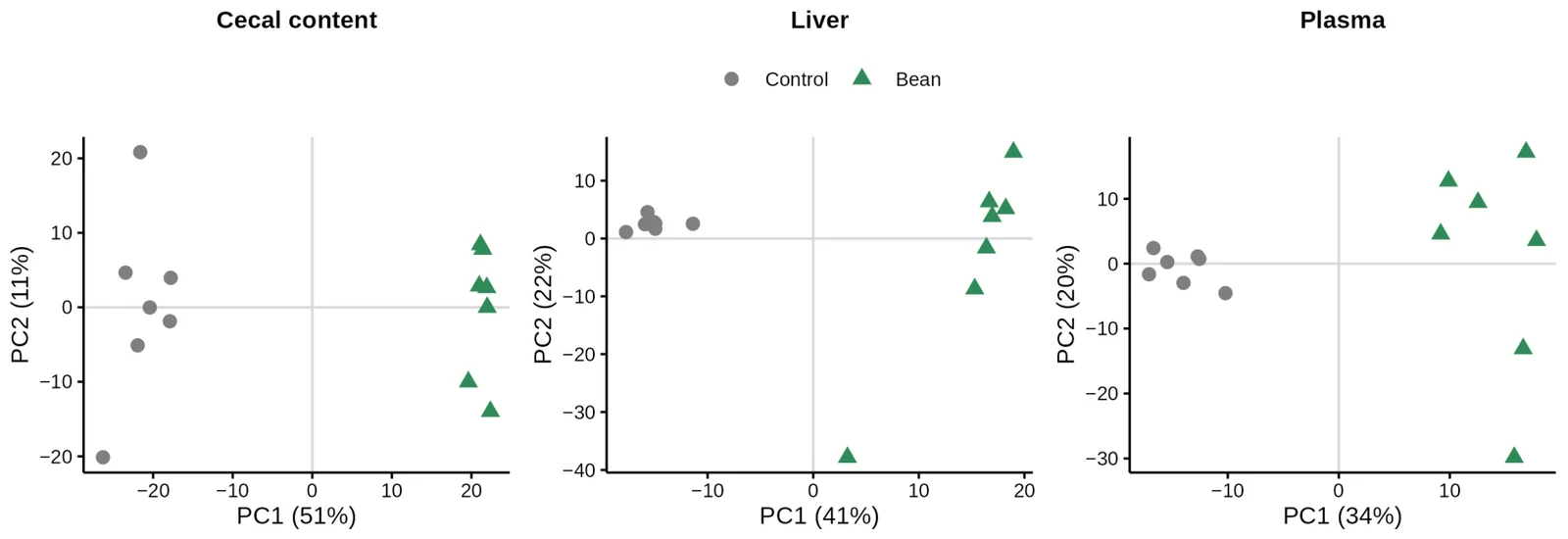
Principal component analysis of log_2_-transformed, Pareto-scaled abundances of all detected metabolites for cecal content, liver, and plasma. Each point is one sample (n = 7 per group; two-animal pools, except that 10 of the 14 plasma samples came from one donor; grey dots, control; green triangles, bean); axes show the first two principal components with percentage of variance explained.

During the study, there were no observable adverse effects of either diet. Of the 40 male mice assigned to the two diet groups (20 per group), none was excluded from pooling or removed from the study, and necropsy phenotypes were obtained from all 40 (100%). Of the 10 two-animal pools per group, 7 were submitted for metabolomic profiling; the 3 pools per group not submitted were those at the extremes of the adiposity distribution or, for 2 bean pools, those with insufficient sample for all three matrices (SM3; Data S3). Untargeted profiling detected 962, 1065, and 1024 metabolites (788, 958, and 919 named) in cecal content, liver, and plasma, respectively; of these, 959, 1065, and 1023 were detected in at least one of the 14 samples, and 959, 1065, and 1019 varied across them and were tested (Table S2). In every compartment, the diet groups separated along the first principal component (51%, 41%, and 34% of variance, respectively; Figure 1; Data S4). A two-component PLS-DA restricted to host metabolites gave leave-one-out Q^2^ of 0.99 (cecal content), 0.94 (liver), and 0.91 (plasma), with 100% leave-one-out classification accuracy, and the observed Q^2^ exceeded all 1000 label-permuted values in liver and plasma and all but one in cecal content (permutation *p*-value ≤ 0.002; mean permuted Q^2^ −0.57 to −0.64). Between 38% and 60% of metabolites differed at *p* < 0.05 in each matrix, the majority surviving FDR correction (Table S2), and Mann–Whitney testing classified 95–96% of all features as Welch’s test did at FDR < 0.05, including 91–95% of the Welch-significant features, with only one metabolite discussed below, hepatic α-linolenate (Welch q-value = 0.047; Mann–Whitney q-value = 0.060), changing status (Data S1). Two points follow from these data: (1) the diet groups separated in every compartment, and the magnitude of the differences, measured by the proportion of metabolites changed and the size of the FC values, was greatest in cecal content and smaller in liver and plasma; and (2) these observations describe where the largest differences were found but do not indicate where the response originated.

The number of metabolites that differed in relative abundance between the bean and control dietary groups in each biospecimen compartment was large, and many of those differences may have compartment-specific relevance. However, we judge that metabolites, metabolite classes, and metabolic pathways that differed significantly in all three compartments are more likely to be relevant to the host, and this judgment is the rationale for the analyses that follow. The results reported below are limited to those metabolites, classes, and pathways.

### 3.2. Thirty-Six Host Metabolites Differ in the Same Direction in All Three Compartments

**Figure 2:**
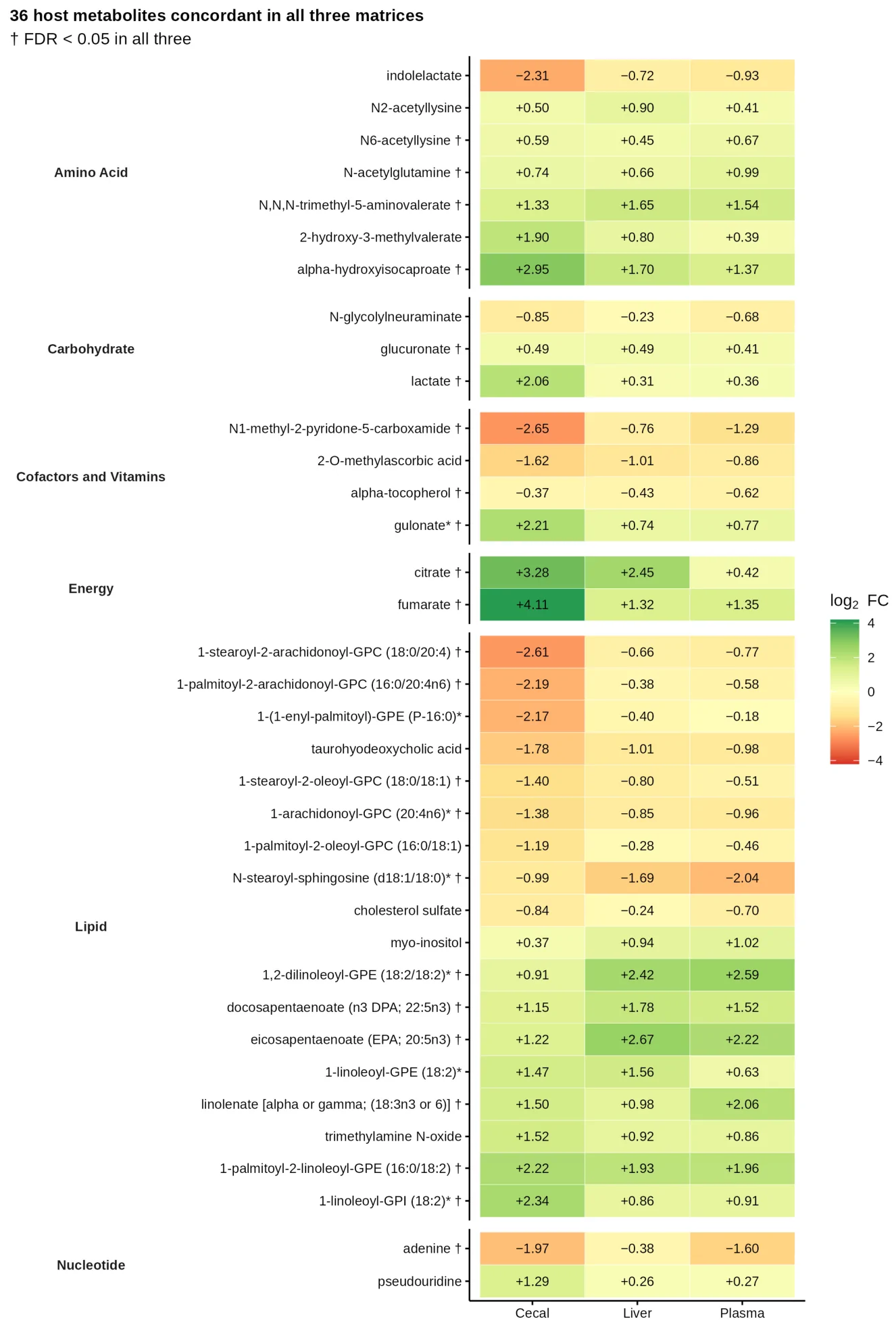
The 36 host metabolites that differed at *p* < 0.05 and in the same direction in cecal content, liver, and plasma (complete set; n = 7 samples per group per matrix). Cell values are log_2_FC (bean/control); green, higher with bean; red, lower. †, FDR < 0.05 in all three matrices; *, identified by retention index, accurate mass, and MS/MS match without a purified standard (Section 2.3). GPC, glycerophosphocholine; GPE, glycerophosphoethanolamine; GPI, glycerophosphoinositol; DPA, docosapentaenoate; EPA, eicosapentaenoate. Diet-exposure markers (Section 2.4; Data S1) are excluded.

Liver and plasma FC values were correlated across host metabolites (Spearman ρ = 0.48, n = 687 matched host metabolites, *p*-value ≈ 10^−40^), whereas the correlation of cecal content with either systemic compartment was weak (ρ ≈ 0.12). Thirty-six host metabolites differed at *p* < 0.05 and in the same direction in all three compartments, 23 of them at FDR < 0.05 in all three (Figure 2; Table S3). The set includes lower C18 ceramide, higher *n*-3 fatty acids, and higher tricarboxylic-acid-cycle intermediates (fumarate, citrate). Given that all three compartments were sampled at one terminal time point, concordance of direction and the larger cecal effect are consistent with a signal that arises in the gut and extends to liver and plasma, and equally consistent with the diet acting on each compartment in parallel; the weak cecal–systemic correlation argues against any simple transfer, but the data do not distinguish these possibilities.

### 3.3. Twenty-Two KEGG Pathways Are Significant in All Three Compartments, and Five Metabolite Classes Meet the Selection Criteria

**Figure 3:**
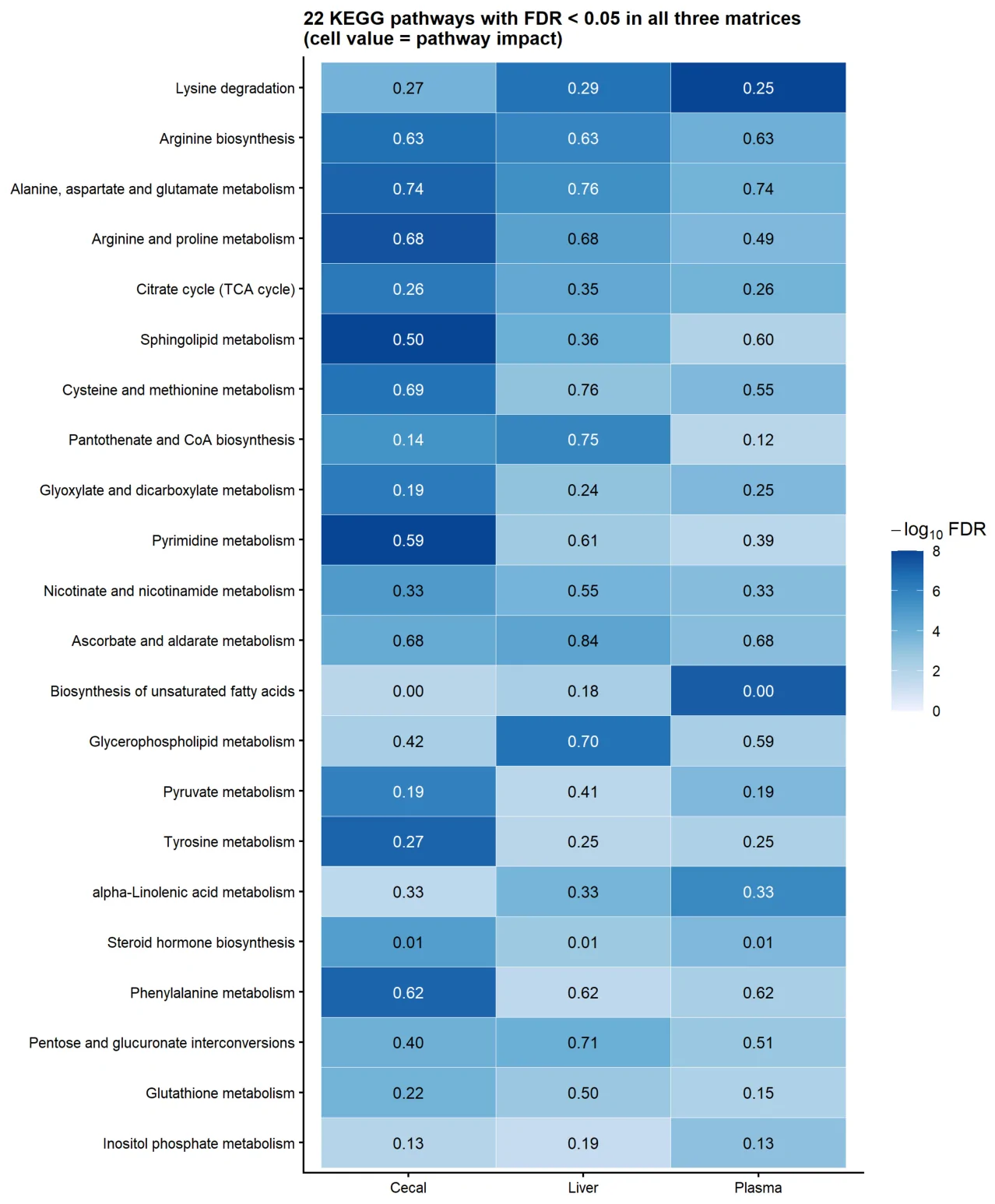
KEGG pathways with FDR < 0.05 in all three matrices (22 pathways; complete set). Cell shading, −log_10_ FDR from the global test (non-directional); cell value, pathway impact from relative betweenness centrality. n = 7 samples per group per matrix. Pathways are ordered by mean −log_10_ FDR across matrices; shading is capped at −log_10_ FDR = 8.

Quantitative enrichment and pathway-topology analysis (MetaboAnalyst 6.0; KEGG, *Mus musculus*) identified 22 pathways with FDR < 0.05 in all three matrices (Figure 3; complete results by matrix in Table S5 and Data S2). Significance and topological impact are distinct quantities and are reported separately: significance reflects the global test on the member metabolites, and impact reflects the relative betweenness centrality of the changed metabolites within the pathway map. From these data, four points can be made: (1) glycerophospholipid metabolism was among the three most significant pathways in liver (*q-value =* 2.7 × 10^−7^; impact 0.70); (2) sphingolipid metabolism was significant in all three matrices; (3) primary bile-acid biosynthesis was significant in cecal content and liver but not plasma, and valine, leucine, and isoleucine degradation in cecal content and plasma; and (4) α-linolenic-acid metabolism was significant in all *three* matrices, whereas linoleic-acid metabolism only in liver, in agreement with the reciprocal fatty-acid shift described in Section 3.5. Because roughly half of the measured metabolites changed in the cecum, near-universal pathway significance in that compartment reflects the global shift, and we consider the impact scores and the pattern across matrices more informative than significance alone.

We used the concordance screen to select the metabolite classes examined in Section 3.4, Section 3.5, Section 3.6, Section 3.7 and Section 3.8, applying two criteria. The entry criterion was representation in the three-compartment set at the metabolite level (Figure 2), the pathway level (Figure 3), or both. The second criterion was that a prediction for the class existed before the data were examined, from the compositional differences between the diets (Table S1) or from our previous reports on this cohort, so that each result could be judged against an expectation rather than described. Five classes met both:

(1) BCAA catabolism, represented by α-hydroxyisocaproate and 2-hydroxy-3-methylvalerate, higher in all three compartments against an estimated 27% lower dietary supply, although the valine, leucine, and isoleucine degradation pathway was significant in cecal content and plasma only;
(2) long-chain polyunsaturated fatty-acid metabolism, represented by eicosapentaenoate, *n*-3 docosapentaenoate, and α-linolenate, by the reciprocal fall of three arachidonoyl-glycerophosphocholines, and by the α-linolenic-acid, unsaturated fatty-acid biosynthesis, and glycerophospholipid pathways, against a 1.5-fold higher α-linolenate supply with no preformed eicosapentaenoate or docosahexaenoate;
(3) sphingolipid metabolism, represented by C18 ceramide and the sphingolipid pathway, against our previous report of lower hepatic ceramides [14];
(4) glutathione metabolism, represented by the glutathione and cysteine-and-methionine pathways, against a 25% lower sulfur amino acid supply;
(5) bile-acid metabolism, represented by taurohyodeoxycholate, against our previous report [15], with the qualification that primary bile-acid biosynthesis was significant in cecal content and liver but not in plasma.

Acylcarnitines met neither criterion in the screen; we report them in Section 3.8 and carry them into Section 3.9 because the 70% lower hepatic lipid in these pools [6] raised the question of altered hepatic fatty-acid handling, and we identify them as the one class included in the phenotype rather than the screen. Classes that met the entry criterion without a prior prediction, i.e., citrate, fumarate, and lactate with the tricarboxylic acid cycle, pyruvate, and glyoxylate pathways, the amino acid pathways of Figure 3, and the cofactor and vitamin differences, are not pursued here: in cecal content they largely reflect microbial metabolism of a different substrate stream, and at a single time point their abundance cannot be interpreted as flux. They remain in Tables S3 and S5.

### 3.4. Branched-Chain Amino Acids and Their Keto Acids Accumulate in Cecal Content Despite a Lower Estimated Dietary Supply

**Figure 4:**
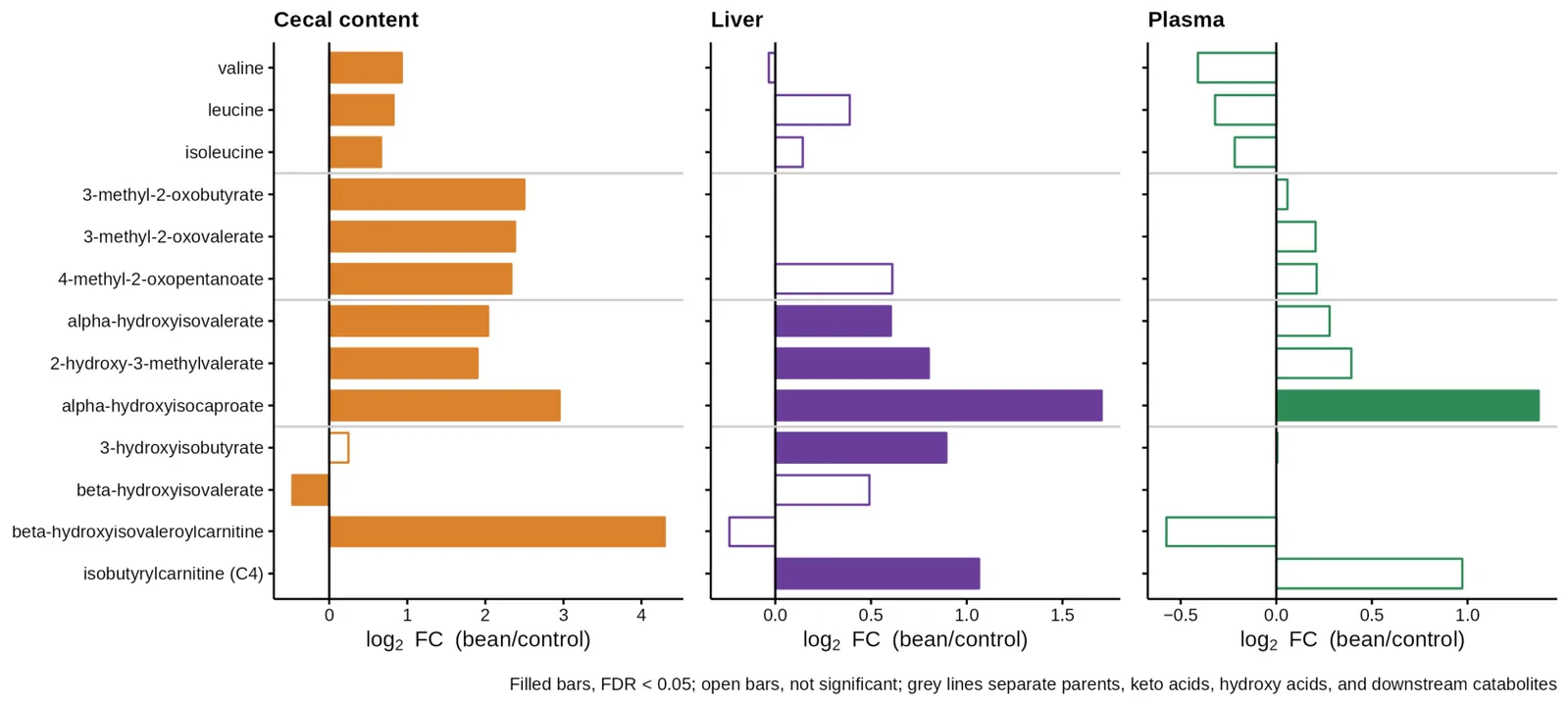
BCAA and their keto and hydroxy acids in cecal content (orange), liver (purple), and plasma (green): log_2_FC (bean/control), n = 7 samples per group; filled bars FDR < 0.05, open bars not significant. The three parent BCAA, the three branched-chain keto acids, the three hydroxy acids, and four downstream catabolites are shown; grey lines separate the classes.

Given that the bean diet, by ingredient-based estimate, supplied 27% less BCAA than the control diet (Table S1), we expected BCAA and their catabolites to be lower in every compartment. This expectation was met only for the parent BCAA in plasma, where the decrease did not reach FDR < 0.05, but was contradicted in cecal content (Figure 4 and Figure S1). In cecal content the parent BCAA were 1.6–1.9-fold higher with bean feeding, the branched-chain keto acids 5.0–5.7-fold higher, and the hydroxy acids 3.7–7.7-fold higher (q-value ≤ 6 × 10^−3^); in liver the downstream hydroxy acids were higher (α-hydroxyisocaproate, which was below detection in 5 of 7 control pools, and α-hydroxyisovalerate, q-value ≤ 8 × 10^−3^); and in plasma the parent valine and leucine were modestly lower (log_2_FC = −0.41 and −0.32; q-value = 0.09 and 0.10). We describe this as cecal accumulation of BCAA and their transamination products despite lower dietary supply. It is compatible with lower ileal digestibility of bean protein followed by microbial transamination in the cecum, but it is equally compatible with differences in absorption, microbial production or consumption, transit time, cecal water content, food intake, or endogenous protein turnover, none of which was measured, and the data do not identify which of these applies.

### 3.5. Long-Chain n-3 and n-6 Fatty Acids Shift Reciprocally in Liver and Plasma

**Figure 5:**
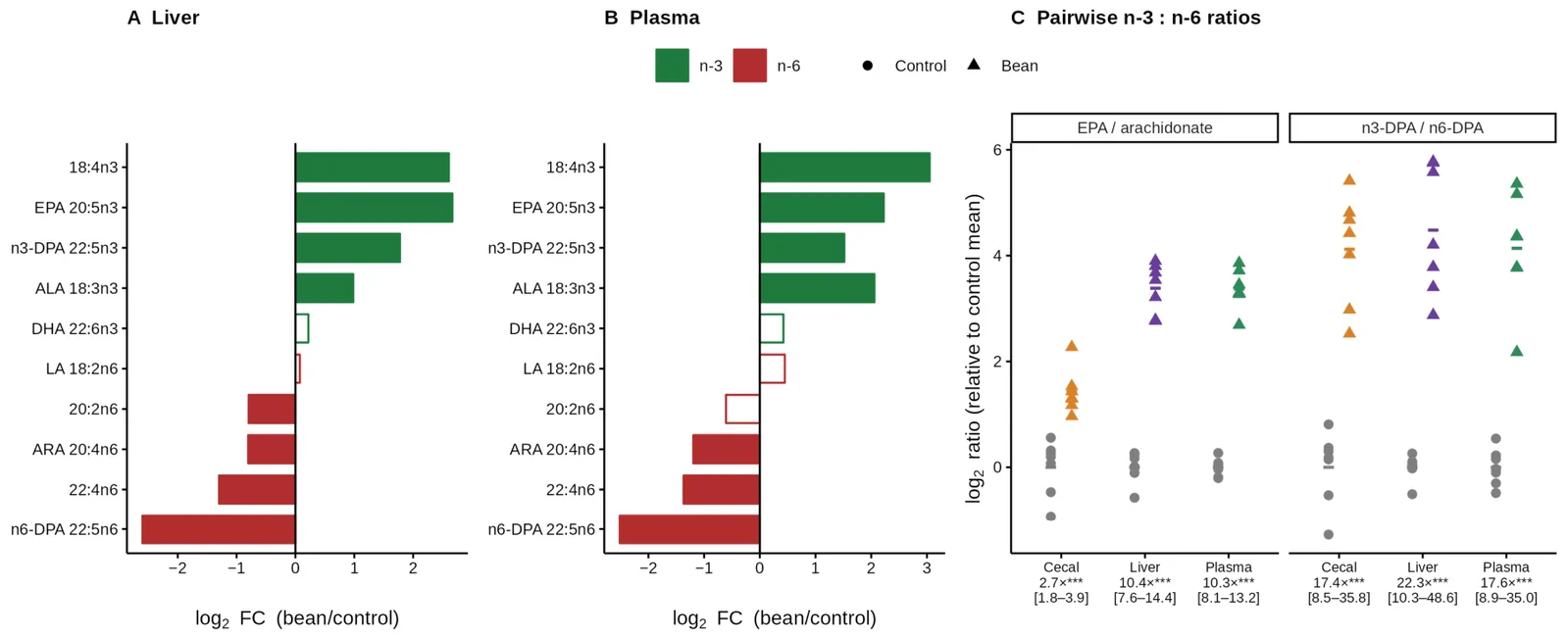
Reciprocal long-chain polyunsaturated fatty-acid shift. (**A**,**B**) log_2_FC (bean/control) for *n*-3 (green) and *n*-6 (red) free fatty acids in liver and plasma, n = 7 samples per group; filled bars FDR < 0.05, open bars not significant. (**C**) Pairwise eicosapentaenoate:arachidonate and *n*-3:*n*-6 docosapentaenoate ratios by matrix, shown as per-sample log_2_ ratios relative to the control mean (horizontal lines, group means), with the between-group FC and 95% CI beneath each matrix; *** *p* < 0.001 (Welch’s *t*-test; all six ratios FDR < 0.001 within matrix). ALA, α-linolenate; ARA, arachidonate; DHA, docosahexaenoate; DPA, docosapentaenoate; EPA, eicosapentaenoate; LA, linoleate; other fatty acids are labeled by chain length:double bonds and *n*-series.

Within the free long-chain polyunsaturated fatty acids, bean feeding was associated with a reciprocal change: *n*-3 species were higher (eicosapentaenoate, log_2_FC = +2.67 in liver and +2.22 in plasma; stearidonate +2.61 and +3.05; *n*-3 docosapentaenoate +1.78 and +1.52) and ≥20-carbon *n*-6 species lower (arachidonate −0.80 and −1.19; adrenate −1.30 and −1.37; *n*-6 docosapentaenoate −2.60 and −2.51), all at FDR < 0.01 (Figure 5A,B and Figure S1). Expressed as pairwise ratios, whose between-group FC is the FC in the molar ratio (Section 2.5), the eicosapentaenoate:arachidonate ratio was 10.4-fold higher in liver (95% CI 7.6–14.4; *p*-value = 1.7 × 10^−8^), 10.3-fold in plasma (8.1–13.2; *p*-value = 1.5 × 10^−8^), and 2.7-fold in cecal content (1.8–3.9; *p*-value = 1.2 × 10^−4^), and the *n*-3:*n*-6 docosapentaenoate ratio was 17–22-fold higher in all three (Figure 5C; Table S6). The 18-carbon *n*-6 precursor linoleate did not differ between diet groups at FDR < 0.05 in any matrix, whereas its ≥20-carbon products were lower (arachidonate:linoleate 0.55-fold in liver, 95% CI 0.46–0.67; 0.32-fold in plasma, 0.25–0.42). On the *n*-3 arm, dietary α-linolenate was, by estimate, 1.5-fold higher in the bean diet, and the stearidonate: α-linolenate ratio, which spans the Δ6-desaturase step, was 3.0-fold higher in liver (2.7–3.4). Neither diet contained preformed eicosapentaenoate or docosahexaenoate. Mead acid (20:3n-9) was lower in liver (q-value = 2 × 10^−4^) and was below detection in 6 of 7 bean plasma samples. Collectively, the pattern is one of higher *n*-3 and lower *n*-6 long-chain products from unchanged linoleate and modestly higher α-linolenate supply. It is consistent with competition between the two precursor families for the shared Δ6/Δ5 desaturase–elongase pathway under a higher *n*-3 precursor supply. Section 3.6 examines whether expression of the shared enzymes changed; enzyme activity was not measured.

### 3.6. Hepatic Desaturase–Elongase Transcripts Are Lower with Bean Feeding; the n-6 Product Indices Follow Them and the n-3 Indices Do Not

If the fatty acid pattern reflected increased host desaturation, transcripts of the shared enzymes would be expected to be higher in the bean group. The genome-wide analysis of these data had reported them lower [6], and the gene-set analysis confirms this. In RNA-seq of the 20 male liver pools, transcripts for *Fads2* (Δ6-desaturase; log_2_FC = −1.16, q-value = 2.3 × 10^−7^), *Elovl5* (−0.99, q-value = 2.2 × 10^−5^), *Fads1* (Δ5-desaturase; −0.71, q-value = 1.8 × 10^−5^), and *Elovl2* (−0.44, q-value = 6.1 × 10^−3^) were lower with bean feeding, and the same differences were present in the 14 pools shared with the metabolomics (Welch *p*-value ≤ 2.8 × 10^−3^; Figure S2A; Table S7). Interpreted alongside the fatty-acid indices of Table S7 (part B) and the ratios of Table S6, these data support four observations:

(1) On the *n*-6 arm the product-to-precursor indices fell in proportion to the transcripts: dihomo-γ-linolenate:linoleate 0.71-fold, arachidonate:dihomo-γ-linolenate 0.78-fold, arachidonate:linoleate 0.55-fold, and adrenate:arachidonate 0.71-fold (Welch *p*-value ≤ 0.014).
(2) On the *n*-3 arm the indices for the same enzymatic steps rose, stearidonate:α-linolenate 3.0-fold and eicosapentaenoate:α-linolenate 3.2-fold, whereas the steps beyond eicosapentaenoate that depend on ELOVL2 fell (*n*-3 docosapentaenoate:eicosapentaenoate 0.55-fold; docosahexaenoate:eicosapentaenoate 0.20-fold), and docosahexaenoate itself did not differ (log_2_FC = +0.22, q-value = 0.30).
(3) Two desaturase-dependent metabolites outside the *n*-3/*n*-6 competition fell with the transcripts: mead acid (20:3n-9), the Δ6/Δ5 product of oleate, was 0.29-fold, and the palmitoleate:palmitate ratio, the metabolite index of stearoyl-CoA desaturase, was 0.55-fold against a *Scd1* transcript change of −1.28 (q-value = 2.2 × 10^−5^).
(4) Across the 14 shared pools the transcript–index correlations were strong (*Fads2* with arachidonate:linoleate, r = +0.87; with eicosapentaenoate:α-linolenate, r = −0.96; *Elovl2* with docosahexaenoate:eicosapentaenoate, r = +0.81), but the two diets form separate clusters on both axes (Figure S2B–D), so these coefficients restate the group difference, and the within-group coefficients at n = 7 were inconsistent in sign (Data S7).

Accordingly, the reciprocal shift of Section 3.5 occurred with transcripts of the desaturase–elongase module at 45–74% of the control level and cannot be attributed to increased transcript abundance. The lower transcripts were not specific to this module: *Scd1*, *Elovl6*, *Fasn*, and *Acaca* were lower by 0.69–1.28 log_2_ units, whereas *Ppara* was modestly lower (−0.28; q-value = 0.07), *Acox1* and *Cyp4a14* did not differ, and *Cpt1a* was lower (−0.53; Table S7). For the other two gene sets, transcripts of the hepatic branched-chain keto-acid dehydrogenase complex and its regulators (*Bckdha*, *Bckdhb*, *Dld*, *Bckdk*, *Ppm1k*) did not differ, *Dbt* and *Bcat2* were modestly higher (+0.20 and +0.31, the latter at 9–12 CPM), and among glutathione-synthesis transcripts *Gclm* (+0.37, q-value = 4.6 × 10^−6^) and *Gsr* (+0.14, q-value = 0.013) were higher while *Gclc*, *Gss*, and *Chac1* did not differ. Two qualifications apply. First, the indices are based on the free fatty-acid pool the platform measures, whereas desaturase indices are conventionally computed on phospholipids or total fatty acids, and the free pool is shaped by lipolysis, esterification, and synthesis. Hence, concordance between transcript and index is consistent with, but does not establish, a change in desaturase activity. Second, transcript abundance is not enzyme activity, and the branched-chain keto-acid dehydrogenase complex is regulated by phosphorylation, so these transcript data constrain, but do not settle, the interpretation of the metabolite axes.

### 3.7. Ceramide-Class Species Are Lower in Liver and Plasma, and C18 Ceramide Is Lower in All Three Compartments

**Figure 6:**
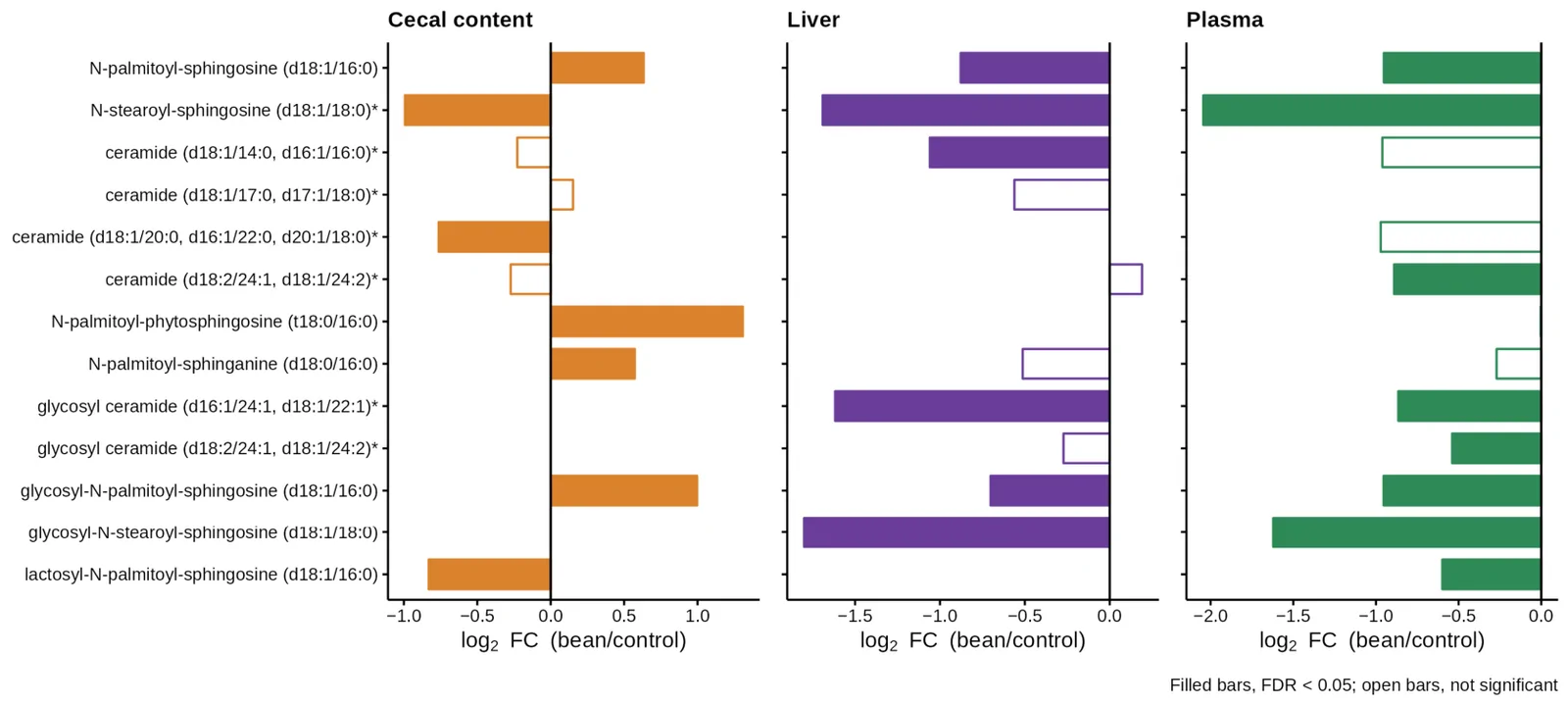
Ceramide-class species in cecal content (orange), liver (purple), and plasma (green): log_2_FC (bean/control), n = 7 samples per group. All ceramide, dihydroceramide, hexosylceramide, and lactosylceramide species detected in at least two matrices are shown (13 species; complete results, including sphingomyelins and sphingosines, in Data S1). Filled bars, FDR < 0.05; open bars, not significant. Negative values indicate lower abundance with bean. *, identified without a purified standard (Section 2.3).

Previously, we have reported that bean consumption lowers hepatic ceramides in this cohort [14]. Consistent with that observation, ceramide-class species were lower with bean feeding in liver and plasma, where the C16 (d18:1/16:0) and C18 (d18:1/18:0) ceramides and their glucosyl conjugates were all lower at FDR < 0.05 (Figure 6); 4 hepatic and 4 plasma species, among them ceramide d18:2/24:1, did not reach FDR < 0.05, so the class-level statement is a predominance and not a uniform decrease. Cecal content differed: C18 ceramide and lactosyl-C16-ceramide were lower, but C16 ceramide, C16 dihydroceramide, and glucosyl-C16-ceramide were higher (FDR < 0.05), and the cecal ceramide composite did not differ by diet (Section 3.9). Free sphingosine and hexadecasphingosine were lower in both cecal content and liver (FDR < 0.01). The most consistent cross-compartment sphingolipid signal is therefore lower C18 ceramide, which was lower in all three matrices, and not the ceramide class as a whole. The liver and plasma values are sphingolipid data from the previously reported run [14] and are shown here so the cecal profile can be interpreted alongside them; the cecal observations are new.

### 3.8. Bile Acids, γ-Glutamyl-Cycle Metabolites, and Acylcarnitines Differ in Abundance by Diet

Bile acids differed in cecal contents and liver, with shifts among primary, conjugated, and secondary or oxo species, whereas the plasma bile-acid pool was largely unchanged; the hepatic bile-acid data are those we reported previously [15], and the cecal profile is new. γ-Glutamyl amino acids and glutathione-related intermediates, including ophthalmate (liver q-value = 6 × 10^−4^, plasma q-value = 2 × 10^−3^), were higher in liver and plasma. These changes reflect metabolite abundance in the γ-glutamyl cycle; they do not measure glutathione synthesis or turnover, and we make no claim about flux. Hydroxybutyrylcarnitines and short- and medium-chain hydroxy- and dicarboxylic acylcarnitines were higher in liver, whereas free 3-hydroxybutyrate did not differ (q-value = 0.37). This pattern is compatible with altered hepatic fatty-acid handling, but acylcarnitine accumulation can reflect incomplete or complete β-oxidation, and the data do not distinguish increased oxidative flux from overflow.

### 3.9. Exploratory Analyses: The Metabolite Classes Differ in Whether Their Diet Association Persists After Adjustment for Adiposity

**Figure 7:**
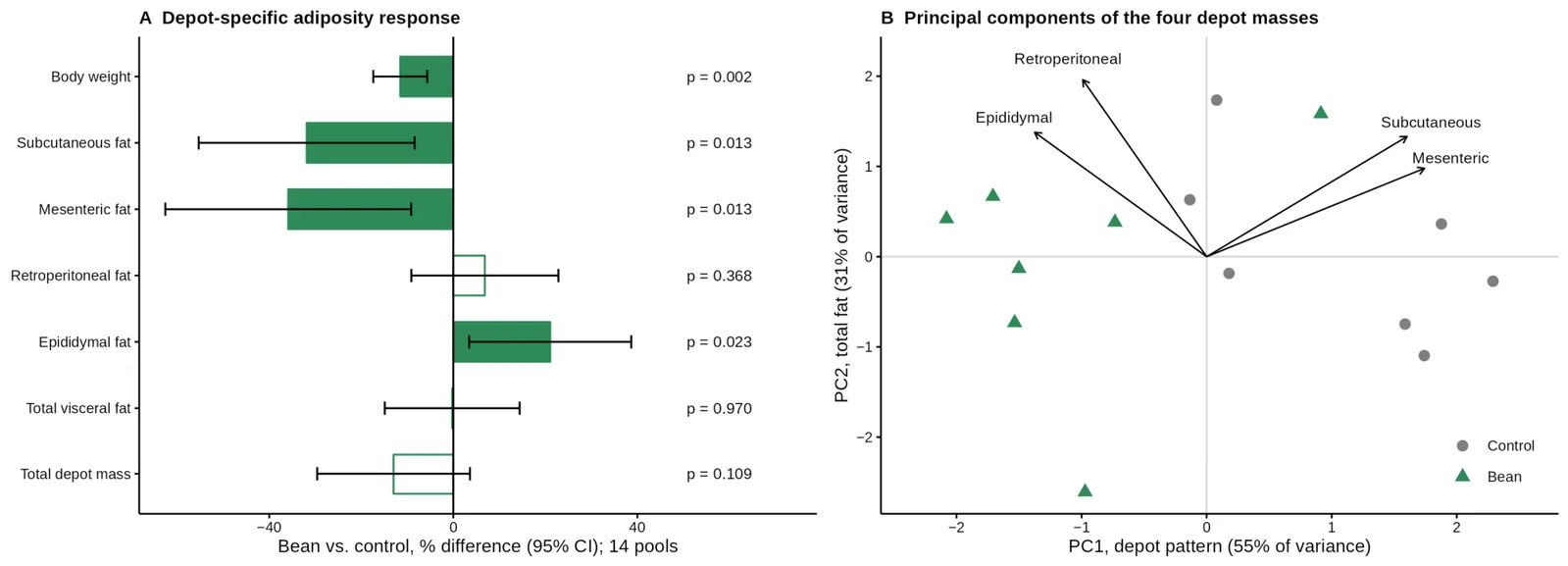
(**A**) Depot-specific adiposity change in the 14 profiled pools, mean percent difference (bean vs. control) with 95% CI and Welch’s *t*-test *p*-value; filled bars *p* < 0.05, open bars not significant. (**B**) Principal component analysis of the four depot masses (z-scored; n = 7 pools per group, phenotype linked to actual donors): scores on the first two components with loading vectors. The first component contrasts subcutaneous and mesenteric with retroperitoneal and epididymal fat and separates the diets; the second, on which all depots load positively, does not.

Linking necropsy weights to the profiled pools showed that the adiposity response was chiefly a redistribution among depots rather than a uniform loss of fat mass. Body weight (−11.5%), subcutaneous fat (−31.9%), and mesenteric fat (−35.9%) were lower with bean feeding, retroperitoneal fat was unchanged, epididymal fat was higher (+21.1%), total visceral fat was unchanged (−0.2%, *p*-value = 0.97), and total depot mass was 13% lower, a difference that did not reach significance in the profiled pools (*p*-value = 0.11; Figure 7A) but did in all 40 animals (−12%, *p*-value = 0.023); the same pattern held in all 40 animals (body weight −10.1%, subcutaneous fat −31.4%, mesenteric fat −32.2%, total visceral fat +1.5%, *p*-value = 0.75; epididymal fat +22.2%). As a four-depot profile, the groups differed (Hotelling’s T^2^ = 21.4, F(4,9) = 4.01, *p*-value = 0.039). Principal component analysis of the four depots (Figure 7B) resolved this profile into a first component (55% of variance) that contrasts subcutaneous and mesenteric fat (loadings +0.55 and +0.60) with retroperitoneal and epididymal fat (−0.34 and −0.47) and separates the diets (control +1.09, bean −1.09; *p*-value = 1.5 × 10^−3^), and a second component (31%) on which all four depots load positively (r = 0.76 with total depot mass) and which does not differ by diet (*p*-value = 0.85). The first component correlates with hepatic lipid (r = 0.91) and body weight (r = 0.83); it is the bean phenotype expressed as a single axis. Hepatic lipid itself was 70% lower in the bean pools (*p*-value = 9.5 × 10^−5^). Therefore, two adjustments were made and are reported separately: for total fat mass, which was lower with bean feeding in the full cohort but not significantly so in the profiled pools, and for the phenotype axis with hepatic lipid, which removes from the diet term everything collinear with the leaner phenotype and is the more stringent of the two.

Against this background we asked, for each metabolite class, whether its association with diet across the three compartments persisted under each adjustment (Table 1; all models, the compartment-level analyses, and the univariate follow-up in Table S4). The results can be summarized in four points:

(1) unadjusted, every class differed by diet across the three compartments (Hotelling–Lawley F(3,10) from 4.0 to 69.5, *p*-value ≤ 0.040), and the diet term persisted for all but the bile-acid class when total depot mass was included (*p*-value ≤ 9.7 × 10^−4^);
(2) under the conservative adjustment for the phenotype axis and hepatic lipid, the branched-chain class retained its diet association after Benjamini–Hochberg correction across the six classes (F(3,8) = 12.8, *p*-value = 2.0 × 10^−3^, q-value = 0.012; leave-one-pool-out maximum *p*-value = 0.011); the glutathione-related class (F(3,8) = 6.5, *p*-value = 0.016, q-value = 0.047; maximum 0.10) and the *n*-3 class (F(3,8) = 5.5, *p*-value = 0.024, q-value = 0.049; maximum 0.067) met the multiplicity-adjusted threshold in the full dataset (q-value < 0.05) but were not robust to the leave-one-pool-out sensitivity analysis (maximum *p*-value above 0.05) and are treated as provisional; and the ketone/acylcarnitine (*p*-value = 0.074, q-value = 0.11), bile-acid (*p*-value = 0.13, q-value = 0.15), and ceramide (*p*-value = 0.20, q-value = 0.20) classes were not retained; with total depot mass and hepatic lipid as the covariates the branched-chain (6.3 × 10^−4^) and glutathione-related (1.6 × 10^−3^) classes were again retained, the ketone/acylcarnitine class sat at the nominal boundary (0.049), and the *n*-3 (0.057), ceramide (0.12), and bile-acid (0.13) classes were not retained;
(3) within the retained classes the compartments contributed unequally: the branched-chain vector holds its sign pattern (cecal and hepatic catabolites higher, plasma parents lower) under every model, the *n*-3 association is carried by liver and plasma and, under the conservative model, the glutathione association by liver, with no cecal contribution to either, and for the ceramide class the diet term of the hepatic composite was *p*-value = 0.041 with hepatic lipid as the only covariate and 0.063 with hepatic lipid and either depot covariate, and the class-level term was not retained in any model containing hepatic lipid (*p*-value = 0.12–0.20; Table S4B), so the ceramide association is not separable from hepatic lipid in these data;
(4) the hepatic bile-acid composite, which shows no diet effect unadjusted (Cohen’s *d* = −0.01), acquires a negative diet coefficient once the phenotype axis is in the model (b = −0.62 to −0.88, *p*-value = 0.028–0.029, Table S4D; first-component *p*-value = 0.008 in the single-covariate model, Supplementary Code report), as would be expected if diet and adiposity acted in opposite directions, although the class-level test does not reach significance under any adjustment.

The permutation *p*-values agreed with the parametric *p*-values for every class (branched-chain 1.7 × 10^−3^; glutathione-related 0.020; *n*-3 0.023; ketone/acylcarnitine 0.080; bile acid 0.12; ceramide 0.20; Table S4G). The class-mean analysis of covariance is interpretable only where the three compartment composites share a direction: for the *n*-3 class, which does, the diet term was retained (F(1,10) = 14.5, *p*-value = 3.4 × 10^−3^; leave-one-pool-out maximum 0.019), and for the ceramide class it was not (*p*-value = 0.062); for the branched-chain and glutathione-related classes the compartments move in opposite directions (plasma parents lower against higher cecal and hepatic catabolites; the cecal glutathione composite lower against higher liver and plasma), the mean cancels (*p*-value = 0.053 and 0.42), and the per-compartment analyses of Table S4D (cecal and hepatic branched-chain composites, *p*-value ≤ 1.2 × 10^−3^ with leave-one-pool-out maxima ≤ 6.2 × 10^−3^; hepatic glutathione composite, *p*-value = 0.010, maximum 0.081) are the one-response checks for those classes. This directional heterogeneity is the reason each class is tested as a vector.

The univariate regressions, in which one covariate enters at a time (Table S4F; Data S3), yielded more nominal diet terms than the class-level analysis retained. With hepatic lipid as the single covariate, the hepatic ceramide (*p*-value = 0.041), hepatic ketone/acylcarnitine (0.010), and plasma *n*-3 (0.001) composites kept a diet term and the hepatic bile-acid composite acquired one (0.017), whereas the class-level test with the same covariate did not retain the ceramide (*p*-value = 0.16), ketone/acylcarnitine (0.054), *n*-3 (0.053), or bile-acid (0.078) class (Table S4B). The class-level test is a joint test of three compartments, to which the cecal composite of each of these classes, and the plasma composite of three of them, contributed no diet term (Table S4D); in addition, the hepatic ceramide, ketone/acylcarnitine, and bile-acid terms did not hold with every pool omitted (leave-one-pool-out maximum *p*-value = 0.23, 0.071, and 0.077). The hepatic ceramide coefficient was −0.80 to −0.92 in each of the nine class-level models, and its standard error rose from 0.16 unadjusted to 0.37 with hepatic lipid in the model, so the estimate was stable and imprecise. For the branched-chain and glutathione-related classes, the two analyses agreed. We do not interpret a nominal diet term for one composite in a single-covariate model as retention of its class.

The cage-aware sensitivity analyses (Section 2.7; Table S8) qualify these results in three ways. First, under the mixed model with a cage random effect and 6 denominator degrees of freedom, the cecal and hepatic branched-chain composites kept their diet terms under the conservative model (b = 1.51, 95% CI 0.54–2.49, *p*-value = 9.1 × 10^−3^; b = 1.39, 95% CI 0.66–2.11, *p*-value = 3.4 × 10^−3^), as did the plasma *n*-3 composite (b = 1.89, 95% CI 0.64–3.13, *p*-value = 0.010); the hepatic glutathione composite sat at the boundary (b = 0.77, 95% CI 0.01–1.53, *p*-value = 0.047); the plasma branched-chain parent (b = −1.20, 95% CI −2.30 to −0.10, *p*-value = 0.037), hepatic ketone/acylcarnitine (b = 1.62, 95% CI 0.36–2.87, *p*-value = 0.020), and hepatic bile-acid (b = −0.88, 95% CI −1.73 to −0.03, *p*-value = 0.044) composites also met *p* < 0.05; and the hepatic *n*-3 (b = 1.39, 95% CI −0.01 to 2.79, *p*-value = 0.052), plasma glutathione (*p*-value = 0.20), and hepatic ceramide (b = −0.77, 95% CI −1.84 to 0.30, *p*-value = 0.13) composites did not. The cage variance was estimated at zero for 12 of the 36 composite models and the intraclass correlation reached 0.85 (plasma ketone/acylcarnitine composite); with eight cages, these estimates are imprecise, and a zero estimate does not establish the absence of a cage effect. Second, leave-one-cage-out separated the classes further. Unadjusted, the cecal and hepatic branched-chain, hepatic glutathione, hepatic and plasma *n*-3, and hepatic ceramide composites kept *p* < 0.05 with every cage omitted (maxima ≤ 0.046), and the class-level tests for the branched-chain, glutathione-related, *n*-3, and ceramide classes did so as well (maxima ≤ 0.017). Under the conservative model no composite kept *p* < 0.05 with every cage omitted: the cecal and hepatic branched-chain and plasma *n*-3 coefficients kept their sign and magnitude in all eight omissions (b = 1.25–1.72, 1.05–1.64, and 1.42–2.75; maximum *p*-value = 0.12, 0.067, and 0.079), and the plasma branched-chain, hepatic ketone/acylcarnitine, and hepatic bile-acid coefficients kept their sign (maximum *p*-value = 0.081, 0.092, and 0.78), whereas the adjusted hepatic glutathione and hepatic *n*-3 coefficients fell to −0.02 and −0.60 when control cage 9 (pools 6, 16, and 26) was omitted (maximum *p*-value = 0.97 and 0.64). At the class level under the conservative model, the branched-chain test reached a maximum *p*-value of 0.055 (cage 9 omitted), the glutathione-related 0.96, the *n*-3 0.20, the ketone/acylcarnitine 0.28, the ceramide 0.60, and the bile-acid 0.97. Third, at the metabolite level, 262, 252, and 133 host metabolites met FDR < 0.05 under the mixed model in cecal content, liver, and plasma, which is 65%, 72%, and 57% of the 401, 352, and 235 that did so by Welch’s test, every one of them within the Welch set, and 20 of the 36 concordant metabolites, among them C18 ceramide, eicosapentaenoate, *n*-3 docosapentaenoate, α-linolenate, α-hydroxyisocaproate, fumarate, and citrate, kept *p* < 0.05 in all three compartments, whereas 2-hydroxy-3-methylvalerate (plasma *p*-value = 0.055) and taurohyodeoxycholate (liver *p*-value = 0.092) did not; the cage variance was estimated at zero for 35%, 54%, and 35% of host metabolites (median intraclass correlation 0.26, 0.00, and 0.18; 90th percentile 0.75, 0.59, and 0.67). These analyses do not add replication, and the adjusted glutathione-related association and the hepatic component of the *n*-3 association depend on one control cage, whereas the plasma *n*-3 coefficient kept its sign and magnitude in every omission; the provisional designation of those two classes rests on this as well as on the leave-one-pool-out result.

The compartment-level analysis, with six responses on 14 observations, is near-saturated and is given in Table S4C as descriptive only. Within-group correlations between composites and subcutaneous fat (Table S4) were imprecise (*n* = 7; median 95% CI width 1.48) and changed sign for 6 of 18 composites when one pool was omitted, so no claim is made from them.

Multivariate analysis of covariance; response, the class composite in cecal content, liver, and plasma (three responses); n = 14 pools; the phenotype is the mean of the two donors of each pool for all three compartments, so that one covariate vector applies to the three responses of a class, although 10 of the 14 plasma composites derive from one of the two donors (Section 2.2); the donor-correct phenotypes are used in Table S4C,E,F. Variance inflation factors in the depot PC1 + hepatic lipid model are 4.8 (diet), 6.0 (depot PC1), and 11.6 (hepatic lipid). The diet term has one degree of freedom, for which the Hotelling–Lawley trace gives an exact F with (3, 10), (3, 9), or (3, 8) degrees of freedom for models with zero, one, or two covariates. Cells give F, the *p*-value, and in brackets the largest diet *p*-value obtained when each pool was omitted in turn. In the depot PC1 + hepatic lipid column, q-value is the Benjamini–Hochberg-adjusted *p*-value across the six classes. Depot PC1, first principal component of the four depot masses (Figure 7B); hepatic lipid, mg/g dry weight. Body weight was not used as a covariate (Section 2.7). Ketone bodies/acylcarnitines were examined on the basis of the hepatic lipid phenotype and not the selection criteria (Section 3.3). Models with the second depot component, the compartment-level analyses, per-compartment diet coefficients, and the univariate regressions with commonality decomposition are in Table S4. The analysis is associative and does not establish independence from adiposity.

## 4. Discussion

The literature on the metabolic effects of common bean is extensive in comparison to that on the organization of those effects across the compartments in which they arise. Because the dietary metabolomics literature focuses primarily on blood [17,18,19,20,21,22,23], the compartment in which a plasma signal originates (the gut, the liver, or the adipose tissue whose mass the diet changes) usually cannot be identified, and we relate the present data to that literature with this limitation in view. Cooked common bean, supplied within an obesogenic diet formulated to nominally equal macronutrient proportions but differing in macronutrient source, and therefore in amino acid and fatty-acid composition, fiber type, and mineral load, was associated with differences in metabolite abundance in every compartment, largest in the gut lumen. Rather than interpret each compartment on its own, we used three-compartment concordance, at the metabolite and pathway levels, as the entry criterion for a metabolite class, and the compositional differences between the diets and our previous reports on this cohort as the source of a prediction against which each class could be judged. Five classes met both criteria: BCAA catabolism, represented in the concordant set by its hydroxy-acid products, which accumulated in the cecum against a lower dietary supply; long-chain polyunsaturated fatty-acid metabolism, in which the *n*-3 products rose and the arachidonoyl species fell while hepatic desaturase–elongase transcripts were lower, not higher; glutathione metabolism, significant as a pathway in all three compartments against a lower sulfur amino acid supply; sphingolipid metabolism, represented by C18 ceramide and drawn from the run of our earlier hepatic report; and bile-acid metabolism, represented by a single concordant species. Acylcarnitines, which met neither criterion, were examined because of the hepatic lipid phenotype. We are not aware of a previous report of the cecal luminal metabolome in a cooked-bean feeding model, and the present one does not clarify where the response originates or whether any element contributes to the leaner phenotype. Below we state what concordance does and does not establish, place each class according to its behavior under adjustment for adiposity, interpret the classes in that order, and define what a single-time-point, whole-food design can establish.

### 4.1. Concordance Is Not Direction

The larger cecal response, the liver–plasma concordance (ρ = 0.48), and the 36 metabolites moving in the same direction across all three compartments are consistent with a gut-derived signal that extends to systemic metabolism. They are equally consistent with the diet acting on each compartment in parallel, given that all three were sampled at the same terminal time point, and the weak cecal–systemic correlation (ρ ≈ 0.12) argues against any simple transfer of the cecal profile to the periphery. We therefore present gut-to-systemic propagation as a hypothesis, not a finding, and these data provide no basis for the assertion that systemic changes are downstream of cecal ones. The cecal metabolome does contain a fermentation-associated signature (dipeptides, polyamines, nucleotides, branched-chain keto acids) consistent with an altered microbial community acting on an altered substrate stream, in the same animals in which we have reported dose-dependent restructuring of that community [16].

### 4.2. Adjustment for Adiposity Bounds the Driver-Versus-Bystander Question but Does Not Settle It

Because common bean lowers adiposity, a metabolite that also covaries with fat mass could reflect the leaner phenotype or contribute to it. Five observations bear on this. First, the adiposity response was a redistribution: subcutaneous and mesenteric fat were lower and epididymal fat higher, total depot mass was not significantly lower in the profiled pools (although it was 12% lower in the full cohort), and the first principal component of the depots, which captures that redistribution and correlates with hepatic lipid, is the phenotype itself (Section 3.9). Second, the diet term for every class except bile acid persisted when we included total fat mass in the model. Third, under the conservative adjustment, for the phenotype axis and hepatic lipid together, the classes separated into tiers: the branched-chain class retained its diet association after correction for multiplicity and with every pool removed; the glutathione-related and *n*-3 classes met the multiplicity-adjusted threshold (q-value = 0.047 and 0.049) but were not robust to the leave-one-pool-out analysis (maximum *p*-value = 0.10 and 0.067), and are provisional; and the ketone/acylcarnitine, ceramide, and bile-acid classes were not retained, the ceramide association being removed by hepatic lipid specifically. Fourth, the hepatic bile-acid composite acquires a negative diet coefficient once the phenotype axis is in the model, as would be expected if diet and adiposity acted in opposite directions (Section 4.3). Fifth, under a model that accounts for cage membership, the branched-chain association persisted in direction and magnitude with every cage omitted, as did the plasma *n*-3 association, whereas the adjusted glutathione-related association and the hepatic *n*-3 association depended on one control cage, and the ceramide, ketone/acylcarnitine, and bile-acid class-level associations were not retained (Section 3.9). We draw a limited conclusion from this: the branched-chain difference, and provisionally the glutathione-related and *n*-3 differences, retained their association with diet after adjustment for fat mass and for the phenotype axis available in this study, and the ceramide and bile-acid differences cannot be separated from that axis.

We do not conclude that the former are independent of adiposity or that they participate in the phenotype, for five reasons:

(1) adiposity is a post-allocation variable that may lie on the pathway from diet to metabolite, and conditioning on it cannot assign the variance it shares with diet;
(2) conditioning on a post-allocation variable can also attenuate a real diet effect or induce one that is not there, so a coefficient that appears only after adjustment is as consistent with conditioning on a mediator as with opposing biology;
(3) the class-level tests have 8–10 denominator degrees of freedom, diet was delivered by 4 cages per diet, and the cage-aware analyses are approximate and depend on single cages in several cases;
(4) within-group correlations with 7 observations are too imprecise to interpret;
(5) no adjustment in a cross-sectional design can establish causal order.

The prior question, whether the bean metabolome reflects or participates in the phenotype, remains open, and the present data identify which metabolite classes merit carrying into a design that can answer it.

### 4.3. The Metabolite Classes

**Branched-chain amino acids.** The BCAA class is best described as a distribution pattern rather than a change in catabolic rate: dietary BCAA was, by estimate, 27% lower in the bean arm, plasma BCAA were modestly lower in the direction of intake, and cecal keto and hydroxy acids were 3.7–7.7-fold higher against that lower supply (Section 3.4). Lower ileal digestibility of bean protein with microbial transamination in the cecum is one hypothesis that would produce this pattern [59,60,61,62]; direct assessment would require ileal digestibility measurement, cecal transaminase activity, or isotopic tracing of dietary leucine, none of which was performed, and the alternatives listed in Section 3.4 are not excluded. The hepatic transcripts bear on one alternative: a hepatic bottleneck in branched-chain keto-acid oxidation would be expected to raise the hepatic hydroxy acids, and transcripts of the dehydrogenase complex, apart from a modest rise in the E2 subunit Dbt (+0.20 log_2_), and of its kinase and phosphatase did not differ (Section 3.6), which weighs against a transcriptional change at that step; the complex is regulated by phosphorylation [63], however, and its activity was not measured. This is the one class whose diet association was retained under every adjustment for adiposity, after correction for multiplicity, and with every pool removed (Section 3.9).

**Long-chain polyunsaturated fatty acids.** The reciprocal *n*-3/*n*-6 shift is interpreted alongside the transcript data of Section 3.6. Linoleate supply and tissue linoleate were unchanged while the ≥20-carbon *n*-6 products fell, estimated α-linolenate supply rose 1.5-fold, and the two precursor families compete for the same Δ6 and Δ5 desaturases and elongases [64,65,66], so the pattern is what precursor competition would predict; what the transcripts add is that those of the shared enzymes were lower (45–61% of control for *Fads2*, *Elovl5*, and *Fads1*), so increased desaturase–elongase transcript abundance cannot account for the pattern. We interpret the *n*-3 pattern, in which stearidonate and eicosapentaenoate accumulated while the ELOVL2-dependent steps beyond eicosapentaenoate fell, as the consequence of a reduced enzyme pool with a preference for *n*-3 substrates operating on a higher α-linolenate supply, with elongation beyond eicosapentaenoate as the bottleneck; this is a hypothesis, and the design does not test it. The magnitude of the eicosapentaenoate rise exceeds what a 26% decrease in *Elovl2* transcript would predict, and reduced peroxisomal β-oxidation of eicosapentaenoate, reduced esterification of eicosapentaenoate into complex lipids, and an *n*-3 source not captured by the ingredient estimate are alternatives that the data do not exclude. *Fads1*, *Fads2*, and *Elovl5* are transcriptional targets of both SREBP-1c and PPARα [67], and the transcript data do not separate the two routes cleanly: lower *Cpt1a* and a modestly lower *Ppara* are compatible with reduced PPARα signaling, although the canonical targets *Acox1* and *Cyp4a14* did not differ, whereas the parallel fall of *Scd1*, *Elovl6*, *Fasn*, and *Acaca*, and of the stearoyl-CoA desaturase metabolite index with *Scd1*, is consistent with reduced SREBP-1c activity. Neither PPARα nor SREBP-1c activity was measured. The dietary fatty-acid values remain ingredient-based estimates, enzyme activity and flux were not measured, and whether the lower transcripts reflect the lower carbohydrate quality of the bean diet, its higher polyunsaturated fatty-acid supply, or the leaner phenotype cannot be decided from these data. The *n*-3 class met the multiplicity-adjusted threshold after adjustment for the phenotype axis and hepatic lipid (q-value = 0.049) but was not robust to the leave-one-pool-out analysis, and the adjusted hepatic association was removed by omitting one control cage (Section 3.9); the association is carried by liver and plasma and not by cecal content; the transcripts were not adjusted for adiposity, so the mechanistic constraint they add is not independent of fat mass.

**Glutathione-related metabolites.** Higher γ-glutamyl amino acids and ophthalmate are consistent with the lower sulfur amino acid content of the bean diet (25% less by estimate but above the National Research Council requirement for the mouse [28]) and with the cysteine-conservation response described under methionine restriction [68,69,70], but they are abundances, not fluxes; the modestly higher *Gclm* and *Gsr* transcripts, with *Gclc* and *Gss* unchanged (Section 3.6), are consistent with a small adjustment of glutathione-synthesis capacity and do not demonstrate it. The class met the multiplicity-adjusted threshold under the conservative model (q-value = 0.047) but was not robust to the leave-one-pool-out analysis or to the omission of one control cage, and total fat mass was itself a significant covariate, so these metabolites were associated with fat mass and with diet in the same model (Section 3.9).

**Ceramides.** Lower hepatic and plasma long-chain ceramides, species implicated in hepatic lipotoxicity and insulin resistance [71,72,73], are those of our previous report in this cohort [14], re-examined here alongside the cecal profile, and provide a directional correlate of the reduced hepatic lipid reported in this model; given the absence of a dietary sphingolipid source, we judge them a plausible candidate for a metabolic, as opposed to dietary, signal. The cecal profile is mixed (C18 lower, C16 higher), which is consistent with a compartment where sphingolipids are also of microbial and epithelial origin, and C18 ceramide is the only species lower in all three compartments. The class retained its diet association after adjustment for total fat mass and for the depot axis alone, but not once hepatic lipid entered the model (class-level *p*-value = 0.16 with hepatic lipid alone and 0.20 with the depot axis added; hepatic composite *p*-value = 0.041 and 0.063), which indicates how closely ceramide and hepatic lipid covary in this design (Section 3.9). The nominal diet term of the hepatic composite with hepatic lipid as the single covariate did not hold when one pool was omitted (leave-one-pool-out maximum *p*-value = 0.23), and we do not rest a conclusion on it. We draw no conclusion about which precedes the other.

**Bile acids.** Bile-acid restructuring, which supplies gut- and microbiome-derived ligands of the farnesoid X receptor (FXR) and Takeda G protein-coupled receptor 5 (TGR5) with plausible forward effects on hepatic lipid metabolism [74,75,76], is drawn from the run of our previous report [15], and the cecal profile is the new element. The hepatic bile-acid composite behaved unlike every other class: it showed no diet effect unadjusted, a negative association with subcutaneous fat and hepatic lipid, and a negative diet coefficient that appeared only once the depot axis or hepatic lipid was in the model (Section 3.9), the signature of two predictors acting in opposite directions. The adiposity direction has precedent in rodents: in C57BL/6J mice, diet-induced obesity lowers hepatic bile-acid content, reversibly with weight loss [77], through suppression of hepatic synthesis by intestinal FXR–FGF15 signaling and reduced ileal reabsorption in mice and rats [78,79], depletion of secondary species in the enterohepatic pool of mice with steatohepatitis [80], and, in rat models of steatosis, reduced hepatocyte uptake-transporter expression with increased sinusoidal efflux [81,82]; the human literature is discordant on direction [83], so we state the sign as the rodent one. If adiposity-dependent regulation lowers hepatic bile acids in the fatter control animals, and bean feeding lowers them directly through fiber-mediated sequestration and fecal excretion or microbial deconjugation [15], the two effects would cancel in the between-group comparison, and the absence of a diet effect would be a cancellation rather than an absence. That is a hypothesis from a two-predictor model with 14 observations, and the design cannot separate the two paths. A diet coefficient that appears only once a post-allocation covariate enters the model is also as consistent with conditioning on a mediator, which can induce an association where none exists, as with opposing biology, and the data do not distinguish these. Its value here is as a discriminant. The procedure of Section 3.9 does not label every composite as diet-associated, and the class whose variance adiposity explains is the one for which a forward causal arrow from diet is least supported by these data, whatever its plausibility on receptor grounds.

**Acylcarnitines.** The hepatic rise in hydroxybutyrylcarnitines and other short- and medium-chain acylcarnitines, with free 3-hydroxybutyrate unchanged, is compatible with altered fatty-acid oxidation [84,85] but cannot be interpreted as increased complete oxidation without flux measurement. The acylcarnitine class is the most entangled with adiposity among those examined; it is also the one class examined on the basis of the hepatic lipid phenotype rather than the concordance screen (Section 3.3), and we weight it accordingly; its class-level diet association was borderline under the conservative adjustments (Section 3.9). Given that all of these classes are implicated by patterns of relative abundance at a single time point, the hypothesis-driven extension of these data-driven observations will require quantitative metabolite concentrations and enzyme activities coupled with kinetic modeling and pathway flux analysis.

### 4.4. Strengths and Limitations

Strengths include a description of the cecal luminal metabolome in this model, joint profiling of three compartments in the same animals, donor-linked phenotypes, hepatic transcript data from the same pools, and leave-one-pool-out and leave-one-cage-out sensitivity analyses. Limitations are: (1) one terminal time point in males; (2) reuse of previously reported liver and plasma profiling data [14,15], rather than independent replication; (3) seven profiled samples per diet, some single-donor plasma samples, and adiposity-ranked pooling without regard to cage. Diet was assigned to 4 cages per diet; joint tests remain pool-based, and cage-aware models are approximate with eight cages. The profiled pools were selected by adiposity and sample availability, potentially restricting the covariate range; (4) nominal macronutrient formulation without finished-diet assays or measured food intake, so actual protein, amino acid, α-linolenate, fiber, and mineral exposures are uncertain; (5) composite membership selected for directional coherence in the same 14 samples, making unadjusted class tests partly circular, with limited degrees of freedom and post-allocation covariate adjustment; (6) pooled hepatic transcript measurements without protein, enzyme-activity, or flux validation; and (7) cecal bulk and dry-matter effects were not quantified in these analyses, so apparent differences in relative abundance may partly reflect luminal water content or sample composition. These findings warrant measured diet composition and intake, both sexes, longitudinal sampling, and targeted metabolite and pathway-flux assays.

## 5. Conclusions

Cooked common bean, supplied within an obesogenic diet formulated to nominally match macronutrient proportions but differing in macronutrient source, was associated with differences in metabolite abundance in cecal content, liver, and plasma, largest in the cecal lumen, which had not previously been profiled in this model. Using three-compartment concordance as the entry criterion and the diets’ compositional differences or our previous reports as the source of a prediction, five metabolite classes met both criteria: BCAA catabolism, in which the keto and hydroxy acids accumulated in the cecum against a lower dietary supply; long-chain polyunsaturated fatty-acid metabolism, in which *n*-3 products rose and *n*-6 products fell while hepatic transcripts of the shared desaturase–elongase enzymes were lower, not higher, so that precursor competition at a reduced enzyme pool remains a hypothesis that the design does not test; glutathione metabolism; sphingolipid metabolism, re-examined from the run of our earlier hepatic report; and bile-acid metabolism. Adjustment for adiposity ordered these classes. Total fat mass was not significantly lower in the profiled pools, although it was 12% lower in the full cohort; the adiposity response was chiefly a redistribution among depots that covaried with hepatic lipid. After adjustment for that phenotype axis and hepatic lipid, the branched-chain class retained its diet association across all three compartments after correction for multiplicity; the glutathione-related class and, in liver and plasma, the *n*-3 class met the multiplicity-adjusted threshold but not the leave-one-pool-out criterion and are provisional; the ceramide class did not separate from hepatic lipid; and the hepatic bile-acid composite showed diet and adiposity coefficients of opposite sign, a pattern we interpret only as a hypothesis. Under a model that accounts for cage membership, the adjusted branched-chain association kept its direction and magnitude with every cage omitted, as did the adjusted plasma *n*-3 association, whereas the adjusted glutathione-related and hepatic *n*-3 associations did not. Concordance is not direction: propagation from the gut, a role for any metabolite in the leaner phenotype, altered enzyme activity, and changes in glutathione or fatty-acid-oxidation flux are hypotheses that a single-time-point, whole-food design cannot test. Designs that follow must sample the cecum before the liver changes, vary one constituent class of the bean matrix at a time with assayed composition, and include both sexes. With that in hand, the cecal metabolome and the branched-chain, *n*-3, and glutathione-related classes identified here can inform studies that establish which effects of common bean consumption improve human well-being and by what route.

## Figures and Tables

**Table 1:** Class-level multivariate analysis of covariance: diet term for each metabolite class across cecal content, liver, and plasma, unadjusted and adjusted for adiposity.

Metabolite Class	Diet Only	+ Total Depot Mass	+ Depot PC1	+ Depot PC1 + Hepatic Lipid	+ Total Depot Mass + Hepatic Lipid
**Branched-chain amino acids**	69.5; 5 × 10^−7^ [3 × 10^−6^]	85.4; 6 × 10^−7^ [4 × 10^−6^]	25.8; 9 × 10^−5^ [2 × 10^−3^]	12.8; 2.0 × 10^−3^ [0.011]; q-value = 0.012	18.1; 6.3 × 10^−4^ [8.1 × 10^−3^]
**Glutathione-related**	33.5; 1.6 × 10^−5^ [7 × 10^−5^]	38.4; 1.9 × 10^−5^ [1 × 10^−4^]	10.9; 2.3 × 10^−3^ [9.1 × 10^−3^]	6.5; 0.016 [0.10]; q-value = 0.047	13.7; 1.6 × 10^−3^ [0.021]
***n*-3 polyunsaturated fatty acids**	23.9; 7 × 10^−5^ [3 × 10^−4^]	16.5; 5.4 × 10^−4^ [2.0 × 10^−3^]	7.3; 8.8 × 10^−3^ [0.016]	5.5; 0.024 [0.067]; q-value = 0.049	3.8; 0.057 [0.38]
**Ketone bodies/acylcarnitines**	5.0; 0.022 [0.045]	14.6; 8.3 × 10^−4^ [0.012]	9.1; 4.4 × 10^−3^ [0.063]	3.4; 0.074 [0.23]; q-value = 0.11	4.1; 0.049 [0.22]
**Ceramides**	18.5; 2.1 × 10^−4^ [7 × 10^−4^]	14.0; 9.7 × 10^−4^ [3.5 × 10^−3^]	5.5; 0.020 [0.073]	1.9; 0.20 [0.66]; q-value = 0.20	2.6; 0.12 [0.64]
**Bile acids**	4.0; 0.040 [0.081]	2.5; 0.12 [0.26]	3.0; 0.088 [0.29]	2.6; 0.13 [0.48]; q-value = 0.15	2.6; 0.13 [0.41]

## Data Availability

The metabolomic and transcript data underlying the results are provided as Supplementary Files: Data S1–S4 (analysis tables), Data S5 (diet workbook), Data S6 (raw Metabolon data tables for all three matrices), Data S7 (hepatic transcript tables), Data S8 (the per-animal necropsy and hepatic-lipid files and the genome-wide liver CPM export read by the code), and Data S9 (per-metabolite cage-aware results), together with the analysis code (Supplementary Code), which can be run from these files. The RNA-seq data are deposited in the Gene Expression Omnibus under accession GSE227120. Data beyond those provided as Supplementary Files are available from the corresponding author on reasonable request.

## Supplementary Materials

The following supporting information can be downloaded at: https://www.mdpi.com/article/10.3390/nu18213669/s1, Table S1: diet formulation (male control and bean diets) and ingredient-based estimates of amino acid and fatty-acid composition; Table S2: differential-abundance summary by matrix; Table S3: the 36 host metabolites concordant across all three compartments; Table S4: depot principal components, class-level and compartment-level MANCOVA for all models with leave-one-pool-out sensitivity, per-compartment diet coefficients, and the univariate regression of all 18 composites on diet and each adiposity covariate with coefficients, CIs, commonality decomposition, and diagnostics; Table S5: KEGG enrichment and pathway-topology results by matrix; Table S6: pairwise polyunsaturated fatty-acid ratios by matrix; Table S7: hepatic transcripts for five gene sets and the matched fatty-acid indices; Table S8: cage-aware sensitivity analysis (multiple-membership mixed model with leave-one-cage-out) for the composites, the class-level models, the per-metabolite comparisons, and the concordant set; Figure S1: dietary supply versus tissue response for polyunsaturated fatty acids and branched-chain amino acids; Figure S2: hepatic transcripts and their relation to the fatty-acid indices in the 14 shared pools; Data S1: per-metabolite differential abundance (Welch and Mann–Whitney), exposure-marker classification with rule, missing-value counts for each matrix, and the plasma single-donor sensitivity analysis (COSU-01-23VW_Bean_vs_Control_metabolomics_analysis_R1.xlsx); Data S2: MetaboAnalyst cross-tissue summary (COSU-01-23VW_MetaboAnalyst_crosstissue_summary.xlsx); Data S3: pooling crosswalk with the cage of every donor, cage key, donor-correct phenotypes, composite membership, and per-pool composite scores (COSU-01-23VW_perpool_matrix_R2.xlsx); Data S4: PCA scores and log_2_ Pareto-scaled matrices (COSU-01-23VW_PCA_scores_and_abundance_matrix.xlsx); Data S5: diet formulation workbook (COSU-01-23VW_Diet_workbook_DataS5.xlsx); Data S6: Metabolon data tables (peak area, batch-normalized, volume-normalized for plasma, and imputed) for cecal content, liver, and plasma (COSU-01-23VW+ CECAL DATA TABLES.XLSX; COSU-01-23VW+ LIVER DATA TABLES.XLSX; COSU-01-23VW+ PLASMA DATA TABLES.XLSX); Data S7: hepatic RNA-seq gene-set results, per-pool CPM, fatty-acid indices, and correlations, with the complete male differential-expression table (COSU-01-23VW_liver_transcripts_DataS7.xlsx); Data S8: per-animal source files read by the Supplementary Code, namely necropsy tissue weights, hepatic dry matter and lipid, and the genome-wide liver CPM export (CSU-236-237-239_DRS_Male_and_Female_Necropsy_Tissue_Weights_5-3-26.xlsx; CSU-236-239 LIVER DRY MATTER and LIPID QUANTIFICATION-MALES SYSTAT.xlsx; Expr_Browser_-_70v0_FM_DRS_-_Liver_GE_condensed.xlsx); Data S9: per-metabolite results of the cage-aware sensitivity analysis for all host metabolites in the three matrices (COSU-01-23VW_cage_sensitivity_DataS9.xlsx); Supplementary Code: R Markdown document and rendered report reproducing the analyses, figures, and tables, with session information listing exact package versions. Supplementary Methods: procedural detail (Sections SM1–SM9), opening with a table that locates each reporting item (Table SM1) (Supplementary_Methods_R2_vs5.docx); Table SM2: Gene sets examined. README: instructions for running the Supplementary Code (README_Supplementary_Code.txt). File names in the Supplementary Materials document match the archive.

## Abbreviations

The following abbreviations are used in this manuscript:

BCAA	branched-chain amino acids
CI	confidence interval
CMTRX	client matrix technical replicate (pooled quality-control sample)
CPM	counts per million (RNA-seq)
EDTA	ethylenediaminetetraacetic acid
FC	fold change
FDR	false-discovery rate
FXR	farnesoid X receptor
HD4	Metabolon Global Discovery platform (fourth generation)
HMDB	Human Metabolome Database
IACUC	Institutional Animal Care and Use Committee
KEGG	Kyoto Encyclopedia of Genes and Genomes
MANCOVA	multivariate analysis of covariance
MS/MS	tandem mass spectrometry
PCA	principal component analysis
PLS-DA	partial least-squares discriminant analysis
PPARα	peroxisome proliferator-activated receptor-α
RNA-seq	RNA sequencing
SREBP-1c	sterol regulatory element-binding protein-1c
TGR5	Takeda G protein-coupled receptor 5
UPLC	ultra-performance liquid chromatography
VIP	variable importance in projection
