# Supplementary material for: Concordant Metabolite Differences Across Cecal Content, Liver, and Plasma in Mice Fed an Obesogenic Diet Containing Common Bean (Phaseolus vulgaris L.): A Multi-Compartment Metabolomics Study
Source: Nutrients. 2026 Nov 5;18(21):3669. doi: 10.3390/nu18213669 (PMC13765519; doi:10.3390/nu18213669)
Supplement: Supplementary file 1 [file nutrients-18-03669-s001.zip › nutrients-4490632-supplementary/Supplementary_Code/DRS_multicompartment_metabolomics.html]

Concordant Metabolite Differences across Cecal Content, Liver, and Plasma in Mice Fed an Obesogenic Diet Containing Common Bean (Phaseolus vulgaris L.): A Multi-Compartment Metabolomics Study


Code 

- Show All Code
- Hide All Code

# Concordant Metabolite Differences across Cecal Content, Liver, and Plasma in Mice Fed an Obesogenic Diet Containing Common Bean (*Phaseolus vulgaris* L.): A Multi-Compartment Metabolomics Study

### Data processing, statistical analysis, figures, and tables

#### 2026-10-22

# 1 Setup

```
suppressPackageStartupMessages({
  library(readxl); library(writexl); library(dplyr); library(tidyr); library(tibble)
  library(stringr); library(ggplot2); library(patchwork); library(scales)
  library(knitr); library(kableExtra); library(limma); library(globaltest); library(lmtest)
})
# Paths are relative to this Rmd (knitr evaluates chunks in its folder). The data files sit beside it or, in the
# supplementary archive, one level up; the MetaboAnalyst library and the outputs sit beside it in both layouts.
data_dir <- if (file.exists("COSU-01-23VW_Diet_workbook_DataS5.xlsx")) "." else ".."
out_dir  <- "output"
dir.create(file.path(out_dir, "figures"), recursive = TRUE, showWarnings = FALSE)
dir.create(file.path(out_dir, "tables"),  recursive = TRUE, showWarnings = FALSE)
seed <- 20260924

mats     <- c(CECAL = "Cecal content", LIVER = "Liver", PLASMA = "Plasma")
mat_col  <- c(CECAL = "#D9822B", LIVER = "#6A3D9A", PLASMA = "#2E8B57")
grp_col  <- c(Control = "grey50", Bean = "#2E8B57")
grp_shp  <- c(Control = 16, Bean = 17)

theme_set(theme_classic(base_size = 10) +
            theme(plot.title = element_text(face = "bold", size = 10), legend.position = "top"))

# Table printing helper
p_cols <- "^(p|q|FDR|holm|P[.]Value|adj[.]P[.]Val)$|^(p|q|FDR)_|_p$|_p_14$"
ktab <- function(x, caption = NULL, digits = 3, ...) {
  x <- as.data.frame(x)
  for (j in grep(p_cols, names(x), ignore.case = TRUE)) if (is.numeric(x[[j]])) x[[j]] <- formatC(x[[j]], format = "g", digits = 2)
  kable(x, caption = caption, digits = digits, ...) |>
    kable_styling(bootstrap_options = c("striped", "condensed"), full_width = FALSE, font_size = 12)
}
fmt_p <- function(p) ifelse(is.na(p), NA, ifelse(p < 0.001, formatC(p, format = "e", digits = 1), formatC(p, format = "f", digits = 3)))
save_fig <- function(p, name, w, h) {
  ggsave(file.path(out_dir, "figures", paste0(name, ".png")), p, width = w, height = h, dpi = 300)
  ggsave(file.path(out_dir, "figures", paste0(name, ".pdf")), p, width = w, height = h)
}
```

# 2 Diet composition

The diet workbook
(`COSU-01-23VW_Diet_workbook_DataS5.xlsx`) gives the
formulation of the 0% and 70% (nominal protein-from-bean) diets and the
ingredient protein and fat fractions, amino-acid profiles, fatty-acid
profiles, and NRC requirements used to estimate amino-acid and
fatty-acid supply. The finished diets were not assayed; the estimates
below are recomputed from these inputs as formulation × ingredient
protein or fat fraction × published profile, plus free amino acids.

```
d5_file <- file.path(data_dir, "COSU-01-23VW_Diet_workbook_DataS5.xlsx")
form <- read_excel(d5_file, "Formulation", skip = 1) |> rename(ingredient = 1, d0 = 2, d70 = 3) |>
  filter(!is.na(d0), ingredient != "Total") |> mutate(d0 = as.numeric(d0), d70 = as.numeric(d70))
form |> rename(`0% bean` = d0, `70% bean` = d70) |> ktab(caption = "Table S1, part A. Diet formulation, g/100 g diet", digits = 2)
```

Table S1, part A. Diet formulation, g/100 g diet

| ingredient | 0% bean | 70% bean |
| --- | --- | --- |
| Casein (acid) | 23.50 | 7.10 |
| Cooked whole bean powder (white kidney) | 0.00 | 40.80 |
| Corn starch | 15.90 | 2.00 |
| Dextrose | 7.20 | 7.20 |
| Sucrose | 25.00 | 21.00 |
| Cellulose (Solka-Floc) | 7.50 | 1.00 |
| Corn oil | 11.32 | 11.32 |
| Anhydrous butter fat | 4.24 | 4.24 |
| AIN-93G mineral mix (Dyets 210025) | 3.80 | 3.80 |
| AIN-93 vitamin mix (Dyets 310025) | 1.10 | 1.10 |
| DL-Methionine | 0.30 | 0.30 |
| L-Tryptophan | 0.00 | 0.06 |
| Choline bitartrate | 0.20 | 0.20 |

```
c(total_0 = sum(form$d0), total_70 = sum(form$d70))
```

```
##  total_0 total_70 
##   100.06   100.12
```

```
est <- as.data.frame(read_excel(d5_file, "AA_FA_estimates", col_names = FALSE))
block <- function(hdr_col1, hdr_col2) {  # rows following a header row until the first blank row
  h <- which(est[[1]] == hdr_col1 & est[[2]] == hdr_col2); r <- h + 1; out <- c()
  while (r <= nrow(est) && !is.na(est[r, 1])) { out <- c(out, r); r <- r + 1 }
  est[out, ]
}
ingr <- block("Ingredient", "Protein g/100 g") |> transmute(ingredient = `...1`, protein = as.numeric(`...2`) / 100, fat = as.numeric(`...3`) / 100)
aa_prof <- block("Amino acid", "Casein") |> transmute(aa = `...1`, casein = as.numeric(`...2`), bean = as.numeric(`...3`))
fa_prof <- block("Fatty acid", "Corn oil") |> transmute(fa = `...1`, corn = as.numeric(`...2`), butter = as.numeric(`...3`), bean = as.numeric(`...4`))
nrc <- block("Amino acid(s)", "0% bean, g/kg") |> transmute(aa = `...1`, nrc = as.numeric(`...4`), wb_0 = as.numeric(`...2`), wb_70 = as.numeric(`...3`)) |> filter(!is.na(nrc))
as_tibble(ingr) |> ktab(caption = "Ingredient protein and fat fractions (g/g)", digits = 3)
```

Ingredient protein and fat fractions (g/g)

| ingredient | protein | fat |
| --- | --- | --- |
| Casein (acid) | 0.85 | 0.015 |
| Cooked whole bean powder (white kidney) | 0.25 | 0.009 |
| Corn oil | 0.00 | 1.000 |
| Anhydrous butter fat | 0.00 | 0.999 |
| DL-Methionine | 1.00 | 0.000 |
| L-Tryptophan | 1.00 | 0.000 |

```
fg <- function(pat, d) form[[d]][grepl(pat, form$ingredient)]
ip <- function(pat, col) ingr[[col]][grepl(pat, ingr$ingredient)]
diet_aa <- function(d) {
  cas <- fg("^Casein", d) * ip("^Casein", "protein"); bea <- fg("bean powder", d) * ip("bean powder", "protein")
  aa <- (cas * aa_prof$casein + bea * aa_prof$bean) / 100; names(aa) <- aa_prof$aa
  aa["Methionine"] <- aa["Methionine"] + fg("Methionine", d); aa["Tryptophan"] <- aa["Tryptophan"] + fg("Tryptophan", d)
  c(aa, protein = cas + bea + fg("Methionine", d) + fg("Tryptophan", d))
}
diet_fa <- function(d) {
  fat <- c(corn = fg("Corn oil", d) * ip("Corn oil", "fat"), butter = fg("butter fat", d) * ip("butter fat", "fat"), bean = fg("bean powder", d) * ip("bean powder", "fat"))
  setNames((fat["corn"] * fa_prof$corn + fat["butter"] * fa_prof$butter + fat["bean"] * fa_prof$bean) / 100, fa_prof$fa)
}
aa0 <- diet_aa("d0"); aa70 <- diet_aa("d70"); fa0 <- diet_fa("d0"); fa70 <- diet_fa("d70")
gkg <- function(a) 10 * c(Valine = a[["Valine"]], Leucine = a[["Leucine"]], Isoleucine = a[["Isoleucine"]],
  `Total BCAA` = a[["Valine"]] + a[["Leucine"]] + a[["Isoleucine"]], `Methionine + cysteine` = a[["Methionine"]] + a[["Cysteine"]],
  Lysine = a[["Lysine"]], Threonine = a[["Threonine"]], Tryptophan = a[["Tryptophan"]], `Phenylalanine + tyrosine` = a[["Phenylalanine"]] + a[["Tyrosine"]],
  Histidine = a[["Histidine"]], Arginine = a[["Arginine"]])
req <- setNames(nrc$nrc, nrc$aa); req <- c(req[c("Valine", "Leucine", "Isoleucine")], `Total BCAA` = sum(req[c("Valine", "Leucine", "Isoleucine")]), req[c("Methionine + cysteine", "Lysine", "Threonine", "Tryptophan", "Phenylalanine + tyrosine", "Histidine", "Arginine")])
table_s1b <- tibble(component = paste0(names(gkg(aa0)), ", g/kg diet"), diet_0 = gkg(aa0), diet_70 = gkg(aa70), ratio_70_0 = diet_70 / diet_0,
                    nrc_requirement = req, ratio_70_req = diet_70 / nrc_requirement, ratio_0_req = diet_0 / nrc_requirement) |>
  bind_rows(tibble(component = c("Protein implied by these inputs, g/100 g diet", "Linoleate 18:2n-6, g/100 g diet", "α-Linolenate 18:3n-3, g/100 g diet"),
                   diet_0 = c(aa0[["protein"]], fa0[["Linoleate 18:2n-6"]], fa0[["α-Linolenate 18:3n-3"]]),
                   diet_70 = c(aa70[["protein"]], fa70[["Linoleate 18:2n-6"]], fa70[["α-Linolenate 18:3n-3"]])) |> mutate(ratio_70_0 = diet_70 / diet_0))
table_s1b |> ktab(caption = "Table S1, part B. Ingredient-based estimates of amino-acid and fatty-acid supply (not assayed), with the NRC (1995) requirement for the mouse", digits = 3)
```

Table S1, part B. Ingredient-based estimates of amino-acid and
fatty-acid supply (not assayed), with the NRC (1995) requirement for the
mouse

| component | diet\_0 | diet\_70 | ratio\_70\_0 | nrc\_requirement | ratio\_70\_req | ratio\_0\_req |
| --- | --- | --- | --- | --- | --- | --- |
| Valine, g/kg diet | 13.183 | 9.338 | 0.708 | 5.0 | 1.868 | 2.637 |
| Leucine, g/kg diet | 18.377 | 13.661 | 0.743 | 7.0 | 1.952 | 2.625 |
| Isoleucine, g/kg diet | 9.987 | 7.475 | 0.748 | 4.0 | 1.869 | 2.497 |
| Total BCAA, g/kg diet | 41.548 | 30.474 | 0.733 | 16.0 | 1.905 | 2.597 |
| Methionine + cysteine, g/kg diet | 9.392 | 7.073 | 0.753 | 5.0 | 1.415 | 1.878 |
| Lysine, g/kg diet | 15.580 | 11.745 | 0.754 | 4.0 | 2.936 | 3.895 |
| Threonine, g/kg diet | 8.390 | 6.615 | 0.788 | 4.0 | 1.654 | 2.097 |
| Tryptophan, g/kg diet | 2.597 | 2.507 | 0.965 | 1.0 | 2.507 | 2.597 |
| Phenylalanine + tyrosine, g/kg diet | 21.173 | 14.863 | 0.702 | 7.6 | 1.956 | 2.786 |
| Histidine, g/kg diet | 5.793 | 4.606 | 0.795 | 2.0 | 2.303 | 2.896 |
| Arginine, g/kg diet | 7.191 | 8.089 | 1.125 | 3.0 | 2.696 | 2.397 |
| Protein implied by these inputs, g/100 g diet | 20.275 | 16.595 | 0.818 | NA | NA | NA |
| Linoleate 18:2n-6, g/100 g diet | 6.120 | 6.230 | 1.018 | NA | NA | NA |
| α-Linolenate 18:3n-3, g/100 g diet | 0.150 | 0.231 | 1.537 | NA | NA | NA |

```
write_xlsx(list(A_formulation = form, B_estimates = table_s1b), file.path(out_dir, "tables", "Table_S1_diet_composition.xlsx"))
s1 <- function(k) table_s1b |> filter(grepl(k, component, fixed = TRUE))
n6n3 <- c(d0 = fa0[["Linoleate 18:2n-6"]] / fa0[["α-Linolenate 18:3n-3"]], d70 = fa70[["Linoleate 18:2n-6"]] / fa70[["α-Linolenate 18:3n-3"]])
```

By estimate, the 70% diet supplied 16.6 vs. 20.3 g protein/100 g (18%
less), 27% less branched-chain amino acid, 25% less methionine plus
cysteine, 1.54-fold α-linolenate, and 1.02-fold linoleate. Both diets
exceed every NRC indispensable amino-acid requirement: the 70% diet by
1.4-fold (methionine + cysteine) to 2.9-fold (lysine), and the 0% diet
by 1.9- to 3.9-fold. The estimated linoleate:α-linolenate ratio is 41
(0%) and 27 (70%). Neither diet contains preformed EPA, DHA, or
arachidonate.

Recomputed estimates against the values stored in the workbook (parts
D2 and E).

```
wb_e <- block("Fatty acid", "0% bean") |> transmute(fa = `...1`, wb_0 = as.numeric(`...2`), wb_70 = as.numeric(`...3`)) |> filter(fa %in% names(fa0))
c(max_abs_diff_aa_gkg = max(abs(c(nrc$wb_0 - gkg(aa0)[nrc$aa], nrc$wb_70 - gkg(aa70)[nrc$aa]))),
  max_abs_diff_fa = max(abs(c(wb_e$wb_0 - fa0[wb_e$fa], wb_e$wb_70 - fa70[wb_e$fa]))))
```

```
## max_abs_diff_aa_gkg     max_abs_diff_fa 
##        3.552714e-15        8.881784e-16
```

# 3 Metabolomics data: import and inspection

Each matrix workbook (Metabolon HD4) contains a chemical annotation,
sample metadata, raw peak areas, batch-normalized data, batch-normalized
imputed data, and natural-log-transformed data; the plasma workbook also
contains a volume-normalized table.

```
read_block <- function(f, sheet) {
  d <- as.data.frame(read_excel(f, sheet))
  m <- as.matrix(d[, -1]); rownames(m) <- d[[1]]; m
}
M <- list()
for (m in names(mats)) {
  f <- file.path(data_dir, "Data_S6_Metabolon", sprintf("COSU-01-23VW+ %s DATA TABLES.XLSX", m))
  ann  <- read_excel(f, "Chemical Annotation") |> mutate(CHEM_ID = as.character(CHEM_ID))
  meta <- read_excel(f, "Sample Meta Data") |>
    mutate(POOL = as.integer(str_extract(CLIENT_SAMPLE_ID, "(?<=P)\\d+$")))
  M[[m]] <- list(file = f, sheets = excel_sheets(f), ann = ann, meta = meta,
                 peak = read_block(f, "Peak Area Data"),
                 bn   = read_block(f, "Batch-normalized Data"),
                 imp  = read_block(f, "Batch-norm Imputed Data"),
                 lg   = read_block(f, "Log Transformed Data"))
  if (m == "PLASMA") M[[m]]$vol <- read_block(f, "Vol_extracted-norm Data")
}
sapply(M, function(x) c(samples = nrow(x$imp), features = ncol(x$imp),
                        annotated = nrow(x$ann), named = sum(x$ann$TYPE == "NAMED"),
                        unnamed = sum(x$ann$TYPE == "UNNAMED")))
```

```
##           CECAL LIVER PLASMA
## samples      16    16     16
## features    962  1065   1024
## annotated   962  1065   1024
## named       788   958    919
## unnamed     174   107    105
```

```
bind_rows(lapply(names(M), function(m) M[[m]]$meta |>
  transmute(matrix = m, PARENT_SAMPLE_NAME, CLIENT_SAMPLE_ID, POOL, GENDER, GROUP_ID))) |>
  count(matrix, GENDER, GROUP_ID) |> ktab(caption = "Samples per matrix, sex, and group")
```

Samples per matrix, sex, and group

| matrix | GENDER | GROUP\_ID | n |
| --- | --- | --- | --- |
| CECAL | Female | BEAN | 1 |
| CECAL | Female | CONTROL | 1 |
| CECAL | Male | BEAN | 7 |
| CECAL | Male | CONTROL | 7 |
| LIVER | Female | BEAN | 1 |
| LIVER | Female | CONTROL | 1 |
| LIVER | Male | BEAN | 7 |
| LIVER | Male | CONTROL | 7 |
| PLASMA | Female | BEAN | 1 |
| PLASMA | Female | CONTROL | 1 |
| PLASMA | Male | BEAN | 7 |
| PLASMA | Male | CONTROL | 7 |

Each workbook holds 16 samples: 14 male pools (7 control, 7 bean) and
one female pool per diet. Only male samples are analyzed. The same pool
numbers were submitted in every matrix:

```
male_pools <- lapply(M, function(x) x$meta |> filter(GENDER == "Male") |>
                       group_by(GROUP_ID) |> summarise(pools = paste(sort(POOL), collapse = ", ")))
male_pools$CECAL |> ktab(caption = "Male pools profiled (identical across matrices)")
```

Male pools profiled (identical across matrices)

| GROUP\_ID | pools |
| --- | --- |
| BEAN | 9, 14, 19, 24, 29, 34, 49 |
| CONTROL | 6, 11, 16, 21, 26, 31, 36 |

```
stopifnot(identical(male_pools$CECAL, male_pools$LIVER), identical(male_pools$CECAL, male_pools$PLASMA))
```

## 3.1 Consistency of the processed tables

```
chk <- sapply(M, function(x) {
  mins <- apply(x$bn, 2, min, na.rm = TRUE)
  imp_is_min <- all(sapply(seq_len(ncol(x$bn)), function(j) {
    i <- is.na(x$bn[, j]); !any(i) || isTRUE(all.equal(unname(x$imp[i, j]), rep(unname(mins[j]), sum(i))))
  }))
  c(same_feature_order  = identical(colnames(x$bn), colnames(x$imp)),
    imputed_equals_observed = isTRUE(all.equal(x$imp[!is.na(x$bn)], x$bn[!is.na(x$bn)])),
    imputed_is_feature_min  = imp_is_min,
    log_sheet_is_ln_imputed = isTRUE(all.equal(unname(x$lg), unname(log(x$imp)), tolerance = 1e-8)),
    log_sheet_is_ln_volnorm = !is.null(x$vol) && isTRUE(all.equal(unname(x$lg), unname(log(x$vol)), tolerance = 1e-8)),
    na_in_peak_matches_bn   = identical(is.na(x$peak), is.na(x$bn)))
})
chk
```

```
##                         CECAL LIVER PLASMA
## same_feature_order       TRUE  TRUE   TRUE
## imputed_equals_observed  TRUE  TRUE   TRUE
## imputed_is_feature_min   TRUE  TRUE   TRUE
## log_sheet_is_ln_imputed  TRUE  TRUE  FALSE
## log_sheet_is_ln_volnorm FALSE FALSE   TRUE
## na_in_peak_matches_bn    TRUE  TRUE   TRUE
```

Missing values in the batch-normalized tables were imputed with the
per-compound minimum across the 16 samples. The log sheet is the natural
log of the imputed table for cecal content and liver and of the
volume-normalized table for plasma. All analyses below start from the
batch-normalized imputed table, restricted to the 14 male samples, and
use log_2_ values where a log scale is required.

```
for (m in names(M)) {
  meta <- M[[m]]$meta |> filter(GENDER == "Male") |>
    mutate(group = factor(ifelse(GROUP_ID == "BEAN", "Bean", "Control"), c("Control", "Bean")))
  ids <- meta$PARENT_SAMPLE_NAME
  M[[m]]$mmeta <- meta
  M[[m]]$x   <- M[[m]]$imp[ids, ]
  M[[m]]$xbn <- M[[m]]$bn[ids, ]
  M[[m]]$g   <- meta$group
}
sapply(M, function(x) table(x$g))
```

```
##         CECAL LIVER PLASMA
## Control     7     7      7
## Bean        7     7      7
```

## 3.2 Feature detection and missing values

```
miss_tab <- bind_rows(lapply(names(M), function(m) {
  nmiss <- colSums(is.na(M[[m]]$xbn)); is_named <- M[[m]]$ann$TYPE[match(colnames(M[[m]]$xbn), M[[m]]$ann$CHEM_ID)] == "NAMED"
  const <- apply(M[[m]]$x, 2, sd) == 0
  tibble(matrix = m, features = length(nmiss), named = sum(is_named), unnamed = sum(!is_named),
         any_missing = sum(nmiss > 0), missing_gt_half = sum(nmiss > 7), named_missing_gt_half = sum(nmiss > 7 & is_named),
         detected_ge1 = sum(nmiss < 14), not_detected = sum(nmiss == 14), constant_after_imputation = sum(const))
}))
miss_tab |> ktab(caption = "Detection and missingness in the 14 male samples (before imputation)")
```

Detection and missingness in the 14 male samples (before imputation)

| matrix | features | named | unnamed | any\_missing | missing\_gt\_half | named\_missing\_gt\_half | detected\_ge1 | not\_detected | constant\_after\_imputation |
| --- | --- | --- | --- | --- | --- | --- | --- | --- | --- |
| CECAL | 962 | 788 | 174 | 320 | 55 | 50 | 959 | 3 | 3 |
| LIVER | 1065 | 958 | 107 | 228 | 13 | 12 | 1065 | 0 | 0 |
| PLASMA | 1024 | 919 | 105 | 310 | 41 | 32 | 1023 | 1 | 5 |

Across the 14 male samples, 320, 228, 310 features (cecal content,
liver, plasma) contained at least one missing value before imputation,
and 55, 13, 41 (50, 12, 32 named) were missing in more than half of the
samples. 959, 1065, 1023 features were detected in at least one
sample.

Features that are constant after imputation (not detected in any male
sample, or detected only at the imputed minimum) cannot be tested and
are listed here.

```
bind_rows(lapply(names(M), function(m) {
  x <- M[[m]]$x; nm <- colSums(is.na(M[[m]]$xbn))
  j <- which(apply(x, 2, sd) == 0)
  tibble(matrix = m, CHEM_ID = colnames(x)[j], name = M[[m]]$ann$CHEMICAL_NAME[match(colnames(x)[j], M[[m]]$ann$CHEM_ID)],
         n_missing_of_14 = nm[j])
})) |> ktab()
```

| matrix | CHEM\_ID | name | n\_missing\_of\_14 |
| --- | --- | --- | --- |
| CECAL | 100001658 | taurolithocholate 3-sulfate | 14 |
| CECAL | 100001988 | 5alpha-pregnan-3beta,20alpha-diol disulfate | 14 |
| CECAL | 100020824 | lithocholic acid sulfate (2) | 14 |
| PLASMA | 926 | caproate (6:0) | 13 |
| PLASMA | 1136 | valerate (5:0) | 13 |
| PLASMA | 100010919 | oleoyl-oleoyl-glycerol (18:1/18:1) [2]\* | 13 |
| PLASMA | 100010965 | 1-oleoyl-2-eicosenoyl-GPC (18:1/20:1)\* | 14 |
| PLASMA | 100020859 | 12-ketolithocholate | 13 |

Plasma: the volume-normalized table differs from the batch-normalized
imputed table only for the sample with a reduced extraction volume.

```
pm <- M$PLASMA$meta
ratio <- M$PLASMA$vol[pm$PARENT_SAMPLE_NAME, ] / M$PLASMA$imp[pm$PARENT_SAMPLE_NAME, ]
tibble(sample = pm$PARENT_SAMPLE_NAME, pool = pm$POOL, vol_extracted_uL = pm$VOL_EXTRACTED,
       median_ratio_vol_to_imputed = apply(ratio, 1, median, na.rm = TRUE)) |>
  filter(abs(median_ratio_vol_to_imputed - 1) > 1e-6 | vol_extracted_uL != 100) |> ktab(digits = 4)
```

| sample | pool | vol\_extracted\_uL | median\_ratio\_vol\_to\_imputed |
| --- | --- | --- | --- |
| COSU-02065 | 19 | 90 | 1.1111 |

The analysis uses the batch-normalized imputed plasma table; the
volume-normalized table rescales one bean sample (pool 19) by a constant
factor.

# 4 Phenotypes and pooling crosswalk

```
nec <- read_excel(file.path(data_dir, "CSU-236-237-239_DRS_Male_and_Female_Necropsy_Tissue_Weights_5-3-26.xlsx"), "Tissue Weights")
nec |> count(STUDY, SEX, DIET) |> ktab(caption = "Necropsy records by study, sex, and diet")
```

Necropsy records by study, sex, and diet

| STUDY | SEX | DIET | n |
| --- | --- | --- | --- |
| CSU-236 | M | 0.0 Protein Bean | 20 |
| CSU-236 | M | 17.5 Protein Bean | 20 |
| CSU-236 | M | 35.0 Protein Bean | 20 |
| CSU-236 | M | 70.0 Protein Bean | 20 |
| CSU-237 | F | 0.0 Protein Bean | 20 |
| CSU-237 | F | 17.5 Protein Bean | 20 |
| CSU-237 | F | 35.0 Protein Bean | 20 |
| CSU-237 | F | 70.0 Protein Bean | 20 |
| CSU-239 | F | Low Fat | 15 |
| CSU-239 | M | Low Fat | 20 |

```
an <- nec |> filter(SEX == "M", DIET %in% c("0.0 Protein Bean", "70.0 Protein Bean")) |>
  mutate(group = factor(ifelse(DIET == "0.0 Protein Bean", "Control", "Bean"), c("Control", "Bean")),
         VIS_FAT_mg = SUM_VISCERAL_FAT_mg, SQ_FAT_mg = SUM_SQ_Fat_mg, TOTAL_DEPOT_mg = SQ_FAT_mg + VIS_FAT_mg)
stopifnot(all(an$SUM_SQ_Fat_mg == an$SQ_FAT_R_mg + an$SQ_FAT_L_mg),
          all(an$SUM_VISCERAL_FAT_mg == an$MES_FAT_mg + an$RP_FAT_mg + an$EP_PM_FAT_mg))
an |> group_by(group) |> summarise(animals = n(), pools = n_distinct(POOL), animals_per_pool = paste(range(table(POOL)), collapse = "-"),
                                   days_on_diet = paste(range(DOD_NX), collapse = "-"), weeks_of_age = paste(round(range(WOA_NX), 1), collapse = "-")) |>
  ktab(caption = "Male animals in the 0% and 70% bean groups")
```

Male animals in the 0% and 70% bean groups

| group | animals | pools | animals\_per\_pool | days\_on\_diet | weeks\_of\_age |
| --- | --- | --- | --- | --- | --- |
| Control | 20 | 10 | 2-2 | 97-101 | 22-22.6 |
| Bean | 20 | 10 | 2-2 | 97-101 | 22-22.6 |

Subcutaneous fat is the sum of the left and right inguinal depots,
total visceral fat is the sum of mesenteric, retroperitoneal, and
epididymal fat (checked above), and total depot mass is the sum of the
four depots (subcutaneous plus total visceral fat). Animals spent 97-101
days on diet.

## 4.1 Pool structure

Within each diet group, the two donors to a pool occupy adjacent
ranks of tibia-normalized subcutaneous fat:

```
pool_rank <- an |> group_by(group) |> mutate(rank_sq_norm = rank(SUM_SQ_FAT_NORM_mg_mm)) |> ungroup()
prof_pools <- lapply(split(M$CECAL$mmeta$POOL, M$CECAL$mmeta$group), sort)
pool_tab <- pool_rank |> group_by(group, POOL) |>
  summarise(donor_ranks = paste(sort(rank_sq_norm), collapse = ", "), .groups = "drop") |>
  mutate(profiled = POOL %in% unlist(prof_pools)) |> arrange(group, POOL)
stopifnot(all(sapply(strsplit(pool_tab$donor_ranks, ", "), function(r) diff(as.numeric(r)) == 1)))
pool_tab |> ktab(caption = "Pools, donor ranks of tibia-normalized subcutaneous fat, and metabolomic profiling status")
```

Pools, donor ranks of tibia-normalized subcutaneous fat, and metabolomic
profiling status

| group | POOL | donor\_ranks | profiled |
| --- | --- | --- | --- |
| Control | 1 | 1, 2 | FALSE |
| Control | 6 | 3, 4 | TRUE |
| Control | 11 | 5, 6 | TRUE |
| Control | 16 | 7, 8 | TRUE |
| Control | 21 | 9, 10 | TRUE |
| Control | 26 | 11, 12 | TRUE |
| Control | 31 | 13, 14 | TRUE |
| Control | 36 | 15, 16 | TRUE |
| Control | 41 | 17, 18 | FALSE |
| Control | 46 | 19, 20 | FALSE |
| Bean | 4 | 1, 2 | FALSE |
| Bean | 9 | 3, 4 | TRUE |
| Bean | 14 | 5, 6 | TRUE |
| Bean | 19 | 7, 8 | TRUE |
| Bean | 24 | 9, 10 | TRUE |
| Bean | 29 | 11, 12 | TRUE |
| Bean | 34 | 13, 14 | TRUE |
| Bean | 39 | 15, 16 | FALSE |
| Bean | 44 | 17, 18 | FALSE |
| Bean | 49 | 19, 20 | TRUE |

```
unprof <- pool_tab |> filter(!profiled) |> group_by(group) |> summarise(txt = paste0("pool ", POOL, " (ranks ", donor_ranks, ")", collapse = "; "))
top_rank <- pool_tab |> filter(group == "Bean", profiled) |> slice_tail(n = 1)
```

Unprofiled control pools: pool 1 (ranks 1, 2); pool 41 (ranks 17,
18); pool 46 (ranks 19, 20). Unprofiled bean pools: pool 4 (ranks 1, 2);
pool 39 (ranks 15, 16); pool 44 (ranks 17, 18). The highest-ranked bean
pool (pool 49, ranks 19, 20) was profiled.

## 4.2 Pool-level phenotypes and the adiposity response

For cecal content and liver, each sample is linked to the mean
phenotype of its two donors.

```
ph_vars <- c(Body_weight = "FINAL_BW_g", Subcutaneous_fat = "SQ_FAT_mg", Mesenteric_fat = "MES_FAT_mg",
             Retroperitoneal_fat = "RP_FAT_mg", Epididymal_fat = "EP_PM_FAT_mg", Total_visceral_fat = "VIS_FAT_mg", Total_depot_mass = "TOTAL_DEPOT_mg")
pool_ph <- an |> group_by(group, POOL) |> summarise(across(all_of(unname(ph_vars)), mean), .groups = "drop")
pool_ph14 <- pool_ph |> filter(POOL %in% unlist(prof_pools))

pct_diff <- function(d) bind_rows(lapply(names(ph_vars), function(v) {
  b <- d[[ph_vars[v]]][d$group == "Bean"]; c0 <- d[[ph_vars[v]]][d$group == "Control"]
  tt <- t.test(b, c0); mc <- mean(c0)
  tibble(phenotype = gsub("_", " ", v), n_control = length(c0), n_bean = length(b), mean_control = mc, mean_bean = mean(b),
         pct_diff = 100 * (mean(b) / mc - 1), ci_low = 100 * tt$conf.int[1] / mc, ci_high = 100 * tt$conf.int[2] / mc, p = tt$p.value)
}))
adip14 <- pct_diff(pool_ph14); adip40 <- pct_diff(an)
adip14 |> ktab(caption = "Bean vs. control, 14 profiled pools (Welch's t-test; % difference relative to the control mean)", digits = 3)
```

Bean vs. control, 14 profiled pools (Welch’s t-test; % difference
relative to the control mean)

| phenotype | n\_control | n\_bean | mean\_control | mean\_bean | pct\_diff | ci\_low | ci\_high | p |
| --- | --- | --- | --- | --- | --- | --- | --- | --- |
| Body weight | 7 | 7 | 49.607 | 43.886 | -11.533 | -17.392 | -5.675 | 0.0022 |
| Subcutaneous fat | 7 | 7 | 2331.286 | 1588.357 | -31.868 | -55.328 | -8.407 | 0.013 |
| Mesenteric fat | 7 | 7 | 1168.857 | 749.500 | -35.878 | -62.590 | -9.165 | 0.013 |
| Retroperitoneal fat | 7 | 7 | 495.929 | 529.929 | 6.856 | -9.123 | 22.834 | 0.37 |
| Epididymal fat | 7 | 7 | 1790.357 | 2167.429 | 21.061 | 3.444 | 38.678 | 0.023 |
| Total visceral fat | 7 | 7 | 3455.143 | 3446.857 | -0.240 | -14.891 | 14.411 | 0.97 |
| Total depot mass | 7 | 7 | 5786.429 | 5035.214 | -12.982 | -29.583 | 3.618 | 0.11 |

```
adip40 |> ktab(caption = "Bean vs. control, all 40 animals (Welch's t-test)", digits = 3)
```

Bean vs. control, all 40 animals (Welch’s t-test)

| phenotype | n\_control | n\_bean | mean\_control | mean\_bean | pct\_diff | ci\_low | ci\_high | p |
| --- | --- | --- | --- | --- | --- | --- | --- | --- |
| Body weight | 20 | 20 | 49.245 | 44.26 | -10.123 | -14.017 | -6.228 | 6.5e-06 |
| Subcutaneous fat | 20 | 20 | 2330.350 | 1599.05 | -31.382 | -45.173 | -17.590 | 4.7e-05 |
| Mesenteric fat | 20 | 20 | 1161.950 | 788.20 | -32.166 | -51.803 | -12.528 | 0.002 |
| Retroperitoneal fat | 20 | 20 | 490.850 | 515.60 | 5.042 | -5.742 | 15.826 | 0.35 |
| Epididymal fat | 20 | 20 | 1809.200 | 2210.10 | 22.159 | 8.748 | 35.570 | 0.0019 |
| Total visceral fat | 20 | 20 | 3462.000 | 3513.90 | 1.499 | -7.798 | 10.796 | 0.75 |
| Total depot mass | 20 | 20 | 5792.350 | 5112.95 | -11.729 | -21.734 | -1.724 | 0.023 |

In the 14 profiled pools, bean feeding was associated with lower body
weight (-11.5%), subcutaneous fat (-31.9%), and mesenteric fat (-35.9%),
higher epididymal fat (+21.1%), and no difference in retroperitoneal fat
(p = 0.368) or total visceral fat (-0.2%, p = 0.970); total depot mass
was -13.0% (p = 0.109). The same pattern held in all 40 animals (body
weight -10.1%, subcutaneous fat -31.4%, mesenteric fat -32.2%,
epididymal fat +22.2%, total visceral fat +1.5%, p = 0.745), except that
total depot mass, which did not differ significantly in the pools, was
-11.7% in all 40 animals (p = 0.023).

```
f7a <- adip14 |> mutate(phenotype = factor(phenotype, rev(phenotype)), sig = p < 0.05) |>
  ggplot(aes(pct_diff, phenotype)) +
  geom_col(aes(fill = sig), colour = "#2E8B57", width = 0.6) +
  geom_errorbarh(aes(xmin = ci_low, xmax = ci_high), height = 0.2) +
  geom_vline(xintercept = 0) +
  geom_text(aes(x = max(ci_high) + 12, label = paste("p =", fmt_p(p))), size = 3, hjust = 0) +
  scale_fill_manual(values = c(`TRUE` = "#2E8B57", `FALSE` = "white"), guide = "none") +
  scale_x_continuous(expand = expansion(mult = c(0.05, 0.25))) +
  labs(x = "Bean vs. control, % difference (95% CI); 14 pools", y = NULL, title = "A  Depot-specific adiposity response")
f7a
```

## 4.3 Pooling crosswalk and donor-specific linkage

The pooling crosswalk
(`COSU-01-23VW_perpool_matrix_R2.xlsx`, sheet
`Pool_crosswalk`) lists the two donors of every pool, the
Metabolon sample of each pool in each matrix, the tissue mass
contributed by each donor, and the plasma volume contributed by each
donor.

```
s3_file <- file.path(data_dir, "COSU-01-23VW_perpool_matrix_R2.xlsx")
cw_raw <- read_excel(s3_file, "Pool_crosswalk")
cw <- tibble(POOL = cw_raw$POOL, donor1 = cw_raw$`POOL ANIMAL DONOR 1`, donor2 = cw_raw$`POOL ANIMAL DONOR 2`,
             CECAL = cw_raw$`METABOLON  CECAL CONTENTS PARENT SAMPLE NAME`, LIVER = cw_raw$`METABOLON  LIVER  PARENT SAMPLE NAME`,
             PLASMA = cw_raw$`METABOLON  PLASMA  PARENT SAMPLE NAME`,
             cecal_mg1 = cw_raw$`METABOLON CECAL WEIGHT 1 (mg)`, cecal_mg2 = cw_raw$`METABOLON CECAL WEIGHT 2 (mg)`,
             liver_mg1 = cw_raw$`METABOLON LIVER WEIGHT 1 (mg)`, liver_mg2 = cw_raw$`METABOLON LIVER WEIGHT 2 (mg)`,
             plasma_uL1 = cw_raw$`METABOLON PLASMA 1 VOL (µL)`, plasma_uL2 = cw_raw$`METABOLON PLASMA 2 VOL (µL)`)
# Consistency with the necropsy pool assignments and the Metabolon sample metadata
nec_pairs <- an |> group_by(POOL) |> summarise(donors = paste(sort(ANIMAL), collapse = ","))
cw_pairs <- cw |> transmute(POOL, donors = mapply(function(a, b) paste(sort(c(a, b)), collapse = ","), donor1, donor2))
stopifnot(isTRUE(all.equal(as.data.frame(nec_pairs |> arrange(POOL)), as.data.frame(cw_pairs |> arrange(POOL)), check.attributes = FALSE)))
for (m in names(M)) stopifnot(all(cw[[m]][match(M[[m]]$mmeta$POOL, cw$POOL)] == M[[m]]$mmeta$PARENT_SAMPLE_NAME))
cw14 <- cw |> filter(!is.na(LIVER)) |> mutate(plasma_donors = (plasma_uL1 > 0) + (plasma_uL2 > 0),
  cecal_mg = cecal_mg1 + cecal_mg2, liver_mg = liver_mg1 + liver_mg2, plasma_uL = plasma_uL1 + plasma_uL2)
cw14 |> select(POOL, donor1, donor2, cecal_mg, liver_mg, plasma_uL1, plasma_uL2, plasma_donors) |> arrange(POOL) |>
  ktab(caption = "Profiled pools: donors, pooled tissue mass (mg), and plasma volume contributed by each donor (µL)", digits = 1)
```

Profiled pools: donors, pooled tissue mass (mg), and plasma volume
contributed by each donor (µL)

| POOL | donor1 | donor2 | cecal\_mg | liver\_mg | plasma\_uL1 | plasma\_uL2 | plasma\_donors |
| --- | --- | --- | --- | --- | --- | --- | --- |
| 6 | 8927 | 8926 | 107.9 | 108.2 | 75 | 75 | 2 |
| 9 | 8972 | 8910 | 109.6 | 109.0 | 0 | 150 | 1 |
| 11 | 8965 | 8967 | 109.6 | 101.2 | 0 | 150 | 1 |
| 14 | 8935 | 8913 | 113.3 | 110.5 | 75 | 75 | 2 |
| 16 | 8953 | 8928 | 91.6 | 114.1 | 75 | 75 | 2 |
| 19 | 8924 | 8887 | 114.7 | 123.7 | 0 | 91 | 1 |
| 21 | 8969 | 8966 | 106.7 | 117.3 | 0 | 150 | 1 |
| 24 | 8938 | 8885 | 110.0 | 110.8 | 150 | 0 | 1 |
| 26 | 8952 | 8925 | 114.2 | 107.4 | 75 | 75 | 2 |
| 29 | 8920 | 8912 | 102.0 | 109.6 | 0 | 150 | 1 |
| 31 | 8901 | 8950 | 105.2 | 104.7 | 150 | 0 | 1 |
| 34 | 8970 | 8973 | 110.2 | 107.0 | 0 | 150 | 1 |
| 36 | 8954 | 8900 | 105.8 | 114.4 | 150 | 0 | 1 |
| 49 | 8889 | 8922 | 102.6 | 105.1 | 0 | 150 | 1 |

Donor animals and Metabolon sample names in the crosswalk agree with
the necropsy pool assignments and the sample metadata. Cecal and liver
samples contained 92–115 mg and 101–124 mg of tissue from both donors.
Of the 14 plasma samples, 4 were two-donor mixtures (pools 6, 14, 16,
26) and 10 came from one donor (4 control, 6 bean).

Each sample is linked to the mean phenotype of the animals that
contributed to it: both donors for cecal content and liver, and the
contributing donor(s) for plasma.

```
sample_donors <- bind_rows(lapply(names(M), function(m) bind_rows(lapply(seq_len(nrow(cw14)), function(i) {
  r <- cw14[i, ]; d <- if (m == "PLASMA") c(r$donor1, r$donor2)[c(r$plasma_uL1, r$plasma_uL2) > 0] else c(r$donor1, r$donor2)
  tibble(matrix = m, sample = r[[m]], POOL = r$POOL, ANIMAL = d)
}))))
ph_cols <- c("FINAL_BW_g", "SQ_FAT_mg", "MES_FAT_mg", "RP_FAT_mg", "EP_PM_FAT_mg", "VIS_FAT_mg", "TOTAL_DEPOT_mg", "SUM_SQ_FAT_NORM_mg_mm")
sample_ph <- sample_donors |> left_join(an |> select(ANIMAL, group, all_of(ph_cols)), by = "ANIMAL") |>
  group_by(matrix, sample, POOL, group) |> summarise(n_donors = n(), across(all_of(ph_cols), mean), .groups = "drop")
sample_ph |> count(matrix, n_donors) |> ktab(caption = "Number of donors linked to each sample")
```

Number of donors linked to each sample

| matrix | n\_donors | n |
| --- | --- | --- |
| CECAL | 2 | 14 |
| LIVER | 2 | 14 |
| PLASMA | 1 | 10 |
| PLASMA | 2 | 4 |

Hepatic lipid was quantified gravimetrically for every animal
(`CSU-236-239 LIVER DRY MATTER and LIPID QUANTIFICATION-MALES SYSTAT.xlsx`):
a liver aliquot was weighed wet and after drying, and its extracted
lipid was weighed. Hepatic lipid is expressed as mg lipid per g dry
liver (lipid mass / dry mass × 1000), and each sample is linked to the
mean of the animals that contributed to it.

```
lip <- read_excel(file.path(data_dir, "CSU-236-239 LIVER DRY MATTER and LIPID QUANTIFICATION-MALES SYSTAT.xlsx")) |>
  rename(lipid_run = 1) |> mutate(dry_matter_pct = 100 * DRY_LIVER_g / WET_LIVER_g, lipid_mg_g_dry = 1000 * LIPID_g / DRY_LIVER_g)
stopifnot(!anyDuplicated(lip$ANIMAL), all(an$ANIMAL %in% lip$ANIMAL), isTRUE(all.equal(lip$PERC_DRY_MATTER * 100, lip$dry_matter_pct)))
an <- an |> left_join(lip |> select(ANIMAL, lipid_run, WET_LIVER_g, DRY_LIVER_g, LIPID_g, dry_matter_pct, LIVER_LIPID = lipid_mg_g_dry), by = "ANIMAL")
an |> group_by(group) |> summarise(n = n(), wet_mg = mean(1000 * WET_LIVER_g), dry_matter_pct = mean(dry_matter_pct), lipid_mg = mean(1000 * LIPID_g),
                                   lipid_mg_per_g_dry = mean(LIVER_LIPID), sd = sd(LIVER_LIPID)) |>
  ktab(caption = "Hepatic lipid quantification, all 40 animals (aliquot masses, dry matter, and lipid per g dry liver)", digits = 1)
```

Hepatic lipid quantification, all 40 animals (aliquot masses, dry
matter, and lipid per g dry liver)

| group | n | wet\_mg | dry\_matter\_pct | lipid\_mg | lipid\_mg\_per\_g\_dry | sd |
| --- | --- | --- | --- | --- | --- | --- |
| Control | 20 | 74.0 | 53.1 | 15.4 | 405.6 | 154.6 |
| Bean | 20 | 73.6 | 62.2 | 5.8 | 129.0 | 57.5 |

```
an |> count(lipid_run, group) |> pivot_wider(names_from = group, values_from = n, values_fill = 0) |> ktab(caption = "Animals per lipid extraction run")
```

Animals per lipid extraction run

| lipid\_run | Control | Bean |
| --- | --- | --- |
| 1 | 2 | 2 |
| 2 | 4 | 3 |
| 3 | 4 | 5 |
| 4 | 4 | 4 |
| 5 | 4 | 4 |
| 6 | 2 | 2 |

```
sample_ph <- sample_ph |> left_join(sample_donors |> left_join(an |> select(ANIMAL, LIVER_LIPID), by = "ANIMAL") |>
  group_by(matrix, sample) |> summarise(LIVER_LIPID = mean(LIVER_LIPID), .groups = "drop"), by = c("matrix", "sample"))
stopifnot(!anyNA(sample_ph$LIVER_LIPID))
ll <- sample_ph |> filter(matrix == "LIVER"); ll_t <- t.test(ll$LIVER_LIPID[ll$group == "Bean"], ll$LIVER_LIPID[ll$group == "Control"])
ll_pct <- 100 * (diff(rev(ll_t$estimate)) / ll_t$estimate[2])
la_t <- t.test(an$LIVER_LIPID[an$group == "Bean"], an$LIVER_LIPID[an$group == "Control"]); la_pct <- 100 * (diff(rev(la_t$estimate)) / la_t$estimate[2])
dm_t <- t.test(an$dry_matter_pct[an$group == "Bean"], an$dry_matter_pct[an$group == "Control"])
ll |> group_by(group) |> summarise(n = n(), mean = mean(LIVER_LIPID), sd = sd(LIVER_LIPID), min = min(LIVER_LIPID), max = max(LIVER_LIPID)) |>
  ktab(caption = "Hepatic lipid (mg/g dry liver) in the 14 profiled liver pools", digits = 1)
```

Hepatic lipid (mg/g dry liver) in the 14 profiled liver pools

| group | n | mean | sd | min | max |
| --- | --- | --- | --- | --- | --- |
| Control | 7 | 415.6 | 103.1 | 270.7 | 557.4 |
| Bean | 7 | 125.9 | 55.5 | 81.4 | 244.0 |

Hepatic lipid was 70% lower in the 14 profiled bean pools (126
vs. 416 mg/g dry liver; Welch p = 9.5e-05) and 68% lower in all 40
animals (p = 9.3e-08). Liver dry matter was 62.2% vs. 53.1% of wet mass
(bean vs. control; p = 1.6e-05).

The workbook also stores a precomputed lipid/dry-weight ratio,
computed from the dry mass rounded to 1 mg. It differs from lipid mass /
dry mass by up to 0.0096 (as a fraction); the check below counts the
animals for which it equals lipid mass divided by dry mass rounded to 1
mg. The stored ratio is not used. The donor-linked phenotype table of
the composite workbook (`Donor_correct_phenotype`, column
`LIVER_LIPID_mg_g_dry`) holds the values used in the models
and is compared here with the values computed above from the unrounded
masses; the difference must be zero.

```
dc <- read_excel(s3_file, "Donor_correct_phenotype") |> transmute(matrix = Tissue, sample = COSU_ID, s3_value = LIVER_LIPID_mg_g_dry)
stored <- sample_donors |> left_join(lip |> select(ANIMAL, stored_ratio = LIPID_g_DIV_DRY_WEIGHT_g), by = "ANIMAL") |>
  group_by(matrix, sample) |> summarise(stored_x1000 = 1000 * mean(stored_ratio), .groups = "drop")
c(animals = nrow(lip), stored_ratio_matches_dry_rounded_to_1mg = sum(abs(lip$LIPID_g_DIV_DRY_WEIGHT_g - lip$LIPID_g / round(lip$DRY_LIVER_g, 3)) < 1e-6),
  max_abs_diff_composite_table_vs_used_mg_g = max(abs(dc$s3_value - sample_ph$LIVER_LIPID[match(paste(dc$matrix, dc$sample), paste(sample_ph$matrix, sample_ph$sample))])),
  max_abs_diff_used_vs_stored_x1000_mg_g = max(abs(sample_ph$LIVER_LIPID - stored$stored_x1000[match(paste(sample_ph$matrix, sample_ph$sample), paste(stored$matrix, stored$sample))])))
```

```
##                                   animals 
##                              9.900000e+01 
##   stored_ratio_matches_dry_rounded_to_1mg 
##                              9.700000e+01 
## max_abs_diff_composite_table_vs_used_mg_g 
##                              5.684342e-14 
##    max_abs_diff_used_vs_stored_x1000_mg_g 
##                              3.776683e+00
```

```
stopifnot(max(abs(dc$s3_value - sample_ph$LIVER_LIPID[match(paste(dc$matrix, dc$sample), paste(sample_ph$matrix, sample_ph$sample))])) < 1e-6)
```

# 5 Diet-exposure markers and the host feature set

Diet-exposure markers are compounds with no endogenous mammalian
biosynthetic route that enter the host only from the bean ingredient or
its cooking. The classification and the rule category of each compound
are read from the per-metabolite annotation workbook
(`COSU-01-23VW_Bean_vs_Control_metabolomics_analysis_R1.xlsx`,
sheets `DA_<matrix>`, columns
`Exposure_marker_corrected`, `Exposure_rule`, and
`Borderline_retained_as_host`), matched by CHEM\_ID. Compounds
with both dietary and endogenous or microbial routes are retained as
host metabolites and flagged as borderline.

```
s1_file <- file.path(data_dir, "COSU-01-23VW_Bean_vs_Control_metabolomics_analysis_R1.xlsx")
for (m in names(M)) {
  cls <- read_excel(s1_file, paste0("DA_", m)) |> transmute(CHEM_ID = as.character(CHEM_ID), exposure = Exposure_marker_corrected,
                                                            exposure_rule = Exposure_rule, borderline = Borderline_retained_as_host)
  stopifnot(setequal(cls$CHEM_ID, M[[m]]$ann$CHEM_ID))
  M[[m]]$ann <- M[[m]]$ann |> select(-any_of(c("exposure", "exposure_rule", "borderline"))) |> left_join(cls, by = "CHEM_ID")
}
expo_tab <- bind_rows(lapply(names(M), function(m) M[[m]]$ann |> filter(exposure | borderline) |>
  transmute(matrix = m, compound = CHEMICAL_NAME, status = ifelse(exposure, "excluded", "borderline, retained"), exposure_rule))) |>
  mutate(present = TRUE) |> pivot_wider(names_from = matrix, values_from = present, values_fill = FALSE) |> arrange(status, exposure_rule, compound)
expo_tab |> ktab(caption = "Diet-exposure markers (excluded) and borderline compounds (retained), with the matrices in which each was measured")
```

Diet-exposure markers (excluded) and borderline compounds (retained),
with the matrices in which each was measured

| compound | status | exposure\_rule | CECAL | LIVER | PLASMA |
| --- | --- | --- | --- | --- | --- |
| 4-ethylcatechol sulfate | borderline, retained | NA | FALSE | FALSE | TRUE |
| 4-methylcatechol sulfate | borderline, retained | NA | TRUE | TRUE | TRUE |
| 4-vinylcatechol sulfate | borderline, retained | NA | FALSE | FALSE | TRUE |
| N-methyl-GABA | borderline, retained | NA | TRUE | TRUE | TRUE |
| caffeic acid sulfate | borderline, retained | NA | FALSE | FALSE | TRUE |
| catechol sulfate | borderline, retained | NA | FALSE | TRUE | TRUE |
| dihydrocaffeate sulfate (2) | borderline, retained | NA | FALSE | FALSE | TRUE |
| nicotinate ribonucleoside | borderline, retained | NA | TRUE | TRUE | TRUE |
| N-acetylpyrraline | excluded | Maillard product (cooking) | FALSE | FALSE | TRUE |
| pyrraline | excluded | Maillard product (cooking) | FALSE | FALSE | TRUE |
| cyclo(met-pro) | excluded | cyclic dipeptide formed on cooking | FALSE | FALSE | TRUE |
| ergothioneine | excluded | fungal/bacterial thione, no mammalian synthesis | TRUE | TRUE | TRUE |
| daidzein | excluded | isoflavone | TRUE | FALSE | FALSE |
| genistein | excluded | isoflavone | TRUE | FALSE | FALSE |
| soyasaponin I | excluded | legume saponin | TRUE | FALSE | FALSE |
| soyasaponin III | excluded | legume saponin | TRUE | FALSE | FALSE |
| S-methylcysteine | excluded | legume sulfur amino acid | TRUE | TRUE | TRUE |
| S-methylcysteine sulfoxide | excluded | legume sulfur amino acid | FALSE | FALSE | TRUE |
| pipecolate | excluded | lysine catabolite, bean-derived (>20-fold; also microbial) | TRUE | TRUE | TRUE |
| S-(3-hydroxypropyl)mercapturic acid (HPMA) | excluded | mercapturic acid of cooking-derived acrolein | TRUE | TRUE | TRUE |
| N-delta-acetylornithine | excluded | plant N-acetyl amino acid | TRUE | TRUE | TRUE |
| N2,N5-diacetylornithine | excluded | plant N-acetyl amino acid | FALSE | TRUE | TRUE |
| N-methylpipecolate | excluded | plant alkaloid | TRUE | TRUE | TRUE |
| N-methylproline | excluded | plant betaine | TRUE | FALSE | TRUE |
| homostachydrine\* | excluded | plant betaine | FALSE | FALSE | TRUE |
| stachydrine | excluded | plant betaine | TRUE | FALSE | TRUE |
| trigonelline (N’-methylnicotinate) | excluded | plant betaine (also human diet marker) | TRUE | TRUE | TRUE |
| arabinose | excluded | plant cell-wall sugar | TRUE | FALSE | TRUE |
| methyl glucopyranoside (alpha + beta) | excluded | plant glycoside | FALSE | TRUE | TRUE |
| 2,3-dihydroxy-2-methylbutyrate | excluded | plant-derived acid | FALSE | FALSE | TRUE |
| N-formylmethionine | excluded | plant/bacterial translation product of the food | TRUE | TRUE | TRUE |
| quinate | excluded | shikimate-pathway intermediate (plant) | TRUE | FALSE | FALSE |
| 3-dehydroshikimate | excluded | shikimate-pathway intermediate (plant/microbial) | TRUE | FALSE | FALSE |

The host feature set is defined as named, non-exposure features that
vary across the 14 male samples.

```
for (m in names(M)) {
  a <- M[[m]]$ann[match(colnames(M[[m]]$x), M[[m]]$ann$CHEM_ID), ]
  M[[m]]$host <- colnames(M[[m]]$x)[a$TYPE == "NAMED" & !a$exposure & apply(M[[m]]$x, 2, sd) > 0]
}
host_n <- sapply(M, function(x) length(x$host))
sapply(M, function(x) c(named = sum(x$ann$TYPE == "NAMED"), exposure_markers = sum(x$ann$exposure), host = length(x$host)))
```

```
##                  CECAL LIVER PLASMA
## named              788   958    919
## exposure_markers    17    10     19
## host               768   948    895
```

The host feature set contains 768, 948, 895 features in cecal
content, liver, and plasma.

# 6 Differential abundance

Fold change (bean/control) is the ratio of group means of the
batch-normalized imputed data. Group differences are tested by Welch’s
t-test on log_2_ values with Benjamini–Hochberg FDR within
matrix; Pareto scaling is a per-feature affine transform and leaves the
t statistic unchanged. Each comparison is repeated with the two-sided
Mann–Whitney U test: exact when the 14 values have no ties (minimum
attainable p for 7 vs. 7 = 5.8e-04) and normal approximation with tie
and continuity correction otherwise (ties arise mainly from
minimum-imputed values). Features that are constant after imputation are
not tested.

```
mw_test <- function(x, y) {
  if (anyDuplicated(c(x, y))) suppressWarnings(wilcox.test(x, y, exact = FALSE, correct = TRUE)$p.value)
  else wilcox.test(x, y, exact = TRUE)$p.value
}
run_da <- function(m, keep = rep(TRUE, nrow(M[[m]]$x))) {
  x <- M[[m]]$x[keep, ]; g <- M[[m]]$g[keep]; l2 <- log2(x); a <- M[[m]]$ann[match(colnames(x), M[[m]]$ann$CHEM_ID), ]
  nm <- is.na(M[[m]]$xbn[keep, ]); var_ok <- apply(x, 2, sd) > 0
  tt <- apply(l2, 2, function(v) if (sd(v) == 0) c(NA, NA) else { t <- t.test(v[g == "Bean"], v[g == "Control"]); c(t$statistic, t$p.value) })
  out <- tibble(matrix = m, CHEM_ID = colnames(x), name = a$CHEMICAL_NAME, super_pathway = a$SUPER_PATHWAY, sub_pathway = a$SUB_PATHWAY,
         type = a$TYPE, HMDB = a$HMDB, KEGG = a$KEGG, INCHIKEY = a$INCHIKEY, exposure = a$exposure, exposure_rule = a$exposure_rule,
         borderline = a$borderline, host = colnames(x) %in% M[[m]]$host,
         n_missing = colSums(nm), n_missing_control = colSums(nm[g == "Control", , drop = FALSE]), n_missing_bean = colSums(nm[g == "Bean", , drop = FALSE]),
         mean_control = colMeans(x[g == "Control", ]), mean_bean = colMeans(x[g == "Bean", ]),
         fc = mean_bean / mean_control, log2fc = log2(fc), welch_t = tt[1, ], p = tt[2, ],
         mw_p = ifelse(var_ok, apply(x, 2, function(v) mw_test(v[g == "Bean"], v[g == "Control"])), NA))
  out |> mutate(q = p.adjust(p, "BH"), mw_q = p.adjust(mw_p, "BH"))
}
DA <- setNames(lapply(names(M), run_da), names(M))
```

```
da_sum <- bind_rows(lapply(DA, function(d) d |> filter(!is.na(p)) |> summarise(
  matrix = first(matrix), tested = n(), p_lt_0.05 = sum(p < 0.05), pct_p_lt_0.05 = 100 * mean(p < 0.05),
  q_lt_0.05 = sum(q < 0.05), mw_q_lt_0.05 = sum(mw_q < 0.05), both = sum(q < 0.05 & mw_q < 0.05),
  welch_only = sum(q < 0.05 & mw_q >= 0.05), mw_only = sum(q >= 0.05 & mw_q < 0.05),
  up_q = sum(q < 0.05 & log2fc > 0), down_q = sum(q < 0.05 & log2fc < 0),
  median_abs_log2fc = median(abs(log2fc)), median_abs_log2fc_q = median(abs(log2fc[q < 0.05])),
  agree_all = 100 * mean((q < 0.05) == (mw_q < 0.05)), mw_sig_among_welch_sig = 100 * mean(mw_q[q < 0.05] < 0.05))))
table_s2 <- da_sum |> transmute(Matrix = mats[matrix], `Metabolites tested` = tested, `p < 0.05 (Welch)` = p_lt_0.05,
  `FDR < 0.05 (Welch)` = q_lt_0.05, `FDR < 0.05 (Mann–Whitney)` = mw_q_lt_0.05, `Both tests` = both, `Welch only` = welch_only, `MW only` = mw_only)
table_s2 |> ktab(caption = "Table S2. Differential-abundance summary by matrix (BH-FDR within matrix)")
```

Table S2. Differential-abundance summary by matrix (BH-FDR within
matrix)

| Matrix | Metabolites tested | p < 0.05 (Welch) | FDR < 0.05 (Welch) | FDR < 0.05 (Mann–Whitney) | Both tests | Welch only | MW only |
| --- | --- | --- | --- | --- | --- | --- | --- |
| Cecal content | 959 | 574 | 531 | 517 | 503 | 28 | 14 |
| Liver | 1065 | 487 | 412 | 412 | 386 | 26 | 26 |
| Plasma | 1019 | 389 | 270 | 264 | 245 | 25 | 19 |

```
write_xlsx(table_s2, file.path(out_dir, "tables", "Table_S2_differential_abundance_summary.xlsx"))
da_sum |> select(matrix, pct_p_lt_0.05, up_q, down_q, median_abs_log2fc, median_abs_log2fc_q, agree_all, mw_sig_among_welch_sig) |>
  ktab(caption = "Direction and magnitude of differences, and agreement between tests", digits = 2)
```

Direction and magnitude of differences, and agreement between tests

| matrix | pct\_p\_lt\_0.05 | up\_q | down\_q | median\_abs\_log2fc | median\_abs\_log2fc\_q | agree\_all | mw\_sig\_among\_welch\_sig |
| --- | --- | --- | --- | --- | --- | --- | --- |
| CECAL | 59.85 | 429 | 102 | 1.02 | 1.60 | 95.62 | 94.73 |
| LIVER | 45.73 | 270 | 142 | 0.47 | 0.93 | 95.12 | 93.69 |
| PLASMA | 38.17 | 148 | 122 | 0.42 | 0.95 | 95.68 | 90.74 |

Between 38% and 60% of tested features differed at p < 0.05 (cecal
content 574, liver 487, plasma 389), of which 531, 412, 270 met FDR <
0.05. The proportion of changed features and the median absolute
log_2_ fold change were largest in cecal content. The
Mann–Whitney test classified 96%, 95%, 96% of features as the Welch test
did at FDR < 0.05, and recovered 94.7%, 93.7%, 90.7% of
Welch-significant features.

```
write_xlsx(lapply(DA, as.data.frame) |> setNames(paste0("DA_", names(DA))) |>
             c(list(exposure_markers = as.data.frame(expo_tab))),
           file.path(out_dir, "tables", "Data_S1_differential_abundance.xlsx"))
```

## 6.1 Plasma: single-donor samples

Every plasma comparison is repeated on the 10 single-donor plasma
samples alone.

```
single <- M$PLASMA$mmeta$POOL %in% cw14$POOL[cw14$plasma_donors == 1]
da_single <- run_da("PLASMA", single)
sens <- DA$PLASMA |> select(CHEM_ID, name, log2fc, p, q) |> inner_join(da_single |> select(CHEM_ID, log2fc_single = log2fc, p_single = p), by = "CHEM_ID")
sig <- sens |> filter(q < 0.05)
sens_rho <- cor(sens$log2fc, sens$log2fc_single, method = "spearman", use = "complete.obs")
tibble(single_donor_control = sum(single & M$PLASMA$g == "Control"), single_donor_bean = sum(single & M$PLASMA$g == "Bean"),
       fdr_sig_full = nrow(sig), same_direction = sum(sign(sig$log2fc) == sign(sig$log2fc_single), na.rm = TRUE),
       p_lt_0.05_single = sum(sig$p_single < 0.05, na.rm = TRUE), spearman_rho_all = sens_rho) |> ktab(caption = "Plasma: full analysis vs. single-donor samples")
```

Plasma: full analysis vs. single-donor samples

| single\_donor\_control | single\_donor\_bean | fdr\_sig\_full | same\_direction | p\_lt\_0.05\_single | spearman\_rho\_all |
| --- | --- | --- | --- | --- | --- |
| 4 | 6 | 2.7e+02 | 270 | 2.4e+02 | 0.956 |

```
sens |> filter(name %in% c("valine", "leucine", "isoleucine")) |> ktab(caption = "Plasma BCAA: full analysis vs. single-donor samples")
```

Plasma BCAA: full analysis vs. single-donor samples

| CHEM\_ID | name | log2fc | p | q | log2fc\_single | p\_single |
| --- | --- | --- | --- | --- | --- | --- |
| 376 | isoleucine | -0.217 | 0.085 | 0.19 | -0.167 | 0.35 |
| 397 | leucine | -0.320 | 0.033 | 0.096 | -0.250 | 0.27 |
| 566 | valine | -0.410 | 0.03 | 0.091 | -0.332 | 0.21 |

```
write_xlsx(sens, file.path(out_dir, "tables", "Data_S1_plasma_single_donor_sensitivity.xlsx"))
```

All 270 plasma features at FDR < 0.05 in the full analysis kept
their direction in the single-donor samples (270 of 270), 244 (90%)
remained at p < 0.05, and fold changes across all features correlated
at Spearman ρ = 0.96. For valine, p = 0.21 in the single-donor
samples.

## 6.2 Statistical power

The nominal p-value that corresponds to FDR < 0.05 differs by
matrix; power is evaluated at p < 0.05 and at the median, across
matrices, of the largest nominal p-value that met FDR < 0.05.

```
fdr_alpha <- sapply(DA, function(d) max(d$p[d$q < 0.05], na.rm = TRUE))
pw_alpha <- c(`p < 0.05` = 0.05, `FDR-equivalent p` = median(fdr_alpha))
pw <- sapply(pw_alpha, function(a) power.t.test(n = 7, sig.level = a, power = 0.8)$delta)
rbind(alpha = signif(pw_alpha, 2), d_80pct_power = round(pw, 2))
```

```
##               p < 0.05 FDR-equivalent p
## alpha             0.05            0.019
## d_80pct_power     1.63            1.930
```

```
signif(fdr_alpha, 2)
```

```
##  CECAL  LIVER PLASMA 
##  0.028  0.019  0.013
```

With 7 samples per group, a two-sample t-test has 80% power for a
standardized difference of d = 1.63 at p < 0.05 and d = 1.93 at the
FDR-equivalent nominal threshold (p = 0.019).

# 7 Principal component analysis

PCA uses all detected, non-constant features (exposure markers and
unnamed features included), log_2_-transformed and
Pareto-scaled.

```
pareto <- function(X) sweep(scale(X, center = TRUE, scale = FALSE), 2, sqrt(apply(X, 2, sd)), "/")
PCA <- lapply(names(M), function(m) {
  X <- log2(M[[m]]$x); X <- X[, apply(X, 2, sd) > 0]
  pc <- prcomp(pareto(X), center = FALSE)
  list(scores = tibble(sample = rownames(X), group = M[[m]]$g, PC1 = pc$x[, 1], PC2 = pc$x[, 2]),
       ve = 100 * pc$sdev^2 / sum(pc$sdev^2), nfeat = ncol(X))
}) |> setNames(names(M))
sapply(PCA, function(p) c(features = p$nfeat, PC1 = round(p$ve[1], 1), PC2 = round(p$ve[2], 1)))
```

```
##          CECAL  LIVER PLASMA
## features 959.0 1065.0 1019.0
## PC1       50.9   40.8   33.9
## PC2       10.7   22.4   19.7
```

```
f1 <- wrap_plots(lapply(names(PCA), function(m) {
  p <- PCA[[m]]
  ggplot(p$scores, aes(PC1, PC2, colour = group, shape = group)) +
    geom_hline(yintercept = 0, colour = "grey85") + geom_vline(xintercept = 0, colour = "grey85") +
    geom_point(size = 2.4) + scale_colour_manual(values = grp_col, name = NULL) + scale_shape_manual(values = grp_shp, name = NULL) +
    labs(title = mats[m], x = sprintf("PC1 (%.0f%%)", p$ve[1]), y = sprintf("PC2 (%.0f%%)", p$ve[2]))
}), nrow = 1, guides = "collect") & theme(legend.position = "top", plot.title = element_text(hjust = 0.5))
f1
```

```
save_fig(f1, "Figure_1_PCA", 9, 3.2)
```

The diet groups separate along PC1 in every matrix (PC1 explains 51%,
41%, 34% of variance in cecal content, liver, and plasma).

Group means on PC1 and the minimum gap between groups along PC1.

```
sapply(PCA, function(p) with(p$scores, c(mean_control = mean(PC1[group == "Control"]), mean_bean = mean(PC1[group == "Bean"]),
  gap = min(PC1[group == "Bean"]) - max(PC1[group == "Control"])))) |> round(1)
```

```
##              CECAL LIVER PLASMA
## mean_control -21.3 -15.1  -14.1
## mean_bean     21.3  15.1   14.1
## gap           37.4  14.6   19.4
```

# 8 PLS-DA on the host feature set

A two-component PLS-DA (NIPALS, single 0/1 response) is fit to
log_2_ abundances of host features. Leave-one-out
cross-validation refits centering and Pareto scaling within every fold;
Q² = 1 − PRESS/SS_tot_. Significance of Q² is assessed against
1000 label permutations with the same procedure; permutation p = (number
of permuted Q² ≥ observed + 1)/1001. VIP is taken from the model fit to
all 14 samples.

```
pls1 <- function(X, y, A = 2) {
  W <- P <- matrix(0, ncol(X), A); Tm <- matrix(0, nrow(X), A); q <- numeric(A); E <- X; f <- y
  for (a in seq_len(A)) {
    w <- crossprod(E, f); w <- w / sqrt(sum(w^2)); t <- E %*% w; tt <- sum(t^2)
    p <- crossprod(E, t) / tt; q[a] <- sum(f * t) / tt
    E <- E - t %*% t(p); f <- f - q[a] * t; W[, a] <- w; P[, a] <- p; Tm[, a] <- t
  }
  list(B = W %*% solve(t(P) %*% W) %*% q, W = W, Tm = Tm, q = q)
}
pls_prep <- function(X) { mu <- colMeans(X); s <- sqrt(apply(X, 2, sd)); s[s == 0] <- 1; list(mu = mu, s = s) }
pls_fit_predict <- function(Xtr, ytr, Xte, A = 2) {
  pp <- pls_prep(Xtr); Z <- sweep(sweep(Xtr, 2, pp$mu), 2, pp$s, "/"); ym <- mean(ytr)
  fit <- pls1(Z, ytr - ym, A)
  as.vector(sweep(sweep(Xte, 2, pp$mu), 2, pp$s, "/") %*% fit$B) + ym
}
loo_q2 <- function(X, y) {
  yh <- sapply(seq_along(y), function(i) pls_fit_predict(X[-i, , drop = FALSE], y[-i], X[i, , drop = FALSE]))
  c(Q2 = 1 - sum((y - yh)^2) / sum((y - mean(y))^2), accuracy = mean((yh > 0.5) == (y == 1)))
}
vip <- function(X, y, A = 2) {
  pp <- pls_prep(X); Z <- sweep(sweep(X, 2, pp$mu), 2, pp$s, "/"); fit <- pls1(Z, y - mean(y), A)
  ss <- fit$q^2 * colSums(fit$Tm^2); Wn <- sweep(fit$W, 2, sqrt(colSums(fit$W^2)), "/")
  list(vip = sqrt(ncol(X) * (Wn^2 %*% ss) / sum(ss))[, 1], R2Y = sum(ss) / sum((y - mean(y))^2))
}
```

```
set.seed(seed)
PLS <- lapply(names(M), function(m) {
  X <- log2(M[[m]]$x[, M[[m]]$host]); y <- as.numeric(M[[m]]$g == "Bean")
  obs <- loo_q2(X, y)
  perm <- replicate(1000, loo_q2(X, sample(y))["Q2"])
  v <- vip(X, y)
  list(obs = obs, perm = perm, vip = v$vip, R2Y = v$R2Y, p = (sum(perm >= obs["Q2"]) + 1) / 1001)
}) |> setNames(names(M))
```

```
pls_tab <- bind_rows(lapply(names(PLS), function(m) tibble(matrix = m, host_features = length(M[[m]]$host),
  R2Y = PLS[[m]]$R2Y, Q2_LOO = PLS[[m]]$obs["Q2"], LOO_accuracy = PLS[[m]]$obs["accuracy"],
  perm_Q2_mean = mean(PLS[[m]]$perm), perm_Q2_max = max(PLS[[m]]$perm), n_perm_ge_obs = sum(PLS[[m]]$perm >= PLS[[m]]$obs["Q2"]),
  perm_p = PLS[[m]]$p, VIP_gt_1 = sum(PLS[[m]]$vip > 1))))
pls_tab |> ktab(caption = "Two-component PLS-DA, host features, leave-one-out cross-validation and 1000 label permutations")
```

Two-component PLS-DA, host features, leave-one-out cross-validation and
1000 label permutations

| matrix | host\_features | R2Y | Q2\_LOO | LOO\_accuracy | perm\_Q2\_mean | perm\_Q2\_max | n\_perm\_ge\_obs | perm\_p | VIP\_gt\_1 |
| --- | --- | --- | --- | --- | --- | --- | --- | --- | --- |
| CECAL | 768 | 0.998 | 0.988 | 1 | -0.574 | 0.988 | 1 | 0.002 | 284 |
| LIVER | 948 | 0.995 | 0.939 | 1 | -0.615 | 0.611 | 0 | 0.001 | 319 |
| PLASMA | 895 | 0.986 | 0.914 | 1 | -0.635 | 0.503 | 0 | 0.001 | 279 |

Leave-one-out Q² was 0.99, 0.94, 0.91 (cecal content, liver, plasma)
with 100%, 100%, 100% classification accuracy; mean permuted Q² was
-0.57, -0.61, -0.64, and 1, 0, 0 of 1000 permuted values reached the
observed Q² (permutation p = 0.002, 0.001, 0.001).

```
bind_rows(lapply(names(PLS), function(m) tibble(matrix = m, CHEM_ID = M[[m]]$host, VIP = PLS[[m]]$vip) |>
  left_join(DA[[m]] |> select(CHEM_ID, name, log2fc, q), by = "CHEM_ID") |> slice_max(VIP, n = 10))) |>
  ktab(caption = "Top 10 host features by VIP in each matrix")
```

Top 10 host features by VIP in each matrix

| matrix | CHEM\_ID | VIP | name | log2fc | q |
| --- | --- | --- | --- | --- | --- |
| CECAL | 100006726 | 3.023 | linoleoyl ethanolamide | 8.769 | 7.7e-08 |
| CECAL | 1248 | 2.531 | thymidine 5’-monophosphate | 6.162 | 6.2e-08 |
| CECAL | 1137 | 2.521 | oleoyl ethanolamide | 5.758 | 6.3e-05 |
| CECAL | 869 | 2.465 | uridine 5’-monophosphate (UMP) | 5.907 | 2.1e-07 |
| CECAL | 361 | 2.458 | inosine | 5.498 | 0.00012 |
| CECAL | 1489 | 2.422 | palmitoyl ethanolamide | 5.476 | 1.4e-05 |
| CECAL | 298 | 2.386 | 2’-deoxycytidine 5’-monophosphate | 5.395 | 9.1e-06 |
| CECAL | 100021236 | 2.380 | dehydrophytosphingosine\* | 5.554 | 7.5e-06 |
| CECAL | 1099 | 2.324 | guanosine | 5.056 | 0.00084 |
| CECAL | 100001026 | 2.319 | galactonate | 5.120 | 2e-06 |
| LIVER | 100001502 | 3.105 | gamma-glutamyl-2-aminobutyrate | 4.902 | 0.00081 |
| LIVER | 100004552 | 3.030 | 1-eicosapentaenoyl-GPE (20:5)\* | 4.111 | 4e-05 |
| LIVER | 100001843 | 2.964 | gamma-glutamylalanine | 4.044 | 0.00012 |
| LIVER | 100001314 | 2.843 | gamma-glutamylthreonine | 3.799 | 0.00022 |
| LIVER | 100001294 | 2.738 | gamma-glutamylglycine | 3.715 | 0.00081 |
| LIVER | 100009142 | 2.690 | 1-stearoyl-2-docosapentaenoyl-GPC (18:0/22:5n6)\* | -3.126 | 0.00024 |
| LIVER | 100001311 | 2.618 | ophthalmate | 3.451 | 0.00058 |
| LIVER | 100004564 | 2.599 | 2-eicosapentaenoyl-GPE (20:5)\* | 3.024 | 3.6e-05 |
| LIVER | 100015832 | 2.573 | behenoylcarnitine (C22)\* | 3.142 | 0.00032 |
| LIVER | 100015762 | 2.567 | docosatrienoate (22:3n6)\* | -2.893 | 0.00013 |
| PLASMA | 100009142 | 3.096 | 1-stearoyl-2-docosapentaenoyl-GPC (18:0/22:5n6)\* | -3.587 | 7e-05 |
| PLASMA | 100001229 | 2.806 | stearidonate (18:4n3) | 3.050 | 1.3e-05 |
| PLASMA | 100002183 | 2.798 | S-methylmethionine | 3.718 | 0.0037 |
| PLASMA | 100006098 | 2.774 | 3-hydroxypyridine sulfate | 3.333 | 0.00053 |
| PLASMA | 100000096 | 2.763 | 4-guanidinobutanoate | -4.218 | 0.01 |
| PLASMA | 100000963 | 2.756 | homocitrulline | 2.911 | 0.00019 |
| PLASMA | 100003365 | 2.731 | hydroxymethylpyrimidine | 2.847 | 2.8e-05 |
| PLASMA | 100001311 | 2.720 | ophthalmate | 3.448 | 0.0021 |
| PLASMA | 100005717 | 2.614 | 1-palmitoyl-GPG (16:0)\* | -2.579 | 1.7e-07 |
| PLASMA | 100009227 | 2.597 | 1-linoleoyl-GPG (18:2)\* | -2.547 | 3.5e-05 |

# 9 Sphingolipids

```
cer_sub <- c("Ceramides", "Dihydroceramides", "Hexosylceramides (HCER)", "Lactosylceramides (LCER)")
cer <- bind_rows(DA) |> filter(sub_pathway %in% cer_sub, !is.na(p))
cer_keep <- cer |> count(name) |> filter(n >= 2) |> pull(name)
cer_ord <- cer |> filter(name %in% cer_keep) |> distinct(name, sub_pathway) |>
  mutate(sub_pathway = factor(sub_pathway, cer_sub)) |> arrange(sub_pathway, name) |> pull(name)
cer <- cer |> filter(name %in% cer_keep)
cer |> select(matrix, name, log2fc, q) |>
  pivot_wider(names_from = matrix, values_from = c(log2fc, q)) |> ktab(caption = sprintf("Ceramide-class species detected in at least two matrices (%d species)", length(cer_keep)))
```

Ceramide-class species detected in at least two matrices (13 species)

| name | log2fc\_CECAL | log2fc\_LIVER | log2fc\_PLASMA | q\_CECAL | q\_LIVER | q\_PLASMA |
| --- | --- | --- | --- | --- | --- | --- |
| N-palmitoyl-sphingosine (d18:1/16:0) | 0.633 | -0.878 | -0.952 | 0.035 | 0.0021 | 0.0043 |
| N-stearoyl-sphingosine (d18:1/18:0)\* | -0.993 | -1.691 | -2.044 | 0.00061 | 0.00048 | 0.00099 |
| N-palmitoyl-sphinganine (d18:0/16:0) | 0.572 | -0.513 | -0.270 | 0.036 | 0.16 | 0.54 |
| lactosyl-N-palmitoyl-sphingosine (d18:1/16:0) | -0.831 | NA | -0.599 | 0.027 | NA | 0.035 |
| glycosyl-N-palmitoyl-sphingosine (d18:1/16:0) | 0.997 | -0.702 | -0.954 | 0.033 | 0.0062 | 0.0018 |
| ceramide (d18:1/14:0, d16:1/16:0)\* | -0.228 | -1.059 | -0.961 | 0.42 | 0.0038 | 0.14 |
| ceramide (d18:1/17:0, d17:1/18:0)\* | 0.152 | -0.561 | NA | 0.46 | 0.19 | NA |
| ceramide (d18:2/24:1, d18:1/24:2)\* | -0.273 | 0.190 | -0.890 | 0.57 | 0.65 | 0.001 |
| ceramide (d18:1/20:0, d16:1/22:0, d20:1/18:0)\* | -0.763 | NA | -0.971 | 0.0019 | NA | 0.05 |
| N-palmitoyl-phytosphingosine (t18:0/16:0) | 1.308 | NA | -0.003 | 0.0038 | NA | 0.89 |
| glycosyl-N-stearoyl-sphingosine (d18:1/18:0) | NA | -1.800 | -1.622 | NA | 0.0029 | 0.0012 |
| glycosyl ceramide (d16:1/24:1, d18:1/22:1)\* | NA | -1.617 | -0.865 | NA | 4.5e-05 | 0.031 |
| glycosyl ceramide (d18:2/24:1, d18:1/24:2)\* | NA | -0.272 | -0.539 | NA | 0.79 | 0.01 |

```
bar_panel <- function(d, m, ord, xlab = expression(log[2]~FC~"(bean/control)"), show_y = TRUE, hlines = NULL) {
  d <- d |> filter(matrix == m) |> mutate(name = factor(name, rev(ord)), sig = q < 0.05)
  p <- ggplot(d, aes(log2fc, name)) +
    geom_col(aes(fill = sig), colour = mat_col[m], width = 0.7) +
    geom_vline(xintercept = 0) +
    scale_fill_manual(values = c(`TRUE` = unname(mat_col[m]), `FALSE` = "white"), guide = "none") +
    scale_y_discrete(drop = FALSE) + labs(title = mats[m], x = xlab, y = NULL)
  if (!is.null(hlines)) p <- p + geom_hline(yintercept = hlines, colour = "grey80")
  if (!show_y) p <- p + theme(axis.text.y = element_blank())
  p
}
f6 <- bar_panel(cer, "CECAL", cer_ord) + bar_panel(cer, "LIVER", cer_ord, show_y = FALSE) + bar_panel(cer, "PLASMA", cer_ord, show_y = FALSE) +
  plot_annotation(caption = "Filled bars, FDR < 0.05; open bars, not significant")
f6
```

```
save_fig(f6, "Figure_6_ceramides", 10, 4.5)
```

```
sph <- bind_rows(DA) |> filter(grepl("sphingosine \\(d18:1/1[68]:0\\)|^sphingosine$|hexadecasphingosine|glycosyl-N-(palmitoyl|stearoyl)|lactosyl-N-palmitoyl|N-palmitoyl-sphinganine", name)) |>
  select(matrix, name, log2fc, q) |> arrange(name, matrix)
sph |> ktab(caption = "Named sphingolipid species referred to in the text")
```

Named sphingolipid species referred to in the text

| matrix | name | log2fc | q |
| --- | --- | --- | --- |
| CECAL | N-palmitoyl-sphinganine (d18:0/16:0) | 0.572 | 0.036 |
| LIVER | N-palmitoyl-sphinganine (d18:0/16:0) | -0.513 | 0.16 |
| PLASMA | N-palmitoyl-sphinganine (d18:0/16:0) | -0.270 | 0.54 |
| CECAL | N-palmitoyl-sphingosine (d18:1/16:0) | 0.633 | 0.035 |
| LIVER | N-palmitoyl-sphingosine (d18:1/16:0) | -0.878 | 0.0021 |
| PLASMA | N-palmitoyl-sphingosine (d18:1/16:0) | -0.952 | 0.0043 |
| CECAL | N-stearoyl-sphingosine (d18:1/18:0)\* | -0.993 | 0.00061 |
| LIVER | N-stearoyl-sphingosine (d18:1/18:0)\* | -1.691 | 0.00048 |
| PLASMA | N-stearoyl-sphingosine (d18:1/18:0)\* | -2.044 | 0.00099 |
| CECAL | glycosyl-N-palmitoyl-sphingosine (d18:1/16:0) | 0.997 | 0.033 |
| LIVER | glycosyl-N-palmitoyl-sphingosine (d18:1/16:0) | -0.702 | 0.0062 |
| PLASMA | glycosyl-N-palmitoyl-sphingosine (d18:1/16:0) | -0.954 | 0.0018 |
| LIVER | glycosyl-N-stearoyl-sphingosine (d18:1/18:0) | -1.800 | 0.0029 |
| PLASMA | glycosyl-N-stearoyl-sphingosine (d18:1/18:0) | -1.622 | 0.0012 |
| CECAL | hexadecasphingosine (d16:1)\* | -3.024 | 3.6e-05 |
| LIVER | hexadecasphingosine (d16:1)\* | -1.479 | 0.0057 |
| CECAL | lactosyl-N-palmitoyl-sphingosine (d18:1/16:0) | -0.831 | 0.027 |
| PLASMA | lactosyl-N-palmitoyl-sphingosine (d18:1/16:0) | -0.599 | 0.035 |
| CECAL | sphingosine | -1.822 | 7.7e-05 |
| LIVER | sphingosine | -0.808 | 0.00044 |
| PLASMA | sphingosine | -0.007 | 0.9 |

```
cer_counts <- cer |> group_by(matrix) |>
  summarise(species = n(), lower_q = sum(q < 0.05 & log2fc < 0), higher_q = sum(q < 0.05 & log2fc > 0), ns = sum(q >= 0.05))
cer_counts |> ktab(caption = "Ceramide-class species by direction at FDR < 0.05")
```

Ceramide-class species by direction at FDR < 0.05

| matrix | species | lower\_q | higher\_q | ns |
| --- | --- | --- | --- | --- |
| CECAL | 10 | 3 | 4 | 3 |
| LIVER | 10 | 6 | 0 | 4 |
| PLASMA | 12 | 8 | 0 | 4 |

```
sq_ <- function(m, n) sph |> filter(matrix == m, name == n)
cer_l241 <- cer |> filter(matrix == "LIVER", name == "ceramide (d18:2/24:1, d18:1/24:2)*")
```

In liver and plasma, the C16 and C18 ceramides and their glucosyl
conjugates were lower with bean feeding at q < 0.05; in liver,
ceramide (d18:2/24:1) did not differ (q = 0.65). In cecal content, C18
ceramide and lactosyl-C16-ceramide were lower, whereas C16 ceramide, C16
dihydroceramide, and glucosyl-C16-ceramide were higher.
N-stearoyl-sphingosine (d18:1/18:0) was lower in all three matrices.
Free sphingosine (q = 7.7e-05 and 4.4e-04) and hexadecasphingosine (q =
3.6e-05 and 0.006) were lower in cecal content and liver.

# 10 Branched-chain amino acids and catabolites

```
bcaa_set <- tribble(~name, ~class,
  "valine", "Parent", "leucine", "Parent", "isoleucine", "Parent",
  "3-methyl-2-oxobutyrate", "Keto acid", "3-methyl-2-oxovalerate", "Keto acid", "4-methyl-2-oxopentanoate", "Keto acid",
  "alpha-hydroxyisovalerate", "Hydroxy acid", "2-hydroxy-3-methylvalerate", "Hydroxy acid", "alpha-hydroxyisocaproate", "Hydroxy acid",
  "3-hydroxyisobutyrate", "Downstream", "beta-hydroxyisovalerate", "Downstream", "beta-hydroxyisovaleroylcarnitine", "Downstream",
  "isobutyrylcarnitine (C4)", "Downstream")
bc <- bind_rows(DA) |> filter(name %in% bcaa_set$name) |> left_join(bcaa_set, by = "name") |>
  mutate(fold = 2^log2fc)
bc |> select(matrix, class, name, log2fc, fold, q, n_missing_control, n_missing_bean) |> arrange(match(name, bcaa_set$name), matrix) |>
  ktab(caption = "BCAA, keto acids, hydroxy acids, and downstream catabolites")
```

BCAA, keto acids, hydroxy acids, and downstream catabolites

| matrix | class | name | log2fc | fold | q | n\_missing\_control | n\_missing\_bean |
| --- | --- | --- | --- | --- | --- | --- | --- |
| CECAL | Parent | valine | 0.929 | 1.904 | 0.0045 | 0 | 0 |
| LIVER | Parent | valine | -0.034 | 0.977 | 0.76 | 0 | 0 |
| PLASMA | Parent | valine | -0.410 | 0.753 | 0.091 | 0 | 0 |
| CECAL | Parent | leucine | 0.824 | 1.771 | 0.0058 | 0 | 0 |
| LIVER | Parent | leucine | 0.389 | 1.310 | 0.28 | 0 | 0 |
| PLASMA | Parent | leucine | -0.320 | 0.801 | 0.096 | 0 | 0 |
| CECAL | Parent | isoleucine | 0.663 | 1.584 | 0.0051 | 0 | 0 |
| LIVER | Parent | isoleucine | 0.144 | 1.105 | 0.64 | 0 | 0 |
| PLASMA | Parent | isoleucine | -0.217 | 0.860 | 0.19 | 0 | 0 |
| CECAL | Keto acid | 3-methyl-2-oxobutyrate | 2.500 | 5.657 | 0.0011 | 0 | 0 |
| PLASMA | Keto acid | 3-methyl-2-oxobutyrate | 0.058 | 1.041 | 0.94 | 0 | 0 |
| CECAL | Keto acid | 3-methyl-2-oxovalerate | 2.380 | 5.205 | 0.0027 | 0 | 0 |
| PLASMA | Keto acid | 3-methyl-2-oxovalerate | 0.205 | 1.153 | 0.63 | 0 | 0 |
| CECAL | Keto acid | 4-methyl-2-oxopentanoate | 2.333 | 5.038 | 0.002 | 0 | 0 |
| LIVER | Keto acid | 4-methyl-2-oxopentanoate | 0.611 | 1.528 | 0.81 | 1 | 3 |
| PLASMA | Keto acid | 4-methyl-2-oxopentanoate | 0.211 | 1.158 | 0.6 | 0 | 0 |
| CECAL | Hydroxy acid | alpha-hydroxyisovalerate | 2.035 | 4.099 | 4.6e-05 | 0 | 0 |
| LIVER | Hydroxy acid | alpha-hydroxyisovalerate | 0.604 | 1.520 | 0.004 | 0 | 0 |
| PLASMA | Hydroxy acid | alpha-hydroxyisovalerate | 0.279 | 1.213 | 0.16 | 0 | 0 |
| CECAL | Hydroxy acid | 2-hydroxy-3-methylvalerate | 1.899 | 3.731 | 0.00026 | 0 | 0 |
| LIVER | Hydroxy acid | 2-hydroxy-3-methylvalerate | 0.802 | 1.743 | 0.0036 | 0 | 0 |
| PLASMA | Hydroxy acid | 2-hydroxy-3-methylvalerate | 0.392 | 1.313 | 0.1 | 0 | 0 |
| CECAL | Hydroxy acid | alpha-hydroxyisocaproate | 2.951 | 7.733 | 2.6e-05 | 0 | 0 |
| LIVER | Hydroxy acid | alpha-hydroxyisocaproate | 1.705 | 3.260 | 0.0076 | 5 | 1 |
| PLASMA | Hydroxy acid | alpha-hydroxyisocaproate | 1.372 | 2.588 | 0.0062 | 0 | 0 |
| CECAL | Downstream | 3-hydroxyisobutyrate | 0.246 | 1.186 | 0.84 | 2 | 4 |
| LIVER | Downstream | 3-hydroxyisobutyrate | 0.893 | 1.857 | 0.0002 | 0 | 0 |
| PLASMA | Downstream | 3-hydroxyisobutyrate | 0.006 | 1.004 | 0.99 | 0 | 0 |
| CECAL | Downstream | beta-hydroxyisovalerate | -0.471 | 0.721 | 0.029 | 0 | 4 |
| LIVER | Downstream | beta-hydroxyisovalerate | 0.492 | 1.406 | 0.42 | 0 | 1 |
| CECAL | Downstream | beta-hydroxyisovaleroylcarnitine | 4.298 | 19.665 | 7.8e-07 | 6 | 0 |
| LIVER | Downstream | beta-hydroxyisovaleroylcarnitine | -0.240 | 0.847 | 0.25 | 0 | 0 |
| PLASMA | Downstream | beta-hydroxyisovaleroylcarnitine | -0.575 | 0.671 | 0.085 | 0 | 0 |
| LIVER | Downstream | isobutyrylcarnitine (C4) | 1.064 | 2.091 | 0.013 | 0 | 0 |
| PLASMA | Downstream | isobutyrylcarnitine (C4) | 0.973 | 1.963 | 0.19 | 3 | 2 |

```
f4 <- bar_panel(bc, "CECAL", bcaa_set$name, hlines = c(4.5, 7.5, 10.5)) + bar_panel(bc, "LIVER", bcaa_set$name, show_y = FALSE, hlines = c(4.5, 7.5, 10.5)) +
  bar_panel(bc, "PLASMA", bcaa_set$name, show_y = FALSE, hlines = c(4.5, 7.5, 10.5)) +
  plot_annotation(caption = "Filled bars, FDR < 0.05; open bars, not significant; grey lines separate parents, keto acids, hydroxy acids, and downstream catabolites")
f4
```

```
save_fig(f4, "Figure_4_BCAA", 10, 4.5)
```

```
rng <- function(cl, m = "CECAL") bc |> filter(matrix == m, class == cl) |> summarise(lo = min(fold), hi = max(fold), qmax = max(q))
cec_par <- rng("Parent"); cec_keto <- rng("Keto acid"); cec_hyd <- rng("Hydroxy acid")
liv_hyd <- bc |> filter(matrix == "LIVER", class == "Hydroxy acid")
pla_par <- bc |> filter(matrix == "PLASMA", class == "Parent")
```

In cecal content, the parent BCAA were 1.6–1.9-fold higher with bean
feeding, the keto acids 5.0–5.7-fold, and the hydroxy acids 3.7–7.7-fold
(q ≤ 0.006). In liver, the hydroxy acids were higher
(alpha-hydroxyisocaproate q = 0.008; alpha-hydroxyisovalerate q = 0.004;
2-hydroxy-3-methylvalerate q = 0.004); α-hydroxyisocaproate was below
detection in 5 of 7 control liver pools. In plasma, valine and leucine
were modestly lower (log_2_FC = -0.41 and -0.32; q = 0.09 and
0.10).

# 11 Polyunsaturated fatty acids

```
FA <- c(LA = "linoleate (18:2n6)", ALA = "linolenate [alpha or gamma; (18:3n3 or 6)]", SDA = "stearidonate (18:4n3)",
        DGLA = "dihomo-linolenate (20:3n3 or n6)", EDA = "dihomo-linoleate (20:2n6)", ARA = "arachidonate (20:4n6)", EPA = "eicosapentaenoate (EPA; 20:5n3)",
        ADR = "adrenate (22:4n6)", n6DPA = "docosapentaenoate (n6 DPA; 22:5n6)", n3DPA = "docosapentaenoate (n3 DPA; 22:5n3)",
        DHA = "docosahexaenoate (DHA; 22:6n3)", MEAD = "mead acid (20:3n9)", POA = "palmitoleate (16:1n7)", PA = "palmitate (16:0)")
sapply(M, function(x) FA %in% x$ann$CHEMICAL_NAME) |> `rownames<-`(names(FA))
```

```
##       CECAL LIVER PLASMA
## LA     TRUE  TRUE   TRUE
## ALA    TRUE  TRUE   TRUE
## SDA    TRUE  TRUE   TRUE
## DGLA   TRUE  TRUE   TRUE
## EDA    TRUE  TRUE   TRUE
## ARA    TRUE  TRUE   TRUE
## EPA    TRUE  TRUE   TRUE
## ADR    TRUE  TRUE   TRUE
## n6DPA  TRUE  TRUE   TRUE
## n3DPA  TRUE  TRUE   TRUE
## DHA    TRUE  TRUE   TRUE
## MEAD   TRUE  TRUE   TRUE
## POA    TRUE  TRUE   TRUE
## PA     TRUE  TRUE   TRUE
```

The platform reports 18:3 and 20:3 without resolving the n-3 and n-6
isomers; they are labeled ALA (α-linolenate) and DGLA
(dihomo-γ-linolenate) below.

```
pufa_lab <- c(SDA = "18:4n3", EPA = "EPA 20:5n3", n3DPA = "n3-DPA 22:5n3", ALA = "ALA 18:3n3", DHA = "DHA 22:6n3",
              LA = "LA 18:2n6", EDA = "20:2n6", ARA = "ARA 20:4n6", ADR = "22:4n6", n6DPA = "n6-DPA 22:5n6")
pufa <- bind_rows(DA) |> filter(name %in% FA[names(pufa_lab)]) |>
  mutate(abbr = names(FA)[match(name, FA)], label = pufa_lab[abbr], family = ifelse(grepl("n3", label), "n-3", "n-6"))
pufa |> select(matrix, label, log2fc, q, n_missing_control, n_missing_bean) |> pivot_wider(names_from = matrix, values_from = c(log2fc, q, n_missing_control, n_missing_bean)) |>
  ktab(caption = "Free long-chain PUFA: log2 fold change and q-value by matrix")
```

Free long-chain PUFA: log2 fold change and q-value by matrix

| label | log2fc\_CECAL | log2fc\_LIVER | log2fc\_PLASMA | q\_CECAL | q\_LIVER | q\_PLASMA | n\_missing\_control\_CECAL | n\_missing\_control\_LIVER | n\_missing\_control\_PLASMA | n\_missing\_bean\_CECAL | n\_missing\_bean\_LIVER | n\_missing\_bean\_PLASMA |
| --- | --- | --- | --- | --- | --- | --- | --- | --- | --- | --- | --- | --- |
| LA 18:2n6 | 0.000 | 0.075 | 0.449 | 0.4 | 0.93 | 0.12 | 0 | 0 | 0 | 0 | 0 | 0 |
| ARA 20:4n6 | 0.048 | -0.803 | -1.194 | 0.36 | 0.0017 | 0.00017 | 0 | 0 | 0 | 0 | 0 | 0 |
| 20:2n6 | 0.350 | -0.795 | -0.607 | 0.16 | 0.019 | 0.1 | 0 | 0 | 0 | 0 | 0 | 0 |
| EPA 20:5n3 | 1.216 | 2.667 | 2.225 | 0.034 | 0.00033 | 0.00019 | 0 | 0 | 0 | 0 | 0 | 0 |
| DHA 22:6n3 | 0.771 | 0.219 | 0.423 | 0.05 | 0.3 | 0.11 | 0 | 0 | 0 | 0 | 0 | 0 |
| n3-DPA 22:5n3 | 1.146 | 1.777 | 1.517 | 0.031 | 0.0012 | 0.0043 | 0 | 0 | 0 | 0 | 0 | 0 |
| 22:4n6 | -0.671 | -1.298 | -1.370 | 0.67 | 0.0001 | 0.0065 | 0 | 0 | 0 | 0 | 0 | 0 |
| 18:4n3 | -0.801 | 2.608 | 3.050 | 0.57 | 0.0008 | 1.3e-05 | 3 | 0 | 0 | 6 | 0 | 0 |
| ALA 18:3n3 | 1.504 | 0.983 | 2.058 | 0.03 | 0.047 | 0.00035 | 0 | 0 | 0 | 0 | 0 | 0 |
| n6-DPA 22:5n6 | -2.571 | -2.600 | -2.515 | 0.12 | 0.00065 | 0.00067 | 0 | 0 | 0 | 0 | 3 | 0 |

```
mead <- DA$LIVER |> filter(name == FA["MEAD"]); mead_p <- DA$PLASMA |> filter(name == FA["MEAD"])
```

In liver and plasma, n-3 species were higher with bean feeding (EPA
+2.67 and +2.22; stearidonate +2.61 and +3.05; n-3 DPA +1.78 and +1.52),
and ≥20-carbon n-6 species were lower (arachidonate -0.80 and -1.19;
adrenate -1.30 and -1.37; n-6 DPA -2.60 and -2.51), all at q < 0.006.
Linoleate did not differ in any matrix (q ≥ 0.12). Mead acid was lower
in liver (q = 2.1e-04) and was below detection in 6 of 7 bean plasma
samples.

## 11.1 Pairwise fatty-acid ratios

For two metabolites, the ratio of median-scaled abundances differs
from the molar ratio by a constant, so the between-group fold change in
a pairwise ratio equals the fold change in the molar ratio. Per-sample
log_2_ ratios are compared by Welch’s t-test (fold change with
95% CI; Cohen’s d) and the Mann–Whitney test; eight ratios (n-3/n-6
pairs at matched chain length, product/precursor pairs on each arm, and
mead acid/arachidonate) are FDR-corrected within matrix.

```
cohen_d <- function(y, g) (mean(y[g == "Bean"]) - mean(y[g == "Control"])) / sqrt((var(y[g == "Bean"]) + var(y[g == "Control"])) / 2)
fa_log_ratio <- function(m, num, den) {
  x <- M[[m]]$x; a <- M[[m]]$ann; id <- function(n) a$CHEM_ID[match(FA[n], a$CHEMICAL_NAME)]
  log2(x[, id(num)]) - log2(x[, id(den)])
}
ratio_test <- function(m, num, den) {
  g <- M[[m]]$g; r <- fa_log_ratio(m, num, den); tt <- t.test(r[g == "Bean"], r[g == "Control"])
  tibble(matrix = m, ratio = paste0(num, "/", den), fold = 2^unname(diff(rev(tt$estimate))), ci_low = 2^tt$conf.int[1], ci_high = 2^tt$conf.int[2],
         welch_t = unname(tt$statistic), df = unname(tt$parameter), p = tt$p.value, cohen_d = cohen_d(r, g), mw_p = mw_test(r[g == "Bean"], r[g == "Control"]))
}
ratio_set <- tribble(~num, ~den, ~type,
  "EPA", "ARA", "n-3/n-6, 20C", "n3DPA", "n6DPA", "n-3/n-6, 22:5", "n3DPA", "ADR", "n-3/n-6, 22C", "DHA", "ARA", "n-3/n-6 end products",
  "ARA", "LA", "n-6 product/precursor", "SDA", "ALA", "n-3 product/precursor (Δ6 step)", "EPA", "ALA", "n-3 product/precursor", "MEAD", "ARA", "n-9/n-6")
table_s6 <- bind_rows(lapply(names(M), function(m) bind_rows(lapply(seq_len(nrow(ratio_set)), function(i) ratio_test(m, ratio_set$num[i], ratio_set$den[i]))) |>
  mutate(q = p.adjust(p, "BH")))) |> left_join(ratio_set |> transmute(ratio = paste0(num, "/", den), type), by = "ratio") |> relocate(type, .after = ratio)
table_s6 |> ktab(caption = "Table S6. Pairwise PUFA ratios (bean/control fold change of the per-sample ratio, Welch 95% CI; q within matrix)", digits = 3)
```

Table S6. Pairwise PUFA ratios (bean/control fold change of the
per-sample ratio, Welch 95% CI; q within matrix)

| matrix | ratio | type | fold | ci\_low | ci\_high | welch\_t | df | p | cohen\_d | mw\_p | q |
| --- | --- | --- | --- | --- | --- | --- | --- | --- | --- | --- | --- |
| CECAL | EPA/ARA | n-3/n-6, 20C | 2.685 | 1.834 | 3.932 | 5.673 | 11.438 | 0.00012 | 3.032 | 0.00058 | 0.00033 |
| CECAL | n3DPA/n6DPA | n-3/n-6, 22:5 | 17.396 | 8.455 | 35.789 | 8.767 | 10.484 | 3.8e-06 | 4.686 | 0.00058 | 3e-05 |
| CECAL | n3DPA/ADR | n-3/n-6, 22C | 4.978 | 2.790 | 8.881 | 6.057 | 11.704 | 6.3e-05 | 3.238 | 0.0012 | 0.00025 |
| CECAL | DHA/ARA | n-3/n-6 end products | 2.289 | 1.600 | 3.275 | 5.373 | 7.673 | 0.00077 | 2.872 | 0.0023 | 0.0012 |
| CECAL | ARA/LA | n-6 product/precursor | 0.811 | 0.399 | 1.648 | -0.644 | 11.973 | 0.53 | -0.344 | 0.53 | 0.53 |
| CECAL | SDA/ALA | n-3 product/precursor (Δ6 step) | 0.098 | 0.036 | 0.265 | -5.229 | 9.523 | 0.00045 | -2.795 | 0.00058 | 0.0009 |
| CECAL | EPA/ALA | n-3 product/precursor | 0.595 | 0.312 | 1.134 | -1.765 | 11.388 | 0.1 | -0.944 | 0.097 | 0.14 |
| CECAL | MEAD/ARA | n-9/n-6 | 0.607 | 0.186 | 1.983 | -0.920 | 11.859 | 0.38 | -0.492 | 0.32 | 0.43 |
| LIVER | EPA/ARA | n-3/n-6, 20C | 10.436 | 7.561 | 14.405 | 16.217 | 9.979 | 1.7e-08 | 8.669 | 0.00058 | 6.8e-08 |
| LIVER | n3DPA/n6DPA | n-3/n-6, 22:5 | 22.347 | 10.269 | 48.630 | 9.605 | 6.477 | 4.6e-05 | 5.134 | 0.00058 | 5.2e-05 |
| LIVER | n3DPA/ADR | n-3/n-6, 22C | 8.018 | 5.702 | 11.275 | 14.632 | 6.573 | 2.9e-06 | 7.821 | 0.00058 | 4.7e-06 |
| LIVER | DHA/ARA | n-3/n-6 end products | 2.037 | 1.760 | 2.357 | 11.091 | 8.620 | 2.1e-06 | 5.929 | 0.00058 | 4.7e-06 |
| LIVER | ARA/LA | n-6 product/precursor | 0.554 | 0.461 | 0.665 | -7.042 | 11.708 | 1.5e-05 | -3.764 | 0.00058 | 2.1e-05 |
| LIVER | SDA/ALA | n-3 product/precursor (Δ6 step) | 3.047 | 2.739 | 3.390 | 23.323 | 9.903 | 5.5e-10 | 12.467 | 0.00058 | 4.4e-09 |
| LIVER | EPA/ALA | n-3 product/precursor | 3.212 | 2.632 | 3.920 | 13.879 | 6.917 | 2.6e-06 | 7.419 | 0.00058 | 4.7e-06 |
| LIVER | MEAD/ARA | n-9/n-6 | 0.514 | 0.380 | 0.694 | -4.926 | 10.056 | 0.00059 | -2.633 | 0.00058 | 0.00059 |
| PLASMA | EPA/ARA | n-3/n-6, 20C | 10.328 | 8.067 | 13.222 | 21.701 | 8.210 | 1.5e-08 | 11.600 | 0.00058 | 1.2e-07 |
| PLASMA | n3DPA/n6DPA | n-3/n-6, 22:5 | 17.618 | 8.869 | 34.996 | 9.817 | 7.246 | 1.9e-05 | 5.247 | 0.00058 | 3.8e-05 |
| PLASMA | n3DPA/ADR | n-3/n-6, 22C | 7.832 | 4.056 | 15.124 | 7.291 | 7.538 | 0.00011 | 3.897 | 0.00058 | 0.00018 |
| PLASMA | DHA/ARA | n-3/n-6 end products | 3.051 | 2.504 | 3.718 | 13.415 | 6.825 | 3.7e-06 | 7.171 | 0.00058 | 1.5e-05 |
| PLASMA | ARA/LA | n-6 product/precursor | 0.323 | 0.250 | 0.419 | -10.074 | 7.857 | 9.1e-06 | -5.385 | 0.00058 | 2.4e-05 |
| PLASMA | SDA/ALA | n-3 product/precursor (Δ6 step) | 2.141 | 1.468 | 3.121 | 4.424 | 11.411 | 0.00094 | 2.365 | 0.0023 | 0.0012 |
| PLASMA | EPA/ALA | n-3 product/precursor | 1.122 | 0.671 | 1.877 | 0.538 | 6.577 | 0.61 | 0.288 | 0.53 | 0.61 |
| PLASMA | MEAD/ARA | n-9/n-6 | 0.649 | 0.528 | 0.798 | -4.768 | 8.503 | 0.0012 | -2.549 | 0.0012 | 0.0014 |

```
write_xlsx(table_s6, file.path(out_dir, "tables", "Table_S6_PUFA_ratios.xlsx"))
r6 <- function(m, r) table_s6 |> filter(matrix == m, ratio == r)
fci <- function(m, r) with(r6(m, r), sprintf("%.1f-fold (95%% CI %.1f–%.1f; p = %s)", fold, ci_low, ci_high, fmt_p(p)))
```

The EPA:arachidonate ratio was 10.4-fold (95% CI 7.6–14.4; p =
1.7e-08) higher in liver, 10.3-fold (95% CI 8.1–13.2; p = 1.5e-08) in
plasma, and 2.7-fold (95% CI 1.8–3.9; p = 1.2e-04) in cecal content. The
n-3:n-6 DPA ratio was 17.4-, 22.3-, 17.6-fold higher in cecal content,
liver, and plasma. Arachidonate:linoleate was 0.55-fold in liver (95% CI
0.46–0.67) and 0.32-fold in plasma (0.25–0.42), and
stearidonate:α-linolenate was 3.0-fold in liver (2.7–3.4).

```
fa_ord <- names(pufa_lab)
pufa_panel <- function(m, ttl) {
  d <- pufa |> filter(matrix == m) |> mutate(label = factor(label, rev(pufa_lab)), sig = q < 0.05)
  ggplot(d, aes(log2fc, label)) + geom_col(aes(fill = interaction(family, sig), colour = family), width = 0.7) + geom_vline(xintercept = 0) +
    scale_colour_manual(values = c(`n-3` = "#1F7A3E", `n-6` = "#B22E2E"), name = NULL,
                        guide = guide_legend(override.aes = list(fill = c("#1F7A3E", "#B22E2E")))) +
    scale_fill_manual(values = c(`n-3.TRUE` = "#1F7A3E", `n-6.TRUE` = "#B22E2E", `n-3.FALSE` = "white", `n-6.FALSE` = "white"), guide = "none") +
    labs(title = ttl, x = expression(log[2]~FC~"(bean/control)"), y = NULL)
}
rat_pts <- bind_rows(lapply(names(M), function(m) bind_rows(lapply(list(c("EPA", "ARA"), c("n3DPA", "n6DPA")), function(r) {
  a <- M[[m]]$ann; x <- M[[m]]$x; id <- function(n) a$CHEM_ID[match(FA[n], a$CHEMICAL_NAME)]
  v <- log2(x[, id(r[1])]) - log2(x[, id(r[2])]); g <- M[[m]]$g
  tibble(matrix = m, ratio = paste0(r[1], "/", r[2]), group = g, value = v - mean(v[g == "Control"]))
}))))
rat_lab <- table_s6 |> filter(ratio %in% c("EPA/ARA", "n3DPA/n6DPA")) |>
  mutate(lab = sprintf("%s\n%.1f×%s\n[%.1f–%.1f]", c(CECAL = "Cecal", LIVER = "Liver", PLASMA = "Plasma")[matrix], fold,
                       ifelse(p < 0.001, "***", ifelse(p < 0.01, "**", ifelse(p < 0.05, "*", ""))), ci_low, ci_high))
rat_pts <- rat_pts |> left_join(rat_lab |> select(matrix, ratio, lab), by = c("matrix", "ratio")) |>
  mutate(ratio = factor(ratio, c("EPA/ARA", "n3DPA/n6DPA"), c("EPA / arachidonate", "n3-DPA / n6-DPA")))
f5c <- ggplot(rat_pts, aes(lab, value, shape = group, colour = interaction(group, matrix))) +
  geom_point(position = position_dodge(width = 0.5), size = 2) +
  stat_summary(aes(group = group), fun = mean, fun.min = mean, fun.max = mean, geom = "crossbar", width = 0.25, linewidth = 0.3, position = position_dodge(width = 0.5)) +
  facet_wrap(~ratio, scales = "free_x") + scale_shape_manual(values = grp_shp, name = NULL) +
  scale_colour_manual(values = c(setNames(rep("grey50", 3), paste0("Control.", names(M))), setNames(mat_col, paste0("Bean.", names(M)))), guide = "none") +
  labs(title = "C  Pairwise n-3 : n-6 ratios", x = NULL, y = expression(log[2]~"ratio (relative to control mean)")) +
  theme(axis.text.x = element_text(size = 7))
f5 <- (pufa_panel("LIVER", "A  Liver") | pufa_panel("PLASMA", "B  Plasma") | f5c) + plot_layout(widths = c(1, 1, 1.6), guides = "collect") &
  theme(legend.position = "top")
f5
```

```
save_fig(f5, "Figure_5_PUFA", 11, 4.6)
```

## 11.2 Dietary supply versus tissue response

Estimated dietary supply (bean/control, from the diet section) is set
against the measured tissue fold changes for the PUFA and BCAA.

```
tis <- function(m, nm) DA[[m]]$log2fc[DA[[m]]$name == nm]
s1a <- tibble(fa = c("LA", "ALA", "EPA", "ARA", "n6DPA"),
              lab = c("LA 18:2n6\n(n-6 parent)", "ALA 18:3n3\n(n-3 parent)", "EPA 20:5n3\n(n-3 product)", "ARA 20:4n6\n(n-6 product)", "n6-DPA 22:5n6\n(n-6 product)"),
              Diet = c(log2(s1("Linoleate")$ratio_70_0), log2(s1("α-Linolenate")$ratio_70_0), NA, NA, NA)) |>
  mutate(Liver = sapply(fa, function(k) tis("LIVER", FA[k])), Plasma = sapply(fa, function(k) tis("PLASMA", FA[k]))) |>
  pivot_longer(c(Diet, Liver, Plasma), names_to = "source", values_to = "log2fc") |> mutate(lab = factor(lab, unique(lab)))
s1b <- tibble(aa = c("Valine", "Leucine", "Isoleucine"), Diet = log2(c(s1("Valine")$ratio_70_0, s1("Leucine")$ratio_70_0, s1("Isoleucine")$ratio_70_0))) |>
  mutate(Plasma = sapply(tolower(aa), function(k) tis("PLASMA", k)), Cecal = sapply(tolower(aa), function(k) tis("CECAL", k))) |>
  pivot_longer(c(Diet, Plasma, Cecal), names_to = "source", values_to = "log2fc") |> mutate(aa = factor(aa, c("Valine", "Leucine", "Isoleucine")))
src_col <- c(Diet = "grey65", Liver = unname(mat_col["LIVER"]), Plasma = unname(mat_col["PLASMA"]), Cecal = unname(mat_col["CECAL"]))
fs1a <- ggplot(s1a, aes(lab, log2fc, fill = source)) + geom_col(position = position_dodge(0.8), width = 0.75, na.rm = TRUE) + geom_hline(yintercept = 0) +
  geom_text(data = s1a |> filter(source == "Diet", is.na(log2fc)), aes(x = as.numeric(lab) - 0.27, y = 0.1, label = "none in diet"), inherit.aes = FALSE, angle = 90, hjust = 0, size = 2.6, colour = "grey40") +
  scale_fill_manual(values = src_col, labels = c(Diet = "Diet (estimated supply)", Liver = "Liver", Plasma = "Plasma"), name = NULL) +
  labs(title = "A  PUFA: dietary supply vs. tissue", x = NULL, y = expression(log[2]~"fold change (bean/control)"))
fs1b <- ggplot(s1b, aes(aa, log2fc, fill = factor(source, c("Diet", "Plasma", "Cecal")))) + geom_col(position = position_dodge(0.8), width = 0.75) + geom_hline(yintercept = 0) +
  scale_fill_manual(values = src_col, labels = c(Diet = "Diet (estimated supply)", Plasma = "Plasma", Cecal = "Cecal content"), name = NULL) +
  labs(title = "B  BCAA: dietary supply vs. tissue", x = NULL, y = expression(log[2]~"fold change (bean/control)"))
fs1 <- fs1a + fs1b + plot_layout(widths = c(1.4, 1))
fs1
```

```
save_fig(fs1, "Figure_S1_diet_vs_tissue", 11, 4.5)
```

Linoleate supply was unchanged (log_2_ ratio +0.03) while
its ≥20-carbon products were lower in liver and plasma; α-linolenate
supply was 1.54-fold higher, and EPA, absent from both diets, was higher
in liver and plasma. Estimated BCAA supply was lower in the bean diet
(log_2_ ratio -0.50, -0.43, -0.42 for valine, leucine,
isoleucine); plasma BCAA were lower in the same direction, whereas cecal
BCAA were higher.

# 12 Bile acids, γ-glutamyl-cycle metabolites, ketone bodies, and acylcarnitines

```
grp_sum <- function(sub) bind_rows(DA) |> filter(sub_pathway %in% sub, !is.na(p)) |> group_by(matrix, sub_pathway) |>
  summarise(n = n(), higher_q = sum(q < 0.05 & log2fc > 0), lower_q = sum(q < 0.05 & log2fc < 0), .groups = "drop")
grp_sum(c("Primary Bile Acid Metabolism", "Secondary Bile Acid Metabolism")) |> ktab(caption = "Bile acids: features changed at FDR < 0.05")
```

Bile acids: features changed at FDR < 0.05

| matrix | sub\_pathway | n | higher\_q | lower\_q |
| --- | --- | --- | --- | --- |
| CECAL | Primary Bile Acid Metabolism | 11 | 0 | 6 |
| CECAL | Secondary Bile Acid Metabolism | 20 | 2 | 7 |
| LIVER | Primary Bile Acid Metabolism | 9 | 0 | 1 |
| LIVER | Secondary Bile Acid Metabolism | 16 | 4 | 3 |
| PLASMA | Primary Bile Acid Metabolism | 9 | 0 | 0 |
| PLASMA | Secondary Bile Acid Metabolism | 12 | 0 | 0 |

```
grp_sum(c("Gamma-glutamyl Amino Acid", "Glutathione Metabolism")) |> ktab(caption = "γ-Glutamyl amino acids and glutathione metabolism: features changed at FDR < 0.05")
```

γ-Glutamyl amino acids and glutathione metabolism: features changed at
FDR < 0.05

| matrix | sub\_pathway | n | higher\_q | lower\_q |
| --- | --- | --- | --- | --- |
| CECAL | Gamma-glutamyl Amino Acid | 16 | 0 | 3 |
| CECAL | Glutathione Metabolism | 3 | 1 | 1 |
| LIVER | Gamma-glutamyl Amino Acid | 16 | 12 | 0 |
| LIVER | Glutathione Metabolism | 14 | 5 | 0 |
| PLASMA | Gamma-glutamyl Amino Acid | 18 | 6 | 0 |
| PLASMA | Glutathione Metabolism | 8 | 2 | 0 |

```
grp_sum(c("Ketone Bodies", "Fatty Acid Metabolism (Acyl Carnitine, Hydroxy)", "Fatty Acid Metabolism (Acyl Carnitine, Dicarboxylate)",
          "Fatty Acid Metabolism (Acyl Carnitine, Short Chain)", "Fatty Acid Metabolism (Acyl Carnitine, Medium Chain)")) |>
  ktab(caption = "Ketone bodies and acylcarnitines: features changed at FDR < 0.05")
```

Ketone bodies and acylcarnitines: features changed at FDR < 0.05

| matrix | sub\_pathway | n | higher\_q | lower\_q |
| --- | --- | --- | --- | --- |
| CECAL | Fatty Acid Metabolism (Acyl Carnitine, Dicarboxylate) | 2 | 1 | 0 |
| CECAL | Fatty Acid Metabolism (Acyl Carnitine, Hydroxy) | 1 | 0 | 0 |
| CECAL | Fatty Acid Metabolism (Acyl Carnitine, Medium Chain) | 1 | 1 | 0 |
| CECAL | Fatty Acid Metabolism (Acyl Carnitine, Short Chain) | 1 | 0 | 1 |
| CECAL | Ketone Bodies | 1 | 1 | 0 |
| LIVER | Fatty Acid Metabolism (Acyl Carnitine, Dicarboxylate) | 3 | 3 | 0 |
| LIVER | Fatty Acid Metabolism (Acyl Carnitine, Hydroxy) | 7 | 4 | 0 |
| LIVER | Fatty Acid Metabolism (Acyl Carnitine, Medium Chain) | 5 | 0 | 0 |
| LIVER | Fatty Acid Metabolism (Acyl Carnitine, Short Chain) | 1 | 0 | 0 |
| LIVER | Ketone Bodies | 1 | 0 | 0 |
| PLASMA | Fatty Acid Metabolism (Acyl Carnitine, Dicarboxylate) | 5 | 0 | 0 |
| PLASMA | Fatty Acid Metabolism (Acyl Carnitine, Hydroxy) | 8 | 0 | 0 |
| PLASMA | Fatty Acid Metabolism (Acyl Carnitine, Medium Chain) | 5 | 0 | 0 |
| PLASMA | Fatty Acid Metabolism (Acyl Carnitine, Short Chain) | 1 | 0 | 0 |
| PLASMA | Ketone Bodies | 1 | 0 | 0 |

```
sel <- bind_rows(DA) |> filter(name %in% c("ophthalmate", "3-hydroxybutyrate (BHBA)", "(R)-3-hydroxybutyrylcarnitine", "(S)-3-hydroxybutyrylcarnitine")) |>
  select(matrix, name, log2fc, q)
sel |> ktab(caption = "Selected metabolites")
```

Selected metabolites

| matrix | name | log2fc | q |
| --- | --- | --- | --- |
| CECAL | 3-hydroxybutyrate (BHBA) | 1.237 | 0.031 |
| CECAL | (R)-3-hydroxybutyrylcarnitine | -0.365 | 0.29 |
| LIVER | 3-hydroxybutyrate (BHBA) | 0.183 | 0.37 |
| LIVER | ophthalmate | 3.451 | 0.00058 |
| LIVER | (R)-3-hydroxybutyrylcarnitine | 2.588 | 2.5e-05 |
| LIVER | (S)-3-hydroxybutyrylcarnitine | 1.232 | 0.004 |
| PLASMA | 3-hydroxybutyrate (BHBA) | 1.210 | 0.052 |
| PLASMA | ophthalmate | 3.448 | 0.0021 |
| PLASMA | (R)-3-hydroxybutyrylcarnitine | 1.680 | 0.36 |
| PLASMA | (S)-3-hydroxybutyrylcarnitine | 0.850 | 0.51 |

```
s_ <- function(m, n) sel |> filter(matrix == m, name == n)
```

Ophthalmate was higher in liver (q = 5.8e-04) and plasma (q = 0.002).
In liver, (R)- and (S)-3-hydroxybutyrylcarnitine were higher
(log_2_FC +2.59 and +1.23), whereas free 3-hydroxybutyrate did
not differ (q = 0.37).

# 13 Cross-compartment concordance

Features are matched across matrices by CHEM\_ID. Spearman
correlations use host features measured in both matrices.

```
hostDA <- lapply(DA, function(d) d |> filter(host))
cor_pair <- function(a, b) { x <- inner_join(hostDA[[a]] |> select(CHEM_ID, fa = log2fc), hostDA[[b]] |> select(CHEM_ID, fb = log2fc), by = "CHEM_ID")
  ct <- cor.test(x$fa, x$fb, method = "spearman", exact = FALSE); tibble(pair = paste(a, b, sep = "–"), n = nrow(x), rho = unname(ct$estimate), p = ct$p.value) }
cc_cor <- bind_rows(cor_pair("LIVER", "PLASMA"), cor_pair("CECAL", "LIVER"), cor_pair("CECAL", "PLASMA"))
cc_cor |> ktab(caption = "Spearman correlation of log2 fold changes between matrices (host features)", digits = 3)
```

Spearman correlation of log2 fold changes between matrices (host
features)

| pair | n | rho | p |
| --- | --- | --- | --- |
| LIVER–PLASMA | 687 | 0.480 | 8e-41 |
| CECAL–LIVER | 546 | 0.125 | 0.0035 |
| CECAL–PLASMA | 513 | 0.124 | 0.0049 |

Liver and plasma fold changes were correlated (ρ = 0.48, n = 687, p =
8.0e-41); correlations of cecal content with liver and plasma were weak
(ρ = 0.12 and 0.12).

Sensitivity of the liver–plasma correlation to the feature set.

```
alls <- function(f) { x <- inner_join(f(DA$LIVER) |> select(CHEM_ID, a = log2fc), f(DA$PLASMA) |> select(CHEM_ID, b = log2fc), by = "CHEM_ID")
  c(n = nrow(x), rho = cor(x$a, x$b, method = "spearman")) }
rbind(all_tested = alls(function(d) filter(d, !is.na(p))), named_tested = alls(function(d) filter(d, !is.na(p), type == "NAMED")),
      host = alls(function(d) filter(d, host))) |> round(3)
```

```
##                n   rho
## all_tested   737 0.508
## named_tested 697 0.495
## host         687 0.480
```

```
wide <- Reduce(function(a, b) inner_join(a, b, by = "CHEM_ID"), lapply(names(hostDA), function(m)
  hostDA[[m]] |> select(CHEM_ID, name, super_pathway, log2fc, p, q) |> rename_with(~ paste0(.x, ".", m), -CHEM_ID)))
conc <- wide |> filter(p.CECAL < 0.05, p.LIVER < 0.05, p.PLASMA < 0.05,
                       sign(log2fc.CECAL) == sign(log2fc.LIVER), sign(log2fc.LIVER) == sign(log2fc.PLASMA)) |>
  mutate(all_fdr = q.CECAL < 0.05 & q.LIVER < 0.05 & q.PLASMA < 0.05) |>
  transmute(CHEM_ID, name = name.CECAL, super_pathway = super_pathway.CECAL, log2fc_cecal = log2fc.CECAL, log2fc_liver = log2fc.LIVER, log2fc_plasma = log2fc.PLASMA,
            q_cecal = q.CECAL, q_liver = q.LIVER, q_plasma = q.PLASMA, all_fdr) |> arrange(super_pathway, log2fc_cecal)
table_s3 <- conc
table_s3 |> ktab(caption = "Table S3. Host metabolites with p < 0.05 and the same direction in all three matrices")
```

Table S3. Host metabolites with p < 0.05 and the same direction in
all three matrices

| CHEM\_ID | name | super\_pathway | log2fc\_cecal | log2fc\_liver | log2fc\_plasma | q\_cecal | q\_liver | q\_plasma | all\_fdr |
| --- | --- | --- | --- | --- | --- | --- | --- | --- | --- |
| 100000463 | indolelactate | Amino Acid | -2.310 | -0.722 | -0.928 | 0.067 | 0.01 | 0.0093 | FALSE |
| 100001721 | N2-acetyllysine | Amino Acid | 0.496 | 0.899 | 0.408 | 0.056 | 0.00045 | 0.12 | FALSE |
| 100001734 | N6-acetyllysine | Amino Acid | 0.587 | 0.455 | 0.667 | 0.013 | 0.0029 | 3.9e-05 | TRUE |
| 100001253 | N-acetylglutamine | Amino Acid | 0.738 | 0.664 | 0.987 | 0.039 | 0.031 | 0.007 | TRUE |
| 100015962 | N,N,N-trimethyl-5-aminovalerate | Amino Acid | 1.330 | 1.651 | 1.536 | 0.0043 | 2.5e-05 | 0.0003 | TRUE |
| 100001541 | 2-hydroxy-3-methylvalerate | Amino Acid | 1.899 | 0.802 | 0.392 | 0.00026 | 0.0036 | 0.1 | FALSE |
| 100000706 | alpha-hydroxyisocaproate | Amino Acid | 2.951 | 1.705 | 1.372 | 2.6e-05 | 0.0076 | 0.0062 | TRUE |
| 100001491 | N-glycolylneuraminate | Carbohydrate | -0.851 | -0.232 | -0.683 | 0.005 | 0.062 | 0.027 | FALSE |
| 100000257 | glucuronate | Carbohydrate | 0.488 | 0.487 | 0.406 | 0.037 | 0.0023 | 0.0018 | TRUE |
| 482 | lactate | Carbohydrate | 2.065 | 0.311 | 0.362 | 0.016 | 0.026 | 0.0011 | TRUE |
| 100001468 | N1-methyl-2-pyridone-5-carboxamide | Cofactors and Vitamins | -2.649 | -0.764 | -1.293 | 0.031 | 0.0031 | 0.0017 | TRUE |
| 100003258 | 2-O-methylascorbic acid | Cofactors and Vitamins | -1.616 | -1.011 | -0.863 | 0.053 | 4.1e-05 | 0.00075 | FALSE |
| 1105 | alpha-tocopherol | Cofactors and Vitamins | -0.374 | -0.427 | -0.620 | 0.00068 | 0.019 | 0.0097 | TRUE |
| 100001586 | gulonate\* | Cofactors and Vitamins | 2.208 | 0.744 | 0.772 | 0.00017 | 0.00066 | 0.011 | TRUE |
| 1124 | citrate | Energy | 3.278 | 2.453 | 0.423 | 0.0003 | 0.00023 | 0.0064 | TRUE |
| 330 | fumarate | Energy | 4.110 | 1.317 | 1.354 | 9e-05 | 0.00042 | 0.00033 | TRUE |
| 100001869 | 1-stearoyl-2-arachidonoyl-GPC (18:0/20:4) | Lipid | -2.606 | -0.656 | -0.771 | 0.0002 | 6.2e-05 | 0.0031 | TRUE |
| 100008914 | 1-palmitoyl-2-arachidonoyl-GPC (16:0/20:4n6) | Lipid | -2.187 | -0.377 | -0.585 | 0.0015 | 0.00024 | 0.0083 | TRUE |
| 100003000 | 1-(1-enyl-palmitoyl)-GPE (P-16:0)\* | Lipid | -2.169 | -0.402 | -0.179 | 0.00082 | 0.036 | 0.091 | FALSE |
| 100003869 | taurohyodeoxycholic acid | Lipid | -1.776 | -1.007 | -0.977 | 0.0034 | 0.026 | 0.095 | FALSE |
| 100008904 | 1-stearoyl-2-oleoyl-GPC (18:0/18:1) | Lipid | -1.398 | -0.800 | -0.507 | 0.027 | 0.00024 | 0.048 | TRUE |
| 100001551 | 1-arachidonoyl-GPC (20:4n6)\* | Lipid | -1.379 | -0.851 | -0.957 | 0.015 | 0.00017 | 1.1e-05 | TRUE |
| 1539 | 1-palmitoyl-2-oleoyl-GPC (16:0/18:1) | Lipid | -1.189 | -0.275 | -0.463 | 0.072 | 0.00022 | 0.016 | FALSE |
| 1547 | N-stearoyl-sphingosine (d18:1/18:0)\* | Lipid | -0.993 | -1.691 | -2.044 | 0.00061 | 0.00048 | 0.00099 | TRUE |
| 100001606 | cholesterol sulfate | Lipid | -0.838 | -0.237 | -0.698 | 8.1e-06 | 0.05 | 0.0026 | FALSE |
| 363 | myo-inositol | Lipid | 0.365 | 0.937 | 1.025 | 0.056 | 0.00048 | 0.0072 | FALSE |
| 100009217 | 1,2-dilinoleoyl-GPE (18:2/18:2)\* | Lipid | 0.913 | 2.417 | 2.590 | 0.026 | 5.9e-05 | 0.0084 | TRUE |
| 100001181 | docosapentaenoate (n3 DPA; 22:5n3) | Lipid | 1.146 | 1.777 | 1.517 | 0.031 | 0.0012 | 0.0043 | TRUE |
| 2050 | eicosapentaenoate (EPA; 20:5n3) | Lipid | 1.216 | 2.667 | 2.225 | 0.034 | 0.00033 | 0.00019 | TRUE |
| 100001570 | 1-linoleoyl-GPE (18:2)\* | Lipid | 1.473 | 1.555 | 0.631 | 0.032 | 0.0001 | 0.11 | FALSE |
| 100001337 | linolenate [alpha or gamma; (18:3n3 or 6)] | Lipid | 1.504 | 0.983 | 2.058 | 0.03 | 0.047 | 0.00035 | TRUE |
| 100003397 | trimethylamine N-oxide | Lipid | 1.520 | 0.921 | 0.859 | 0.0018 | 0.036 | 0.083 | FALSE |
| 100001870 | 1-palmitoyl-2-linoleoyl-GPE (16:0/18:2) | Lipid | 2.223 | 1.933 | 1.957 | 0.00069 | 4e-05 | 5.5e-05 | TRUE |
| 100001778 | 1-linoleoyl-GPI (18:2)\* | Lipid | 2.339 | 0.862 | 0.909 | 0.0043 | 0.01 | 0.0014 | TRUE |
| 880 | adenine | Nucleotide | -1.974 | -0.376 | -1.605 | 0.011 | 0.042 | 0.003 | TRUE |
| 821 | pseudouridine | Nucleotide | 1.294 | 0.259 | 0.266 | 0.0059 | 0.1 | 0.044 | FALSE |

```
write_xlsx(table_s3, file.path(out_dir, "tables", "Table_S3_concordant_metabolites.xlsx"))
```

Of 450 host features measured in all three matrices, 36 differed at p
< 0.05 in the same direction in all three, 23 of them at FDR <
0.05 in all three.

Concordant features that the exposure-marker classification removes
from the host set.

```
bind_rows(lapply(names(DA), function(m) DA[[m]] |> filter(exposure, !is.na(p)) |> select(CHEM_ID, name, log2fc, p) |> mutate(matrix = m))) |>
  pivot_wider(names_from = matrix, values_from = c(log2fc, p)) |>
  filter(p_CECAL < 0.05, p_LIVER < 0.05, p_PLASMA < 0.05, sign(log2fc_CECAL) == sign(log2fc_LIVER), sign(log2fc_LIVER) == sign(log2fc_PLASMA)) |> ktab()
```

| CHEM\_ID | name | log2fc\_CECAL | log2fc\_LIVER | log2fc\_PLASMA | p\_CECAL | p\_LIVER | p\_PLASMA |
| --- | --- | --- | --- | --- | --- | --- | --- |
| 194 | N-formylmethionine | 1.949 | 0.677 | 3.619 | 2.6e-05 | 0.0027 | 1.7e-08 |
| 1025 | pipecolate | 0.450 | 5.469 | 4.569 | 0.022 | 1.8e-10 | 1.8e-09 |
| 100002154 | ergothioneine | 1.814 | 5.108 | 1.020 | 0.012 | 2.8e-13 | 0.0013 |
| 100002749 | S-methylcysteine | 3.964 | 4.447 | 3.538 | 3.6e-07 | 5.7e-07 | 2e-07 |
| 100004523 | N-delta-acetylornithine | 1.387 | 4.156 | 4.256 | 2e-05 | 7.3e-09 | 2.1e-09 |
| 100004568 | S-(3-hydroxypropyl)mercapturic acid (HPMA) | 1.602 | 1.081 | 0.517 | 0.013 | 0.0073 | 0.0068 |
| 100005383 | N-methylpipecolate | 4.635 | 1.298 | 2.126 | 1.7e-08 | 0.0099 | 0.018 |

```
f2d <- conc |> mutate(lab = paste0(name, ifelse(all_fdr, " †", "")), lab = factor(lab, rev(lab))) |>
  pivot_longer(starts_with("log2fc_"), names_to = "matrix", values_to = "log2fc") |>
  mutate(matrix = factor(c(log2fc_cecal = "Cecal", log2fc_liver = "Liver", log2fc_plasma = "Plasma")[matrix], c("Cecal", "Liver", "Plasma")))
f2 <- ggplot(f2d, aes(matrix, lab, fill = log2fc)) + geom_tile(colour = "white") + geom_text(aes(label = sprintf("%+.2f", log2fc)), size = 2.8) +
  scale_fill_distiller(palette = "RdYlGn", direction = 1, limits = c(-4.2, 4.2), name = expression(log[2]~FC)) +
  facet_grid(super_pathway ~ ., scales = "free_y", space = "free_y", switch = "y") +
  labs(x = NULL, y = NULL, title = sprintf("%d host metabolites concordant in all three matrices", nrow(conc)), subtitle = "† FDR < 0.05 in all three") +
  theme(strip.placement = "outside", strip.background = element_blank(), strip.text.y.left = element_text(angle = 0, size = 8, face = "bold"), legend.position = "right", axis.text.y = element_text(size = 8), plot.title.position = "plot")
f2
```

```
save_fig(f2, "Figure_2_concordant_heatmap", 7.5, 11)
```

# 14 KEGG enrichment and pathway topology

Quantitative pathway analysis follows the MetaboAnalyst 6.0 procedure
using its *Mus musculus* KEGG pathway library
(`mmu.qs`) and compound database
(`compound_db.qs`), stored locally in
`metaboanalyst_lib/`. Named compounds with an HMDB identifier
are mapped to KEGG through the compound database (for entries carrying
several HMDB identifiers, the last identifier is used). Data are
glog_10_-transformed (MetaboAnalyst LogNorm) and Pareto-scaled
without sample normalization. Each pathway with at least one matched
compound is tested with the global test (`globaltest::gt`);
FDR (BH) is computed across tested pathways within matrix. Impact is the
sum of relative betweenness centrality of the matched compounds.

```
lib_dir <- "metaboanalyst_lib"
cdb <- qs2::qs_read(file.path(lib_dir, "compound_db.qs")); klib <- qs2::qs_read(file.path(lib_dir, "mmu.qs"))
glog10 <- function(x) { mv <- min(abs(x[x != 0])) / 10; log10((x + sqrt(x^2 + mv^2)) / 2) }
KEGG <- lapply(names(M), function(m) {
  x <- M[[m]]$x; a <- M[[m]]$ann[match(colnames(x), M[[m]]$ann$CHEM_ID), ]
  sel <- a$TYPE == "NAMED" & !is.na(a$HMDB); hm <- sub(".*,", "", a$HMDB[sel])
  kg <- cdb$kegg_id[match(hm, cdb$hmdb_id)]; ok <- !is.na(kg) & kg != ""
  X <- x[, sel][, ok]; kg <- kg[ok]; X <- X[, !duplicated(kg)]; kg <- kg[!duplicated(kg)]
  X <- glog10(X); X <- pareto(X); colnames(X) <- kg; X <- X[, is.finite(colSums(X))]
  res <- bind_rows(lapply(names(klib$mset.list), function(pid) {
    hit <- intersect(klib$mset.list[[pid]], colnames(X)); if (!length(hit)) return(NULL)
    tibble(pathway_id = pid, pathway = names(klib$path.ids)[match(pid, klib$path.ids)], total = length(klib$mset.list[[pid]]), hits = length(hit),
           p = p.value(gt(M[[m]]$g, X[, hit, drop = FALSE])), impact = sum(klib$rbc.list[[pid]][hit]))
  })) |> mutate(holm = p.adjust(p, "holm"), FDR = p.adjust(p, "fdr"), matrix = m) |> arrange(p)
  list(res = res, n_hmdb = sum(sel), n_in_db = sum(hm %in% cdb$hmdb_id), n_kegg = ncol(X))
}) |> setNames(names(M))
sapply(KEGG, function(k) c(named_with_HMDB = k$n_hmdb, matched_in_compound_db = k$n_in_db, unique_KEGG_used = k$n_kegg,
                           pathways_tested = nrow(k$res), FDR_lt_0.05 = sum(k$res$FDR < 0.05)))
```

```
##                        CECAL LIVER PLASMA
## named_with_HMDB          635   793    755
## matched_in_compound_db   595   738    694
## unique_KEGG_used         376   409    355
## pathways_tested           62    63     64
## FDR_lt_0.05               57    34     36
```

```
kegg_all <- bind_rows(lapply(KEGG, `[[`, "res")) |> relocate(matrix)
kegg_all |> group_by(matrix) |> slice_min(p, n = 5) |> ktab(caption = "Five most significant pathways per matrix (global test)", digits = 3)
```

Five most significant pathways per matrix (global test)

| matrix | pathway\_id | pathway | total | hits | p | impact | holm | FDR |
| --- | --- | --- | --- | --- | --- | --- | --- | --- |
| CECAL | mmu00600 | Sphingolipid metabolism | 32 | 7 | 2.8e-10 | 0.500 | 1.7e-08 | 1e-08 |
| CECAL | mmu00240 | Pyrimidine metabolism | 39 | 17 | 3.3e-10 | 0.594 | 2e-08 | 1e-08 |
| CECAL | mmu00330 | Arginine and proline metabolism | 36 | 15 | 1.2e-09 | 0.680 | 7.3e-08 | 2.5e-08 |
| CECAL | mmu00670 | One carbon pool by folate | 26 | 12 | 2e-09 | 0.477 | 1.2e-07 | 3.1e-08 |
| CECAL | mmu00230 | Purine metabolism | 71 | 17 | 5.9e-09 | 0.243 | 3.4e-07 | 7.4e-08 |
| LIVER | mmu00564 | Glycerophospholipid metabolism | 36 | 14 | 5.7e-09 | 0.697 | 3.6e-07 | 2.7e-07 |
| LIVER | mmu00565 | Ether lipid metabolism | 20 | 3 | 9.3e-09 | 0.082 | 5.8e-07 | 2.7e-07 |
| LIVER | mmu00310 | Lysine degradation | 30 | 10 | 1.3e-08 | 0.287 | 7.7e-07 | 2.7e-07 |
| LIVER | mmu00220 | Arginine biosynthesis | 14 | 12 | 9.2e-08 | 0.628 | 5.5e-06 | 1.5e-06 |
| LIVER | mmu00770 | Pantothenate and CoA biosynthesis | 20 | 12 | 1.2e-07 | 0.748 | 7.3e-06 | 1.6e-06 |
| PLASMA | mmu00310 | Lysine degradation | 30 | 8 | 1.1e-13 | 0.245 | 6.8e-12 | 6.8e-12 |
| PLASMA | mmu01040 | Biosynthesis of unsaturated fatty acids | 36 | 10 | 1.5e-09 | 0.000 | 9.7e-08 | 4.9e-08 |
| PLASMA | mmu00592 | alpha-Linolenic acid metabolism | 13 | 3 | 9.5e-08 | 0.333 | 5.9e-06 | 2e-06 |
| PLASMA | mmu00220 | Arginine biosynthesis | 14 | 12 | 7e-06 | 0.628 | 0.00042 | 0.00011 |
| PLASMA | mmu00020 | Citrate cycle (TCA cycle) | 20 | 6 | 1.5e-05 | 0.258 | 0.00091 | 0.00017 |

```
kw <- kegg_all |> select(matrix, pathway_id, pathway, FDR, impact) |> pivot_wider(names_from = matrix, values_from = c(FDR, impact))
k22 <- kw |> filter(FDR_CECAL < 0.05, FDR_LIVER < 0.05, FDR_PLASMA < 0.05) |>
  mutate(mean_mlog10 = (-log10(FDR_CECAL) - log10(FDR_LIVER) - log10(FDR_PLASMA)) / 3) |> arrange(desc(mean_mlog10))
table_s5 <- k22 |> arrange(desc(impact_LIVER)) |> select(pathway_id, pathway, FDR_CECAL, impact_CECAL, FDR_LIVER, impact_LIVER, FDR_PLASMA, impact_PLASMA)
table_s5 |> ktab(caption = "Table S5. KEGG pathways with FDR < 0.05 in all three matrices, sorted by liver impact", digits = 2)
```

Table S5. KEGG pathways with FDR < 0.05 in all three matrices, sorted
by liver impact

| pathway\_id | pathway | FDR\_CECAL | impact\_CECAL | FDR\_LIVER | impact\_LIVER | FDR\_PLASMA | impact\_PLASMA |
| --- | --- | --- | --- | --- | --- | --- | --- |
| mmu00053 | Ascorbate and aldarate metabolism | 6.9e-05 | 0.68 | 3.4e-05 | 0.84 | 0.00055 | 0.68 |
| mmu00250 | Alanine, aspartate and glutamate metabolism | 8.1e-08 | 0.74 | 3.2e-06 | 0.76 | 0.00017 | 0.74 |
| mmu00270 | Cysteine and methionine metabolism | 2.6e-07 | 0.69 | 0.00095 | 0.76 | 0.00032 | 0.55 |
| mmu00770 | Pantothenate and CoA biosynthesis | 5.3e-06 | 0.14 | 1.6e-06 | 0.75 | 0.017 | 0.12 |
| mmu00040 | Pentose and glucuronate interconversions | 0.00013 | 0.40 | 0.0001 | 0.71 | 0.0017 | 0.51 |
| mmu00564 | Glycerophospholipid metabolism | 0.0043 | 0.42 | 2.7e-07 | 0.70 | 0.0042 | 0.59 |
| mmu00330 | Arginine and proline metabolism | 2.5e-08 | 0.68 | 2.2e-05 | 0.68 | 0.00031 | 0.49 |
| mmu00220 | Arginine biosynthesis | 2e-07 | 0.63 | 1.5e-06 | 0.63 | 0.00011 | 0.63 |
| mmu00360 | Phenylalanine metabolism | 8.1e-08 | 0.62 | 0.027 | 0.62 | 0.0042 | 0.62 |
| mmu00240 | Pyrimidine metabolism | 1e-08 | 0.59 | 0.0023 | 0.61 | 0.018 | 0.39 |
| mmu00760 | Nicotinate and nicotinamide metabolism | 1.1e-05 | 0.33 | 0.00018 | 0.55 | 0.00052 | 0.33 |
| mmu00480 | Glutathione metabolism | 0.00017 | 0.22 | 0.0018 | 0.50 | 0.0072 | 0.15 |
| mmu00620 | Pyruvate metabolism | 8.8e-07 | 0.19 | 0.019 | 0.41 | 0.00031 | 0.19 |
| mmu00600 | Sphingolipid metabolism | 1e-08 | 0.50 | 0.00011 | 0.36 | 0.0098 | 0.60 |
| mmu00020 | Citrate cycle (TCA cycle) | 4.1e-07 | 0.26 | 5e-05 | 0.35 | 0.00017 | 0.26 |
| mmu00592 | alpha-Linolenic acid metabolism | 0.023 | 0.33 | 0.00019 | 0.33 | 2e-06 | 0.33 |
| mmu00310 | Lysine degradation | 0.00016 | 0.27 | 2.7e-07 | 0.29 | 6.8e-12 | 0.25 |
| mmu00350 | Tyrosine metabolism | 8.1e-08 | 0.27 | 0.016 | 0.25 | 0.0061 | 0.25 |
| mmu00630 | Glyoxylate and dicarboxylate metabolism | 5.1e-07 | 0.19 | 0.00093 | 0.24 | 0.00031 | 0.25 |
| mmu00562 | Inositol phosphate metabolism | 0.013 | 0.13 | 0.044 | 0.19 | 0.00082 | 0.13 |
| mmu01040 | Biosynthesis of unsaturated fatty acids | 0.016 | 0.00 | 0.0042 | 0.18 | 4.9e-08 | 0.00 |
| mmu00140 | Steroid hormone biosynthesis | 1.5e-05 | 0.01 | 0.002 | 0.01 | 0.00031 | 0.01 |

```
write_xlsx(list(Table_S5 = table_s5, all_pathways_by_matrix = kegg_all), file.path(out_dir, "tables", "Table_S5_KEGG_pathways.xlsx"))
kp <- function(id, m) kw[[paste0("FDR_", m)]][kw$pathway_id == id]
liv_top <- KEGG$LIVER$res[1, ]
```

22 pathways reached FDR < 0.05 in all three matrices. In liver,
the pathway with the smallest p-value was Glycerophospholipid metabolism
(FDR = 2.7e-07; impact 0.70). Sphingolipid metabolism was significant in
all three matrices. Primary bile-acid biosynthesis had FDR = 0.013,
0.005, 0.204 and valine, leucine, and isoleucine degradation FDR =
4.5e-06, 0.163, 0.014 (cecal content, liver, plasma). α-Linolenic acid
metabolism was significant in all three matrices and linoleic acid
metabolism had FDR = 0.316, 9.3e-04, 0.072.

```
f3d <- k22 |> mutate(pathway = factor(pathway, rev(pathway))) |>
  pivot_longer(c(FDR_CECAL, FDR_LIVER, FDR_PLASMA, impact_CECAL, impact_LIVER, impact_PLASMA), names_to = c(".value", "matrix"), names_sep = "_") |>
  mutate(mlog = pmin(-log10(FDR), 8), matrix = factor(c(CECAL = "Cecal", LIVER = "Liver", PLASMA = "Plasma")[matrix], c("Cecal", "Liver", "Plasma")))
f3 <- ggplot(f3d, aes(matrix, pathway, fill = mlog)) + geom_tile(colour = "white") +
  geom_text(aes(label = sprintf("%.2f", impact), colour = mlog > 5), size = 3) +
  scale_colour_manual(values = c(`TRUE` = "white", `FALSE` = "black"), guide = "none") +
  scale_fill_distiller(palette = "Blues", direction = 1, limits = c(0, 8), name = expression(-log[10]~FDR)) +
  labs(x = NULL, y = NULL, title = sprintf("%d KEGG pathways with FDR < 0.05 in all three matrices\n(cell value = pathway impact)", nrow(k22))) +
  theme(legend.position = "right")
f3
```

```
save_fig(f3, "Figure_3_KEGG", 7.5, 9)
```

# 15 Hepatic transcripts

```
rna <- as.data.frame(suppressWarnings(read_excel(file.path(data_dir, "Expr_Browser_-_70v0_FM_DRS_-_Liver_GE_condensed.xlsx"))))
cpm <- as.matrix(rna[, grep("^Male", names(rna))]); rownames(cpm) <- rna$Identifier
c(genes = nrow(cpm), male_samples = ncol(cpm), duplicated_symbols = sum(duplicated(rna$Name)), missing_values = sum(is.na(cpm)))
```

```
##              genes       male_samples duplicated_symbols     missing_values 
##              25527                 20                  0                 20
```

```
summary(colSums(cpm, na.rm = TRUE))
```

```
##    Min. 1st Qu.  Median    Mean 3rd Qu.    Max. 
##  878653  969202 1046155 1021996 1069783 1194002
```

The workbook holds gene-level TMM-normalized CPM for 10 male pools
per diet (column sums near 10^6^). Genes with CPM ≥ 1 in at
least 5 of the 20 pools are retained and analyzed on log_2_(CPM
+ 1) with limma-trend (empirical-Bayes moderated t with a mean–variance
trend), with BH-FDR across retained genes.

```
rgrp <- factor(ifelse(grepl("70%", colnames(cpm)), "Bean", "Control"), c("Control", "Bean"))
keep <- rowSums(cpm >= 1, na.rm = TRUE) >= 5
ly <- log2(cpm[keep, ] + 1)
fit <- eBayes(lmFit(ly, model.matrix(~rgrp)), trend = TRUE)
tt_all <- topTable(fit, coef = 2, number = Inf, sort.by = "none") |> rownames_to_column("Identifier") |>
  mutate(gene = rna$Name[match(Identifier, rna$Identifier)]) |> relocate(gene)
c(retained = sum(keep), prior_df = round(fit$df.prior, 2), residual_df = fit$df.residual[1])
```

```
##    retained    prior_df residual_df 
##    12116.00        4.74       18.00
```

```
# Expression Browser sample k of each diet corresponds to the k-th pool of that diet in ascending pool order
rna_pool <- tibble(sample = colnames(cpm), group = rgrp, k = as.integer(str_extract(colnames(cpm), "\\d+$"))) |>
  mutate(POOL = ifelse(group == "Control", 5 * (k - 1) + 1, 5 * (k - 1) + 4), shared = POOL %in% unlist(prof_pools))
s7_file <- file.path(data_dir, "COSU-01-23VW_liver_transcripts_DataS7.xlsx")
pp20 <- read_excel(s7_file, "perpool_CPM_all20") |> mutate(POOL = as.integer(sub("P", "", pool_id)))
map_check <- sapply(c("Fads2", "Fads1", "Elovl5", "Elovl2", "Scd1"), function(gn)
  max(abs(cpm[rna$Name == gn, rna_pool$sample] - pp20[[gn]][match(rna_pool$POOL, pp20$POOL)])))
map_check
```

```
##  Fads2  Fads1 Elovl5 Elovl2   Scd1 
##      0      0      0      0      0
```

```
stopifnot(all(map_check < 0.01))
```

The per-pool CPM table of the transcript workbook
(`COSU-01-23VW_liver_transcripts_DataS7.xlsx`, sheet
`perpool_CPM_all20`) identifies each RNA sample by pool
number; its values agree with the Expression Browser columns under this
mapping (maximum absolute difference 0 CPM). The 14 pools profiled by
metabolomics are the same two-animal pools in both assays.

```
gsets <- list(`PUFA desaturation/elongation` = c("Fads2", "Fads1", "Elovl5", "Elovl2"), `Lipogenesis (SREBP-1c targets)` = c("Scd1", "Elovl6", "Fasn", "Acaca"),
              `Fatty-acid oxidation (PPARα axis)` = c("Ppara", "Acox1", "Cpt1a", "Cyp4a14"), `BCAA catabolism` = c("Bcat2", "Bckdha", "Bckdhb", "Dbt", "Dld", "Bckdk", "Ppm1k"),
              `Glutathione` = c("Gclc", "Gclm", "Gss", "Chac1", "Gsr"))
gs <- enframe(gsets, "gene_set", "gene") |> unnest(gene)
sh <- rna_pool$shared
gcpm <- function(gn, cols = TRUE) cpm[rna$Name == gn, cols]
s7a <- gs |> left_join(tt_all |> select(gene, logFC, AveExpr, t, P.Value, adj.P.Val), by = "gene") |>
  mutate(pct_control = 100 * 2^logFC,
         mean_cpm_control = sapply(gene, function(gn) mean(gcpm(gn, rgrp == "Control"))),
         mean_cpm_bean    = sapply(gene, function(gn) mean(gcpm(gn, rgrp == "Bean"))),
         log2fc_14 = sapply(gene, function(gn) { v <- log2(gcpm(gn, sh) + 1); mean(v[rgrp[sh] == "Bean"]) - mean(v[rgrp[sh] == "Control"]) }),
         welch_p_14 = sapply(gene, function(gn) { v <- log2(gcpm(gn, sh) + 1); t.test(v[rgrp[sh] == "Bean"], v[rgrp[sh] == "Control"])$p.value }),
         mw_p_14    = sapply(gene, function(gn) { v <- log2(gcpm(gn, sh) + 1); mw_test(v[rgrp[sh] == "Bean"], v[rgrp[sh] == "Control"]) }))
s7a |> select(gene_set, gene, mean_cpm_control, mean_cpm_bean, logFC, adj.P.Val, pct_control, log2fc_14, welch_p_14, mw_p_14) |>
  ktab(caption = "Table S7, part A. Hepatic transcripts for five gene sets (limma-trend, 10 pools per diet, FDR across 12,116 genes; Welch and Mann–Whitney on the 7 + 7 pools shared with metabolomics)", digits = 2)
```

Table S7, part A. Hepatic transcripts for five gene sets (limma-trend,
10 pools per diet, FDR across 12,116 genes; Welch and Mann–Whitney on
the 7 + 7 pools shared with metabolomics)

| gene\_set | gene | mean\_cpm\_control | mean\_cpm\_bean | logFC | adj.P.Val | pct\_control | log2fc\_14 | welch\_p\_14 | mw\_p\_14 |
| --- | --- | --- | --- | --- | --- | --- | --- | --- | --- |
| PUFA desaturation/elongation | Fads2 | 1585.45 | 720.81 | -1.16 | 2.3e-07 | 44.86 | -1.30 | 5.1e-08 | 0.00058 |
| PUFA desaturation/elongation | Fads1 | 1016.57 | 628.92 | -0.71 | 1.8e-05 | 61.10 | -0.78 | 1.7e-05 | 0.00058 |
| PUFA desaturation/elongation | Elovl5 | 1778.44 | 911.38 | -0.99 | 2.2e-05 | 50.51 | -1.13 | 0.00027 | 0.00058 |
| PUFA desaturation/elongation | Elovl2 | 799.66 | 598.92 | -0.44 | 0.0061 | 73.50 | -0.52 | 0.0028 | 0.0023 |
| Lipogenesis (SREBP-1c targets) | Scd1 | 25523.33 | 10725.38 | -1.28 | 2.2e-05 | 41.17 | -1.46 | 5.7e-05 | 0.00058 |
| Lipogenesis (SREBP-1c targets) | Elovl6 | 368.51 | 186.94 | -0.95 | 0.0004 | 51.93 | -1.19 | 9.9e-06 | 0.00058 |
| Lipogenesis (SREBP-1c targets) | Fasn | 2625.22 | 1562.61 | -0.69 | 0.0054 | 61.81 | -0.89 | 0.00016 | 0.00058 |
| Lipogenesis (SREBP-1c targets) | Acaca | 346.99 | 202.32 | -0.74 | 7.4e-05 | 59.78 | -0.86 | 3.4e-05 | 0.00058 |
| Fatty-acid oxidation (PPARα axis) | Ppara | 106.37 | 85.72 | -0.28 | 0.069 | 82.27 | -0.41 | 0.0034 | 0.0023 |
| Fatty-acid oxidation (PPARα axis) | Acox1 | 2734.91 | 2538.45 | -0.10 | 0.51 | 93.20 | -0.14 | 0.07 | 0.053 |
| Fatty-acid oxidation (PPARα axis) | Cpt1a | 502.28 | 346.81 | -0.53 | 2.3e-06 | 69.17 | -0.60 | 4.9e-07 | 0.00058 |
| Fatty-acid oxidation (PPARα axis) | Cyp4a14 | 438.96 | 530.06 | 0.22 | 0.53 | 116.46 | 0.18 | 0.61 | 0.71 |
| BCAA catabolism | Bcat2 | 9.40 | 11.90 | 0.31 | 0.012 | 124.36 | 0.34 | 0.0068 | 0.0041 |
| BCAA catabolism | Bckdha | 258.76 | 266.37 | 0.04 | 0.72 | 102.97 | 0.02 | 0.76 | 0.62 |
| BCAA catabolism | Bckdhb | 62.62 | 57.03 | -0.14 | 0.24 | 90.83 | -0.18 | 0.062 | 0.053 |
| BCAA catabolism | Dbt | 58.94 | 67.52 | 0.20 | 0.036 | 114.94 | 0.22 | 0.041 | 0.017 |
| BCAA catabolism | Dld | 121.95 | 118.90 | -0.04 | 0.67 | 97.56 | -0.05 | 0.34 | 0.21 |
| BCAA catabolism | Bckdk | 14.59 | 15.16 | 0.06 | 0.61 | 103.90 | 0.02 | 0.83 | 1 |
| BCAA catabolism | Ppm1k | 62.33 | 72.01 | 0.22 | 0.056 | 116.42 | 0.17 | 0.19 | 0.21 |
| Glutathione | Gclc | 558.91 | 586.83 | 0.06 | 0.66 | 104.41 | 0.13 | 0.28 | 0.38 |
| Glutathione | Gclm | 198.51 | 256.86 | 0.37 | 4.6e-06 | 129.13 | 0.38 | 1.6e-07 | 0.00058 |
| Glutathione | Gss | 76.82 | 81.00 | 0.09 | 0.4 | 106.24 | 0.16 | 0.1 | 0.097 |
| Glutathione | Chac1 | 3.06 | 3.54 | 0.17 | 0.57 | 112.58 | 0.57 | 0.023 | 0.011 |
| Glutathione | Gsr | 88.79 | 97.75 | 0.14 | 0.013 | 110.01 | 0.13 | 0.004 | 0.0023 |

```
g7 <- function(gn) s7a |> filter(gene == gn)
```

Transcripts of Fads2 (log_2_FC = -1.16, q = 2.3e-07), Elovl5
(-0.99, q = 2.2e-05), Fads1 (-0.71, q = 1.8e-05), and Elovl2 (-0.44, q =
0.006) were lower with bean feeding, at 45%, 51%, 61%, 74% of control;
in the 14 shared pools, Welch p ≤ 0.003. Scd1, Elovl6, Fasn, and Acaca
were lower by 0.69–1.28 log_2_ units (Scd1 q = 2.2e-05). Ppara
was modestly lower (-0.28, q = 0.07), Acox1 did not differ (q = 0.51),
and Cpt1a was lower (-0.53). Among BCAA-catabolism transcripts, Bckdha,
Bckdhb, Dld, Bckdk, and Ppm1k did not differ (q ≥ 0.06), whereas Dbt
(+0.20, q = 0.036) and Bcat2 (+0.31, q = 0.012; 9–12 CPM) were modestly
higher. Among glutathione-synthesis transcripts, Gclm (+0.37, q =
4.6e-06) and Gsr (+0.14, q = 0.013) were higher, and Gclc, Gss, and
Chac1 did not differ.

## 15.1 Fatty-acid indices and transcript–index relations

Product-to-precursor indices are formed per pool from the liver
batch-normalized imputed data, as for the pairwise ratios.

```
idx_def <- tribble(~index, ~num, ~den, ~step, ~transcripts,
  "DGLA/LA", "DGLA", "LA", "Δ6 + ELOVL5, n-6", "Fads2, Elovl5",
  "ARA/DGLA", "ARA", "DGLA", "Δ5, n-6", "Fads1",
  "ARA/LA", "ARA", "LA", "n-6 arm, C18 → C20", "Fads2, Elovl5, Fads1",
  "ADR/ARA", "ADR", "ARA", "ELOVL2/5, n-6", "Elovl2, Elovl5",
  "SDA/ALA", "SDA", "ALA", "Δ6, n-3", "Fads2",
  "EPA/ALA", "EPA", "ALA", "n-3 arm, C18 → C20", "Fads2, Elovl5, Fads1",
  "n3DPA/EPA", "n3DPA", "EPA", "ELOVL2, n-3", "Elovl2",
  "DHA/EPA", "DHA", "EPA", "ELOVL2 + Δ6 + β-oxidation, n-3", "Elovl2, Fads2",
  "EPA/ARA", "EPA", "ARA", "end products, n-3 vs. n-6", "",
  "POA/PA", "POA", "PA", "SCD1", "Scd1",
  "MEAD", "MEAD", NA, "Δ6/Δ5 product of oleate (single metabolite)", "Fads2, Fads1")
la <- M$LIVER$ann; lx <- M$LIVER$x; lid <- function(n) la$CHEM_ID[match(FA[n], la$CHEMICAL_NAME)]
fa_idx <- sapply(seq_len(nrow(idx_def)), function(i) log2(lx[, lid(idx_def$num[i])]) - if (is.na(idx_def$den[i])) 0 else log2(lx[, lid(idx_def$den[i])]))
colnames(fa_idx) <- idx_def$index; rownames(fa_idx) <- M$LIVER$mmeta$POOL
idx_tab <- bind_rows(lapply(seq_len(nrow(idx_def)), function(i) { v <- fa_idx[, idx_def$index[i]]; g <- M$LIVER$g; t <- t.test(v[g == "Bean"], v[g == "Control"])
  tibble(index = idx_def$index[i], step = idx_def$step[i], fold = 2^unname(diff(rev(t$estimate))), ci_low = 2^t$conf.int[1], ci_high = 2^t$conf.int[2],
         welch_p = t$p.value, mw_p = mw_test(v[g == "Bean"], v[g == "Control"]), transcripts = idx_def$transcripts[i],
         transcript_log2fc = paste(sprintf("%.2f", s7a$logFC[match(strsplit(idx_def$transcripts[i], ", ")[[1]], s7a$gene)]), collapse = ", ")) }))
idx_tab |> ktab(caption = "Table S7, part B. Liver fatty-acid indices in the 14 shared pools (geometric-mean fold change of per-pool ratios; Welch's t-test on log2 ratios) and transcripts for the corresponding step", digits = 3)
```

Table S7, part B. Liver fatty-acid indices in the 14 shared pools
(geometric-mean fold change of per-pool ratios; Welch’s t-test on log2
ratios) and transcripts for the corresponding step

| index | step | fold | ci\_low | ci\_high | welch\_p | mw\_p | transcripts | transcript\_log2fc |
| --- | --- | --- | --- | --- | --- | --- | --- | --- |
| DGLA/LA | Δ6 + ELOVL5, n-6 | 0.709 | 0.593 | 0.848 | 0.0014 | 0.0041 | Fads2, Elovl5 | -1.16, -0.99 |
| ARA/DGLA | Δ5, n-6 | 0.781 | 0.648 | 0.941 | 0.014 | 0.017 | Fads1 | -0.71 |
| ARA/LA | n-6 arm, C18 → C20 | 0.554 | 0.461 | 0.665 | 1.5e-05 | 0.00058 | Fads2, Elovl5, Fads1 | -1.16, -0.99, -0.71 |
| ADR/ARA | ELOVL2/5, n-6 | 0.711 | 0.597 | 0.847 | 0.0015 | 0.0023 | Elovl2, Elovl5 | -0.44, -0.99 |
| SDA/ALA | Δ6, n-3 | 3.047 | 2.739 | 3.390 | 5.5e-10 | 0.00058 | Fads2 | -1.16 |
| EPA/ALA | n-3 arm, C18 → C20 | 3.212 | 2.632 | 3.920 | 2.6e-06 | 0.00058 | Fads2, Elovl5, Fads1 | -1.16, -0.99, -0.71 |
| n3DPA/EPA | ELOVL2, n-3 | 0.546 | 0.480 | 0.622 | 3.6e-07 | 0.00058 | Elovl2 | -0.44 |
| DHA/EPA | ELOVL2 + Δ6 + β-oxidation, n-3 | 0.195 | 0.138 | 0.276 | 7.1e-06 | 0.00058 | Elovl2, Fads2 | -0.44, -1.16 |
| EPA/ARA | end products, n-3 vs. n-6 | 10.436 | 7.561 | 14.405 | 1.7e-08 | 0.00058 |  |  |
| POA/PA | SCD1 | 0.547 | 0.445 | 0.672 | 3.7e-05 | 0.0012 | Scd1 | -1.28 |
| MEAD | Δ6/Δ5 product of oleate (single metabolite) | 0.290 | 0.215 | 0.390 | 1.3e-05 | 0.00058 | Fads2, Fads1 | -1.16, -0.71 |

```
dha <- DA$LIVER |> filter(name == FA["DHA"])
ix <- function(i) idx_tab |> filter(index == i)
```

On the n-6 arm, DGLA:LA (0.71-fold), ARA:DGLA (0.78), ARA:LA (0.55),
and adrenate:ARA (0.71) were lower (Welch p ≤ 0.014). On the n-3 arm,
SDA:ALA (3.0-fold) and EPA:ALA (3.2) were higher, whereas n-3 DPA:EPA
(0.55) and DHA:EPA (0.20) were lower; DHA itself did not differ
(log_2_FC = +0.22, q = 0.30). Mead acid was 0.29-fold and
palmitoleate:palmitate 0.55-fold of control.

```
pairs <- tribble(~gene, ~index, "Fads2", "ARA/LA", "Fads2", "DGLA/LA", "Fads1", "ARA/DGLA", "Elovl5", "ARA/LA", "Fads2", "SDA/ALA",
                 "Fads2", "EPA/ALA", "Elovl2", "n3DPA/EPA", "Elovl2", "DHA/EPA", "Elovl2", "ADR/ARA", "Scd1", "POA/PA")
rp <- rna_pool |> filter(shared)
tx <- function(gn) setNames(log2(cpm[rna$Name == gn, rp$sample]), rp$POOL)  # log2 CPM
sp_p <- function(x, y) { ct <- suppressWarnings(cor.test(x, y, method = "spearman")); c(rho = unname(ct$estimate), p = ct$p.value) }
cor_tab <- bind_rows(lapply(seq_len(nrow(pairs)), function(i) {
  x <- tx(pairs$gene[i]); y <- fa_idx[names(x), pairs$index[i]]; g <- rp$group
  a <- sp_p(x, y); wc <- sp_p(x[g == "Control"], y[g == "Control"]); wb <- sp_p(x[g == "Bean"], y[g == "Bean"])
  pc <- cor.test(x[g == "Control"], y[g == "Control"]); pb <- cor.test(x[g == "Bean"], y[g == "Bean"])
  tibble(gene = pairs$gene[i], index = pairs$index[i], pearson_14 = cor(x, y), spearman_14 = a["rho"], spearman_14_p = a["p"],
         spearman_control = wc["rho"], spearman_control_p = wc["p"], spearman_bean = wb["rho"], spearman_bean_p = wb["p"],
         pearson_control = unname(pc$estimate), pearson_control_lo = pc$conf.int[1], pearson_control_hi = pc$conf.int[2],
         pearson_bean = unname(pb$estimate), pearson_bean_lo = pb$conf.int[1], pearson_bean_hi = pb$conf.int[2])
}))
cor_tab |> select(gene, index, pearson_14, spearman_14, spearman_14_p, spearman_control, spearman_control_p, spearman_bean, spearman_bean_p) |>
  ktab(caption = "Table S7, part C. Transcript–index correlations (log2 CPM vs. log2 index), 14 shared pools and within each diet (n = 7)", digits = 2)
```

Table S7, part C. Transcript–index correlations (log2 CPM vs. log2
index), 14 shared pools and within each diet (n = 7)

| gene | index | pearson\_14 | spearman\_14 | spearman\_14\_p | spearman\_control | spearman\_control\_p | spearman\_bean | spearman\_bean\_p |
| --- | --- | --- | --- | --- | --- | --- | --- | --- |
| Fads2 | ARA/LA | 0.87 | 0.71 | 0.006 | -0.57 | 0.2 | 0.21 | 0.66 |
| Fads2 | DGLA/LA | 0.81 | 0.71 | 0.0056 | -0.04 | 0.96 | 0.36 | 0.44 |
| Fads1 | ARA/DGLA | 0.53 | 0.45 | 0.11 | 0.04 | 0.96 | -0.57 | 0.2 |
| Elovl5 | ARA/LA | 0.88 | 0.80 | 0.001 | -0.07 | 0.91 | 0.43 | 0.35 |
| Fads2 | SDA/ALA | -0.97 | -0.77 | 0.0019 | 0.29 | 0.56 | -0.43 | 0.35 |
| Fads2 | EPA/ALA | -0.96 | -0.80 | 0.001 | 0.07 | 0.91 | -0.43 | 0.35 |
| Elovl2 | n3DPA/EPA | 0.69 | 0.68 | 0.0095 | -0.25 | 0.59 | 0.00 | 1 |
| Elovl2 | DHA/EPA | 0.81 | 0.78 | 0.0014 | -0.21 | 0.66 | 0.79 | 0.048 |
| Elovl2 | ADR/ARA | 0.75 | 0.77 | 0.0021 | 0.04 | 0.96 | 0.79 | 0.048 |
| Scd1 | POA/PA | 0.85 | 0.81 | 0.00081 | 0.32 | 0.5 | 0.32 | 0.5 |

```
cr <- function(gn, ixn) cor_tab |> filter(gene == gn, index == ixn)
```

Across the 14 shared pools, Fads2 correlated with ARA:LA (r = +0.87)
and with EPA:ALA (r = -0.96), and Elovl2 with DHA:EPA (r = +0.81). These
across-pool coefficients reflect the separation of the two diet groups
on both axes. Within groups (n = 7), the control and bean Spearman
coefficients had opposite signs for 8 of 10 gene–index pairs.

```
gs_genes <- intersect(rna$Name, gs$gene)
table_s7 <- list(A_gene_sets = s7a, B_fatty_acid_indices = idx_tab, C_transcript_index_correlations = cor_tab,
                 per_pool_cpm = bind_cols(rna_pool, as.data.frame(t(cpm[match(gs_genes, rna$Name), , drop = FALSE])) |> setNames(gs_genes)),
                 per_pool_indices = as.data.frame(fa_idx) |> rownames_to_column("POOL"))
write_xlsx(table_s7, file.path(out_dir, "tables", "Table_S7_hepatic_transcripts.xlsx"))
write_xlsx(tt_all, file.path(out_dir, "tables", "Data_S7_male_liver_limma_trend_all_genes.xlsx"))
```

```
fs2a_d <- s7a |> mutate(gene = factor(gene, rev(gs$gene)), gene_set = factor(gene_set, names(gsets)),
  cls = ifelse(adj.P.Val >= 0.05, "FDR ≥ 0.05", ifelse(logFC < 0, "lower", "higher")),
  lab = ifelse(adj.P.Val < 0.05, paste("q =", formatC(adj.P.Val, format = "e", digits = 1)), "n.s."))
fs2a <- ggplot(fs2a_d, aes(logFC, gene, fill = cls)) + geom_col(width = 0.7) + geom_vline(xintercept = 0) +
  geom_text(aes(x = ifelse(logFC < 0, logFC - 0.03, logFC + 0.03), label = lab, hjust = ifelse(logFC < 0, 1, 0)), size = 2.6) +
  facet_grid(gene_set ~ ., scales = "free_y", space = "free_y") +
  scale_fill_manual(values = c(lower = "#5146C2", higher = "#C47A14", `FDR ≥ 0.05` = "grey70"), name = NULL) +
  scale_x_continuous(expand = expansion(mult = 0.25)) +
  labs(title = "A  Hepatic transcripts, five gene sets", x = expression(log[2]~"fold change, bean/control (10 pools per diet)"), y = NULL) +
  theme(strip.text.y = element_text(angle = 0, hjust = 0), strip.background = element_blank(), axis.text.y = element_text(face = "italic"))
sc_panel <- function(gn, ixn, ttl, ylab) {
  d <- tibble(x = 2^tx(gn), y = 2^fa_idx[names(tx(gn)), ixn], group = rp$group)
  ggplot(d, aes(x, y, colour = group, shape = group)) + geom_point(size = 2.2) +
    scale_x_log10() + scale_y_log10() +
    scale_colour_manual(values = c(Control = "grey40", Bean = "#1A9E77"), name = NULL) + scale_shape_manual(values = c(Control = 16, Bean = 15), name = NULL) +
    labs(subtitle = sprintf("r = %+.2f across 14 pools", cr(gn, ixn)$pearson_14)) +
    labs(title = ttl, x = paste0(gn, ", CPM"), y = ylab)
}
fs2 <- fs2a / (sc_panel("Fads2", "ARA/LA", "B  n-6 index vs. Fads2", "arachidonate / linoleate (scaled)") |
               sc_panel("Fads2", "EPA/ALA", "C  n-3 index vs. Fads2", "EPA / α-linolenate (scaled)") |
               sc_panel("Elovl2", "DHA/EPA", "D  ELOVL2 step vs. Elovl2", "DHA / EPA (scaled)")) +
  plot_layout(heights = c(1.6, 1), guides = "collect") & theme(legend.position = "bottom")
fs2
```

```
save_fig(fs2, "Figure_S2_transcripts_indices", 11, 9.5)
```

# 16 Metabolite composites, diet, and adiposity

Composite membership is read from the composite workbook
(`COSU-01-23VW_perpool_matrix_R2.xlsx`, sheet
`Composite_members`; CHEM\_ID per composite and matrix). Six
classes are defined per matrix (18 composites): ceramides, bile acids, a
matrix-specific BCAA class (keto and hydroxy acids in cecal content,
catabolites in liver, parent BCAA in plasma), n-3 PUFA,
glutathione-cycle metabolites, and ketone bodies with short- and
medium-chain acylcarnitines. Each composite is the per-sample mean of
the Pareto-scaled log_2_ abundances of its members; members
that are constant across the 14 samples carry no information and are
dropped.

```
tissue_key <- c(Cecal = "CECAL", Liver = "LIVER", Plasma = "PLASMA")
memb <- read_excel(s3_file, "Composite_members") |> mutate(matrix = tissue_key[Tissue], CHEM_ID = as.character(CHEM_ID)) |>
  left_join(bind_rows(DA) |> select(matrix, CHEM_ID, name, log2fc, q, p), by = c("matrix", "CHEM_ID"))
stopifnot(!anyNA(memb$name), all(memb$name == memb$CHEMICAL_NAME))
memb <- memb |> mutate(used = !is.na(p))
memb |> group_by(composite = Composite, matrix) |> summarise(listed = n(), used = sum(used), higher_q = sum(used & q < 0.05 & log2fc > 0),
  lower_q = sum(used & q < 0.05 & log2fc < 0), .groups = "drop") |> ktab(caption = "Composite membership: listed and used members, and members changed at FDR < 0.05")
```

Composite membership: listed and used members, and members changed at
FDR < 0.05

| composite | matrix | listed | used | higher\_q | lower\_q |
| --- | --- | --- | --- | --- | --- |
| BCAA ketoacid/hydroxy-acid | CECAL | 6 | 6 | 6 | 0 |
| BCAA-catabolite | LIVER | 5 | 5 | 5 | 0 |
| BCAA-parent | PLASMA | 3 | 3 | 0 | 0 |
| Bile acid | CECAL | 33 | 31 | 2 | 13 |
| Bile acid | LIVER | 25 | 25 | 4 | 4 |
| Bile acid | PLASMA | 22 | 21 | 0 | 0 |
| Ceramide | CECAL | 14 | 14 | 4 | 4 |
| Ceramide | LIVER | 13 | 13 | 0 | 7 |
| Ceramide | PLASMA | 17 | 17 | 0 | 8 |
| Glutathione cycle | CECAL | 19 | 19 | 1 | 4 |
| Glutathione cycle | LIVER | 30 | 30 | 17 | 0 |
| Glutathione cycle | PLASMA | 26 | 26 | 8 | 0 |
| Ketone / FA-oxidation tone | CECAL | 3 | 3 | 1 | 1 |
| Ketone / FA-oxidation tone | LIVER | 10 | 10 | 5 | 0 |
| Ketone / FA-oxidation tone | PLASMA | 12 | 12 | 0 | 0 |
| n-3 PUFA | CECAL | 5 | 5 | 4 | 0 |
| n-3 PUFA | LIVER | 6 | 6 | 5 | 0 |
| n-3 PUFA | PLASMA | 5 | 5 | 4 | 0 |

```
memb |> filter(!used) |> select(Composite, matrix, CHEMICAL_NAME) |> ktab(caption = "Listed members dropped (constant across the 14 samples)")
```

Listed members dropped (constant across the 14 samples)

| Composite | matrix | CHEMICAL\_NAME |
| --- | --- | --- |
| Bile acid | CECAL | taurolithocholate 3-sulfate |
| Bile acid | CECAL | lithocholic acid sulfate (2) |
| Bile acid | PLASMA | 12-ketolithocholate |

```
scores <- bind_rows(lapply(names(M), function(m) {
  z <- pareto(log2(M[[m]]$x[, apply(M[[m]]$x, 2, sd) > 0]))
  mm <- memb |> filter(matrix == m, used)
  bind_rows(lapply(unique(mm$Composite), function(k) tibble(matrix = m, composite = k, sample = rownames(z), POOL = M[[m]]$mmeta$POOL,
    group = M[[m]]$g, n_members = sum(mm$Composite == k), score = rowMeans(z[, mm$CHEM_ID[mm$Composite == k], drop = FALSE]))))
})) |> left_join(sample_ph |> select(-group, -POOL), by = c("matrix", "sample")) |>
  mutate(label = paste(c(CECAL = "Cecal", LIVER = "Liver", PLASMA = "Plasma")[matrix], composite))
stopifnot(!anyNA(scores$SQ_FAT_mg), n_distinct(scores$label) == 18)
```

## 16.1 Adiposity profile of the profiled pools

The class-level models need one covariate vector per pool, so each
pool is linked to the mean phenotype of its two donors (the liver and
cecal linkage); hepatic lipid is the two-donor mean in mg/g dry liver.
The four depot masses are compared between diets as a profile by
two-sample Hotelling’s T² and reduced by principal component analysis of
their z-scores. PC1 is oriented so that control pools score positive,
and PC2 so that its loadings are positive.

```
dep_vars <- c(Subcutaneous = "SQ_FAT_mg", Mesenteric = "MES_FAT_mg", Retroperitoneal = "RP_FAT_mg", Epididymal = "EP_PM_FAT_mg")
pool14 <- pool_ph14 |> left_join(sample_ph |> filter(matrix == "LIVER") |> select(POOL, LIVER_LIPID), by = "POOL") |> arrange(POOL)
stopifnot(nrow(pool14) == 14, !anyNA(pool14$LIVER_LIPID))
depot_pca <- function(d) {
  pc <- prcomp(as.matrix(d[, dep_vars]), scale. = TRUE)
  s <- c(sign(mean(pc$x[d$group == "Control", 1])), sign(sum(pc$rotation[, 2])),
         sign(pc$rotation["EP_PM_FAT_mg", 3]), sign(pc$rotation["MES_FAT_mg", 4]))
  pc$x <- sweep(pc$x, 2, s, "*"); pc$rotation <- sweep(pc$rotation, 2, s, "*"); pc
}
pca_dep <- depot_pca(pool14)
pool14 <- pool14 |> mutate(PC1 = pca_dep$x[, 1], PC2 = pca_dep$x[, 2])

s4a_vars <- c(`Body weight (g)` = "FINAL_BW_g", `Subcutaneous fat (mg)` = "SQ_FAT_mg", `Mesenteric fat (mg)` = "MES_FAT_mg",
              `Retroperitoneal fat (mg)` = "RP_FAT_mg", `Epididymal fat (mg)` = "EP_PM_FAT_mg", `Total visceral fat (mg)` = "VIS_FAT_mg",
              `Total depot mass (mg)` = "TOTAL_DEPOT_mg", `Hepatic lipid (mg/g dry liver)` = "LIVER_LIPID")
table_s4a <- bind_rows(lapply(names(s4a_vars), function(v) {
  x <- pool14[[s4a_vars[v]]]; b <- x[pool14$group == "Bean"]; c0 <- x[pool14$group == "Control"]
  tibble(phenotype = v, control_mean = mean(c0), bean_mean = mean(b), pct_diff = 100 * (mean(b) / mean(c0) - 1), p = t.test(b, c0)$p.value)
}))
table_s4a |> ktab(caption = "Table S4A, part 1. Adiposity phenotype of the 14 profiled pools (two-donor means; Welch's t-test)", digits = 1)
```

Table S4A, part 1. Adiposity phenotype of the 14 profiled pools
(two-donor means; Welch’s t-test)

| phenotype | control\_mean | bean\_mean | pct\_diff | p |
| --- | --- | --- | --- | --- |
| Body weight (g) | 49.6 | 43.9 | -11.5 | 0.0022 |
| Subcutaneous fat (mg) | 2331.3 | 1588.4 | -31.9 | 0.013 |
| Mesenteric fat (mg) | 1168.9 | 749.5 | -35.9 | 0.013 |
| Retroperitoneal fat (mg) | 495.9 | 529.9 | 6.9 | 0.37 |
| Epididymal fat (mg) | 1790.4 | 2167.4 | 21.1 | 0.023 |
| Total visceral fat (mg) | 3455.1 | 3446.9 | -0.2 | 0.97 |
| Total depot mass (mg) | 5786.4 | 5035.2 | -13.0 | 0.11 |
| Hepatic lipid (mg/g dry liver) | 415.6 | 125.9 | -69.7 | 9.5e-05 |

```
ve <- 100 * pca_dep$sdev^2 / sum(pca_dep$sdev^2)
table_s4a_pc <- bind_rows(lapply(1:4, function(k) {
  sc <- pca_dep$x[, k]; ld <- pca_dep$rotation[, k]
  tibble(component = paste0("PC", k), variance_pct = ve[k], loading_subcutaneous = ld[["SQ_FAT_mg"]], loading_mesenteric = ld[["MES_FAT_mg"]],
         loading_retroperitoneal = ld[["RP_FAT_mg"]], loading_epididymal = ld[["EP_PM_FAT_mg"]],
         control_mean_score = mean(sc[pool14$group == "Control"]), bean_mean_score = mean(sc[pool14$group == "Bean"]),
         p = t.test(sc[pool14$group == "Bean"], sc[pool14$group == "Control"])$p.value,
         r_body_weight = cor(sc, pool14$FINAL_BW_g), r_total_depot_mass = cor(sc, pool14$TOTAL_DEPOT_mg), r_hepatic_lipid = cor(sc, pool14$LIVER_LIPID))
}))
table_s4a_pc |> ktab(caption = "Table S4A, part 2. Principal components of the four z-scored depot masses (14 pools)", digits = 2)
```

Table S4A, part 2. Principal components of the four z-scored depot
masses (14 pools)

| component | variance\_pct | loading\_subcutaneous | loading\_mesenteric | loading\_retroperitoneal | loading\_epididymal | control\_mean\_score | bean\_mean\_score | p | r\_body\_weight | r\_total\_depot\_mass | r\_hepatic\_lipid |
| --- | --- | --- | --- | --- | --- | --- | --- | --- | --- | --- | --- |
| PC1 | 54.90 | 0.55 | 0.60 | -0.34 | -0.47 | 1.09 | -1.09 | 0.0015 | 0.83 | 0.60 | 0.91 |
| PC2 | 30.91 | 0.46 | 0.34 | 0.67 | 0.47 | 0.06 | -0.06 | 0.85 | 0.41 | 0.76 | 0.16 |
| PC3 | 10.72 | 0.17 | 0.05 | -0.66 | 0.73 | -0.12 | 0.12 | 0.52 | -0.09 | 0.24 | -0.22 |
| PC4 | 3.47 | -0.68 | 0.73 | 0.01 | 0.12 | -0.05 | 0.05 | 0.63 | 0.09 | -0.05 | -0.06 |

```
hotelling_t2 <- function(X, g) {
  X <- as.matrix(X); a <- X[g == "Control", , drop = FALSE]; b <- X[g == "Bean", , drop = FALSE]; n1 <- nrow(a); n2 <- nrow(b); k <- ncol(X)
  S <- ((n1 - 1) * cov(a) + (n2 - 1) * cov(b)) / (n1 + n2 - 2); d <- colMeans(b) - colMeans(a)
  T2 <- drop(n1 * n2 / (n1 + n2) * t(d) %*% solve(S, d)); Fv <- T2 * (n1 + n2 - k - 1) / (k * (n1 + n2 - 2))
  c(T2 = T2, F = Fv, df1 = k, df2 = n1 + n2 - k - 1, p = pf(Fv, k, n1 + n2 - k - 1, lower.tail = FALSE))
}
ht <- hotelling_t2(pool14[, dep_vars], pool14$group)
ht
```

```
##          T2           F         df1         df2           p 
## 21.38813350  4.01027503  4.00000000  9.00000000  0.03884009
```

As a four-depot profile the diets differed (Hotelling’s T² = 21.4,
F(4,9) = 4.01, p = 0.039). PC1 (55% of variance) contrasts subcutaneous
and mesenteric fat (loadings +0.55 and +0.60) with retroperitoneal and
epididymal fat (-0.34 and -0.47) and separates the diets (mean score
control +1.09, bean -1.09; p = 0.002). PC2 (31%) has positive loadings
on all four depots, correlates with total depot mass (r = 0.76), and
does not differ by diet (p = 0.847). PC1 correlates with hepatic lipid
(r = 0.91) and body weight (r = 0.83). Total depot mass was 13.0% lower
in the pools (p = 0.109) and 11.7% lower in all 40 animals (p =
0.023).

## 16.2 Multivariate analysis of covariance

For each of the six classes, the response is the vector of its three
compartment composites (cecal content, liver, plasma; p = 3; n = 14
pools; the BCAA class combines the cecal keto/hydroxy-acid, hepatic
catabolite, and plasma parent composites). Predictors are diet and, in
nine models, none or one or two z-scored covariates. The diet term has
one degree of freedom, so the Hotelling-Lawley trace, Pillai’s trace,
Wilks’ Λ, and Roy’s largest root give the same exact F with (p, n − q −
p + 1) degrees of freedom, where q is the number of model parameters.
Covariate terms are tested the same way. Each model is refitted with
each pool omitted in turn (depot components held at their full-sample
values), and the largest diet p-value is the leave-one-pool-out
sensitivity. Under each model the six class-level diet p-values are
BH-adjusted as one family.

```
mv_models <- list(M0 = character(0), M6 = "TOTAL_DEPOT_mg", M7 = "PC2", M1 = "PC1", M2 = c("PC1", "PC2"), M3 = "LIVER_LIPID",
                  M4 = c("PC1", "LIVER_LIPID"), M8 = c("TOTAL_DEPOT_mg", "LIVER_LIPID"), M5 = "SQ_FAT_mg")
cov_lab <- c(TOTAL_DEPOT_mg = "total depot mass", PC1 = "depot PC1", PC2 = "depot PC2", LIVER_LIPID = "hepatic lipid", SQ_FAT_mg = "subcutaneous fat")
model_lab <- sapply(mv_models, function(cv) if (length(cv) == 0) "Diet only" else paste("Diet +", paste(cov_lab[cv], collapse = " + ")))
mv_classes <- list(`Branched-chain amino acids` = c(CECAL = "BCAA ketoacid/hydroxy-acid", LIVER = "BCAA-catabolite", PLASMA = "BCAA-parent"),
                   `n-3 polyunsaturated fatty acids` = rep("n-3 PUFA", 3), `Glutathione-related` = rep("Glutathione cycle", 3),
                   Ceramides = rep("Ceramide", 3), `Bile acids` = rep("Bile acid", 3), `Ketone bodies / acylcarnitines` = rep("Ketone / FA-oxidation tone", 3))
mv_classes <- lapply(mv_classes, function(k) setNames(k, names(mats)))

design <- function(d, cv) {  # diet (bean = 1) and z-scored covariates
  X <- cbind(`(Intercept)` = 1, diet = as.numeric(d$group == "Bean"))
  if (length(cv)) X <- cbind(X, scale(as.matrix(d[, cv, drop = FALSE])))
  X
}
mv_term <- function(Y, X, term) {  # Hotelling-Lawley test of one 1-df term of the multivariate linear model
  Y <- as.matrix(Y); Xr <- X[, colnames(X) != term, drop = FALSE]
  E <- crossprod(qr.resid(qr(X), Y)); H <- crossprod(qr.resid(qr(Xr), Y)) - E
  hl <- sum(diag(H %*% solve(E))); p <- ncol(Y); df2 <- nrow(Y) - ncol(X) - p + 1
  c(HL = hl, F = hl * df2 / p, df1 = p, df2 = df2, p = pf(hl * df2 / p, p, df2, lower.tail = FALSE))
}
mv_fit <- function(Y, d, cv) {
  X <- design(d, cv); r <- mv_term(Y, X, "diet")
  loo <- max(sapply(seq_len(nrow(d)), function(i) mv_term(as.matrix(Y)[-i, , drop = FALSE], design(d[-i, ], cv), "diet")[["p"]]))
  cov_p <- sapply(cv, function(v) mv_term(Y, X, v)[["p"]])
  tibble(HL = r[["HL"]], F = r[["F"]], df = sprintf("(%d, %d)", r[["df1"]], r[["df2"]]), p = r[["p"]], p_loo_max = loo,
         covariate_p = if (length(cv)) paste(sprintf("%s %.3f", cov_lab[cv], cov_p), collapse = "; ") else "—")
}
ols_diet <- function(y, d, cv) {  # diet coefficient of a single composite under the same predictors
  X <- design(d, cv); co <- summary(lm(y ~ X - 1))$coefficients
  co[c("Xdiet", if (length(cv)) paste0("X", cv)), , drop = FALSE]
}
class_Y <- function(cl) {  # 14 x 3 matrix of the class composites, rows in pool14 order
  sapply(names(mats), function(m) { s <- scores |> filter(matrix == m, composite == mv_classes[[cl]][[m]]) |> arrange(POOL)
    stopifnot(length(s$POOL) == nrow(pool14), all(s$POOL == pool14$POOL)); s$score })
}
vif_design <- function(d, cv) { X <- design(d, cv)[, -1, drop = FALSE]
  if (ncol(X) == 1) return(c(diet = 1)); sapply(colnames(X), function(j) 1 / (1 - summary(lm(X[, j] ~ X[, colnames(X) != j]))$r.squared)) }
```

```
table_s4b <- bind_rows(lapply(names(mv_classes), function(cl) bind_rows(lapply(names(mv_models), function(mo)
  mv_fit(class_Y(cl), pool14, mv_models[[mo]]) |> mutate(class = cl, model = mo, predictors = model_lab[[mo]], .before = 1))))) |>
  group_by(model) |> mutate(q = p.adjust(p, "BH")) |> ungroup()
table_s4b |> ktab(caption = "Table S4B. Class-level MANCOVA: diet term for each class (response, the class composite in cecal content, liver, and plasma) under each model; q, BH across the six classes within a model", digits = 2)
```

Table S4B. Class-level MANCOVA: diet term for each class (response, the
class composite in cecal content, liver, and plasma) under each model;
q, BH across the six classes within a model

| class | model | predictors | HL | F | df | p | p\_loo\_max | covariate\_p | q |
| --- | --- | --- | --- | --- | --- | --- | --- | --- | --- |
| Branched-chain amino acids | M0 | Diet only | 20.85 | 69.49 | (3, 10) | 5.3e-07 | 3.4e-06 | — | 3.2e-06 |
| Branched-chain amino acids | M6 | Diet + total depot mass | 28.45 | 85.35 | (3, 9) | 6.2e-07 | 4.2e-06 | total depot mass 0.147 | 3.7e-06 |
| Branched-chain amino acids | M7 | Diet + depot PC2 | 36.88 | 110.63 | (3, 9) | 2e-07 | 1.6e-06 | depot PC2 0.056 | 1.2e-06 |
| Branched-chain amino acids | M1 | Diet + depot PC1 | 8.62 | 25.85 | (3, 9) | 9.3e-05 | 0.0022 | depot PC1 0.070 | 0.00056 |
| Branched-chain amino acids | M2 | Diet + depot PC1 + depot PC2 | 14.97 | 39.93 | (3, 8) | 3.7e-05 | 0.00086 | depot PC1 0.095; depot PC2 0.078 | 0.00022 |
| Branched-chain amino acids | M3 | Diet + hepatic lipid | 4.90 | 14.71 | (3, 9) | 0.00081 | 0.0058 | hepatic lipid 0.061 | 0.0049 |
| Branched-chain amino acids | M4 | Diet + depot PC1 + hepatic lipid | 4.79 | 12.77 | (3, 8) | 0.002 | 0.011 | depot PC1 0.193; hepatic lipid 0.173 | 0.012 |
| Branched-chain amino acids | M8 | Diet + total depot mass + hepatic lipid | 6.79 | 18.09 | (3, 8) | 0.00063 | 0.0081 | total depot mass 0.147; hepatic lipid 0.067 | 0.0038 |
| Branched-chain amino acids | M5 | Diet + subcutaneous fat | 14.07 | 42.21 | (3, 9) | 1.3e-05 | 0.0015 | subcutaneous fat 0.484 | 7.5e-05 |
| n-3 polyunsaturated fatty acids | M0 | Diet only | 7.17 | 23.91 | (3, 10) | 7e-05 | 0.00027 | — | 0.00014 |
| n-3 polyunsaturated fatty acids | M6 | Diet + total depot mass | 5.49 | 16.47 | (3, 9) | 0.00054 | 0.002 | total depot mass 0.523 | 0.0011 |
| n-3 polyunsaturated fatty acids | M7 | Diet + depot PC2 | 7.18 | 21.54 | (3, 9) | 0.00019 | 0.00077 | depot PC2 0.542 | 0.00038 |
| n-3 polyunsaturated fatty acids | M1 | Diet + depot PC1 | 2.43 | 7.28 | (3, 9) | 0.0088 | 0.016 | depot PC1 0.362 | 0.013 |
| n-3 polyunsaturated fatty acids | M2 | Diet + depot PC1 + depot PC2 | 2.41 | 6.44 | (3, 8) | 0.016 | 0.028 | depot PC1 0.418; depot PC2 0.594 | 0.024 |
| n-3 polyunsaturated fatty acids | M3 | Diet + hepatic lipid | 1.26 | 3.77 | (3, 9) | 0.053 | 0.28 | hepatic lipid 0.959 | 0.082 |
| n-3 polyunsaturated fatty acids | M4 | Diet + depot PC1 + hepatic lipid | 2.05 | 5.47 | (3, 8) | 0.024 | 0.067 | depot PC1 0.194; hepatic lipid 0.545 | 0.049 |
| n-3 polyunsaturated fatty acids | M8 | Diet + total depot mass + hepatic lipid | 1.44 | 3.84 | (3, 8) | 0.057 | 0.38 | total depot mass 0.552; hepatic lipid 0.947 | 0.085 |
| n-3 polyunsaturated fatty acids | M5 | Diet + subcutaneous fat | 3.65 | 10.96 | (3, 9) | 0.0023 | 0.0066 | subcutaneous fat 0.228 | 0.0035 |
| Glutathione-related | M0 | Diet only | 10.04 | 33.47 | (3, 10) | 1.6e-05 | 7.2e-05 | — | 4.8e-05 |
| Glutathione-related | M6 | Diet + total depot mass | 12.81 | 38.43 | (3, 9) | 1.9e-05 | 0.0001 | total depot mass 0.019 | 5.6e-05 |
| Glutathione-related | M7 | Diet + depot PC2 | 11.71 | 35.12 | (3, 9) | 2.7e-05 | 0.00013 | depot PC2 0.272 | 8.1e-05 |
| Glutathione-related | M1 | Diet + depot PC1 | 3.64 | 10.93 | (3, 9) | 0.0023 | 0.0091 | depot PC1 0.412 | 0.007 |
| Glutathione-related | M2 | Diet + depot PC1 + depot PC2 | 4.44 | 11.84 | (3, 8) | 0.0026 | 0.01 | depot PC1 0.361; depot PC2 0.248 | 0.0052 |
| Glutathione-related | M3 | Diet + hepatic lipid | 1.76 | 5.29 | (3, 9) | 0.022 | 0.14 | hepatic lipid 0.915 | 0.067 |
| Glutathione-related | M4 | Diet + depot PC1 + hepatic lipid | 2.43 | 6.47 | (3, 8) | 0.016 | 0.1 | depot PC1 0.382; hepatic lipid 0.811 | 0.047 |
| Glutathione-related | M8 | Diet + total depot mass + hepatic lipid | 5.12 | 13.65 | (3, 8) | 0.0016 | 0.021 | total depot mass 0.018; hepatic lipid 0.639 | 0.0049 |
| Glutathione-related | M5 | Diet + subcutaneous fat | 7.00 | 21.01 | (3, 9) | 0.00021 | 0.0047 | subcutaneous fat 0.027 | 0.00063 |
| Ceramides | M0 | Diet only | 5.54 | 18.46 | (3, 10) | 0.00021 | 0.00074 | — | 0.00032 |
| Ceramides | M6 | Diet + total depot mass | 4.67 | 14.00 | (3, 9) | 0.00097 | 0.0035 | total depot mass 0.101 | 0.0012 |
| Ceramides | M7 | Diet + depot PC2 | 6.02 | 18.05 | (3, 9) | 0.00038 | 0.0013 | depot PC2 0.517 | 0.00057 |
| Ceramides | M1 | Diet + depot PC1 | 1.83 | 5.50 | (3, 9) | 0.02 | 0.073 | depot PC1 0.528 | 0.024 |
| Ceramides | M2 | Diet + depot PC1 + depot PC2 | 1.95 | 5.20 | (3, 8) | 0.028 | 0.088 | depot PC1 0.382; depot PC2 0.374 | 0.033 |
| Ceramides | M3 | Diet + hepatic lipid | 0.74 | 2.21 | (3, 9) | 0.16 | 0.62 | hepatic lipid 0.590 | 0.16 |
| Ceramides | M4 | Diet + depot PC1 + hepatic lipid | 0.72 | 1.93 | (3, 8) | 0.2 | 0.66 | depot PC1 0.915; hepatic lipid 0.970 | 0.2 |
| Ceramides | M8 | Diet + total depot mass + hepatic lipid | 0.99 | 2.63 | (3, 8) | 0.12 | 0.64 | total depot mass 0.254; hepatic lipid 0.975 | 0.13 |
| Ceramides | M5 | Diet + subcutaneous fat | 3.07 | 9.20 | (3, 9) | 0.0042 | 0.1 | subcutaneous fat 0.082 | 0.005 |
| Bile acids | M0 | Diet only | 1.21 | 4.04 | (3, 10) | 0.04 | 0.081 | — | 0.04 |
| Bile acids | M6 | Diet + total depot mass | 0.84 | 2.53 | (3, 9) | 0.12 | 0.26 | total depot mass 0.190 | 0.12 |
| Bile acids | M7 | Diet + depot PC2 | 1.22 | 3.65 | (3, 9) | 0.057 | 0.11 | depot PC2 0.845 | 0.057 |
| Bile acids | M1 | Diet + depot PC1 | 1.00 | 2.99 | (3, 9) | 0.088 | 0.29 | depot PC1 0.062 | 0.088 |
| Bile acids | M2 | Diet + depot PC1 + depot PC2 | 1.01 | 2.69 | (3, 8) | 0.12 | 0.35 | depot PC1 0.072; depot PC2 0.756 | 0.12 |
| Bile acids | M3 | Diet + hepatic lipid | 1.06 | 3.17 | (3, 9) | 0.078 | 0.29 | hepatic lipid 0.091 | 0.093 |
| Bile acids | M4 | Diet + depot PC1 + hepatic lipid | 0.97 | 2.58 | (3, 8) | 0.13 | 0.48 | depot PC1 0.613; hepatic lipid 0.792 | 0.15 |
| Bile acids | M8 | Diet + total depot mass + hepatic lipid | 0.97 | 2.59 | (3, 8) | 0.13 | 0.41 | total depot mass 0.508; hepatic lipid 0.278 | 0.13 |
| Bile acids | M5 | Diet + subcutaneous fat | 0.87 | 2.61 | (3, 9) | 0.12 | 0.29 | subcutaneous fat 0.143 | 0.12 |
| Ketone bodies / acylcarnitines | M0 | Diet only | 1.51 | 5.05 | (3, 10) | 0.022 | 0.045 | — | 0.026 |
| Ketone bodies / acylcarnitines | M6 | Diet + total depot mass | 4.88 | 14.64 | (3, 9) | 0.00083 | 0.012 | total depot mass 0.015 | 0.0012 |
| Ketone bodies / acylcarnitines | M7 | Diet + depot PC2 | 1.88 | 5.65 | (3, 9) | 0.019 | 0.044 | depot PC2 0.185 | 0.022 |
| Ketone bodies / acylcarnitines | M1 | Diet + depot PC1 | 3.02 | 9.05 | (3, 9) | 0.0044 | 0.063 | depot PC1 0.134 | 0.0088 |
| Ketone bodies / acylcarnitines | M2 | Diet + depot PC1 + depot PC2 | 4.56 | 12.15 | (3, 8) | 0.0024 | 0.079 | depot PC1 0.084; depot PC2 0.112 | 0.0052 |
| Ketone bodies / acylcarnitines | M3 | Diet + hepatic lipid | 1.24 | 3.73 | (3, 9) | 0.054 | 0.22 | hepatic lipid 0.486 | 0.082 |
| Ketone bodies / acylcarnitines | M4 | Diet + depot PC1 + hepatic lipid | 1.27 | 3.40 | (3, 8) | 0.074 | 0.23 | depot PC1 0.185; hepatic lipid 0.569 | 0.11 |
| Ketone bodies / acylcarnitines | M8 | Diet + total depot mass + hepatic lipid | 1.54 | 4.10 | (3, 8) | 0.049 | 0.22 | total depot mass 0.048; hepatic lipid 0.877 | 0.085 |
| Ketone bodies / acylcarnitines | M5 | Diet + subcutaneous fat | 5.88 | 17.65 | (3, 9) | 0.00041 | 0.018 | subcutaneous fat 0.016 | 0.00082 |

```
t1_models <- c("M0", "M6", "M1", "M4", "M8")
fmt_sci <- function(p) sapply(p, function(x) if (x < 0.01) sub("e-0?", " × 10^-", formatC(x, format = "e", digits = 1)) else if (x < 0.095) sprintf("%.3f", x) else sprintf("%.2f", x))
table1 <- table_s4b |> filter(model %in% t1_models) |>
  mutate(cell = sprintf("%.1f; %s [%s]%s", F, fmt_sci(p), fmt_sci(p_loo_max), ifelse(model == "M4", paste0("; q = ", fmt_sci(q)), "")),
         model = factor(paste0(model, ": ", model_lab[as.character(model)]), paste0(t1_models, ": ", model_lab[t1_models]))) |>
  select(class, model, cell) |> pivot_wider(names_from = model, values_from = cell) |>
  mutate(class = factor(class, c("Branched-chain amino acids", "Glutathione-related", "n-3 polyunsaturated fatty acids",
                                 "Ketone bodies / acylcarnitines", "Ceramides", "Bile acids"))) |> arrange(class)
table1 |> ktab(caption = "Table 1. Class-level MANCOVA: F; p [leave-one-pool-out maximum p] for the diet term, unadjusted and adjusted for adiposity (n = 14 pools; q, BH across the six classes)")
```

Table 1. Class-level MANCOVA: F; p [leave-one-pool-out maximum p] for
the diet term, unadjusted and adjusted for adiposity (n = 14 pools; q,
BH across the six classes)

| class | M0: Diet only | M6: Diet + total depot mass | M1: Diet + depot PC1 | M4: Diet + depot PC1 + hepatic lipid | M8: Diet + total depot mass + hepatic lipid |
| --- | --- | --- | --- | --- | --- |
| Branched-chain amino acids | 69.5; 5.3 × 10^-7 [3.4 × 10^-6] | 85.4; 6.2 × 10^-7 [4.2 × 10^-6] | 25.8; 9.3 × 10^-5 [2.2 × 10^-3] | 12.8; 2.0 × 10^-3 [0.011]; q = 0.012 | 18.1; 6.3 × 10^-4 [8.1 × 10^-3] |
| Glutathione-related | 33.5; 1.6 × 10^-5 [7.2 × 10^-5] | 38.4; 1.9 × 10^-5 [1.0 × 10^-4] | 10.9; 2.3 × 10^-3 [9.1 × 10^-3] | 6.5; 0.016 [0.10]; q = 0.047 | 13.7; 1.6 × 10^-3 [0.021] |
| n-3 polyunsaturated fatty acids | 23.9; 7.0 × 10^-5 [2.7 × 10^-4] | 16.5; 5.4 × 10^-4 [2.0 × 10^-3] | 7.3; 8.8 × 10^-3 [0.016] | 5.5; 0.024 [0.067]; q = 0.049 | 3.8; 0.057 [0.38] |
| Ketone bodies / acylcarnitines | 5.0; 0.022 [0.045] | 14.6; 8.3 × 10^-4 [0.012] | 9.1; 4.4 × 10^-3 [0.063] | 3.4; 0.074 [0.23]; q = 0.11 | 4.1; 0.049 [0.22] |
| Ceramides | 18.5; 2.1 × 10^-4 [7.4 × 10^-4] | 14.0; 9.7 × 10^-4 [3.5 × 10^-3] | 5.5; 0.020 [0.073] | 1.9; 0.20 [0.66]; q = 0.20 | 2.6; 0.12 [0.64] |
| Bile acids | 4.0; 0.040 [0.081] | 2.5; 0.12 [0.26] | 3.0; 0.088 [0.29] | 2.6; 0.13 [0.48]; q = 0.15 | 2.6; 0.13 [0.41] |

```
m4 <- table_s4b |> filter(model == "M4"); m8 <- table_s4b |> filter(model == "M8")
vif_tab <- bind_rows(lapply(names(mv_models)[-1], function(mo) { v <- vif_design(pool14, mv_models[[mo]])
  tibble(model = mo, predictors = model_lab[[mo]], VIF = paste(sprintf("%s %.1f", c("diet", cov_lab[mv_models[[mo]]]), v), collapse = "; "), max_VIF = max(v)) }))
vif_tab |> ktab(caption = "Variance inflation factors of the class-level designs (14 pools)", digits = 1)
```

Variance inflation factors of the class-level designs (14 pools)

| model | predictors | VIF | max\_VIF |
| --- | --- | --- | --- |
| M6 | Diet + total depot mass | diet 1.3; total depot mass 1.3 | 1.3 |
| M7 | Diet + depot PC2 | diet 1.0; depot PC2 1.0 | 1.0 |
| M1 | Diet + depot PC1 | diet 2.4; depot PC1 2.4 | 2.4 |
| M2 | Diet + depot PC1 + depot PC2 | diet 2.4; depot PC1 2.4; depot PC2 1.0 | 2.4 |
| M3 | Diet + hepatic lipid | diet 4.6; hepatic lipid 4.6 | 4.6 |
| M4 | Diet + depot PC1 + hepatic lipid | diet 4.8; depot PC1 6.0; hepatic lipid 11.6 | 11.6 |
| M8 | Diet + total depot mass + hepatic lipid | diet 4.8; total depot mass 1.7; hepatic lipid 6.1 | 6.1 |
| M5 | Diet + subcutaneous fat | diet 1.8; subcutaneous fat 1.8 | 1.8 |

```
vif4 <- vif_design(pool14, mv_models$M4); vif8 <- vif_design(pool14, mv_models$M8)
```

The hand-coded Hotelling-Lawley test is checked against
`stats::anova.mlm` (full vs. reduced multivariate linear
model) for every class and model.

```
chk <- bind_rows(lapply(names(mv_classes), function(cl) bind_rows(lapply(names(mv_models), function(mo) {
  Y <- class_Y(cl); X <- design(pool14, mv_models[[mo]]); Xr <- X[, colnames(X) != "diet", drop = FALSE]
  a <- anova(lm(Y ~ X - 1), lm(Y ~ Xr - 1), test = "Hotelling-Lawley")
  tibble(class = cl, model = mo, p_anova_mlm = a$`Pr(>F)`[2])
})))) |> left_join(table_s4b |> select(class, model, p), by = c("class", "model"))
c(max_abs_diff_p = max(abs(chk$p - chk$p_anova_mlm)), cor_PC1_hepatic_lipid = cor(pool14$PC1, pool14$LIVER_LIPID))
```

```
##        max_abs_diff_p cor_PC1_hepatic_lipid 
##          4.718448e-16          9.092913e-01
```

The compartment-level analysis uses the six composites of one
compartment as the response (p = 6) with the same predictor sets. Each
sample is linked to the phenotype of its own donors, so the depot
components are refitted on the donor-linked depot masses of that
compartment; for cecal content and liver these are the pool values
above.

```
comp_data <- lapply(setNames(names(mats), names(mats)), function(m) {
  w <- scores |> filter(matrix == m) |> select(sample, POOL, group, composite, score) |> pivot_wider(names_from = composite, values_from = score) |>
    left_join(sample_ph |> filter(matrix == m) |> select(sample, all_of(c(unname(dep_vars), "TOTAL_DEPOT_mg", "LIVER_LIPID"))), by = "sample") |>
    arrange(POOL)
  pc <- depot_pca(w); w$PC1 <- pc$x[, 1]; w$PC2 <- pc$x[, 2]; w
})
comp_names <- function(m) unname(sapply(mv_classes, function(k) k[[m]]))
table_s4c <- bind_rows(lapply(names(mats), function(m) bind_rows(lapply(names(mv_models), function(mo)
  mv_fit(as.matrix(comp_data[[m]][, comp_names(m)]), comp_data[[m]], mv_models[[mo]]) |>
    mutate(compartment = mats[[m]], model = mo, predictors = model_lab[[mo]], .before = 1)))))
table_s4c |> ktab(caption = "Table S4C. Compartment-level MANCOVA (response, the six composites of one compartment; donor-linked phenotypes): diet term under each model", digits = 2)
```

Table S4C. Compartment-level MANCOVA (response, the six composites of
one compartment; donor-linked phenotypes): diet term under each model

| compartment | model | predictors | HL | F | df | p | p\_loo\_max | covariate\_p |
| --- | --- | --- | --- | --- | --- | --- | --- | --- |
| Cecal content | M0 | Diet only | 18.32 | 21.38 | (6, 7) | 0.00036 | 0.0016 | — |
| Cecal content | M6 | Diet + total depot mass | 14.57 | 14.57 | (6, 6) | 0.0024 | 0.015 | total depot mass 0.043 |
| Cecal content | M7 | Diet + depot PC2 | 18.39 | 18.39 | (6, 6) | 0.0013 | 0.0052 | depot PC2 0.134 |
| Cecal content | M1 | Diet + depot PC1 | 7.42 | 7.42 | (6, 6) | 0.014 | 0.07 | depot PC1 0.812 |
| Cecal content | M2 | Diet + depot PC1 + depot PC2 | 7.50 | 6.25 | (6, 5) | 0.031 | 0.23 | depot PC1 0.817; depot PC2 0.185 |
| Cecal content | M3 | Diet + hepatic lipid | 3.84 | 3.84 | (6, 6) | 0.063 | 0.23 | hepatic lipid 0.374 |
| Cecal content | M4 | Diet + depot PC1 + hepatic lipid | 3.85 | 3.21 | (6, 5) | 0.11 | 0.33 | depot PC1 0.534; hepatic lipid 0.235 |
| Cecal content | M8 | Diet + total depot mass + hepatic lipid | 3.68 | 3.07 | (6, 5) | 0.12 | 0.44 | total depot mass 0.035; hepatic lipid 0.239 |
| Cecal content | M5 | Diet + subcutaneous fat | 10.57 | 10.57 | (6, 6) | 0.0056 | 0.13 | subcutaneous fat 0.215 |
| Liver | M0 | Diet only | 27.60 | 32.19 | (6, 7) | 9.4e-05 | 0.00051 | — |
| Liver | M6 | Diet + total depot mass | 23.85 | 23.85 | (6, 6) | 0.00061 | 0.0031 | total depot mass 0.071 |
| Liver | M7 | Diet + depot PC2 | 33.59 | 33.59 | (6, 6) | 0.00023 | 0.0013 | depot PC2 0.648 |
| Liver | M1 | Diet + depot PC1 | 31.72 | 31.72 | (6, 6) | 0.00027 | 0.0022 | depot PC1 0.041 |
| Liver | M2 | Diet + depot PC1 + depot PC2 | 32.06 | 26.71 | (6, 5) | 0.0012 | 0.0082 | depot PC1 0.041; depot PC2 0.481 |
| Liver | M3 | Diet + hepatic lipid | 9.87 | 9.87 | (6, 6) | 0.0068 | 0.04 | hepatic lipid 0.336 |
| Liver | M4 | Diet + depot PC1 + hepatic lipid | 15.84 | 13.20 | (6, 5) | 0.0062 | 0.037 | depot PC1 0.333; hepatic lipid 0.974 |
| Liver | M8 | Diet + total depot mass + hepatic lipid | 13.39 | 11.16 | (6, 5) | 0.0091 | 0.042 | total depot mass 0.133; hepatic lipid 0.460 |
| Liver | M5 | Diet + subcutaneous fat | 15.09 | 15.09 | (6, 6) | 0.0022 | 0.011 | subcutaneous fat 0.012 |
| Plasma | M0 | Diet only | 15.95 | 18.60 | (6, 7) | 0.00056 | 0.0023 | — |
| Plasma | M6 | Diet + total depot mass | 19.62 | 19.62 | (6, 6) | 0.0011 | 0.0048 | total depot mass 0.069 |
| Plasma | M7 | Diet + depot PC2 | 15.86 | 15.86 | (6, 6) | 0.0019 | 0.0077 | depot PC2 0.040 |
| Plasma | M1 | Diet + depot PC1 | 7.00 | 7.00 | (6, 6) | 0.016 | 0.049 | depot PC1 0.721 |
| Plasma | M2 | Diet + depot PC1 + depot PC2 | 6.90 | 5.75 | (6, 5) | 0.037 | 0.094 | depot PC1 0.781; depot PC2 0.076 |
| Plasma | M3 | Diet + hepatic lipid | 5.34 | 5.34 | (6, 6) | 0.031 | 0.11 | hepatic lipid 0.877 |
| Plasma | M4 | Diet + depot PC1 + hepatic lipid | 51.17 | 42.64 | (6, 5) | 0.00039 | 0.0069 | depot PC1 0.013; hepatic lipid 0.018 |
| Plasma | M8 | Diet + total depot mass + hepatic lipid | 9.24 | 7.70 | (6, 5) | 0.02 | 0.086 | total depot mass 0.128; hepatic lipid 0.930 |
| Plasma | M5 | Diet + subcutaneous fat | 10.77 | 10.77 | (6, 6) | 0.0054 | 0.03 | subcutaneous fat 0.342 |

```
coef_loo <- function(y, d, cv) {
  co <- ols_diet(y, d, cv)
  loo <- max(sapply(seq_len(nrow(d)), function(i) ols_diet(y[-i], d[-i, ], cv)["Xdiet", 4]))
  tibble(b_diet = co["Xdiet", 1], se = co["Xdiet", 2], p = co["Xdiet", 4], p_loo_max = loo,
         p_covariate_1 = if (length(cv) >= 1) co[paste0("X", cv[1]), 4] else NA_real_, p_covariate_2 = if (length(cv) == 2) co[paste0("X", cv[2]), 4] else NA_real_)
}
table_s4d <- bind_rows(lapply(names(mv_classes), function(cl) { Y <- class_Y(cl); bind_rows(lapply(names(mv_models), function(mo)
  bind_rows(lapply(names(mats), function(m) coef_loo(Y[, m], pool14, mv_models[[mo]]) |>
    mutate(class = cl, model = mo, compartment = mats[[m]], composite = mv_classes[[cl]][[m]], .before = 1))))) }))
table_s4d |> select(-p_covariate_1, -p_covariate_2) |>
  ktab(caption = "Table S4D. Per-compartment diet coefficient for each class under each class-level model (OLS on the compartment composite; two-donor pool phenotype)", digits = 2)
```

Table S4D. Per-compartment diet coefficient for each class under each
class-level model (OLS on the compartment composite; two-donor pool
phenotype)

| class | model | compartment | composite | b\_diet | se | p | p\_loo\_max |
| --- | --- | --- | --- | --- | --- | --- | --- |
| Branched-chain amino acids | M0 | Cecal content | BCAA ketoacid/hydroxy-acid | 2.05 | 0.16 | 2.7e-08 | 1.7e-07 |
| Branched-chain amino acids | M0 | Liver | BCAA-catabolite | 1.30 | 0.13 | 4.1e-07 | 2e-06 |
| Branched-chain amino acids | M0 | Plasma | BCAA-parent | -0.57 | 0.25 | 0.037 | 0.074 |
| Branched-chain amino acids | M6 | Cecal content | BCAA ketoacid/hydroxy-acid | 2.11 | 0.19 | 2.2e-07 | 1.9e-06 |
| Branched-chain amino acids | M6 | Liver | BCAA-catabolite | 1.39 | 0.14 | 9.2e-07 | 2.9e-05 |
| Branched-chain amino acids | M6 | Plasma | BCAA-parent | -0.72 | 0.27 | 0.024 | 0.055 |
| Branched-chain amino acids | M7 | Cecal content | BCAA ketoacid/hydroxy-acid | 2.06 | 0.16 | 6.8e-08 | 4.3e-07 |
| Branched-chain amino acids | M7 | Liver | BCAA-catabolite | 1.30 | 0.14 | 1.1e-06 | 6.4e-06 |
| Branched-chain amino acids | M7 | Plasma | BCAA-parent | -0.60 | 0.22 | 0.018 | 0.044 |
| Branched-chain amino acids | M1 | Cecal content | BCAA ketoacid/hydroxy-acid | 1.81 | 0.24 | 1.3e-05 | 0.00043 |
| Branched-chain amino acids | M1 | Liver | BCAA-catabolite | 1.54 | 0.19 | 5.8e-06 | 0.00012 |
| Branched-chain amino acids | M1 | Plasma | BCAA-parent | -0.39 | 0.39 | 0.34 | 0.9 |
| Branched-chain amino acids | M2 | Cecal content | BCAA ketoacid/hydroxy-acid | 1.83 | 0.25 | 2.3e-05 | 0.0011 |
| Branched-chain amino acids | M2 | Liver | BCAA-catabolite | 1.55 | 0.20 | 1.3e-05 | 0.0003 |
| Branched-chain amino acids | M2 | Plasma | BCAA-parent | -0.45 | 0.34 | 0.22 | 0.43 |
| Branched-chain amino acids | M3 | Cecal content | BCAA ketoacid/hydroxy-acid | 1.53 | 0.32 | 0.00052 | 0.0034 |
| Branched-chain amino acids | M3 | Liver | BCAA-catabolite | 1.51 | 0.29 | 0.00026 | 0.002 |
| Branched-chain amino acids | M3 | Plasma | BCAA-parent | -0.97 | 0.53 | 0.096 | 0.27 |
| Branched-chain amino acids | M4 | Cecal content | BCAA ketoacid/hydroxy-acid | 1.52 | 0.34 | 0.0012 | 0.0062 |
| Branched-chain amino acids | M4 | Liver | BCAA-catabolite | 1.42 | 0.28 | 0.00045 | 0.0026 |
| Branched-chain amino acids | M4 | Plasma | BCAA-parent | -1.20 | 0.45 | 0.023 | 0.11 |
| Branched-chain amino acids | M8 | Cecal content | BCAA ketoacid/hydroxy-acid | 1.39 | 0.27 | 0.0005 | 0.01 |
| Branched-chain amino acids | M8 | Liver | BCAA-catabolite | 1.44 | 0.29 | 0.00058 | 0.0067 |
| Branched-chain amino acids | M8 | Plasma | BCAA-parent | -0.87 | 0.56 | 0.15 | 0.38 |
| Branched-chain amino acids | M5 | Cecal content | BCAA ketoacid/hydroxy-acid | 2.02 | 0.23 | 2.3e-06 | 0.00046 |
| Branched-chain amino acids | M5 | Liver | BCAA-catabolite | 1.41 | 0.18 | 7e-06 | 0.0021 |
| Branched-chain amino acids | M5 | Plasma | BCAA-parent | -0.76 | 0.33 | 0.045 | 0.12 |
| n-3 polyunsaturated fatty acids | M0 | Cecal content | n-3 PUFA | 1.28 | 0.54 | 0.036 | 0.075 |
| n-3 polyunsaturated fatty acids | M0 | Liver | n-3 PUFA | 1.45 | 0.26 | 0.00011 | 0.00033 |
| n-3 polyunsaturated fatty acids | M0 | Plasma | n-3 PUFA | 1.68 | 0.19 | 1.6e-06 | 6.8e-06 |
| n-3 polyunsaturated fatty acids | M6 | Cecal content | n-3 PUFA | 1.34 | 0.64 | 0.059 | 0.18 |
| n-3 polyunsaturated fatty acids | M6 | Liver | n-3 PUFA | 1.23 | 0.27 | 0.00075 | 0.0023 |
| n-3 polyunsaturated fatty acids | M6 | Plasma | n-3 PUFA | 1.61 | 0.22 | 1.7e-05 | 0.00013 |
| n-3 polyunsaturated fatty acids | M7 | Cecal content | n-3 PUFA | 1.31 | 0.54 | 0.034 | 0.078 |
| n-3 polyunsaturated fatty acids | M7 | Liver | n-3 PUFA | 1.43 | 0.25 | 0.00015 | 0.00049 |
| n-3 polyunsaturated fatty acids | M7 | Plasma | n-3 PUFA | 1.68 | 0.20 | 4.3e-06 | 2e-05 |
| n-3 polyunsaturated fatty acids | M1 | Cecal content | n-3 PUFA | 0.46 | 0.81 | 0.58 | 0.82 |
| n-3 polyunsaturated fatty acids | M1 | Liver | n-3 PUFA | 1.13 | 0.39 | 0.015 | 0.076 |
| n-3 polyunsaturated fatty acids | M1 | Plasma | n-3 PUFA | 1.45 | 0.30 | 0.00049 | 0.003 |
| n-3 polyunsaturated fatty acids | M2 | Cecal content | n-3 PUFA | 0.53 | 0.82 | 0.53 | 0.83 |
| n-3 polyunsaturated fatty acids | M2 | Liver | n-3 PUFA | 1.09 | 0.39 | 0.019 | 0.08 |
| n-3 polyunsaturated fatty acids | M2 | Plasma | n-3 PUFA | 1.46 | 0.31 | 0.00092 | 0.0047 |
| n-3 polyunsaturated fatty acids | M3 | Cecal content | n-3 PUFA | 0.97 | 1.21 | 0.44 | 0.98 |
| n-3 polyunsaturated fatty acids | M3 | Liver | n-3 PUFA | 1.25 | 0.57 | 0.05 | 0.29 |
| n-3 polyunsaturated fatty acids | M3 | Plasma | n-3 PUFA | 1.51 | 0.43 | 0.0047 | 0.044 |
| n-3 polyunsaturated fatty acids | M4 | Cecal content | n-3 PUFA | 1.40 | 1.13 | 0.25 | 0.73 |
| n-3 polyunsaturated fatty acids | M4 | Liver | n-3 PUFA | 1.39 | 0.57 | 0.036 | 0.21 |
| n-3 polyunsaturated fatty acids | M4 | Plasma | n-3 PUFA | 1.60 | 0.44 | 0.0044 | 0.036 |
| n-3 polyunsaturated fatty acids | M8 | Cecal content | n-3 PUFA | 0.86 | 1.29 | 0.52 | 0.68 |
| n-3 polyunsaturated fatty acids | M8 | Liver | n-3 PUFA | 1.45 | 0.54 | 0.023 | 0.17 |
| n-3 polyunsaturated fatty acids | M8 | Plasma | n-3 PUFA | 1.57 | 0.45 | 0.0062 | 0.082 |
| n-3 polyunsaturated fatty acids | M5 | Cecal content | n-3 PUFA | 1.32 | 0.76 | 0.11 | 0.52 |
| n-3 polyunsaturated fatty acids | M5 | Liver | n-3 PUFA | 0.98 | 0.29 | 0.0059 | 0.023 |
| n-3 polyunsaturated fatty acids | M5 | Plasma | n-3 PUFA | 1.47 | 0.25 | 0.00012 | 0.0084 |
| Glutathione-related | M0 | Cecal content | Glutathione cycle | -0.55 | 0.35 | 0.15 | 0.29 |
| Glutathione-related | M0 | Liver | Glutathione cycle | 0.95 | 0.12 | 5e-06 | 2e-05 |
| Glutathione-related | M0 | Plasma | Glutathione cycle | 0.65 | 0.18 | 0.0039 | 0.0094 |
| Glutathione-related | M6 | Cecal content | Glutathione cycle | -0.68 | 0.41 | 0.12 | 0.42 |
| Glutathione-related | M6 | Liver | Glutathione cycle | 0.77 | 0.09 | 2.4e-06 | 1.3e-05 |
| Glutathione-related | M6 | Plasma | Glutathione cycle | 0.68 | 0.21 | 0.0087 | 0.024 |
| Glutathione-related | M7 | Cecal content | Glutathione cycle | -0.57 | 0.34 | 0.12 | 0.24 |
| Glutathione-related | M7 | Liver | Glutathione cycle | 0.93 | 0.11 | 2.5e-06 | 1.3e-05 |
| Glutathione-related | M7 | Plasma | Glutathione cycle | 0.65 | 0.19 | 0.0059 | 0.014 |
| Glutathione-related | M1 | Cecal content | Glutathione cycle | -0.24 | 0.56 | 0.67 | 0.98 |
| Glutathione-related | M1 | Liver | Glutathione cycle | 0.78 | 0.19 | 0.0014 | 0.007 |
| Glutathione-related | M1 | Plasma | Glutathione cycle | 0.73 | 0.29 | 0.029 | 0.092 |
| Glutathione-related | M2 | Cecal content | Glutathione cycle | -0.30 | 0.55 | 0.59 | 0.88 |
| Glutathione-related | M2 | Liver | Glutathione cycle | 0.75 | 0.15 | 0.00063 | 0.0022 |
| Glutathione-related | M2 | Plasma | Glutathione cycle | 0.73 | 0.31 | 0.038 | 0.12 |
| Glutathione-related | M3 | Cecal content | Glutathione cycle | -0.41 | 0.79 | 0.62 | 0.91 |
| Glutathione-related | M3 | Liver | Glutathione cycle | 0.81 | 0.27 | 0.012 | 0.11 |
| Glutathione-related | M3 | Plasma | Glutathione cycle | 0.67 | 0.41 | 0.13 | 0.31 |
| Glutathione-related | M4 | Cecal content | Glutathione cycle | -0.56 | 0.82 | 0.51 | 0.97 |
| Glutathione-related | M4 | Liver | Glutathione cycle | 0.87 | 0.27 | 0.0097 | 0.081 |
| Glutathione-related | M4 | Plasma | Glutathione cycle | 0.63 | 0.43 | 0.18 | 0.46 |
| Glutathione-related | M8 | Cecal content | Glutathione cycle | -0.24 | 0.81 | 0.77 | 0.95 |
| Glutathione-related | M8 | Liver | Glutathione cycle | 0.98 | 0.16 | 0.00012 | 0.003 |
| Glutathione-related | M8 | Plasma | Glutathione cycle | 0.64 | 0.43 | 0.17 | 0.49 |
| Glutathione-related | M5 | Cecal content | Glutathione cycle | -0.78 | 0.48 | 0.13 | 0.7 |
| Glutathione-related | M5 | Liver | Glutathione cycle | 0.66 | 0.11 | 7.6e-05 | 0.0012 |
| Glutathione-related | M5 | Plasma | Glutathione cycle | 0.68 | 0.25 | 0.021 | 0.08 |
| Ceramides | M0 | Cecal content | Ceramide | -0.20 | 0.16 | 0.24 | 0.51 |
| Ceramides | M0 | Liver | Ceramide | -0.90 | 0.16 | 0.00013 | 0.00041 |
| Ceramides | M0 | Plasma | Ceramide | -0.84 | 0.18 | 0.00042 | 0.0012 |
| Ceramides | M6 | Cecal content | Ceramide | -0.17 | 0.19 | 0.37 | 0.55 |
| Ceramides | M6 | Liver | Ceramide | -0.92 | 0.19 | 0.00058 | 0.0031 |
| Ceramides | M6 | Plasma | Ceramide | -0.63 | 0.15 | 0.0015 | 0.0044 |
| Ceramides | M7 | Cecal content | Ceramide | -0.19 | 0.17 | 0.27 | 0.54 |
| Ceramides | M7 | Liver | Ceramide | -0.90 | 0.17 | 0.00026 | 0.0008 |
| Ceramides | M7 | Plasma | Ceramide | -0.83 | 0.16 | 0.00035 | 0.001 |
| Ceramides | M1 | Cecal content | Ceramide | -0.14 | 0.26 | 0.59 | 0.79 |
| Ceramides | M1 | Liver | Ceramide | -0.92 | 0.26 | 0.0053 | 0.026 |
| Ceramides | M1 | Plasma | Ceramide | -0.52 | 0.25 | 0.063 | 0.22 |
| Ceramides | M2 | Cecal content | Ceramide | -0.14 | 0.27 | 0.62 | 0.83 |
| Ceramides | M2 | Liver | Ceramide | -0.92 | 0.28 | 0.0077 | 0.034 |
| Ceramides | M2 | Plasma | Ceramide | -0.48 | 0.22 | 0.055 | 0.18 |
| Ceramides | M3 | Cecal content | Ceramide | -0.10 | 0.35 | 0.79 | 0.88 |
| Ceramides | M3 | Liver | Ceramide | -0.84 | 0.37 | 0.041 | 0.23 |
| Ceramides | M3 | Plasma | Ceramide | -0.38 | 0.36 | 0.31 | 0.89 |
| Ceramides | M4 | Cecal content | Ceramide | -0.10 | 0.38 | 0.8 | 0.92 |
| Ceramides | M4 | Liver | Ceramide | -0.82 | 0.39 | 0.063 | 0.28 |
| Ceramides | M4 | Plasma | Ceramide | -0.44 | 0.37 | 0.26 | 0.79 |
| Ceramides | M8 | Cecal content | Ceramide | -0.11 | 0.38 | 0.78 | 0.87 |
| Ceramides | M8 | Liver | Ceramide | -0.82 | 0.39 | 0.063 | 0.48 |
| Ceramides | M8 | Plasma | Ceramide | -0.55 | 0.30 | 0.1 | 0.37 |
| Ceramides | M5 | Cecal content | Ceramide | -0.14 | 0.22 | 0.55 | 0.79 |
| Ceramides | M5 | Liver | Ceramide | -0.80 | 0.22 | 0.0044 | 0.18 |
| Ceramides | M5 | Plasma | Ceramide | -0.50 | 0.19 | 0.023 | 0.058 |
| Bile acids | M0 | Cecal content | Bile acid | -0.73 | 0.20 | 0.0037 | 0.0088 |
| Bile acids | M0 | Liver | Bile acid | 0.00 | 0.21 | 0.99 | 0.98 |
| Bile acids | M0 | Plasma | Bile acid | 0.47 | 0.49 | 0.35 | 0.68 |
| Bile acids | M6 | Cecal content | Bile acid | -0.59 | 0.22 | 0.022 | 0.14 |
| Bile acids | M6 | Liver | Bile acid | -0.22 | 0.21 | 0.31 | 0.57 |
| Bile acids | M6 | Plasma | Bile acid | 0.11 | 0.52 | 0.83 | 0.94 |
| Bile acids | M7 | Cecal content | Bile acid | -0.73 | 0.21 | 0.0051 | 0.012 |
| Bile acids | M7 | Liver | Bile acid | -0.01 | 0.22 | 0.96 | 0.99 |
| Bile acids | M7 | Plasma | Bile acid | 0.45 | 0.49 | 0.38 | 0.8 |
| Bile acids | M1 | Cecal content | Bile acid | -0.52 | 0.32 | 0.13 | 0.27 |
| Bile acids | M1 | Liver | Bile acid | -0.62 | 0.25 | 0.029 | 0.12 |
| Bile acids | M1 | Plasma | Bile acid | 0.26 | 0.78 | 0.74 | 0.98 |
| Bile acids | M2 | Cecal content | Bile acid | -0.50 | 0.32 | 0.16 | 0.52 |
| Bile acids | M2 | Liver | Bile acid | -0.64 | 0.25 | 0.028 | 0.077 |
| Bile acids | M2 | Plasma | Bile acid | 0.20 | 0.79 | 0.81 | 0.91 |
| Bile acids | M3 | Cecal content | Bile acid | -0.50 | 0.45 | 0.29 | 0.74 |
| Bile acids | M3 | Liver | Bile acid | -0.97 | 0.35 | 0.017 | 0.077 |
| Bile acids | M3 | Plasma | Bile acid | 0.05 | 1.07 | 0.96 | 1 |
| Bile acids | M4 | Cecal content | Bile acid | -0.57 | 0.47 | 0.26 | 0.67 |
| Bile acids | M4 | Liver | Bile acid | -0.88 | 0.35 | 0.029 | 0.13 |
| Bile acids | M4 | Plasma | Bile acid | 0.05 | 1.15 | 0.96 | 0.99 |
| Bile acids | M8 | Cecal content | Bile acid | -0.63 | 0.45 | 0.19 | 0.56 |
| Bile acids | M8 | Liver | Bile acid | -0.88 | 0.35 | 0.03 | 0.15 |
| Bile acids | M8 | Plasma | Bile acid | 0.38 | 1.06 | 0.73 | 0.9 |
| Bile acids | M5 | Cecal content | Bile acid | -0.57 | 0.28 | 0.062 | 0.33 |
| Bile acids | M5 | Liver | Bile acid | -0.43 | 0.23 | 0.084 | 0.2 |
| Bile acids | M5 | Plasma | Bile acid | 0.21 | 0.67 | 0.76 | 0.89 |
| Ketone bodies / acylcarnitines | M0 | Cecal content | Ketone / FA-oxidation tone | -0.20 | 0.30 | 0.52 | 0.89 |
| Ketone bodies / acylcarnitines | M0 | Liver | Ketone / FA-oxidation tone | 1.17 | 0.30 | 0.0019 | 0.0048 |
| Ketone bodies / acylcarnitines | M0 | Plasma | Ketone / FA-oxidation tone | 0.24 | 0.34 | 0.49 | 0.99 |
| Ketone bodies / acylcarnitines | M6 | Cecal content | Ketone / FA-oxidation tone | -0.47 | 0.31 | 0.15 | 0.35 |
| Ketone bodies / acylcarnitines | M6 | Liver | Ketone / FA-oxidation tone | 1.46 | 0.29 | 0.00038 | 0.015 |
| Ketone bodies / acylcarnitines | M6 | Plasma | Ketone / FA-oxidation tone | 0.01 | 0.37 | 0.98 | 0.98 |
| Ketone bodies / acylcarnitines | M7 | Cecal content | Ketone / FA-oxidation tone | -0.23 | 0.28 | 0.43 | 0.77 |
| Ketone bodies / acylcarnitines | M7 | Liver | Ketone / FA-oxidation tone | 1.19 | 0.30 | 0.0021 | 0.0059 |
| Ketone bodies / acylcarnitines | M7 | Plasma | Ketone / FA-oxidation tone | 0.22 | 0.33 | 0.53 | 0.93 |
| Ketone bodies / acylcarnitines | M1 | Cecal content | Ketone / FA-oxidation tone | -0.15 | 0.49 | 0.76 | 0.97 |
| Ketone bodies / acylcarnitines | M1 | Liver | Ketone / FA-oxidation tone | 1.96 | 0.36 | 0.00021 | 0.0064 |
| Ketone bodies / acylcarnitines | M1 | Plasma | Ketone / FA-oxidation tone | 0.41 | 0.55 | 0.48 | 0.76 |
| Ketone bodies / acylcarnitines | M2 | Cecal content | Ketone / FA-oxidation tone | -0.22 | 0.45 | 0.64 | 1 |
| Ketone bodies / acylcarnitines | M2 | Liver | Ketone / FA-oxidation tone | 2.00 | 0.34 | 0.00016 | 0.018 |
| Ketone bodies / acylcarnitines | M2 | Plasma | Ketone / FA-oxidation tone | 0.34 | 0.53 | 0.53 | 0.95 |
| Ketone bodies / acylcarnitines | M3 | Cecal content | Ketone / FA-oxidation tone | -0.64 | 0.66 | 0.35 | 0.64 |
| Ketone bodies / acylcarnitines | M3 | Liver | Ketone / FA-oxidation tone | 1.89 | 0.61 | 0.01 | 0.071 |
| Ketone bodies / acylcarnitines | M3 | Plasma | Ketone / FA-oxidation tone | 0.30 | 0.77 | 0.71 | 0.98 |
| Ketone bodies / acylcarnitines | M4 | Cecal content | Ketone / FA-oxidation tone | -0.80 | 0.66 | 0.26 | 0.45 |
| Ketone bodies / acylcarnitines | M4 | Liver | Ketone / FA-oxidation tone | 1.62 | 0.51 | 0.01 | 0.053 |
| Ketone bodies / acylcarnitines | M4 | Plasma | Ketone / FA-oxidation tone | 0.21 | 0.81 | 0.8 | 0.95 |
| Ketone bodies / acylcarnitines | M8 | Cecal content | Ketone / FA-oxidation tone | -0.41 | 0.63 | 0.53 | 0.92 |
| Ketone bodies / acylcarnitines | M8 | Liver | Ketone / FA-oxidation tone | 1.68 | 0.59 | 0.017 | 0.1 |
| Ketone bodies / acylcarnitines | M8 | Plasma | Ketone / FA-oxidation tone | 0.56 | 0.74 | 0.47 | 0.95 |
| Ketone bodies / acylcarnitines | M5 | Cecal content | Ketone / FA-oxidation tone | -0.54 | 0.39 | 0.2 | 0.34 |
| Ketone bodies / acylcarnitines | M5 | Liver | Ketone / FA-oxidation tone | 1.79 | 0.30 | 0.0001 | 0.086 |
| Ketone bodies / acylcarnitines | M5 | Plasma | Ketone / FA-oxidation tone | 0.07 | 0.47 | 0.88 | 0.96 |

```
table_s4e <- bind_rows(lapply(names(mats), function(m) bind_rows(lapply(c("M4", "M8"), function(mo) bind_rows(lapply(comp_names(m), function(k)
  coef_loo(comp_data[[m]][[k]], comp_data[[m]], mv_models[[mo]]) |> mutate(compartment = mats[[m]], composite = k, model = mo, .before = 1)))))))
table_s4e |> ktab(caption = "Table S4E. Univariate regression of each composite on diet with depot PC1 + hepatic lipid (M4) or total depot mass + hepatic lipid (M8); donor-linked phenotypes", digits = 2)
```

Table S4E. Univariate regression of each composite on diet with depot
PC1 + hepatic lipid (M4) or total depot mass + hepatic lipid (M8);
donor-linked phenotypes

| compartment | composite | model | b\_diet | se | p | p\_loo\_max | p\_covariate\_1 | p\_covariate\_2 |
| --- | --- | --- | --- | --- | --- | --- | --- | --- |
| Cecal content | BCAA ketoacid/hydroxy-acid | M4 | 1.52 | 0.34 | 0.0012 | 0.0062 | 0.93 | 0.26 |
| Cecal content | n-3 PUFA | M4 | 1.40 | 1.13 | 0.25 | 0.73 | 0.11 | 0.27 |
| Cecal content | Glutathione cycle | M4 | -0.56 | 0.82 | 0.51 | 0.97 | 0.41 | 0.6 |
| Cecal content | Ceramide | M4 | -0.10 | 0.38 | 0.8 | 0.92 | 0.97 | 0.87 |
| Cecal content | Bile acid | M4 | -0.57 | 0.47 | 0.26 | 0.67 | 0.53 | 0.88 |
| Cecal content | Ketone / FA-oxidation tone | M4 | -0.80 | 0.66 | 0.26 | 0.45 | 0.28 | 0.2 |
| Cecal content | BCAA ketoacid/hydroxy-acid | M8 | 1.39 | 0.27 | 0.0005 | 0.01 | 0.043 | 0.012 |
| Cecal content | n-3 PUFA | M8 | 0.86 | 1.29 | 0.52 | 0.68 | 0.7 | 0.67 |
| Cecal content | Glutathione cycle | M8 | -0.24 | 0.81 | 0.77 | 0.95 | 0.38 | 0.54 |
| Cecal content | Ceramide | M8 | -0.11 | 0.38 | 0.78 | 0.87 | 0.91 | 0.84 |
| Cecal content | Bile acid | M8 | -0.63 | 0.45 | 0.19 | 0.56 | 0.24 | 0.91 |
| Cecal content | Ketone / FA-oxidation tone | M8 | -0.41 | 0.63 | 0.53 | 0.92 | 0.13 | 0.91 |
| Liver | BCAA-catabolite | M4 | 1.42 | 0.28 | 0.00045 | 0.0026 | 0.17 | 0.55 |
| Liver | n-3 PUFA | M4 | 1.39 | 0.57 | 0.036 | 0.21 | 0.28 | 0.54 |
| Liver | Glutathione cycle | M4 | 0.87 | 0.27 | 0.0097 | 0.081 | 0.32 | 0.67 |
| Liver | Ceramide | M4 | -0.82 | 0.39 | 0.063 | 0.28 | 0.75 | 0.73 |
| Liver | Bile acid | M4 | -0.88 | 0.35 | 0.029 | 0.13 | 0.25 | 0.31 |
| Liver | Ketone / FA-oxidation tone | M4 | 1.62 | 0.51 | 0.01 | 0.053 | 0.031 | 0.37 |
| Liver | BCAA-catabolite | M8 | 1.44 | 0.29 | 0.00058 | 0.0067 | 0.3 | 0.87 |
| Liver | n-3 PUFA | M8 | 1.45 | 0.54 | 0.023 | 0.17 | 0.13 | 0.66 |
| Liver | Glutathione cycle | M8 | 0.98 | 0.16 | 0.00012 | 0.003 | 0.00086 | 0.17 |
| Liver | Ceramide | M8 | -0.82 | 0.39 | 0.063 | 0.48 | 0.77 | 0.77 |
| Liver | Bile acid | M8 | -0.88 | 0.35 | 0.03 | 0.15 | 0.26 | 0.053 |
| Liver | Ketone / FA-oxidation tone | M8 | 1.68 | 0.59 | 0.017 | 0.1 | 0.14 | 0.67 |
| Plasma | BCAA-parent | M4 | -1.13 | 0.36 | 0.01 | 0.18 | 0.075 | 0.023 |
| Plasma | n-3 PUFA | M4 | 1.57 | 0.32 | 0.00066 | 0.0091 | 0.12 | 0.31 |
| Plasma | Glutathione cycle | M4 | 0.60 | 0.34 | 0.11 | 0.25 | 0.61 | 0.62 |
| Plasma | Ceramide | M4 | -0.64 | 0.31 | 0.063 | 0.44 | 0.28 | 0.7 |
| Plasma | Bile acid | M4 | 0.43 | 0.93 | 0.66 | 0.97 | 0.87 | 0.87 |
| Plasma | Ketone / FA-oxidation tone | M4 | 0.16 | 0.65 | 0.81 | 0.97 | 0.54 | 0.57 |
| Plasma | BCAA-parent | M8 | -1.05 | 0.44 | 0.037 | 0.22 | 0.67 | 0.31 |
| Plasma | n-3 PUFA | M8 | 1.61 | 0.38 | 0.0017 | 0.018 | 0.63 | 0.93 |
| Plasma | Glutathione cycle | M8 | 0.59 | 0.36 | 0.14 | 0.38 | 0.87 | 0.83 |
| Plasma | Ceramide | M8 | -0.87 | 0.20 | 0.0013 | 0.012 | 0.0013 | 0.26 |
| Plasma | Bile acid | M8 | 0.76 | 0.90 | 0.42 | 0.71 | 0.22 | 0.53 |
| Plasma | Ketone / FA-oxidation tone | M8 | 0.34 | 0.66 | 0.62 | 0.96 | 0.4 | 0.72 |

```
lba_pc1 <- bind_rows(lapply(c("M1", "M2", "M4"), function(mo) { co <- ols_diet(class_Y("Bile acids")[, "LIVER"], pool14, mv_models[[mo]])
  tibble(model = mo, b_diet = co["Xdiet", 1], p_diet = co["Xdiet", 4], p_PC1 = co["XPC1", 4]) }))
lba_pc1 |> ktab(caption = "Hepatic bile-acid composite: diet and depot PC1 coefficients in the models that contain PC1", digits = 3)
```

Hepatic bile-acid composite: diet and depot PC1 coefficients in the
models that contain PC1

| model | b\_diet | p\_diet | p\_PC1 |
| --- | --- | --- | --- |
| M1 | -0.616 | 0.029 | 0.0075 |
| M2 | -0.636 | 0.028 | 0.0079 |
| M4 | -0.880 | 0.029 | 0.25 |

The class-level diet term under the conservative model (M4) is also
tested by Freedman–Lane residual permutation: the residuals of the
covariate-only model are permuted 10,000 times and added back to its
fitted values, the full model is refitted, and p = (number of permuted
traces ≥ observed + 1)/10,001. As a one-response check, the class-mean
composite (mean of the three compartment composites) is analyzed by
ANCOVA with the same covariates; the mean cancels where the compartments
differ in direction.

```
perm_hl <- function(Y, d, cv, B = 10000) {
  set.seed(seed); Y <- as.matrix(Y); X <- design(d, cv); Xr <- X[, colnames(X) != "diet", drop = FALSE]
  qr_r <- qr(Xr); fit_r <- qr.fitted(qr_r, Y); res_r <- qr.resid(qr_r, Y); obs <- mv_term(Y, X, "diet")[["HL"]]
  perm <- replicate(B, mv_term(fit_r + res_r[sample.int(nrow(Y)), , drop = FALSE], X, "diet")[["HL"]])
  (sum(perm >= obs) + 1) / (B + 1)
}
table_s4g <- bind_rows(lapply(names(mv_classes), function(cl) {
  Y <- class_Y(cl); cv <- mv_models$M4; cm <- coef_loo(rowMeans(Y), pool14, cv); r <- m4 |> filter(class == cl)
  dirs <- table_s4d |> filter(class == cl, model == "M4") |> pull(b_diet)
  tibble(class = cl, direction = paste(ifelse(dirs > 0, "+", "−"), collapse = ", "), HL = r$HL, F = r$F, p = r$p, p_perm = perm_hl(Y, pool14, cv),
         classmean_F = (cm$b_diet / cm$se)^2, classmean_p = cm$p, classmean_p_loo_max = cm$p_loo_max)
}))
table_s4g |> ktab(caption = "Table S4G. Permutation test and class-mean ANCOVA for the class-level diet term under M4 (diet + depot PC1 + hepatic lipid), 14 pools", digits = 2)
```

Table S4G. Permutation test and class-mean ANCOVA for the class-level
diet term under M4 (diet + depot PC1 + hepatic lipid), 14 pools

| class | direction | HL | F | p | p\_perm | classmean\_F | classmean\_p | classmean\_p\_loo\_max |
| --- | --- | --- | --- | --- | --- | --- | --- | --- |
| Branched-chain amino acids | +, +, − | 4.79 | 12.77 | 0.002 | 0.0017 | 4.80 | 0.053 | 0.17 |
| n-3 polyunsaturated fatty acids | +, +, + | 2.05 | 5.47 | 0.024 | 0.023 | 14.51 | 0.0034 | 0.02 |
| Glutathione-related | −, +, + | 2.43 | 6.47 | 0.016 | 0.02 | 0.71 | 0.42 | 0.87 |
| Ceramides | −, −, − | 0.72 | 1.93 | 0.2 | 0.2 | 4.41 | 0.062 | 0.28 |
| Bile acids | −, −, + | 0.97 | 2.58 | 0.13 | 0.12 | 1.37 | 0.27 | 0.43 |
| Ketone bodies / acylcarnitines | −, +, + | 1.27 | 3.40 | 0.074 | 0.08 | 0.64 | 0.44 | 0.79 |

```
cl4 <- function(cl, col = "p") m4[[col]][m4$class == cl]
cl8 <- function(cl) m8$p[m8$class == cl]
g4 <- function(cl, col) table_s4g[[col]][table_s4g$class == cl]
d4 <- function(cl, m, mo = "M4", col = "p") table_s4d[[col]][table_s4d$class == cl & table_s4d$model == mo & table_s4d$compartment == mats[[m]]]
m0 <- table_s4b |> filter(model == "M0"); m6 <- table_s4b |> filter(model == "M6", class != "Bile acids")
```

Unadjusted, every class differed by diet across the three
compartments (F(3,10) = 4.0 to 69.5, p ≤ 0.040), and with total depot
mass in the model the diet term persisted for every class except bile
acids (p ≤ 9.7e-04). Under M4 (depot PC1 + hepatic lipid), the diet term
was retained for the branched-chain class (F(3,8) = 12.8, p = 0.002, q =
0.012; leave-one-pool-out maximum 0.011), the glutathione-related class
(F = 6.5, p = 0.016, q = 0.047; maximum 0.101), and the n-3 class (F =
5.5, p = 0.024, q = 0.049; maximum 0.067), but not for the
ketone/acylcarnitine (p = 0.074, q = 0.11), bile-acid (p = 0.126, q =
0.15), or ceramide (p = 0.203, q = 0.20) classes. Under M8 (total depot
mass + hepatic lipid), p = 6.3e-04 (branched-chain), 0.002
(glutathione-related), 0.049 (ketone/acylcarnitine), 0.057 (n-3), 0.122
(ceramides), and 0.125 (bile acids). Variance inflation factors in M4
were 4.8, 6.0, 11.6 (diet, PC1, hepatic lipid) and in M8 4.8, 1.7, 6.1;
in the single-covariate models they were ≤ 2.4 for the depot covariates
and 4.6 for hepatic lipid.

Under M4, the hepatic ceramide coefficient had p = 0.063. The cecal
and hepatic branched-chain coefficients had p ≤ 0.001
(leave-one-pool-out maxima ≤ 0.006) and the hepatic glutathione
coefficient p = 0.010 (maximum 0.081). The hepatic bile-acid composite,
with no unadjusted diet effect (Cohen’s d = -0.01), had a negative diet
coefficient in the models containing PC1 (b = -0.62 to -0.88, p =
0.028–0.029; PC1 p = 0.008 in M1). Permutation p-values under M4 were
Branched-chain amino acids 0.002; n-3 polyunsaturated fatty acids 0.023;
Glutathione-related 0.020; Ceramides 0.199; Bile acids 0.125; Ketone
bodies / acylcarnitines 0.080. The class-mean ANCOVA gave F(1,10) =
14.5, p = 0.003 (maximum 0.019) for the n-3 class, p = 0.062 for
ceramides, and p = 0.053 and 0.418 for the branched-chain and
glutathione-related classes, whose compartment composites differ in
sign.

## 16.3 Univariate regressions (descriptive follow-up)

As descriptive follow-up, the relation of each composite to diet and
adiposity is modeled by ordinary least squares with diet group and one
adiposity covariate, using the phenotype of the animals that contributed
to each sample. Variance is partitioned by commonality analysis: unique
diet = R²(full) − R²(covariate), unique covariate = R²(full) − R²(diet),
shared = R²(diet) + R²(covariate) − R²(full); negative shared values
indicate suppression. The diet term is tested by the t-test of its
coefficient in the two-predictor model, with BH-FDR across the 18
composites for each covariate. Residuals are checked by Shapiro–Wilk and
Breusch–Pagan tests.

```
covs <- c(`Subcutaneous fat` = "SQ_FAT_mg", `Body weight` = "FINAL_BW_g", `Mesenteric fat` = "MES_FAT_mg",
          `Total visceral fat` = "VIS_FAT_mg", `Epididymal fat` = "EP_PM_FAT_mg", `Hepatic lipid` = "LIVER_LIPID")
ols_one <- function(d, cv) {
  d$diet <- as.numeric(d$group == "Bean"); d$cov <- d[[cv]]
  f <- lm(score ~ diet + cov, d); r2 <- function(fm) summary(lm(fm, d))$r.squared
  Rd <- r2(score ~ diet); Rc <- r2(score ~ cov); Rf <- summary(f)$r.squared; ci <- confint(f); co <- summary(f)$coefficients
  tibble(R2_diet_alone = Rd, R2_cov_alone = Rc, R2_full = Rf, unique_diet = Rf - Rc, unique_cov = Rf - Rd, shared = Rd + Rc - Rf,
         b_diet = co["diet", 1], se_diet = co["diet", 2], ci_diet_lo = ci["diet", 1], ci_diet_hi = ci["diet", 2], p_diet = co["diet", 4],
         b_cov = co["cov", 1], se_cov = co["cov", 2], ci_cov_lo = ci["cov", 1], ci_cov_hi = ci["cov", 2], p_cov = co["cov", 4],
         VIF = 1 / (1 - summary(lm(cov ~ diet, d))$r.squared), shapiro_p = shapiro.test(resid(f))$p.value, bp_p = unname(bptest(f)$p.value))
}
s4 <- scores |> group_by(label, composite, matrix, n_members) |> group_modify(function(d, k) bind_rows(lapply(names(covs), function(cn)
  ols_one(d, covs[cn]) |> mutate(covariate = cn)))) |> ungroup() |> relocate(covariate, .after = n_members) |>
  group_by(covariate) |> mutate(q_diet = p.adjust(p_diet, "BH")) |> ungroup()
grp_test <- scores |> group_by(label) |> summarise(cohen_d = cohen_d(score, group),
  p_diet_welch = t.test(score[group == "Bean"], score[group == "Control"])$p.value, .groups = "drop") |> mutate(q_diet_welch = p.adjust(p_diet_welch, "BH"))
top_bean <- pool_ph14 |> filter(group == "Bean") |> slice_max(SQ_FAT_mg, n = 1) |> pull(POOL)
s4_drop <- scores |> filter(POOL != top_bean) |> group_by(label) |> group_modify(function(d, k) ols_one(d, "SQ_FAT_mg")) |> ungroup()
within_r <- function(d, drop_pool = NULL) {
  d <- d |> filter(!POOL %in% drop_pool)
  bind_rows(lapply(c("Control", "Bean"), function(gp) { e <- d |> filter(group == gp); ct <- cor.test(e$score, e$SQ_FAT_mg)
    tibble(group = gp, n = nrow(e), r = unname(ct$estimate), lo = ct$conf.int[1], hi = ct$conf.int[2], p = ct$p.value) }))
}
s4_within <- scores |> group_by(label) |>
  group_modify(function(d, k) bind_rows(within_r(d) |> mutate(set = "all"), within_r(d, top_bean) |> mutate(set = "excl_top_bean"))) |> ungroup()
```

The highest-adiposity profiled bean pool, used in the sensitivity
analyses, is pool 49.

```
pick <- function(cv, col) s4 |> filter(covariate == cv) |> select(label, all_of(col))
w <- s4_within |> filter(!(group == "Control" & set == "excl_top_bean")) |>
  mutate(col = paste0("r_", group, ifelse(set == "all", "", "_excl")), val = sprintf("%.2f (p=%.2f)", r, p)) |> select(label, col, val) |>
  pivot_wider(names_from = col, values_from = val)
table_s4f <- s4 |> filter(covariate == "Subcutaneous fat") |> left_join(grp_test, by = "label") |>
  transmute(label, composite, matrix, n = n_members, cohen_d, p_diet_welch, q_diet_welch, R2_diet_pct = 100 * R2_diet_alone,
            unique_diet_pct = 100 * unique_diet, shared_pct = 100 * shared, unique_SQ_pct = 100 * unique_cov, p_diet_given_SQ = p_diet,
            q_given_SQ = q_diet, p_SQ_given_diet = p_cov, VIF, shapiro_p, bp_p) |>
  left_join(pick("Body weight", "p_diet") |> rename(p_diet_given_BW = p_diet), by = "label") |>
  left_join(pick("Hepatic lipid", "p_diet") |> rename(p_diet_given_liver_lipid = p_diet), by = "label") |>
  left_join(s4_drop |> select(label, p_diet_given_SQ_excl_top = p_diet), by = "label") |>
  left_join(w, by = "label") |> arrange(composite, matrix)
table_s4f |> select(-label) |> ktab(caption = sprintf("Table S4F. Regression of all 18 composites on diet and subcutaneous fat (OLS; donor-linked phenotype), with alternative covariates, omission of bean pool %d, diagnostics, and within-group Pearson r", top_bean), digits = 2)
```

Table S4F. Regression of all 18 composites on diet and subcutaneous fat
(OLS; donor-linked phenotype), with alternative covariates, omission of
bean pool 49, diagnostics, and within-group Pearson r

| composite | matrix | n | cohen\_d | p\_diet\_welch | q\_diet\_welch | R2\_diet\_pct | unique\_diet\_pct | shared\_pct | unique\_SQ\_pct | p\_diet\_given\_SQ | q\_given\_SQ | p\_SQ\_given\_diet | VIF | shapiro\_p | bp\_p | p\_diet\_given\_BW | p\_diet\_given\_liver\_lipid | p\_diet\_given\_SQ\_excl\_top | r\_Control | r\_Bean | r\_Bean\_excl |
| --- | --- | --- | --- | --- | --- | --- | --- | --- | --- | --- | --- | --- | --- | --- | --- | --- | --- | --- | --- | --- | --- |
| BCAA ketoacid/hydroxy-acid | CECAL | 6 | 6.75 | 3.1e-08 | 5.6e-07 | 93.00 | 50.52 | 42.48 | 0.02 | 2.3e-06 | 4.1e-05 | 0.86 | 1.79 | 0.98 | 0.89 | 2.4e-05 | 0.00052 | 0.00046 | -0.28 (p=0.54) | 0.07 (p=0.88) | 0.90 (p=0.01) |
| BCAA-catabolite | LIVER | 5 | 5.28 | 1.1e-06 | 1e-05 | 89.04 | 58.29 | 30.76 | 0.78 | 7e-06 | 6.3e-05 | 0.38 | 1.79 | 0.62 | 0.5 | 1.3e-05 | 0.00026 | 0.0021 | -0.29 (p=0.53) | 0.46 (p=0.30) | 0.70 (p=0.12) |
| BCAA-parent | PLASMA | 3 | -1.25 | 0.037 | 0.061 | 31.34 | 33.58 | -2.24 | 5.17 | 0.034 | 0.062 | 0.36 | 1.64 | 0.13 | 0.95 | 0.015 | 0.02 | 0.064 | -0.55 (p=0.20) | -0.19 (p=0.68) | -0.16 (p=0.76) |
| Bile acid | CECAL | 31 | -1.92 | 0.0056 | 0.011 | 51.83 | 17.64 | 34.19 | 3.12 | 0.062 | 0.1 | 0.4 | 1.79 | 0.82 | 0.31 | 0.25 | 0.29 | 0.33 | -0.04 (p=0.93) | 0.33 (p=0.46) | 0.39 (p=0.45) |
| Bile acid | LIVER | 25 | -0.01 | 0.99 | 0.99 | 0.00 | 18.94 | -18.94 | 42.10 | 0.084 | 0.13 | 0.016 | 1.79 | 0.92 | 0.57 | 0.2 | 0.017 | 0.13 | -0.52 (p=0.23) | -0.75 (p=0.05) | -0.64 (p=0.17) |
| Bile acid | PLASMA | 21 | 0.52 | 0.37 | 0.44 | 7.27 | 1.35 | 5.92 | 2.30 | 0.69 | 0.73 | 0.61 | 1.64 | 0.18 | 0.013 | 0.64 | 0.64 | 0.47 | 0.13 (p=0.78) | -0.18 (p=0.70) | -0.68 (p=0.14) |
| Ceramide | CECAL | 14 | -0.66 | 0.25 | 0.32 | 11.19 | 3.04 | 8.15 | 1.28 | 0.55 | 0.62 | 0.7 | 1.79 | 0.65 | 0.34 | 0.78 | 0.79 | 0.63 | 0.08 (p=0.87) | 0.13 (p=0.77) | -0.08 (p=0.88) |
| Ceramide | LIVER | 13 | -2.94 | 0.00014 | 0.00049 | 71.64 | 31.61 | 40.04 | 1.10 | 0.0044 | 0.013 | 0.52 | 1.79 | 0.65 | 0.18 | 0.003 | 0.041 | 0.18 | 0.71 (p=0.08) | -0.03 (p=0.95) | -0.53 (p=0.28) |
| Ceramide | PLASMA | 17 | -2.58 | 0.0011 | 0.0028 | 65.95 | 15.93 | 50.02 | 14.30 | 0.013 | 0.028 | 0.017 | 1.64 | 0.28 | 0.69 | 0.28 | 0.068 | 0.064 | -0.08 (p=0.87) | 0.79 (p=0.03) | 0.54 (p=0.27) |
| Glutathione cycle | CECAL | 19 | -0.83 | 0.15 | 0.21 | 16.65 | 18.92 | -2.28 | 3.84 | 0.13 | 0.17 | 0.48 | 1.79 | 0.93 | 0.58 | 0.31 | 0.62 | 0.7 | -0.09 (p=0.84) | -0.33 (p=0.47) | 0.35 (p=0.50) |
| Glutathione cycle | LIVER | 30 | 4.16 | 2.9e-05 | 0.00013 | 83.46 | 22.27 | 61.19 | 9.99 | 7.6e-05 | 0.00034 | 0.0018 | 1.79 | 0.16 | 0.41 | 0.0041 | 0.012 | 0.0012 | -0.19 (p=0.69) | -0.93 (p=0.00) | -0.58 (p=0.23) |
| Glutathione cycle | PLASMA | 26 | 1.90 | 0.004 | 0.009 | 51.35 | 32.21 | 19.14 | 0.01 | 0.021 | 0.041 | 0.96 | 1.64 | 0.099 | 0.97 | 0.045 | 0.096 | 0.13 | 0.13 (p=0.78) | -0.03 (p=0.94) | 0.08 (p=0.88) |
| Ketone / FA-oxidation tone | CECAL | 3 | -0.35 | 0.52 | 0.55 | 3.49 | 14.29 | -10.79 | 12.85 | 0.2 | 0.24 | 0.22 | 1.79 | 0.88 | 0.35 | 0.66 | 0.35 | 0.0038 | -0.75 (p=0.05) | -0.22 (p=0.63) | -0.89 (p=0.02) |
| Ketone / FA-oxidation tone | LIVER | 10 | 2.11 | 0.0062 | 0.011 | 56.58 | 73.84 | -17.26 | 20.03 | 0.0001 | 0.00038 | 0.011 | 1.79 | 0.35 | 0.44 | 0.0005 | 0.01 | 0.086 | -0.22 (p=0.63) | 0.80 (p=0.03) | 0.14 (p=0.79) |
| Ketone / FA-oxidation tone | PLASMA | 12 | 0.38 | 0.5 | 0.55 | 4.00 | 0.70 | 3.30 | 1.36 | 0.78 | 0.78 | 0.7 | 1.64 | 0.29 | 0.03 | 0.45 | 0.79 | 0.79 | -0.17 (p=0.71) | -0.12 (p=0.80) | -0.29 (p=0.57) |
| n-3 PUFA | CECAL | 5 | 1.26 | 0.047 | 0.071 | 31.73 | 18.91 | 12.82 | 0.05 | 0.11 | 0.15 | 0.93 | 1.79 | 0.073 | 0.32 | 0.53 | 0.44 | 0.52 | 0.03 (p=0.95) | 0.05 (p=0.92) | -0.54 (p=0.27) |
| n-3 PUFA | LIVER | 6 | 3.02 | 0.00052 | 0.0016 | 72.73 | 18.51 | 54.22 | 9.64 | 0.0059 | 0.015 | 0.032 | 1.79 | 0.4 | 0.2 | 0.037 | 0.05 | 0.00018 | 0.18 (p=0.70) | -0.75 (p=0.05) | 0.76 (p=0.08) |
| n-3 PUFA | PLASMA | 5 | 4.66 | 2.5e-06 | 1.5e-05 | 86.34 | 42.05 | 44.29 | 1.57 | 6.9e-05 | 0.00034 | 0.26 | 1.64 | 0.27 | 0.5 | 0.00034 | 0.001 | 0.0067 | -0.51 (p=0.24) | -0.30 (p=0.52) | 0.51 (p=0.30) |

```
sig_sq <- table_s4f |> filter(q_given_SQ < 0.05) |> arrange(p_diet_given_SQ)
robust <- s4 |> group_by(label) |> summarise(max_p = max(p_diet), .groups = "drop")
lba <- table_s4f |> filter(label == "Liver Bile acid"); lba_ll <- s4 |> filter(label == "Liver Bile acid", covariate == "Hepatic lipid")
robust |> arrange(max_p) |> ktab(caption = "Largest adjusted diet p-value across the six adiposity covariates")
```

Largest adjusted diet p-value across the six adiposity covariates

| label | max\_p |
| --- | --- |
| Liver BCAA-catabolite | 0.00026 |
| Cecal BCAA ketoacid/hydroxy-acid | 0.00052 |
| Plasma n-3 PUFA | 0.001 |
| Liver Ketone / FA-oxidation tone | 0.01 |
| Liver Glutathione cycle | 0.012 |
| Liver Ceramide | 0.041 |
| Liver n-3 PUFA | 0.05 |
| Plasma Glutathione cycle | 0.096 |
| Plasma BCAA-parent | 0.21 |
| Plasma Ceramide | 0.28 |
| Cecal Bile acid | 0.29 |
| Cecal n-3 PUFA | 0.53 |
| Cecal Glutathione cycle | 0.62 |
| Cecal Ketone / FA-oxidation tone | 0.66 |
| Plasma Bile acid | 0.71 |
| Plasma Ketone / FA-oxidation tone | 0.79 |
| Cecal Ceramide | 0.79 |
| Liver Bile acid | 0.99 |

After adjustment for subcutaneous fat, 9 of 18 composites retained a
diet association at FDR < 0.05 (adjusted p ≤ 0.021, q ≤ 0.041). The
liver bile-acid composite showed no diet effect (Cohen’s d = -0.01) and
a negative association with subcutaneous fat (p = 0.016) and hepatic
lipid (p = 0.009).

```
w_all <- s4_within |> filter(set == "all")
flip <- s4_within |> filter(group == "Bean") |> select(label, set, r) |> pivot_wider(names_from = set, values_from = r) |>
  filter(sign(all) != sign(excl_top_bean))
```

Within-group correlations between composites and subcutaneous fat
were imprecise (n = 7; median 95% CI width 1.48; range of bounds -0.99
to 0.97), and 6 of 18 bean-group correlations changed sign when bean
pool 49 was omitted.

## 16.4 Figure 7 and table export

```
ld <- as_tibble(pca_dep$rotation[, 1:2], rownames = "var") |> mutate(depot = names(dep_vars)[match(var, dep_vars)])
arrow_k <- 0.75 * max(abs(pca_dep$x[, 1:2])) / max(abs(pca_dep$rotation[, 1:2]))
f7b <- ggplot(pool14, aes(PC1, PC2)) +
  geom_hline(yintercept = 0, colour = "grey85") + geom_vline(xintercept = 0, colour = "grey85") +
  geom_segment(data = ld, aes(x = 0, y = 0, xend = arrow_k * PC1, yend = arrow_k * PC2), inherit.aes = FALSE,
               arrow = arrow(length = unit(0.15, "cm")), linewidth = 0.4) +
  geom_text(data = ld, aes(x = 1.12 * arrow_k * PC1, y = 1.12 * arrow_k * PC2, label = depot), inherit.aes = FALSE, size = 3) +
  geom_point(aes(colour = group, shape = group), size = 2.6) +
  scale_colour_manual(values = grp_col, name = NULL) + scale_shape_manual(values = grp_shp, name = NULL) +
  scale_x_continuous(expand = expansion(mult = 0.12)) + theme(legend.position = "inside", legend.position.inside = c(0.9, 0.12)) +
  labs(title = "B  Principal components of the four depot masses",
       x = sprintf("PC1, depot pattern (%.0f%% of variance)", ve[1]), y = sprintf("PC2, total fat (%.0f%% of variance)", ve[2]))
f7 <- f7a + f7b + plot_layout(widths = c(1, 1))
f7
```

```
save_fig(f7, "Figure_7_adiposity", 12, 4.4)
```

```
write_xlsx(list(Table_1 = table1, models = tibble(model = names(mv_models), predictors = unname(model_lab))),
           file.path(out_dir, "tables", "Table_1_class_MANCOVA.xlsx"))
write_xlsx(list(S4A_phenotype = table_s4a, S4A_depot_PCA = table_s4a_pc,
                S4A_Hotelling = tibble(T2 = ht[["T2"]], F = ht[["F"]], df1 = ht[["df1"]], df2 = ht[["df2"]], p = ht[["p"]]),
                S4B_class_MANCOVA = table_s4b, S4B_VIF = vif_tab, S4C_compartment_MANCOVA = table_s4c,
                S4D_compartment_coefficients = table_s4d, S4E_univariate_M4_M8 = table_s4e,
                S4F_univariate_SQ = table_s4f, S4F_all_covariates = s4, S4F_without_top_bean_pool = s4_drop, S4F_within_group = s4_within,
                S4G_permutation_classmean = table_s4g, composite_membership = memb, per_sample_scores = scores),
           file.path(out_dir, "tables", "Table_S4_adiposity_adjustment.xlsx"))
```

# 17 Cage-aware sensitivity analysis

Diets were assigned to whole cages (4 cages of 5 mice per diet) with
body-weight balancing by exchange of individual animals, so the cage was
the unit of diet assignment and delivery and the treatment-level
replication is 4 cages per diet; the allocation was balanced rather than
an unrestricted randomization of cages, so the analysis below is
model-based. Pools were formed by adjacent adiposity rank without regard
to cage, so a pool holds two animals from one cage or from two cages,
and the 14 profiled pools draw on 8 cages (multiple membership). The
cage of each donor is read from the pooling crosswalk
(`COSU-01-23VW_perpool_matrix_R2.xlsx`, sheet
`Pool_crosswalk`, columns
`POOL ANIMAL DONOR 1 CAGE` and
`POOL ANIMAL DONOR 2 CAGE`). The sensitivity analysis is a
multiple-membership linear mixed model, `y = X b + W u + e`,
with `u ~ N(0, s2_cage I)` over the 8 cages,
`e ~ N(0, s2_res I)`, and `W` the 14 × 8
membership matrix carrying weight 1/2 on each donor cage (1 on a single
cage for a same-cage pool; for plasma, the cage(s) of the contributing
donor(s)). The variance ratio is estimated by profile REML, the diet
coefficient by generalized least squares at the estimate, and diet is
tested by an approximate Wald t statistic on 6 denominator degrees of
freedom (8 cages − 2 diets) with a 95% confidence interval on the same t
distribution. Each model is also refitted with every pool containing a
donor from one cage omitted in turn (leave-one-cage-out; 10–12 pools and
7 cages remain, and the Wald test then uses 5 degrees of freedom), and
the class-level MANCOVA of Table 1 is refitted the same way on the
pool-level model. A cage variance estimated at zero means that eight
cages do not separate cage variance from residual variance, not that
cage effects are absent.

```
cage_cols <- c("POOL ANIMAL DONOR 1 CAGE", "POOL ANIMAL DONOR 2 CAGE")
stopifnot(all(cage_cols %in% names(cw_raw)))
cg <- cw_raw |> transmute(POOL, cage1 = as.integer(.data[[cage_cols[1]]]), cage2 = as.integer(.data[[cage_cols[2]]]))
cw14 <- cw14 |> select(-any_of(c("cage1", "cage2"))) |> left_join(cg, by = "POOL")
pool14 <- pool14 |> select(-any_of(c("cage1", "cage2"))) |> left_join(cg, by = "POOL")
stopifnot(!anyNA(pool14$cage1), !anyNA(pool14$cage2))
cages <- sort(unique(c(pool14$cage1, pool14$cage2)))
cage_diet <- sapply(cages, function(k) as.character(unique(pool14$group[pool14$cage1 == k | pool14$cage2 == k])))
stopifnot(length(cage_diet) == length(cages)); names(cage_diet) <- cages
memb_W <- function(c1, c2, w1 = rep(0.5, length(c1)), w2 = rep(0.5, length(c1))) {  # rows = pools (pool14 order), columns = cages
  W <- matrix(0, length(c1), length(cages), dimnames = list(NULL, cages))
  for (i in seq_along(c1)) { W[i, as.character(c1[i])] <- W[i, as.character(c1[i])] + w1[i]; if (w2[i] > 0) W[i, as.character(c2[i])] <- W[i, as.character(c2[i])] + w2[i] }
  W
}
W_pool <- memb_W(pool14$cage1, pool14$cage2)
pl <- cw14[match(pool14$POOL, cw14$POOL), ]
pl_n <- (pl$plasma_uL1 > 0) + (pl$plasma_uL2 > 0)
W_plasma <- memb_W(pool14$cage1, pool14$cage2, (pl$plasma_uL1 > 0) / pl_n, (pl$plasma_uL2 > 0) / pl_n)
stopifnot(all(abs(rowSums(W_pool) - 1) < 1e-12), all(abs(rowSums(W_plasma) - 1) < 1e-12))
same_cage <- pool14$POOL[pool14$cage1 == pool14$cage2]
tibble(cage = cages, diet = cage_diet, pools = sapply(seq_along(cages), function(j) paste(pool14$POOL[W_pool[, j] > 0], collapse = ", ")),
       n_pools_touched = colSums(W_pool > 0)) |> ktab(caption = "Cages represented among the 14 profiled pools, with the pools that hold a donor from each cage")
```

Cages represented among the 14 profiled pools, with the pools that hold
a donor from each cage

| cage | diet | pools | n\_pools\_touched |
| --- | --- | --- | --- |
| 1 | Bean | 19, 24, 49 | 3 |
| 4 | Control | 31, 36 | 2 |
| 6 | Bean | 9, 14, 29 | 3 |
| 8 | Bean | 19, 24, 29, 49 | 4 |
| 9 | Control | 6, 16, 26 | 3 |
| 14 | Control | 16, 26, 31, 36 | 4 |
| 17 | Control | 11, 21 | 2 |
| 18 | Bean | 9, 34 | 2 |

Cages 4, 9, 14, 17 received the control diet and cages 1, 6, 8, 18
the bean diet; all 4 cages of each diet contribute to the profiled
pools. 5 of the 14 pools (6, 11, 14, 21, 34) hold two animals from one
cage and 9 span two cages. Omitting all pools that hold a donor from one
cage removes 2–4 pools.

```
mm_lmm <- function(y, X, W, df_contain = ncol(W) - 2) {  # multiple-membership LMM, profile REML over lambda = s2_cage / s2_res
  n <- nrow(X); k <- ncol(X); G <- W %*% t(W); I <- diag(n)
  reml_nll <- function(loglam) {
    V <- I + exp(loglam) * G; Vi <- solve(V); C <- t(X) %*% Vi %*% X
    b <- solve(C, t(X) %*% Vi %*% y); r <- y - X %*% b; s2 <- drop(t(r) %*% Vi %*% r) / (n - k)
    0.5 * ((n - k) * log(s2) + as.numeric(determinant(V, logarithm = TRUE)$modulus) + as.numeric(determinant(C, logarithm = TRUE)$modulus)) + 0.5 * (n - k)
  }
  op <- optimize(reml_nll, c(-12, 8)); nll0 <- reml_nll(-30)
  lam <- if (nll0 <= op$objective + 1e-9) 0 else exp(op$minimum)
  V <- I + lam * G; Vi <- solve(V); C <- solve(t(X) %*% Vi %*% X); b <- unname(drop(C %*% t(X) %*% Vi %*% y)); r <- y - X %*% b
  s2e <- drop(t(r) %*% Vi %*% r) / (n - k); se <- unname(sqrt(diag(C) * s2e)); tt <- b / se; tc <- qt(0.975, df_contain)
  c(b_diet = b[2], se_diet = se[2], ci_lo = b[2] - tc * se[2], ci_hi = b[2] + tc * se[2], t_diet = tt[2],
    p_wald = 2 * pt(abs(tt[2]), df_contain, lower.tail = FALSE), df = df_contain, s2_cage = lam * s2e, s2_resid = s2e, icc = lam / (1 + lam))
}
loco_keep <- lapply(seq_along(cages), function(j) W_pool[, j] == 0)  # pools retained when cage j is omitted
names(loco_keep) <- cages
mm_loco <- function(y, X, W, b_full) {  # leave-one-cage-out for the mixed model: max Wald p and the range of the diet coefficient
  out <- sapply(loco_keep, function(keep) { Wk <- W[keep, colSums(W[keep, , drop = FALSE]) > 0, drop = FALSE]
    r <- mm_lmm(y[keep], X[keep, , drop = FALSE], Wk); c(r[["b_diet"]], r[["p_wald"]]) })
  c(loco_p_max = max(out[2, ]), loco_b_min = min(out[1, ]), loco_b_max = max(out[1, ]), loco_sign_consistent = all(sign(out[1, ]) == sign(b_full)))
}
```

```
table_s8a <- bind_rows(lapply(names(mv_classes), function(cl) { Y <- class_Y(cl); bind_rows(lapply(c("M0", "M4"), function(mo) bind_rows(lapply(names(mats), function(m) {
  cv <- mv_models[[mo]]; X <- design(pool14, cv); Wm <- if (m == "PLASMA") W_plasma else W_pool
  co <- ols_diet(Y[, m], pool14, cv); r <- mm_lmm(Y[, m], X, Wm); lo <- mm_loco(Y[, m], X, Wm, r[["b_diet"]])
  tibble(class = cl, compartment = mats[[m]], model = mo, b_ols = co["Xdiet", 1], p_ols = co["Xdiet", 4],
         b_mm = r[["b_diet"]], se_mm = r[["se_diet"]], ci_lo = r[["ci_lo"]], ci_hi = r[["ci_hi"]], p_mm_df6 = r[["p_wald"]],
         s2_cage = r[["s2_cage"]], s2_resid = r[["s2_resid"]], icc = r[["icc"]],
         loco_p_max = lo[["loco_p_max"]], loco_b_min = lo[["loco_b_min"]], loco_b_max = lo[["loco_b_max"]], loco_sign_consistent = as.logical(lo[["loco_sign_consistent"]]))
})))) }))
table_s8a |> ktab(caption = "Table S8A. Per-composite multiple-membership mixed model (cage random effect): diet coefficient with 95% CI and approximate Wald p on 6 df, cage variance, and leave-one-cage-out sensitivity, unadjusted (M0) and adjusted for depot PC1 + hepatic lipid (M4)", digits = 3)
```

Table S8A. Per-composite multiple-membership mixed model (cage random
effect): diet coefficient with 95% CI and approximate Wald p on 6 df,
cage variance, and leave-one-cage-out sensitivity, unadjusted (M0) and
adjusted for depot PC1 + hepatic lipid (M4)

| class | compartment | model | b\_ols | p\_ols | b\_mm | se\_mm | ci\_lo | ci\_hi | p\_mm\_df6 | s2\_cage | s2\_resid | icc | loco\_p\_max | loco\_b\_min | loco\_b\_max | loco\_sign\_consistent |
| --- | --- | --- | --- | --- | --- | --- | --- | --- | --- | --- | --- | --- | --- | --- | --- | --- |
| Branched-chain amino acids | Cecal content | M0 | 2.047 | 2.7e-08 | 2.050 | 0.211 | 1.533 | 2.567 | 6.9e-05 | 0.049 | 0.068 | 0.420 | 0.004 | 1.939 | 2.189 | TRUE |
| Branched-chain amino acids | Liver | M0 | 1.299 | 4.1e-07 | 1.318 | 0.181 | 0.876 | 1.760 | 0.00034 | 0.041 | 0.040 | 0.505 | 0.007 | 1.180 | 1.505 | TRUE |
| Branched-chain amino acids | Plasma | M0 | -0.574 | 0.037 | -0.555 | 0.255 | -1.179 | 0.068 | 0.072 | 0.013 | 0.201 | 0.063 | 0.238 | -0.704 | -0.347 | TRUE |
| Branched-chain amino acids | Cecal content | M4 | 1.521 | 0.0012 | 1.513 | 0.400 | 0.535 | 2.490 | 0.0091 | 0.064 | 0.056 | 0.533 | 0.116 | 1.254 | 1.718 | TRUE |
| Branched-chain amino acids | Liver | M4 | 1.419 | 0.00045 | 1.387 | 0.297 | 0.661 | 2.112 | 0.0034 | 0.011 | 0.051 | 0.183 | 0.067 | 1.051 | 1.638 | TRUE |
| Branched-chain amino acids | Plasma | M4 | -1.204 | 0.023 | -1.204 | 0.450 | -2.305 | -0.104 | 0.037 | 0.000 | 0.148 | 0.000 | 0.081 | -3.061 | -0.873 | TRUE |
| n-3 polyunsaturated fatty acids | Cecal content | M0 | 1.280 | 0.036 | 1.280 | 0.542 | -0.046 | 2.607 | 0.056 | 0.000 | 1.028 | 0.000 | 0.334 | 0.897 | 1.788 | TRUE |
| n-3 polyunsaturated fatty acids | Liver | M0 | 1.446 | 0.00011 | 1.446 | 0.256 | 0.821 | 2.072 | 0.0013 | 0.000 | 0.229 | 0.000 | 0.022 | 1.311 | 1.722 | TRUE |
| n-3 polyunsaturated fatty acids | Plasma | M0 | 1.682 | 1.6e-06 | 1.681 | 0.257 | 1.053 | 2.309 | 0.00061 | 0.082 | 0.078 | 0.512 | 0.011 | 1.520 | 2.021 | TRUE |
| n-3 polyunsaturated fatty acids | Cecal content | M4 | 1.398 | 0.25 | 1.434 | 1.168 | -1.423 | 4.292 | 0.27 | 0.080 | 0.901 | 0.081 | 0.849 | -0.800 | 2.278 | FALSE |
| n-3 polyunsaturated fatty acids | Liver | M4 | 1.386 | 0.036 | 1.386 | 0.572 | -0.013 | 2.786 | 0.052 | 0.000 | 0.239 | 0.000 | 0.643 | -0.599 | 2.263 | FALSE |
| n-3 polyunsaturated fatty acids | Plasma | M4 | 1.603 | 0.0044 | 1.886 | 0.510 | 0.639 | 3.134 | 0.01 | 0.114 | 0.075 | 0.601 | 0.079 | 1.419 | 2.745 | TRUE |
| Glutathione-related | Cecal content | M0 | -0.547 | 0.15 | -0.546 | 0.355 | -1.414 | 0.321 | 0.17 | 0.003 | 0.435 | 0.007 | 0.594 | -1.036 | -0.233 | TRUE |
| Glutathione-related | Liver | M0 | 0.948 | 5e-06 | 0.939 | 0.164 | 0.537 | 1.340 | 0.0012 | 0.033 | 0.035 | 0.481 | 0.019 | 0.793 | 1.140 | TRUE |
| Glutathione-related | Plasma | M0 | 0.646 | 0.0039 | 0.648 | 0.196 | 0.168 | 1.128 | 0.016 | 0.016 | 0.104 | 0.132 | 0.097 | 0.553 | 0.753 | TRUE |
| Glutathione-related | Cecal content | M4 | -0.557 | 0.51 | -0.567 | 0.824 | -2.583 | 1.449 | 0.52 | 0.014 | 0.479 | 0.027 | 0.862 | -1.224 | 0.372 | FALSE |
| Glutathione-related | Liver | M4 | 0.871 | 0.0097 | 0.770 | 0.309 | 0.014 | 1.527 | 0.047 | 0.040 | 0.033 | 0.547 | 0.967 | -0.015 | 1.321 | FALSE |
| Glutathione-related | Plasma | M4 | 0.626 | 0.18 | 0.626 | 0.430 | -0.426 | 1.677 | 0.2 | 0.000 | 0.135 | 0.000 | 0.808 | -0.277 | 0.812 | FALSE |
| Ceramides | Cecal content | M0 | -0.196 | 0.24 | -0.196 | 0.159 | -0.586 | 0.194 | 0.26 | 0.000 | 0.089 | 0.000 | 0.500 | -0.318 | -0.117 | TRUE |
| Ceramides | Liver | M0 | -0.902 | 0.00013 | -0.908 | 0.199 | -1.394 | -0.422 | 0.0038 | 0.033 | 0.079 | 0.295 | 0.046 | -1.024 | -0.619 | TRUE |
| Ceramides | Plasma | M0 | -0.844 | 0.00042 | -0.825 | 0.199 | -1.312 | -0.339 | 0.006 | 0.029 | 0.083 | 0.257 | 0.050 | -1.072 | -0.691 | TRUE |
| Ceramides | Cecal content | M4 | -0.100 | 0.8 | -0.100 | 0.380 | -1.031 | 0.831 | 0.8 | 0.000 | 0.106 | 0.000 | 0.933 | -0.358 | -0.036 | TRUE |
| Ceramides | Liver | M4 | -0.818 | 0.063 | -0.773 | 0.437 | -1.842 | 0.296 | 0.13 | 0.045 | 0.091 | 0.333 | 0.347 | -1.106 | -0.515 | TRUE |
| Ceramides | Plasma | M4 | -0.441 | 0.26 | -0.441 | 0.379 | -1.369 | 0.488 | 0.29 | 0.006 | 0.097 | 0.056 | 0.932 | -0.906 | 0.975 | FALSE |
| Bile acids | Cecal content | M0 | -0.734 | 0.0037 | -0.734 | 0.204 | -1.234 | -0.234 | 0.011 | 0.000 | 0.146 | 0.000 | 0.073 | -1.081 | -0.674 | TRUE |
| Bile acids | Liver | M0 | -0.004 | 0.99 | 0.005 | 0.260 | -0.632 | 0.642 | 0.99 | 0.058 | 0.132 | 0.305 | 0.969 | -0.115 | 0.247 | FALSE |
| Bile acids | Plasma | M0 | 0.471 | 0.35 | 0.310 | 0.558 | -1.055 | 1.675 | 0.6 | 0.254 | 0.606 | 0.295 | 0.707 | -0.275 | 0.717 | FALSE |
| Bile acids | Cecal content | M4 | -0.570 | 0.26 | -0.570 | 0.473 | -1.727 | 0.586 | 0.27 | 0.000 | 0.163 | 0.000 | 0.614 | -0.838 | 0.624 | FALSE |
| Bile acids | Liver | M4 | -0.880 | 0.029 | -0.880 | 0.346 | -1.726 | -0.034 | 0.044 | 0.000 | 0.087 | 0.000 | 0.782 | -1.120 | -0.255 | TRUE |
| Bile acids | Plasma | M4 | 0.055 | 0.96 | 0.005 | 1.242 | -3.034 | 3.044 | 1 | 0.390 | 0.665 | 0.370 | 0.983 | -0.983 | 0.780 | FALSE |
| Ketone bodies / acylcarnitines | Cecal content | M0 | -0.199 | 0.52 | -0.170 | 0.446 | -1.261 | 0.920 | 0.72 | 0.270 | 0.210 | 0.562 | 0.971 | -0.499 | 0.074 | FALSE |
| Ketone bodies / acylcarnitines | Liver | M0 | 1.169 | 0.0019 | 1.169 | 0.296 | 0.445 | 1.892 | 0.0075 | 0.000 | 0.306 | 0.000 | 0.065 | 0.956 | 1.443 | TRUE |
| Ketone bodies / acylcarnitines | Plasma | M0 | 0.243 | 0.49 | 0.061 | 0.427 | -0.984 | 1.105 | 0.89 | 0.245 | 0.189 | 0.564 | 0.965 | -0.343 | 0.716 | FALSE |
| Ketone bodies / acylcarnitines | Cecal content | M4 | -0.799 | 0.26 | -0.727 | 0.798 | -2.680 | 1.226 | 0.4 | 0.390 | 0.160 | 0.710 | 0.930 | -1.361 | 0.098 | FALSE |
| Ketone bodies / acylcarnitines | Liver | M4 | 1.617 | 0.01 | 1.617 | 0.514 | 0.360 | 2.875 | 0.02 | 0.000 | 0.193 | 0.000 | 0.092 | 1.129 | 3.036 | TRUE |
| Ketone bodies / acylcarnitines | Plasma | M4 | 0.210 | 0.8 | -0.085 | 0.728 | -1.868 | 1.697 | 0.91 | 0.437 | 0.079 | 0.847 | 0.940 | -0.365 | 0.848 | FALSE |

```
table_s8b <- bind_rows(lapply(names(mv_classes), function(cl) { Y <- class_Y(cl); bind_rows(lapply(c("M0", "M4"), function(mo) {
  cv <- mv_models[[mo]]; X <- design(pool14, cv); par <- mv_term(Y, X, "diet")
  lp <- sapply(loco_keep, function(keep) mv_term(Y[keep, , drop = FALSE], X[keep, , drop = FALSE], "diet")[["p"]])
  tibble(class = cl, model = mo, F = par[["F"]], p = par[["p"]], loco_p_max = max(lp), loco_p_min = min(lp), n_pools_min = min(sapply(loco_keep, sum)))
})) })) |> group_by(model) |> mutate(q = p.adjust(p, "BH")) |> ungroup()
table_s8b |> ktab(caption = "Table S8B. Class-level MANCOVA (pool-level model of Table 1): leave-one-cage-out sensitivity, every pool holding a donor from the omitted cage removed", digits = 3)
```

Table S8B. Class-level MANCOVA (pool-level model of Table 1):
leave-one-cage-out sensitivity, every pool holding a donor from the
omitted cage removed

| class | model | F | p | loco\_p\_max | loco\_p\_min | n\_pools\_min | q |
| --- | --- | --- | --- | --- | --- | --- | --- |
| Branched-chain amino acids | M0 | 69.489 | 5.3e-07 | 0.000 | 0.000 | 10 | 3.2e-06 |
| Branched-chain amino acids | M4 | 12.769 | 0.002 | 0.055 | 0.002 | 10 | 0.012 |
| n-3 polyunsaturated fatty acids | M0 | 23.913 | 7e-05 | 0.003 | 0.000 | 10 | 0.00014 |
| n-3 polyunsaturated fatty acids | M4 | 5.470 | 0.024 | 0.195 | 0.006 | 10 | 0.049 |
| Glutathione-related | M0 | 33.472 | 1.6e-05 | 0.003 | 0.000 | 10 | 4.8e-05 |
| Glutathione-related | M4 | 6.473 | 0.016 | 0.961 | 0.011 | 10 | 0.047 |
| Ceramides | M0 | 18.462 | 0.00021 | 0.017 | 0.000 | 10 | 0.00032 |
| Ceramides | M4 | 1.932 | 0.2 | 0.601 | 0.079 | 10 | 0.2 |
| Bile acids | M0 | 4.036 | 0.04 | 0.281 | 0.017 | 10 | 0.04 |
| Bile acids | M4 | 2.581 | 0.13 | 0.968 | 0.034 | 10 | 0.15 |
| Ketone bodies / acylcarnitines | M0 | 5.048 | 0.022 | 0.188 | 0.000 | 10 | 0.026 |
| Ketone bodies / acylcarnitines | M4 | 3.398 | 0.074 | 0.279 | 0.006 | 10 | 0.11 |

```
pool_order <- function(m) match(M[[m]]$mmeta$POOL, pool14$POOL)
feat_cage <- bind_rows(lapply(names(M), function(m) {
  Wm <- (if (m == "PLASMA") W_plasma else W_pool)[pool_order(m), , drop = FALSE]; g <- as.numeric(M[[m]]$g == "Bean"); X0 <- cbind(1, diet = g)
  l2 <- log2(M[[m]]$x[, M[[m]]$host, drop = FALSE]); d <- DA[[m]] |> filter(CHEM_ID %in% M[[m]]$host)
  res <- t(apply(l2, 2, function(y) { r <- mm_lmm(y, X0, Wm); c(r[["b_diet"]], r[["se_diet"]], r[["p_wald"]], r[["s2_cage"]], r[["s2_resid"]], r[["icc"]]) }))
  colnames(res) <- c("b_mm", "se_mm", "p_mm_df6", "s2_cage", "s2_resid", "icc")
  tibble(matrix = m, CHEM_ID = colnames(l2)) |> bind_cols(as_tibble(res)) |>
    left_join(d |> select(CHEM_ID, name, log2fc, p_welch = p), by = "CHEM_ID") |>
    mutate(q_welch = p.adjust(p_welch, "BH"), q_mm_df6 = p.adjust(p_mm_df6, "BH"))  # BH within the host feature set
}))
table_s8c <- feat_cage |> group_by(matrix) |> summarise(host_features = n(), welch_q05 = sum(q_welch < 0.05), mm_df6_q05 = sum(q_mm_df6 < 0.05),
  retained_of_welch = sum(q_welch < 0.05 & q_mm_df6 < 0.05), pct_retained = 100 * retained_of_welch / welch_q05,
  cage_var_zero_pct = 100 * mean(s2_cage == 0), icc_median = median(icc), icc_q90 = quantile(icc, 0.9), .groups = "drop") |> mutate(matrix = mats[matrix])
table_s8c |> ktab(caption = "Table S8C. Host features by matrix: FDR < 0.05 by Welch's t-test and by the cage-aware mixed model (6 df), and the cage variance component", digits = 2)
```

Table S8C. Host features by matrix: FDR < 0.05 by Welch’s t-test and
by the cage-aware mixed model (6 df), and the cage variance component

| matrix | host\_features | welch\_q05 | mm\_df6\_q05 | retained\_of\_welch | pct\_retained | cage\_var\_zero\_pct | icc\_median | icc\_q90 |
| --- | --- | --- | --- | --- | --- | --- | --- | --- |
| Cecal content | 768 | 401 | 262 | 262 | 65.34 | 35.29 | 0.26 | 0.75 |
| Liver | 948 | 352 | 252 | 252 | 71.59 | 53.80 | 0.00 | 0.59 |
| Plasma | 895 | 235 | 133 | 133 | 56.60 | 35.42 | 0.18 | 0.67 |

```
fw <- Reduce(function(a, b) inner_join(a, b, by = c("CHEM_ID", "name")), lapply(names(M), function(m)
  feat_cage |> filter(matrix == m) |> select(CHEM_ID, name, log2fc, p_mm_df6, icc) |> rename_with(~ paste0(.x, "_", m), -c(CHEM_ID, name))))
table_s8d <- conc |> select(CHEM_ID, name) |> inner_join(fw, by = c("CHEM_ID", "name")) |>
  mutate(retained_mm = p_mm_df6_CECAL < 0.05 & p_mm_df6_LIVER < 0.05 & p_mm_df6_PLASMA < 0.05) |> arrange(p_mm_df6_CECAL)
stopifnot(nrow(table_s8d) == nrow(conc))
table_s8d |> select(-CHEM_ID) |> ktab(caption = "Table S8D. The 36 concordant host metabolites under the cage-aware mixed model (p on 6 df)", digits = 3)
```

Table S8D. The 36 concordant host metabolites under the cage-aware mixed
model (p on 6 df)

| name | log2fc\_CECAL | p\_mm\_df6\_CECAL | icc\_CECAL | log2fc\_LIVER | p\_mm\_df6\_LIVER | icc\_LIVER | log2fc\_PLASMA | p\_mm\_df6\_PLASMA | icc\_PLASMA | retained\_mm |
| --- | --- | --- | --- | --- | --- | --- | --- | --- | --- | --- |
| cholesterol sulfate | -0.838 | 4.9e-05 | 0.000 | -0.237 | 0.17 | 0.836 | -0.698 | 0.00082 | 0.155 | FALSE |
| fumarate | 4.110 | 8.3e-05 | 0.000 | 1.317 | 0.00042 | 0.000 | 1.354 | 0.00046 | 0.000 | TRUE |
| gulonate\* | 2.208 | 0.0002 | 0.000 | 0.744 | 0.00052 | 0.000 | 0.772 | 0.0048 | 0.000 | TRUE |
| alpha-hydroxyisocaproate | 2.951 | 0.00051 | 0.405 | 1.705 | 0.0054 | 0.334 | 1.372 | 0.03 | 0.754 | TRUE |
| 1-palmitoyl-2-linoleoyl-GPE (16:0/18:2) | 2.223 | 0.00052 | 0.000 | 1.933 | 3.2e-05 | 0.000 | 1.957 | 7.7e-05 | 0.000 | TRUE |
| 1-stearoyl-2-arachidonoyl-GPC (18:0/20:4) | -2.606 | 0.0012 | 0.191 | -0.656 | 0.00027 | 0.182 | -0.771 | 0.011 | 0.527 | TRUE |
| alpha-tocopherol | -0.374 | 0.0014 | 0.000 | -0.427 | 0.013 | 0.077 | -0.620 | 0.0065 | 0.000 | TRUE |
| 1-(1-enyl-palmitoyl)-GPE (P-16:0)\* | -2.169 | 0.0016 | 0.000 | -0.402 | 0.075 | 0.559 | -0.179 | 0.06 | 0.421 | FALSE |
| 1-palmitoyl-2-arachidonoyl-GPC (16:0/20:4n6) | -2.187 | 0.0021 | 0.000 | -0.377 | 9.6e-05 | 0.000 | -0.585 | 0.023 | 0.418 | TRUE |
| citrate | 3.278 | 0.0026 | 0.434 | 2.453 | 0.0024 | 0.554 | 0.423 | 0.012 | 0.538 | TRUE |
| N-stearoyl-sphingosine (d18:1/18:0)\* | -0.993 | 0.0044 | 0.342 | -1.691 | 0.00079 | 0.000 | -2.044 | 0.00086 | 0.000 | TRUE |
| taurohyodeoxycholic acid | -1.776 | 0.0046 | 0.000 | -1.007 | 0.092 | 0.673 | -0.977 | 0.045 | 0.000 | FALSE |
| N-glycolylneuraminate | -0.851 | 0.0051 | 0.126 | -0.232 | 0.042 | 0.000 | -0.683 | 0.013 | 0.000 | TRUE |
| 1-linoleoyl-GPI (18:2)\* | 2.339 | 0.014 | 0.694 | 0.862 | 0.0031 | 0.000 | 0.909 | 0.00095 | 0.000 | TRUE |
| N1-methyl-2-pyridone-5-carboxamide | -2.649 | 0.015 | 0.000 | -0.764 | 0.0045 | 0.408 | -1.293 | 0.0019 | 0.478 | TRUE |
| N,N,N-trimethyl-5-aminovalerate | 1.330 | 0.018 | 0.575 | 1.651 | 9.2e-05 | 0.257 | 1.536 | 0.0028 | 0.562 | TRUE |
| pseudouridine | 1.294 | 0.018 | 0.362 | 0.259 | 0.057 | 0.000 | 0.266 | 0.024 | 0.000 | FALSE |
| docosapentaenoate (n3 DPA; 22:5n3) | 1.146 | 0.021 | 0.000 | 1.777 | 0.00057 | 0.000 | 1.517 | 0.0031 | 0.000 | TRUE |
| trimethylamine N-oxide | 1.520 | 0.022 | 0.932 | 0.921 | 0.065 | 0.479 | 0.859 | 0.044 | 0.000 | FALSE |
| linolenate [alpha or gamma; (18:3n3 or 6)] | 1.504 | 0.023 | 0.000 | 0.983 | 0.022 | 0.000 | 2.058 | 0.00091 | 0.657 | TRUE |
| 2-hydroxy-3-methylvalerate | 1.899 | 0.023 | 0.852 | 0.802 | 0.0048 | 0.108 | 0.392 | 0.055 | 0.000 | FALSE |
| 1-stearoyl-2-oleoyl-GPC (18:0/18:1) | -1.398 | 0.025 | 0.000 | -0.800 | 0.00015 | 0.000 | -0.507 | 0.072 | 0.341 | FALSE |
| glucuronate | 0.488 | 0.029 | 0.000 | 0.487 | 0.011 | 0.438 | 0.406 | 0.023 | 0.811 | TRUE |
| eicosapentaenoate (EPA; 20:5n3) | 1.216 | 0.032 | 0.156 | 2.667 | 7.7e-05 | 0.000 | 2.225 | 0.00013 | 0.209 | TRUE |
| 1-arachidonoyl-GPC (20:4n6)\* | -1.379 | 0.034 | 0.709 | -0.851 | 8.2e-05 | 0.000 | -0.957 | 3.6e-05 | 0.000 | TRUE |
| lactate | 2.065 | 0.035 | 0.382 | 0.311 | 0.047 | 0.375 | 0.362 | 0.0012 | 0.000 | TRUE |
| N2-acetyllysine | 0.496 | 0.044 | 0.000 | 0.899 | 0.00076 | 0.000 | 0.408 | 0.067 | 0.000 | FALSE |
| myo-inositol | 0.365 | 0.047 | 0.000 | 0.937 | 0.00034 | 0.000 | 1.025 | 0.016 | 0.498 | TRUE |
| indolelactate | -2.310 | 0.05 | 0.000 | -0.722 | 0.0048 | 0.000 | -0.928 | 0.023 | 0.547 | FALSE |
| N6-acetyllysine | 0.587 | 0.054 | 0.574 | 0.455 | 0.0042 | 0.208 | 0.667 | 0.0001 | 0.199 | FALSE |
| adenine | -1.974 | 0.058 | 0.842 | -0.376 | 0.12 | 0.823 | -1.605 | 0.0017 | 0.000 | FALSE |
| 1-palmitoyl-2-oleoyl-GPC (16:0/18:1) | -1.189 | 0.06 | 0.000 | -0.275 | 0.0004 | 0.000 | -0.463 | 0.031 | 0.428 | FALSE |
| 2-O-methylascorbic acid | -1.616 | 0.086 | 0.400 | -1.011 | 7.2e-05 | 0.000 | -0.863 | 0.00048 | 0.194 | FALSE |
| N-acetylglutamine | 0.738 | 0.13 | 0.679 | 0.664 | 0.033 | 0.213 | 0.987 | 0.0015 | 0.000 | FALSE |
| 1,2-dilinoleoyl-GPE (18:2/18:2)\* | 0.913 | 0.2 | 0.977 | 2.417 | 0.00013 | 0.000 | 2.590 | 0.024 | 0.895 | FALSE |
| 1-linoleoyl-GPE (18:2)\* | 1.473 | 0.38 | 0.907 | 1.555 | 6.4e-05 | 0.000 | 0.631 | 0.048 | 0.000 | FALSE |

```
write_xlsx(list(S8A_composites_MM_LMM = table_s8a, S8B_class_LOCO = table_s8b, S8C_feature_summary = table_s8c, S8D_concordant_36 = table_s8d |> select(-CHEM_ID),
                per_feature_cage = feat_cage), file.path(out_dir, "tables", "Table_S8_cage_sensitivity.xlsx"))
d9 <- lapply(names(M), function(m) feat_cage |> filter(matrix == m) |>
  transmute(CHEM_ID, CHEMICAL_NAME = name, log2FC_bean_vs_control = log2fc, Welch_p = p_welch, Welch_FDR = q_welch, MM_LMM_b_diet = b_mm, MM_LMM_SE = se_mm,
            MM_LMM_p_6df = p_mm_df6, MM_LMM_FDR = q_mm_df6, cage_variance = s2_cage, residual_variance = s2_resid, ICC = icc) |>
  arrange(MM_LMM_p_6df)) |> setNames(paste0("Cage_", names(M)))
write_xlsx(c(d9, list(S8A_composites_MM_LMM = table_s8a, S8B_class_LOCO = table_s8b, S8C_feature_summary = table_s8c, S8D_concordant_36 = table_s8d |> select(-CHEM_ID))),
           file.path(out_dir, "tables", "COSU-01-23VW_cage_sensitivity_DataS9.xlsx"))  # Data S9
s8 <- function(cl, m, mo, col = "p_mm_df6") table_s8a[[col]][table_s8a$class == cl & table_s8a$compartment == mats[[m]] & table_s8a$model == mo]
s8b_ <- function(cl, mo, col = "loco_p_max") table_s8b[[col]][table_s8b$class == cl & table_s8b$model == mo]
```

Under the mixed model with 6 denominator degrees of freedom, 262,
252, 133 host features met FDR < 0.05 in cecal content, liver, and
plasma, against 401, 352, 235 by Welch’s test (65%, 72%, 57% retained;
every retained feature was in the Welch set). The cage variance was
estimated at zero for 35%, 54%, 35% of host features (median intraclass
correlation 0.26, 0.00, 0.18; 90th percentile 0.75, 0.59, 0.67). Of the
36 concordant metabolites, 20 kept p < 0.05 in all three
compartments. For the composites under M4, the cecal and hepatic
branched-chain terms had p = 0.009 and 0.003 (leave-one-cage-out maxima
0.116 and 0.067), the hepatic glutathione term p = 0.047 (maximum
0.967), and the plasma and hepatic n-3 terms p = 0.010 and 0.052 (maxima
0.079 and 0.643). Class-level leave-one-cage-out maxima under M4 were
0.055 (branched-chain), 0.961 (glutathione-related), 0.195 (n-3), 0.279
(ketone/acylcarnitine), 0.601 (ceramides), and 0.968 (bile acids).

# 18 Session information

```
sessionInfo()
```

```
## R version 4.6.1 (2026-06-24 ucrt)
## Platform: x86_64-w64-mingw32/x64
## Running under: Windows 11 x64 (build 26100)
## 
## Matrix products: default
##   LAPACK version 3.12.1
## 
## locale:
## [1] LC_COLLATE=English_United States.utf8 
## [2] LC_CTYPE=English_United States.utf8   
## [3] LC_MONETARY=English_United States.utf8
## [4] LC_NUMERIC=C                          
## [5] LC_TIME=English_United States.utf8    
## 
## time zone: America/Denver
## tzcode source: internal
## 
## attached base packages:
## [1] stats     graphics  grDevices utils     datasets  methods   base     
## 
## other attached packages:
##  [1] lmtest_0.9-40     zoo_1.9-1         globaltest_5.66.0 survival_3.8-6   
##  [5] limma_3.68.5      kableExtra_1.4.1  knitr_1.52        scales_1.4.0     
##  [9] patchwork_1.3.2   ggplot2_4.0.3     stringr_1.6.0     tibble_3.3.1     
## [13] tidyr_1.3.2       dplyr_1.2.1       writexl_2.0.1     readxl_1.5.0.1   
## 
## loaded via a namespace (and not attached):
##  [1] KEGGREST_1.51.1      gtable_0.3.6         xfun_0.61           
##  [4] bslib_0.12.0         Biobase_2.72.0       lattice_0.22-9      
##  [7] vctrs_0.7.3          tools_4.6.1          generics_0.1.4      
## [10] stats4_4.6.1         AnnotationDbi_1.74.0 RSQLite_3.53.3      
## [13] blob_1.3.0           pkgconfig_2.0.3      Matrix_1.7-5        
## [16] RColorBrewer_1.1-3   S4Vectors_0.50.3     S7_0.2.2            
## [19] RcppParallel_6.2.1   lifecycle_1.0.5      compiler_4.6.1      
## [22] farver_2.1.2         Biostrings_2.80.2    textshaping_1.0.5   
## [25] statmod_1.5.2        Seqinfo_1.2.0        htmltools_0.5.9     
## [28] sass_0.4.10          yaml_2.3.12          crayon_1.5.3        
## [31] pillar_1.11.1        jquerylib_0.1.4      cachem_1.1.0        
## [34] qs2_0.3.1            tidyselect_1.2.1     digest_0.6.39       
## [37] stringi_1.8.9        purrr_1.2.2          labeling_0.4.3      
## [40] splines_4.6.1        fastmap_1.2.0        grid_4.6.1          
## [43] cli_3.6.6            magrittr_2.0.5       XML_3.99-0.25       
## [46] withr_3.0.3          bit64_4.8.6          XVector_0.52.0      
## [49] rmarkdown_2.32       httr_1.4.9           bit_4.6.0           
## [52] otel_0.2.0           cellranger_1.1.0     png_0.1-9           
## [55] stringfish_0.19.2    memoise_2.0.1        evaluate_1.0.5      
## [58] IRanges_2.46.0       viridisLite_0.4.3    rlang_1.3.0         
## [61] Rcpp_1.1.2           xtable_1.8-8         glue_1.8.1          
## [64] DBI_1.3.0            xml2_1.6.0           BiocGenerics_0.58.1 
## [67] svglite_2.2.2        rstudioapi_0.19.0    annotate_1.90.0     
## [70] jsonlite_2.0.0       R6_2.6.1             systemfonts_1.3.2
```
