# Supplementary material for: Concordant Metabolite Differences Across Cecal Content, Liver, and Plasma in Mice Fed an Obesogenic Diet Containing Common Bean (Phaseolus vulgaris L.): A Multi-Compartment Metabolomics Study
Source: Nutrients. 2026 Nov 5;18(21):3669. doi: 10.3390/nu18213669 (PMC13765519; doi:10.3390/nu18213669)
Supplement: Supplementary file 1 [file nutrients-18-03669-s001.zip › nutrients-4490632-supplementary/Supplementary_Materials_R2_vs5.pdf]

Supplementary Materials

Concordant Metabolite Differences across Cecal Content, Liver, and Plasma in Mice Fed an Obesogenic Diet Containing Common Bean (Phaseolus vulgaris L.): A Multi-Compartment Metabolomics Study

Thompson HJ, Fitzgerald VK, Lutsiv T, McGinley JN, Neil ES, Hussan H. Nutrients. All tables and figures in this document are reproduced by the Supplementary Code (an R Markdown document) from Data S1, S3, S5, S6, S7, and S8 (the per-animal phenotype and hepatic-lipid files and the genome-wide liver CPM export).

Table S1. Diet composition

Formulation of the 0% and 70% (nominal) protein-from-bean diets fed to the male cohort, and ingredient-based estimates of their amino acid and fatty-acid composition. The diets were prepared in the Cancer Prevention Laboratory from purified ingredients except for the bean and were formulated, as previously reported for this study, to nominally equal macronutrient proportions weight by weight, from ingredient and supplier values (58.6% carbohydrate, 20% protein, 15.5% fat, 5.9% micronutrients; AIN-93G basis; 32.5% of energy from fat). The finished diets were not assayed. The formulation and the estimates, with their sources, are in the diet workbook (Data S5).

A. Formulation, g/100 g diet (source: CSU-236-237-239 diet formulation record)

Ingredient	0% bean	70% bean
Cooked whole bean powder (white kidney)	0	40.8
Casein (acid, ≥85% protein)	23.5	7.1
Corn starch (regular; resistant starch unavailable)	15.9	2.0
Sucrose	25.0	21.0
Dextrose	7.2	7.2
Cellulose (Solka-Floc)	7.5	1.0
Corn oil	11.32	11.32
Anhydrous butter fat	4.24	4.24
AIN-93G mineral mix (Dyets 210025)	3.8	3.8
AIN-93 vitamin mix (Dyets 310025)	1.1	1.1
DL-Methionine / L-Tryptophan / choline bitartrate	0.3 / 0 / 0.2	0.3 / 0.06 / 0.2

B. Ingredient-based estimates of amino acid and fatty-acid supply (not assayed; published composition data for casein, cooked white bean, corn oil, and milk fat; Data S5), with the NRC (1995) requirement for the mouse

Component	0% bean	70% bean	70% / 0%	NRC requirement	70% diet / requirement
Valine, g/kg diet	13.2	9.3	0.71	5.0	1.9
Leucine, g/kg	18.4	13.7	0.74	7.0	2.0
Isoleucine, g/kg	10.0	7.5	0.75	4.0	1.9
Total branched-chain amino acids (BCAA), g/kg	41.5	30.5	0.73	16.0	1.9
Methionine + cysteine, g/kg (incl. 3 g/kg DL-methionine)	9.4	7.1	0.75	5.0	1.4
Lysine, g/kg	15.6	11.7	0.75	4.0	2.9
Threonine, g/kg	8.4	6.6	0.79	4.0	1.7
Tryptophan, g/kg	2.6	2.5	0.97	1.0	2.5
Phenylalanine + tyrosine, g/kg	21.2	14.9	0.70	7.6	2.0
Histidine, g/kg	5.8	4.6	0.80	2.0	2.3
Arginine, g/kg	7.2	8.1	1.12	3.0	2.7

Component	0% bean	70% bean	70% / 0%	NRC requirement	70% diet / requirement
Protein implied by these inputs, g/100 g diet	20.3	16.6	0.82	—	—
Linoleate 18:2n-6, g/100 g diet	6.12	6.23	1.02	—	—
α-Linolenate 18:3n-3, g/100 g diet	0.150	0.231	1.54	—	—
Preformed EPA / DHA / arachidonate	none	none	—	—	—
Cellulose / bean powder, g/100 g diet (formulation)	7.5 / 0	1.0 / 40.8	—	—	—

Table S1. *The bean ingredient was white kidney bean cooked as a whole food with its leachate, freeze-dried, and milled; it replaced the corresponding amounts of casein, corn starch, cellulose, and sucrose (L-tryptophan, 0.06 g/100 g, was added), with the bean entered at 40 g protein/100 g from the Nutrition Facts label of the canned product expressed on a dry-weight basis, and the 0% and 70% designations are nominal percentages of dietary protein from bean. Resistant corn starch was unavailable at formulation and regular corn starch was used in both diets. Part B values are estimates: formulation × ingredient protein or fat fraction × published profile (casein 85% protein; bean powder 25.0 g protein and 0.9 g fat per 100 g, from USDA FoodData Central SR Legacy NDB 16050 on a dry-matter basis at an assumed 5% residual moisture; amino acid profiles from Gorissen et al. 2018 for casein and USDA 16050 for cooked white bean; fatty-acid profiles from USDA 04518, 01003, and 16050). Both the label-derived and the published protein values rest on the nitrogen-to-protein factor 6.25; they differ because the label conversion omitted fiber and ash from the dry weight, and the estimates therefore place the amino acid supply of the 70% diet below that of the 0% diet (implied protein 16.6 vs. 20.3 g/100 g). NRC requirements are those of the National Research Council (Nutrient Requirements of Laboratory Animals, 4th rev. ed., 1995) for the growing mouse, with sulfur and aromatic amino acids as pairs; both diets exceed every requirement, the narrowest margin being methionine + cysteine in the 70% diet. The absolute dietary n-6:n-3 ratio (about 41 in the control and 27 in the bean diet under the profiles used) varies roughly two-fold across plausible corn-oil α-linolenate specifications (Data S5, part F) and is not interpreted; the direction, higher α-linolenate with unchanged linoleate in the bean diet, holds under every assumption.*

Table S2. Differential-abundance summary

Matrix	Metabolites tested	p < 0.05 (Welch)	FDR < 0.05 (Welch)	FDR < 0.05 (Mann–Whitney)	Both tests	Welch only	MW only
Cecal content	959	574	531	517	503	28	14
Liver	1065	487	412	412	386	26	26
Plasma	1019	389	270	264	245	25	19

Table S2. *Counts of tested metabolites (named plus unnamed) and of those differing [tested = detected in at least one of the 14 male samples and not constant across them; Section 3.1] between bean and control by Welch's t-test on $\log_2$ Pareto-scaled values and by the two-sided Mann–Whitney U test (exact without ties; normal approximation with tie and continuity correction otherwise), each with Benjamini–Hochberg FDR within matrix; agreement columns give the number of features significant by both, by Welch only, and by Mann–Whitney only at FDR < 0.05. With 7 vs. 7 observations the minimum attainable Mann–Whitney p-value is 5.8×10^{-4}. Missing values before imputation: features with any missing value 320 / 228 / 310, and features missing in more than half of samples 55 / 13 / 41 (named: 50 / 12 / 32), for cecal content / liver / plasma; per-feature counts are in Data S1. Complete per-metabolite results (fold change (FC), both tests, FDR, pathway annotation, exposure-marker flag with rule, missingness) are in Data S1.*

Table S3. Host metabolites concordant across all three compartments

Metabolite	Super-pathway	Cecal	Liver	Plasma
2-hydroxy-3-methylvalerate	Amino Acid	+1.90	+0.80	+0.39
N,N,N-trimethyl-5-aminovalerate †	Amino Acid	+1.33	+1.65	+1.54
N-acetylglutamine †	Amino Acid	+0.74	+0.66	+0.99
N2-acetyllysine	Amino Acid	+0.50	+0.90	+0.41
N6-acetyllysine †	Amino Acid	+0.59	+0.45	+0.67
alpha-hydroxyisocaproate †	Amino Acid	+2.95	+1.70	+1.37
indolelactate	Amino Acid	-2.31	-0.72	-0.93
N-glycolylneuraminate	Carbohydrate	-0.85	-0.23	-0.68
glucuronate †	Carbohydrate	+0.49	+0.49	+0.41
lactate †	Carbohydrate	+2.06	+0.31	+0.36

Metabolite	Super-pathway	Cecal	Liver	Plasma
2-O-methylascorbic acid	Cofactors and Vitamins	-1.62	-1.01	-0.86
N1-methyl-2-pyridone-5-carboxamide †	Cofactors and Vitamins	-2.65	-0.76	-1.29
alpha-tocopherol †	Cofactors and Vitamins	-0.37	-0.43	-0.62
gulonate* †	Cofactors and Vitamins	+2.21	+0.74	+0.77
citrate †	Energy	+3.28	+2.45	+0.42
fumarate †	Energy	+4.11	+1.32	+1.35
1,2-dilinoleoyl-GPE (18:2/18:2)* †	Lipid	+0.91	+2.42	+2.59
1-(1-enyl-palmitoyl)-GPE (P-16:0)*	Lipid	-2.17	-0.40	-0.18
1-arachidonoyl-GPC (20:4n6)* †	Lipid	-1.38	-0.85	-0.96
1-linoleoyl-GPE (18:2)*	Lipid	+1.47	+1.56	+0.63
1-linoleoyl-GPI (18:2)* †	Lipid	+2.34	+0.86	+0.91
1-palmitoyl-2-arachidonoyl-GPC (16:0/20:4n6) †	Lipid	-2.19	-0.38	-0.58
1-palmitoyl-2-linoleoyl-GPE (16:0/18:2) †	Lipid	+2.22	+1.93	+1.96
1-palmitoyl-2-oleoyl-GPC (16:0/18:1)	Lipid	-1.19	-0.28	-0.46
1-stearoyl-2-arachidonoyl-GPC (18:0/20:4) †	Lipid	-2.61	-0.66	-0.77
1-stearoyl-2-oleoyl-GPC (18:0/18:1) †	Lipid	-1.40	-0.80	-0.51
N-stearoyl-sphingosine (d18:1/18:0)* †	Lipid	-0.99	-1.69	-2.04
cholesterol sulfate	Lipid	-0.84	-0.24	-0.70
docosapentaenoate (n3 DPA; 22:5n3) †	Lipid	+1.15	+1.78	+1.52
eicosapentaenoate (EPA; 20:5n3) †	Lipid	+1.22	+2.67	+2.22
linolenate [alpha or gamma; (18:3n3 or 6)] †	Lipid	+1.50	+0.98	+2.06
myo-inositol	Lipid	+0.37	+0.94	+1.02
taurohyodeoxycholic acid	Lipid	-1.78	-1.01	-0.98
trimethylamine N-oxide	Lipid	+1.52	+0.92	+0.86
adenine †	Nucleotide	-1.97	-0.38	-1.60
pseudouridine	Nucleotide	+1.29	+0.26	+0.27

Table S3. *The 36 host metabolites that differed at $p < 0.05$ (Welch) and in the same direction in cecal content, liver, and plasma, with diet-exposure markers excluded (Section 2.4; Data S1). Values are log_2FC (bean/control); positive = higher with bean. † FDR < 0.05 in all three matrices (23 metabolites). α-Hydroxyisocaproate was below detection in 5 of 7 control liver pools (minimum-imputed).*

Table S4. Adiposity phenotype, depot principal components, and multivariate adjustment of the metabolite classes for adiposity

Table S4A. Adiposity phenotype of the 14 profiled pools (two-donor values) and principal components of the four depot masses.

Two-sample Hotelling's T^2 on the four depot masses (n = 7 per group): $T^2 = 21.39$, $F(4,9) = 4.01$, p-value = 0.039.

Phenotype	Control mean	Bean mean	% difference	Welch p
Body weight (g)	49.6	43.9	-11.5	0.002
Subcutaneous fat (mg)	2331.3	1588.4	-31.9	0.013
Mesenteric fat (mg)	1168.9	749.5	-35.9	0.013
Retroperitoneal fat (mg)	495.9	529.9	6.9	0.368
Epididymal fat (mg)	1790.4	2167.4	21.1	0.023
Total visceral fat (mg)	3455.1	3446.9	-0.2	0.97
Total depot mass (mg)	5786.4	5035.2	-13.0	0.109
Hepatic lipid (mg/g dry)	415.6	125.9	-69.7	9.5×10^{-5}

Component	Variance explained (%)	Subcutaneous	Mesenteric	Retroperitoneal	Epididymal	Control mean score	Bean mean score	Welch p (diet)	r with body weight	r with total depot mass	r with hepatic lipid
PC1	54.9	0.55	0.6	-0.34	-0.47	1.09	-1.09	0.002	0.83	0.6	0.91
PC2	30.9	0.46	0.34	0.67	0.47	0.06	-0.06	0.847	0.41	0.76	0.16
PC3	10.7	0.17	0.05	-0.66	0.73	-0.12	0.12	0.518	-0.09	0.24	-0.22
PC4	3.5	-0.68	0.73	0.01	0.12	-0.05	0.05	0.627	0.09	-0.05	-0.06

Table S4B. Class-level MANCOVA: response = the class composite in cecal content, liver, and plasma (three responses); diet term under each model.

Class	Model	Predictors	Hotelling–Lawley trace	F	df	p (diet)	Leave-one-pool-out max p	Covariate p
Branched-chain amino acids	M0	Diet only	20.85	69.49	(3, 10)	5.3×10^{-7}	3.4×10^{-6}	—
Branched-chain amino acids	M6	Diet + total depot mass	28.45	85.35	(3, 9)	6.2×10^{-7}	4.2×10^{-6}	total depot mass 0.147
Branched-chain amino acids	M7	Diet + depot PC2	36.88	110.63	(3, 9)	2×10^{-7}	1.6×10^{-6}	depot PC2 0.056
Branched-chain amino acids	M1	Diet + depot PC1	8.62	25.85	(3, 9)	9.3×10^{-5}	0.002	depot PC1 0.070
Branched-chain amino acids	M2	Diet + depot PC1 + PC2	14.97	39.93	(3, 8)	3.7×10^{-5}	0.00086	depot PC1 0.095; depot PC2 0.078
Branched-chain amino acids	M3	Diet + hepatic lipid	4.90	14.71	(3, 9)	0.00081	0.006	hepatic lipid 0.061
Branched-chain amino acids	M4	Diet + depot PC1 + hepatic lipid	4.79	12.77	(3, 8)	0.002; q-value = 0.012	0.011	depot PC1 0.193; hepatic lipid 0.173
Branched-chain amino acids	M8	Diet + total depot mass + hepatic lipid	6.8	18.09	(3, 8)	0.00063	0.008	total depot mass 0.147; hepatic lipid 0.067
Branched-chain amino acids	M5	Diet + subcutaneous fat	14.07	42.21	(3, 9)	1.3×10^{-5}	0.001	subcutaneous fat 0.484
n-3 polyunsaturated fatty acids	M0	Diet only	7.17	23.91	(3, 10)	7×10^{-5}	0.00027	—
n-3 polyunsaturated fatty acids	M6	Diet + total depot mass	5.49	16.47	(3, 9)	0.00054	0.002	total depot mass 0.523
n-3 polyunsaturated fatty acids	M7	Diet + depot PC2	7.18	21.54	(3, 9)	0.00019	0.00077	depot PC2 0.542
n-3 polyunsaturated fatty acids	M1	Diet + depot PC1	2.43	7.28	(3, 9)	0.009	0.016	depot PC1 0.362
n-3 polyunsaturated fatty acids	M2	Diet + depot PC1 + PC2	2.41	6.44	(3, 8)	0.016	0.028	depot PC1 0.418; depot PC2 0.594
n-3 polyunsaturated fatty acids	M3	Diet + hepatic lipid	1.26	3.77	(3, 9)	0.053	0.276	hepatic lipid 0.959
n-3 polyunsaturated fatty acids	M4	Diet + depot PC1 + hepatic lipid	2.05	5.47	(3, 8)	0.024; q-value = 0.049	0.067	depot PC1 0.194; hepatic lipid 0.545
n-3 polyunsaturated fatty acids	M8	Diet + total depot mass + hepatic lipid	1.44	3.84	(3, 8)	0.057	0.378	total depot mass 0.552; hepatic lipid 0.947
n-3 polyunsaturated fatty acids	M5	Diet + subcutaneous fat	3.65	10.96	(3, 9)	0.002	0.007	subcutaneous fat 0.228
Glutathione-related	M0	Diet only	10.04	33.47	(3, 10)	1.6×10^{-5}	7.2×10^{-5}	—
Glutathione-related	M6	Diet + total depot mass	12.81	38.43	(3, 9)	1.9×10^{-5}	0.0001	total depot mass 0.019

Class	Model	Predictors	Hotelling–Lawley trace	F	df	p (diet)	Leave-one-pool-out max p	Covariate p
Glutathione-related	M7	Diet + depot PC2	11.71	35.12	(3, 9)	2.7×10^{-5}	0.00013	depot PC2 0.272
Glutathione-related	M1	Diet + depot PC1	3.64	10.93	(3, 9)	0.002	0.009	depot PC1 0.412
Glutathione-related	M2	Diet + depot PC1 + PC2	4.44	11.84	(3, 8)	0.003	0.01	depot PC1 0.361; depot PC2 0.248
Glutathione-related	M3	Diet + hepatic lipid	1.76	5.29	(3, 9)	0.022	0.136	hepatic lipid 0.915
Glutathione-related	M4	Diet + depot PC1 + hepatic lipid	2.43	6.47	(3, 8)	0.016; q-value = 0.047	0.101	depot PC1 0.382; hepatic lipid 0.811
Glutathione-related	M8	Diet + total depot mass + hepatic lipid	5.1	13.65	(3, 8)	0.002	0.021	total depot mass 0.018; hepatic lipid 0.639
Glutathione-related	M5	Diet + subcutaneous fat	7.0	21.01	(3, 9)	0.00021	0.005	subcutaneous fat 0.027
Ceramides	M0	Diet only	5.54	18.46	(3, 10)	0.00021	0.00074	—
Ceramides	M6	Diet + total depot mass	4.67	14.0	(3, 9)	0.00097	0.003	total depot mass 0.101
Ceramides	M7	Diet + depot PC2	6.02	18.05	(3, 9)	0.00038	0.001	depot PC2 0.517
Ceramides	M1	Diet + depot PC1	1.83	5.5	(3, 9)	0.02	0.073	depot PC1 0.528
Ceramides	M2	Diet + depot PC1 + PC2	1.95	5.2	(3, 8)	0.028	0.088	depot PC1 0.382; depot PC2 0.374
Ceramides	M3	Diet + hepatic lipid	0.74	2.2	(3, 9)	0.157	0.616	hepatic lipid 0.590
Ceramides	M4	Diet + depot PC1 + hepatic lipid	0.72	1.93	(3, 8)	0.203; q-value = 0.20	0.662	depot PC1 0.915; hepatic lipid 0.970
Ceramides	M8	Diet + total depot mass + hepatic lipid	0.99	2.63	(3, 8)	0.122	0.637	total depot mass 0.254; hepatic lipid 0.975
Ceramides	M5	Diet + subcutaneous fat	3.07	9.2	(3, 9)	0.004	0.102	subcutaneous fat 0.082
Bile acids	M0	Diet only	1.21	4.04	(3, 10)	0.04	0.081	—
Bile acids	M6	Diet + total depot mass	0.84	2.53	(3, 9)	0.123	0.259	total depot mass 0.190
Bile acids	M7	Diet + depot PC2	1.22	3.65	(3, 9)	0.057	0.112	depot PC2 0.845
Bile acids	M1	Diet + depot PC1	1.0	2.99	(3, 9)	0.088	0.291	depot PC1 0.062
Bile acids	M2	Diet + depot PC1 + PC2	1.01	2.69	(3, 8)	0.117	0.35	depot PC1 0.072; depot PC2 0.756
Bile acids	M3	Diet + hepatic lipid	1.06	3.17	(3, 9)	0.078	0.294	hepatic lipid 0.091
Bile acids	M4	Diet + depot PC1 + hepatic lipid	0.97	2.58	(3, 8)	0.126; q-value = 0.15	0.48	depot PC1 0.613; hepatic lipid 0.792
Bile acids	M8	Diet + total depot mass + hepatic lipid	0.97	2.6	(3, 8)	0.125	0.410	total depot mass 0.508; hepatic lipid 0.278
Bile acids	M5	Diet + subcutaneous fat	0.87	2.61	(3, 9)	0.116	0.294	subcutaneous fat 0.143
Ketone bodies / acylcarnitines	M0	Diet only	1.51	5.05	(3, 10)	0.022	0.045	—
Ketone bodies / acylcarnitines	M6	Diet + total depot mass	4.88	14.64	(3, 9)	0.00083	0.012	total depot mass 0.015
Ketone bodies / acylcarnitines	M7	Diet + depot PC2	1.88	5.65	(3, 9)	0.019	0.044	depot PC2 0.185
Ketone bodies / acylcarnitines	M1	Diet + depot PC1	3.02	9.05	(3, 9)	0.004	0.063	depot PC1 0.134
Ketone bodies / acylcarnitines	M2	Diet + depot PC1 + PC2	4.56	12.15	(3, 8)	0.002	0.079	depot PC1 0.084; depot PC2 0.112
Ketone bodies / acylcarnitines	M3	Diet + hepatic lipid	1.24	3.73	(3, 9)	0.054	0.221	hepatic lipid 0.486
Ketone bodies / acylcarnitines	M4	Diet + depot PC1 + hepatic lipid	1.27	3.40	(3, 8)	0.074; q-value = 0.11	0.234	depot PC1 0.185; hepatic lipid 0.569
Ketone bodies / acylcarnitines	M8	Diet + total depot mass + hepatic lipid	1.54	4.10	(3, 8)	0.049	0.216	total depot mass 0.048; hepatic lipid 0.877
Ketone bodies / acylcarnitines	M5	Diet + subcutaneous fat	5.88	17.65	(3, 9)	0.00041	0.018	subcutaneous fat 0.016

Table S4C. Compartment-level MANCOVA: response = the six composites measured in one compartment (six responses); diet term under each model (donor-correct phenotypes). These models have six responses on 14 observations, are near-saturated, and are descriptive only.

Compartment	Model	Predictors	Hotelling–Lawley trace	F	df	p (diet)	Leave-one-pool-out max p	Covariate p	Note
Cecal	M0	Diet only	18.32	21.38	(6, 7)	0.00036	0.002	—	
Cecal	M6	Diet + total depot mass	14.57	14.57	(6, 6)	0.002	0.015	total depot mass 0.043	

Compartment	Model	Predictors	Hotelling–Lawley trace	F	df	p (diet)	Leave-one-pool-out max p	Covariate p	Note
Cecal	M7	Diet + depot PC2	18.39	18.39	(6, 6)	0.001	0.005	depot PC2 0.134	
Cecal	M1	Diet + depot PC1	7.42	7.42	(6, 6)	0.014	0.07	depot PC1 0.812	
Cecal	M2	Diet + depot PC1 + PC2	7.5	6.25	(6, 5)	0.031	0.226	depot PC1 0.817; depot PC2 0.185	
Cecal	M3	Diet + hepatic lipid	3.84	3.84	(6, 6)	0.063	0.235	hepatic lipid 0.374	
Cecal	M4	Diet + depot PC1 + hepatic lipid	3.85	3.21	(6, 5)	0.111	0.328	depot PC1 0.534; hepatic lipid 0.235	
Cecal	M8	Diet + total depot mass + hepatic lipid	3.68	3.1	(6, 5)	0.120	0.443	total depot mass 0.035; hepatic lipid 0.239	
Cecal	M5	Diet + subcutaneous fat	10.57	10.57	(6, 6)	0.006	0.135	subcutaneous fat 0.215	
Liver	M0	Diet only	27.6	32.19	(6, 7)	9.4×10^{-5}	0.00051	—	
Liver	M6	Diet + total depot mass	23.85	23.85	(6, 6)	0.00061	0.003	total depot mass 0.071	
Liver	M7	Diet + depot PC2	33.59	33.59	(6, 6)	0.00023	0.001	depot PC2 0.648	
Liver	M1	Diet + depot PC1	31.72	31.72	(6, 6)	0.00027	0.002	depot PC1 0.041	
Liver	M2	Diet + depot PC1 + PC2	32.06	26.71	(6, 5)	0.001	0.008	depot PC1 0.041; depot PC2 0.481	
Liver	M3	Diet + hepatic lipid	9.87	9.87	(6, 6)	0.007	0.04	hepatic lipid 0.336	
Liver	M4	Diet + depot PC1 + hepatic lipid	15.84	13.20	(6, 5)	0.006	0.037	depot PC1 0.333; hepatic lipid 0.974	
Liver	M8	Diet + total depot mass + hepatic lipid	13.4	11.16	(6, 5)	0.009	0.042	total depot mass 0.133; hepatic lipid 0.460	
Liver	M5	Diet + subcutaneous fat	15.09	15.09	(6, 6)	0.002	0.011	subcutaneous fat 0.012	
Plasma	M0	Diet only	15.95	18.6	(6, 7)	0.00056	0.002	—	
Plasma	M6	Diet + total depot mass	19.62	19.62	(6, 6)	0.001	0.005	total depot mass 0.069	
Plasma	M7	Diet + depot PC2	15.86	15.86	(6, 6)	0.002	0.008	depot PC2 0.040	
Plasma	M1	Diet + depot PC1	7.0	7.0	(6, 6)	0.016	0.049	depot PC1 0.721	
Plasma	M2	Diet + depot PC1 + PC2	6.9	5.75	(6, 5)	0.037	0.094	depot PC1 0.781; depot PC2 0.076	
Plasma	M3	Diet + hepatic lipid	5.34	5.34	(6, 6)	0.031	0.106	hepatic lipid 0.877	
Plasma	M4	Diet + depot PC1 + hepatic lipid	51.17	42.64	(6, 5)	0.00039	0.007	depot PC1 0.013; hepatic lipid 0.018	near-saturated fit; not interpreted
Plasma	M8	Diet + total depot mass + hepatic lipid	9.24	7.70	(6, 5)	0.020	0.086	total depot mass 0.128; hepatic lipid 0.930	
Plasma	M5	Diet + subcutaneous fat	10.77	10.77	(6, 6)	0.005	0.03	subcutaneous fat 0.342	

Table S4D. Per-compartment diet coefficient for each class under each class-level model (ordinary least squares on the compartment composite with the same predictors).

Class	Model	Compartment	Composite	b (diet)	SE	p	Leave-one-pool-out max p
Branched-chain amino acids	M0	Cecal	BCAA ketoacid/hydroxy-acid	2.05	0.16	2.7×10^{-8}	1.7×10^{-7}
Branched-chain amino acids	M0	Liver	BCAA-catabolite	1.3	0.13	4.1×10^{-7}	2×10^{-6}
Branched-chain amino acids	M0	Plasma	BCAA-parent	-0.57	0.25	0.037	0.074
Branched-chain amino acids	M6	Cecal	BCAA ketoacid/hydroxy-acid	2.11	0.19	2.2×10^{-7}	1.9×10^{-6}
Branched-chain amino acids	M6	Liver	BCAA-catabolite	1.39	0.14	9.2×10^{-7}	2.9×10^{-5}
Branched-chain amino acids	M6	Plasma	BCAA-parent	-0.72	0.27	0.024	0.055
Branched-chain amino acids	M7	Cecal	BCAA ketoacid/hydroxy-acid	2.06	0.16	6.8×10^{-8}	4.3×10^{-7}
Branched-chain amino acids	M7	Liver	BCAA-catabolite	1.3	0.14	1.1×10^{-6}	6.4×10^{-6}
Branched-chain amino acids	M7	Plasma	BCAA-parent	-0.6	0.22	0.018	0.044
Branched-chain amino acids	M1	Cecal	BCAA ketoacid/hydroxy-acid	1.81	0.24	1.3×10^{-5}	0.00043

Class	Model	Compartment	Composite	b (diet)	SE	p	Leave-one-pool-out max p
Branched-chain amino acids	M1	Liver	BCAA-catabolite	1.54	0.19	5.8×10^{-6}	0.00012
Branched-chain amino acids	M1	Plasma	BCAA-parent	-0.39	0.39	0.338	0.895
Branched-chain amino acids	M2	Cecal	BCAA ketoacid/hydroxy-acid	1.83	0.25	2.3×10^{-5}	0.001
Branched-chain amino acids	M2	Liver	BCAA-catabolite	1.55	0.2	1.3×10^{-5}	0.0003
Branched-chain amino acids	M2	Plasma	BCAA-parent	-0.45	0.34	0.218	0.434
Branched-chain amino acids	M3	Cecal	BCAA ketoacid/hydroxy-acid	1.53	0.32	0.00052	0.003
Branched-chain amino acids	M3	Liver	BCAA-catabolite	1.51	0.29	0.00026	0.002
Branched-chain amino acids	M3	Plasma	BCAA-parent	-0.97	0.53	0.096	0.271
Branched-chain amino acids	M4	Cecal	BCAA ketoacid/hydroxy-acid	1.52	0.34	0.001	0.006
Branched-chain amino acids	M4	Liver	BCAA-catabolite	1.42	0.28	0.00045	0.003
Branched-chain amino acids	M4	Plasma	BCAA-parent	-1.20	0.45	0.023	0.108
Branched-chain amino acids	M8	Cecal	BCAA ketoacid/hydroxy-acid	1.39	0.27	0.00050	0.010
Branched-chain amino acids	M8	Liver	BCAA-catabolite	1.44	0.29	0.0006	0.007
Branched-chain amino acids	M8	Plasma	BCAA-parent	-0.87	0.56	0.148	0.384
Branched-chain amino acids	M5	Cecal	BCAA ketoacid/hydroxy-acid	2.02	0.23	2.3×10^{-6}	0.00046
Branched-chain amino acids	M5	Liver	BCAA-catabolite	1.41	0.18	7×10^{-6}	0.002
Branched-chain amino acids	M5	Plasma	BCAA-parent	-0.76	0.33	0.045	0.115
n-3 polyunsaturated fatty acids	M0	Cecal	n-3 PUFA	1.28	0.54	0.036	0.075
n-3 polyunsaturated fatty acids	M0	Liver	n-3 PUFA	1.45	0.26	0.00011	0.00033
n-3 polyunsaturated fatty acids	M0	Plasma	n-3 PUFA	1.68	0.19	1.6×10^{-6}	6.8×10^{-6}
n-3 polyunsaturated fatty acids	M6	Cecal	n-3 PUFA	1.34	0.64	0.059	0.184
n-3 polyunsaturated fatty acids	M6	Liver	n-3 PUFA	1.23	0.27	0.00075	0.002
n-3 polyunsaturated fatty acids	M6	Plasma	n-3 PUFA	1.61	0.22	1.7×10^{-5}	0.00013
n-3 polyunsaturated fatty acids	M7	Cecal	n-3 PUFA	1.31	0.54	0.034	0.078
n-3 polyunsaturated fatty acids	M7	Liver	n-3 PUFA	1.43	0.25	0.00015	0.00049
n-3 polyunsaturated fatty acids	M7	Plasma	n-3 PUFA	1.68	0.2	4.3×10^{-6}	2×10^{-5}
n-3 polyunsaturated fatty acids	M1	Cecal	n-3 PUFA	0.46	0.81	0.581	0.819
n-3 polyunsaturated fatty acids	M1	Liver	n-3 PUFA	1.13	0.39	0.015	0.076
n-3 polyunsaturated fatty acids	M1	Plasma	n-3 PUFA	1.45	0.3	0.00049	0.003
n-3 polyunsaturated fatty acids	M2	Cecal	n-3 PUFA	0.53	0.82	0.531	0.828
n-3 polyunsaturated fatty acids	M2	Liver	n-3 PUFA	1.09	0.39	0.019	0.08
n-3 polyunsaturated fatty acids	M2	Plasma	n-3 PUFA	1.46	0.31	0.00092	0.005
n-3 polyunsaturated fatty acids	M3	Cecal	n-3 PUFA	0.97	1.21	0.436	0.977
n-3 polyunsaturated fatty acids	M3	Liver	n-3 PUFA	1.25	0.57	0.050	0.289
n-3 polyunsaturated fatty acids	M3	Plasma	n-3 PUFA	1.5	0.43	0.005	0.044
n-3 polyunsaturated fatty acids	M4	Cecal	n-3 PUFA	1.40	1.13	0.245	0.726
n-3 polyunsaturated fatty acids	M4	Liver	n-3 PUFA	1.39	0.57	0.036	0.210
n-3 polyunsaturated fatty acids	M4	Plasma	n-3 PUFA	1.6	0.44	0.004	0.036
n-3 polyunsaturated fatty acids	M8	Cecal	n-3 PUFA	0.86	1.3	0.519	0.678
n-3 polyunsaturated fatty acids	M8	Liver	n-3 PUFA	1.45	0.54	0.023	0.169
n-3 polyunsaturated fatty acids	M8	Plasma	n-3 PUFA	1.57	0.45	0.006	0.082
n-3 polyunsaturated fatty acids	M5	Cecal	n-3 PUFA	1.32	0.76	0.109	0.525
n-3 polyunsaturated fatty acids	M5	Liver	n-3 PUFA	0.98	0.29	0.006	0.023
n-3 polyunsaturated fatty acids	M5	Plasma	n-3 PUFA	1.47	0.25	0.00012	0.008
Glutathione-related	M0	Cecal	Glutathione cycle	-0.55	0.35	0.148	0.292
Glutathione-related	M0	Liver	Glutathione cycle	0.95	0.12	5×10^{-6}	2×10^{-5}

Class	Model	Compartment	Composite	b (diet)	SE	p	Leave-one-pool-out max p
Glutathione-related	M0	Plasma	Glutathione cycle	0.65	0.18	0.004	0.009
Glutathione-related	M6	Cecal	Glutathione cycle	-0.68	0.41	0.123	0.424
Glutathione-related	M6	Liver	Glutathione cycle	0.77	0.09	2.4×10^{-6}	1.3×10^{-5}
Glutathione-related	M6	Plasma	Glutathione cycle	0.68	0.21	0.009	0.024
Glutathione-related	M7	Cecal	Glutathione cycle	-0.57	0.34	0.123	0.242
Glutathione-related	M7	Liver	Glutathione cycle	0.93	0.11	2.5×10^{-6}	1.3×10^{-5}
Glutathione-related	M7	Plasma	Glutathione cycle	0.65	0.19	0.006	0.014
Glutathione-related	M1	Cecal	Glutathione cycle	-0.24	0.56	0.673	0.984
Glutathione-related	M1	Liver	Glutathione cycle	0.78	0.19	0.001	0.007
Glutathione-related	M1	Plasma	Glutathione cycle	0.73	0.29	0.029	0.092
Glutathione-related	M2	Cecal	Glutathione cycle	-0.3	0.55	0.593	0.881
Glutathione-related	M2	Liver	Glutathione cycle	0.75	0.15	0.00063	0.002
Glutathione-related	M2	Plasma	Glutathione cycle	0.73	0.31	0.038	0.12
Glutathione-related	M3	Cecal	Glutathione cycle	-0.4	0.79	0.616	0.911
Glutathione-related	M3	Liver	Glutathione cycle	0.81	0.27	0.012	0.11
Glutathione-related	M3	Plasma	Glutathione cycle	0.67	0.41	0.126	0.311
Glutathione-related	M4	Cecal	Glutathione cycle	-0.56	0.82	0.510	0.965
Glutathione-related	M4	Liver	Glutathione cycle	0.87	0.27	0.01	0.081
Glutathione-related	M4	Plasma	Glutathione cycle	0.63	0.43	0.176	0.457
Glutathione-related	M8	Cecal	Glutathione cycle	-0.24	0.81	0.774	0.951
Glutathione-related	M8	Liver	Glutathione cycle	0.98	0.16	0.00012	0.003
Glutathione-related	M8	Plasma	Glutathione cycle	0.64	0.43	0.170	0.490
Glutathione-related	M5	Cecal	Glutathione cycle	-0.78	0.48	0.134	0.704
Glutathione-related	M5	Liver	Glutathione cycle	0.66	0.11	7.6×10^{-5}	0.001
Glutathione-related	M5	Plasma	Glutathione cycle	0.68	0.25	0.021	0.08
Ceramides	M0	Cecal	Ceramide	-0.2	0.16	0.242	0.513
Ceramides	M0	Liver	Ceramide	-0.9	0.16	0.00013	0.00041
Ceramides	M0	Plasma	Ceramide	-0.84	0.18	0.00042	0.001
Ceramides	M6	Cecal	Ceramide	-0.17	0.19	0.374	0.549
Ceramides	M6	Liver	Ceramide	-0.92	0.19	0.00058	0.003
Ceramides	M6	Plasma	Ceramide	-0.63	0.15	0.002	0.004
Ceramides	M7	Cecal	Ceramide	-0.19	0.17	0.268	0.541
Ceramides	M7	Liver	Ceramide	-0.9	0.17	0.00026	0.0008
Ceramides	M7	Plasma	Ceramide	-0.83	0.16	0.00035	0.001
Ceramides	M1	Cecal	Ceramide	-0.14	0.26	0.586	0.791
Ceramides	M1	Liver	Ceramide	-0.92	0.26	0.005	0.026
Ceramides	M1	Plasma	Ceramide	-0.52	0.25	0.063	0.219
Ceramides	M2	Cecal	Ceramide	-0.14	0.27	0.618	0.826
Ceramides	M2	Liver	Ceramide	-0.92	0.28	0.008	0.034
Ceramides	M2	Plasma	Ceramide	-0.48	0.22	0.055	0.18
Ceramides	M3	Cecal	Ceramide	-0.10	0.35	0.789	0.883
Ceramides	M3	Liver	Ceramide	-0.84	0.37	0.041	0.227
Ceramides	M3	Plasma	Ceramide	-0.38	0.36	0.311	0.892
Ceramides	M4	Cecal	Ceramide	-0.10	0.38	0.798	0.920
Ceramides	M4	Liver	Ceramide	-0.82	0.39	0.063	0.280
Ceramides	M4	Plasma	Ceramide	-0.44	0.37	0.264	0.785

Class	Model	Compartment	Composite	b (diet)	SE	p	Leave-one-pool-out max p
Ceramides	M8	Cecal	Ceramide	-0.1	0.38	0.785	0.871
Ceramides	M8	Liver	Ceramide	-0.82	0.39	0.063	0.482
Ceramides	M8	Plasma	Ceramide	-0.55	0.30	0.1	0.367
Ceramides	M5	Cecal	Ceramide	-0.14	0.22	0.549	0.795
Ceramides	M5	Liver	Ceramide	-0.8	0.22	0.004	0.18
Ceramides	M5	Plasma	Ceramide	-0.5	0.19	0.023	0.058
Bile acids	M0	Cecal	Bile acid	-0.73	0.2	0.004	0.009
Bile acids	M0	Liver	Bile acid	-0.0	0.21	0.987	0.979
Bile acids	M0	Plasma	Bile acid	0.47	0.49	0.351	0.683
Bile acids	M6	Cecal	Bile acid	-0.59	0.22	0.022	0.138
Bile acids	M6	Liver	Bile acid	-0.22	0.21	0.305	0.565
Bile acids	M6	Plasma	Bile acid	0.11	0.52	0.833	0.943
Bile acids	M7	Cecal	Bile acid	-0.7	0.2	0.005	0.012
Bile acids	M7	Liver	Bile acid	-0.01	0.22	0.964	0.991
Bile acids	M7	Plasma	Bile acid	0.45	0.49	0.382	0.802
Bile acids	M1	Cecal	Bile acid	-0.5	0.32	0.132	0.266
Bile acids	M1	Liver	Bile acid	-0.62	0.25	0.029	0.116
Bile acids	M1	Plasma	Bile acid	0.26	0.78	0.743	0.981
Bile acids	M2	Cecal	Bile acid	-0.50	0.32	0.158	0.515
Bile acids	M2	Liver	Bile acid	-0.64	0.25	0.028	0.077
Bile acids	M2	Plasma	Bile acid	0.2	0.79	0.805	0.907
Bile acids	M3	Cecal	Bile acid	-0.50	0.45	0.286	0.742
Bile acids	M3	Liver	Bile acid	-0.97	0.35	0.017	0.077
Bile acids	M3	Plasma	Bile acid	0.05	1.07	0.963	0.995
Bile acids	M4	Cecal	Bile acid	-0.57	0.47	0.255	0.669
Bile acids	M4	Liver	Bile acid	-0.88	0.35	0.029	0.133
Bile acids	M4	Plasma	Bile acid	0.05	1.15	0.963	0.989
Bile acids	M8	Cecal	Bile acid	-0.6	0.45	0.192	0.56
Bile acids	M8	Liver	Bile acid	-0.88	0.35	0.030	0.150
Bile acids	M8	Plasma	Bile acid	0.38	1.06	0.729	0.901
Bile acids	M5	Cecal	Bile acid	-0.57	0.28	0.062	0.331
Bile acids	M5	Liver	Bile acid	-0.43	0.23	0.084	0.203
Bile acids	M5	Plasma	Bile acid	0.21	0.67	0.765	0.887
Ketone bodies / acylcarnitines	M0	Cecal	Ketone / FA-oxidation tone	-0.2	0.3	0.522	0.892
Ketone bodies / acylcarnitines	M0	Liver	Ketone / FA-oxidation tone	1.17	0.3	0.002	0.005
Ketone bodies / acylcarnitines	M0	Plasma	Ketone / FA-oxidation tone	0.24	0.34	0.493	0.992
Ketone bodies / acylcarnitines	M6	Cecal	Ketone / FA-oxidation tone	-0.47	0.31	0.154	0.349
Ketone bodies / acylcarnitines	M6	Liver	Ketone / FA-oxidation tone	1.46	0.29	0.00038	0.015
Ketone bodies / acylcarnitines	M6	Plasma	Ketone / FA-oxidation tone	0.01	0.37	0.982	0.981
Ketone bodies / acylcarnitines	M7	Cecal	Ketone / FA-oxidation tone	-0.23	0.28	0.433	0.767
Ketone bodies / acylcarnitines	M7	Liver	Ketone / FA-oxidation tone	1.19	0.3	0.002	0.006
Ketone bodies / acylcarnitines	M7	Plasma	Ketone / FA-oxidation tone	0.22	0.33	0.526	0.933
Ketone bodies / acylcarnitines	M1	Cecal	Ketone / FA-oxidation tone	-0.15	0.49	0.756	0.973
Ketone bodies / acylcarnitines	M1	Liver	Ketone / FA-oxidation tone	1.96	0.36	0.00021	0.006
Ketone bodies / acylcarnitines	M1	Plasma	Ketone / FA-oxidation tone	0.41	0.55	0.476	0.761
Ketone bodies / acylcarnitines	M2	Cecal	Ketone / FA-oxidation tone	-0.22	0.45	0.635	0.996

Class	Model	Compartment	Composite	b (diet)	SE	p	Leave-one-pool-out max p
Ketone bodies / acylcarnitines	M2	Liver	Ketone / FA-oxidation tone	2.0	0.34	0.00016	0.018
Ketone bodies / acylcarnitines	M2	Plasma	Ketone / FA-oxidation tone	0.34	0.53	0.534	0.952
Ketone bodies / acylcarnitines	M3	Cecal	Ketone / FA-oxidation tone	-0.64	0.66	0.353	0.636
Ketone bodies / acylcarnitines	M3	Liver	Ketone / FA-oxidation tone	1.9	0.61	0.01	0.071
Ketone bodies / acylcarnitines	M3	Plasma	Ketone / FA-oxidation tone	0.3	0.77	0.706	0.980
Ketone bodies / acylcarnitines	M4	Cecal	Ketone / FA-oxidation tone	-0.80	0.66	0.256	0.449
Ketone bodies / acylcarnitines	M4	Liver	Ketone / FA-oxidation tone	1.62	0.51	0.010	0.053
Ketone bodies / acylcarnitines	M4	Plasma	Ketone / FA-oxidation tone	0.2	0.81	0.801	0.951
Ketone bodies / acylcarnitines	M8	Cecal	Ketone / FA-oxidation tone	-0.4	0.63	0.532	0.92
Ketone bodies / acylcarnitines	M8	Liver	Ketone / FA-oxidation tone	1.68	0.59	0.017	0.102
Ketone bodies / acylcarnitines	M8	Plasma	Ketone / FA-oxidation tone	0.56	0.74	0.465	0.949
Ketone bodies / acylcarnitines	M5	Cecal	Ketone / FA-oxidation tone	-0.54	0.39	0.198	0.343
Ketone bodies / acylcarnitines	M5	Liver	Ketone / FA-oxidation tone	1.79	0.3	0.0001	0.086
Ketone bodies / acylcarnitines	M5	Plasma	Ketone / FA-oxidation tone	0.07	0.47	0.88	0.964

Table S4E. Univariate regression of each composite on diet with depot PC1 + hepatic lipid (M4) or total depot mass + hepatic lipid (M8), donor-correct phenotypes.

Compartment	Composite	Model	b (diet)	SE	p (diet)	Leave-one-pool-out max p	p covariate 1	p covariate 2
Cecal	BCAA ketoacid/hydroxy-acid	M4	1.52	0.34	0.001	0.006	0.931	0.264
Cecal	Bile acid	M4	-0.57	0.47	0.255	0.669	0.529	0.880
Cecal	Ceramide	M4	-0.10	0.38	0.798	0.920	0.973	0.874
Cecal	Glutathione cycle	M4	-0.56	0.82	0.510	0.965	0.409	0.597
Cecal	Ketone / FA-oxidation tone	M4	-0.80	0.66	0.256	0.449	0.279	0.201
Cecal	n-3 PUFA	M4	1.40	1.13	0.245	0.726	0.110	0.270
Cecal	BCAA ketoacid/hydroxy-acid	M8	1.39	0.27	0.00050	0.010	0.043	0.012
Cecal	Bile acid	M8	-0.6	0.45	0.192	0.56	0.243	0.91
Cecal	Ceramide	M8	-0.1	0.38	0.785	0.871	0.912	0.843
Cecal	Glutathione cycle	M8	-0.24	0.81	0.774	0.951	0.383	0.543
Cecal	Ketone / FA-oxidation tone	M8	-0.4	0.63	0.532	0.92	0.132	0.906
Cecal	n-3 PUFA	M8	0.86	1.3	0.519	0.678	0.701	0.672
Liver	BCAA-catabolite	M4	1.42	0.28	0.00045	0.003	0.17	0.555
Liver	Bile acid	M4	-0.88	0.35	0.029	0.133	0.248	0.305
Liver	Ceramide	M4	-0.82	0.39	0.063	0.280	0.75	0.728
Liver	Glutathione cycle	M4	0.87	0.27	0.01	0.081	0.32	0.666
Liver	Ketone / FA-oxidation tone	M4	1.62	0.51	0.010	0.053	0.031	0.373
Liver	n-3 PUFA	M4	1.39	0.57	0.036	0.210	0.277	0.537
Liver	BCAA-catabolite	M8	1.44	0.29	0.0006	0.007	0.3	0.870
Liver	Bile acid	M8	-0.88	0.35	0.030	0.150	0.258	0.053
Liver	Ceramide	M8	-0.82	0.39	0.063	0.482	0.766	0.769
Liver	Glutathione cycle	M8	0.98	0.16	0.00012	0.003	0.00086	0.168
Liver	Ketone / FA-oxidation tone	M8	1.68	0.59	0.017	0.102	0.140	0.671
Liver	n-3 PUFA	M8	1.45	0.54	0.023	0.169	0.128	0.656
Plasma	BCAA-parent	M4	-1.13	0.36	0.01	0.178	0.075	0.023
Plasma	Bile acid	M4	0.43	0.93	0.657	0.97	0.867	0.869
Plasma	Ceramide	M4	-0.64	0.31	0.063	0.441	0.285	0.7
Plasma	Glutathione cycle	M4	0.6	0.34	0.113	0.254	0.606	0.616
Plasma	Ketone / FA-oxidation tone	M4	0.16	0.65	0.809	0.973	0.540	0.571

Compartment	Composite	Model	b (diet)	SE	p (diet)	Leave-one-pool-out max p	p covariate 1	p covariate 2
Plasma	n-3 PUFA	M4	1.57	0.32	0.00066	0.009	0.118	0.307
Plasma	BCAA-parent	M8	-1.05	0.44	0.037	0.221	0.668	0.31
Plasma	Bile acid	M8	0.76	0.9	0.416	0.71	0.219	0.525
Plasma	Ceramide	M8	-0.87	0.2	0.001	0.012	0.001	0.258
Plasma	Glutathione cycle	M8	0.59	0.36	0.136	0.376	0.870	0.83
Plasma	Ketone / FA-oxidation tone	M8	0.34	0.66	0.620	0.962	0.397	0.723
Plasma	n-3 PUFA	M8	1.6	0.38	0.002	0.018	0.626	0.928

Table S4. *Part F is the univariate analysis, reported as descriptive follow-up to the class-level analysis of part B. A: pool phenotype is the mean of the two donors; Hotelling's T^2 is the two-sample test on the four-depot mean vector; principal components are of the z-scored depot masses (signs set so that control pools score positive on PC1 and all PC2 loadings are positive). B–E: multivariate analysis of covariance by general linear hypothesis on the multivariate linear model; the diet term has one degree of freedom, so the Hotelling–Lawley trace equals Pillai, Wilks, and Roy and gives an exact F with $(m, n - k - m + 1)$ degrees of freedom (m = number of responses, k = number of parameters); covariates are z-scored; the leave-one-pool-out column is the largest diet p-value obtained when each of the 14 pools is omitted in turn. In part B, q-value for model M4 is the Benjamini–Hochberg-adjusted p-value across the six classes; the six class-level diet terms under a covariate set are treated as one family, and part C is not corrected because it is descriptive. B and D use the two-donor pool phenotype for all three compartments (10 of 14 plasma composites derive from one of the two donors); C and E use the phenotype of the actual donors of each sample, with the depot components refitted on those phenotypes. The plasma M4 model (six responses, four parameters) is near-saturated and its estimates are not interpreted. D gives the ordinary-least-squares diet coefficient for each compartment composite under the predictors of the corresponding class-level model (Pareto-scaled units). Body weight is excluded from the models of parts B–E because it combines fat and lean mass and correlates 0.83 with depot PC1. Model codes M0–M8 are listed with their predictors in part B and defined in the Supplementary Code. Variance inflation factors from the design matrix: M4, 4.8 (diet), 6.0 (depot PC1), 11.6 (hepatic lipid); M8, 4.8, 1.7, 6.1; single-covariate models ≤ 2.4 for the depot covariates and 4.6 for hepatic lipid. We judge these analyses to be exploratory and associative: adiposity is a post-allocation variable, conditioning on it cannot assign the variance it shares with diet, and with 8–10 denominator degrees of freedom a non-significant adjusted diet term is a failure to separate the two, not evidence of no effect.*

Table S4F. Univariate regression of each composite on diet group and one adiposity covariate (all 18 composites), with within-group correlations

Composite	Matrix	n	Cohen d	p diet	q (18)	R^2 diet %	Unique diet %	Shared %	Unique SQ %	p diet ∣ SQ	q ∣ SQ (18)	p SQ ∣ diet	p diet ∣ BW	p diet ∣ liver lipid	p diet ∣ SQ, excl. 49	VIF	Shapiro p	B–P p
BCAA ketoacid/hydroxy-acid	Cecal	6	6.75	3.1×10^{-8}	5.6×10^{-7}	93	51	42	0	2.3×10^{-6}	4.1×10^{-5}	0.864	2.4×10^{-5}	5.2×10^{-4}	4.6×10^{-4}	1.79	0.98	0.89
BCAA-catabolite	Liver	5	5.28	1.1×10^{-6}	1.0×10^{-5}	89	58	31	1	7.0×10^{-6}	6.3×10^{-5}	0.377	1.3×10^{-5}	2.6×10^{-4}	0.002	1.79	0.62	0.50
BCAA-parent	Plasma	3	-1.25	0.037	0.061	31	34	-2	5	0.034	0.062	0.364	0.015	0.020	0.064	1.64	0.13	0.95
Bile acid	Cecal	31	-1.92	0.006	0.011	52	18	34	3	0.062	0.102	0.401	0.250	0.286	0.331	1.79	0.82	0.31
Bile acid	Liver	25	-0.01	0.987	0.987	0	19	-19	42	0.084	0.127	0.016	0.205	0.017	0.128	1.79	0.92	0.57
Bile acid	Plasma	21	0.52	0.369	0.443	7	1	6	2	0.693	0.733	0.608	0.645	0.637	0.467	1.64	0.18	0.01
Ceramide	Cecal	14	-0.66	0.246	0.317	11	3	8	1	0.549	0.617	0.696	0.779	0.789	0.627	1.79	0.65	0.34
Ceramide	Liver	13	-2.94	1.4×10^{-4}	4.9×10^{-4}	72	32	40	1	0.004	0.013	0.519	0.003	0.041	0.180	1.79	0.65	0.18
Ceramide	Plasma	17	-2.58	0.001	0.003	66	16	50	14	0.013	0.028	0.017	0.278	0.068	0.064	1.64	0.28	0.69
Glutathione cycle	Cecal	19	-0.83	0.149	0.206	17	19	-2	4	0.134	0.172	0.481	0.310	0.616	0.704	1.79	0.93	0.58
Glutathione cycle	Liver	30	4.16	2.9×10^{-5}	1.3×10^{-4}	83	22	61	10	7.6×10^{-5}	3.4×10^{-4}	0.002	0.004	0.012	0.001	1.79	0.16	0.41
Glutathione cycle	Plasma	26	1.90	0.004	0.009	51	32	19	0	0.021	0.041	0.957	0.045	0.096	0.129	1.64	0.10	0.97
Ketone / FA-oxidation tone	Cecal	3	-0.35	0.523	0.553	3	14	-11	13	0.198	0.237	0.220	0.655	0.353	0.004	1.79	0.88	0.35
Ketone / FA-oxidation tone	Liver	10	2.11	0.006	0.011	57	74	-17	20	1.0×10^{-4}	3.8×10^{-4}	0.011	5.0×10^{-4}	0.010	0.086	1.79	0.35	0.44
Ketone / FA-oxidation tone	Plasma	12	0.38	0.504	0.553	4	1	3	1	0.781	0.781	0.698	0.450	0.787	0.789	1.64	0.29	0.03
n-3 PUFA	Cecal	5	1.26	0.047	0.071	32	19	13	0	0.109	0.150	0.933	0.532	0.436	0.525	1.79	0.07	0.32
n-3 PUFA	Liver	6	3.02	5.2×10^{-4}	0.002	73	19	54	10	0.006	0.015	0.032	0.037	0.050	1.8×10^{-4}	1.79	0.40	0.20
n-3 PUFA	Plasma	5	4.66	2.5×10^{-6}	1.5×10^{-5}	86	42	44	2	6.9×10^{-5}	3.4×10^{-4}	0.258	3.4×10^{-4}	0.001	0.007	1.64	0.27	0.50

Within-group Pearson correlation of each composite with subcutaneous fat (donor-correct), r with p, n = 7 per group; last column after omitting pool 49 (n = 6):

Composite	Matrix	Control	Bean	Bean excl. pool 49
BCAA ketoacid/hydroxy-acid	Cecal	-0.28 (p = 0.54)	0.07 (p = 0.88)	0.90 (p = 0.01), n = 6
BCAA-catabolite	Liver	-0.29 (p = 0.53)	0.46 (p = 0.30)	0.70 (p = 0.12), n = 6
BCAA-parent	Plasma	-0.55 (p = 0.20)	-0.19 (p = 0.68)	-0.16 (p = 0.76), n = 6
Bile acid	Cecal	-0.04 (p = 0.93)	0.33 (p = 0.46)	0.39 (p = 0.45), n = 6
Bile acid	Liver	-0.52 (p = 0.23)	-0.75 (p = 0.05)	-0.64 (p = 0.17), n = 6
Bile acid	Plasma	0.13 (p = 0.78)	-0.18 (p = 0.70)	-0.68 (p = 0.14), n = 6
Ceramide	Cecal	0.08 (p = 0.87)	0.13 (p = 0.77)	-0.08 (p = 0.88), n = 6
Ceramide	Liver	0.71 (p = 0.08)	-0.03 (p = 0.95)	-0.53 (p = 0.28), n = 6
Ceramide	Plasma	-0.08 (p = 0.87)	0.79 (p = 0.03)	0.54 (p = 0.27), n = 6
Glutathione cycle	Cecal	-0.09 (p = 0.84)	-0.33 (p = 0.47)	0.35 (p = 0.50), n = 6
Glutathione cycle	Liver	-0.19 (p = 0.69)	-0.93 (p = 0.00)	-0.58 (p = 0.23), n = 6
Glutathione cycle	Plasma	0.13 (p = 0.78)	-0.03 (p = 0.94)	0.08 (p = 0.88), n = 6
Ketone / FA-oxidation tone	Cecal	-0.75 (p = 0.05)	-0.22 (p = 0.63)	-0.89 (p = 0.02), n = 6
Ketone / FA-oxidation tone	Liver	-0.22 (p = 0.63)	0.80 (p = 0.03)	0.14 (p = 0.79), n = 6
Ketone / FA-oxidation tone	Plasma	-0.17 (p = 0.71)	-0.12 (p = 0.80)	-0.29 (p = 0.57), n = 6
n-3 PUFA	Cecal	0.03 (p = 0.95)	0.05 (p = 0.92)	-0.54 (p = 0.27), n = 6
n-3 PUFA	Liver	0.18 (p = 0.70)	-0.75 (p = 0.05)	0.76 (p = 0.08), n = 6
n-3 PUFA	Plasma	-0.51 (p = 0.24)	-0.30 (p = 0.52)	0.51 (p = 0.30), n = 6

Table S4F. *Each composite is the mean Pareto-scaled abundance of its member metabolites (membership in Data S3, sheet Composite_members); n = number of members used (members constant across the 14 samples are omitted). Cohen d and p diet: standardized between-group difference and Welch's t-test; q (18): BH-adjusted across the 18 composites. Ordinary least squares with diet group and subcutaneous fat (SQ) as predictors, phenotype linked to the actual donors of each sample (mean of both donors for cecal and liver; single donor for 10 of 14 plasma samples): R^2 diet, diet alone; unique diet, R^2 full − R^2 SQ alone; shared, R^2 diet + R^2 SQ − R^2 full (commonality decomposition; negative values indicate suppression); unique SQ, R^2 full − R^2 diet. p diet ∣ SQ and p SQ ∣ diet: t-tests of each term in the two-predictor model; q ∣ SQ (18): BH across composites for the adjusted diet term. Sensitivity: adjusted diet p with body weight (BW) or hepatic lipid (mg lipid per g dry liver, quantified per animal) as covariate, and with SQ as covariate after omitting the highest-adiposity bean pool (pool 49). VIF, variance inflation factor of the SQ model; Shapiro, Shapiro–Wilk on residuals; B–P, Breusch–Pagan. Within-group Pearson r with p, and after omitting pool 49 (n = 6). Full coefficients with standard error (SE) and 95% confidence interval (CI) for every term, and results with mesenteric, total visceral, and epididymal fat as covariates, are produced by the Supplementary Code; Data S3 gives the coefficients for the subcutaneous-fat model (sheet OLS_coefficients) and the commonality decomposition with mesenteric, total visceral, and epididymal fat as covariates (sheet OLS_alternative_covariates, two-donor phenotype). A nominal diet term for one composite in these single-covariate models is not interpreted as retention of its class under part B (Section 3.9). We judge these analyses to be exploratory and associative: adiposity is a post-allocation variable, the shared component cannot be assigned, and with 14 observations the model cannot establish independence from adiposity.*

Table S4G. Permutation test and class-mean analysis of covariance for the class-level diet term under the conservative model (diet + depot PC1 + hepatic lipid), n = 14 pools.

Class	Direction of diet coefficient (cecal, liver, plasma)	Hotelling–Lawley trace	Parametric p, F(3, 8)	Permutation p	Class-mean ANCOVA F(1, 10)	Class-mean p	Leave-one-pool-out max p
Branched-chain amino acids	+, +, −	4.79	0.0020	0.0017	4.80	0.053	0.17
Glutathione-related	−, +, +	2.43	0.016	0.020	0.71	0.42	0.87
n-3 polyunsaturated fatty acids	+, +, +	2.05	0.024	0.023	14.5	0.0034	0.019
Ketone bodies / acylcarnitines	−, +, +	1.27	0.074	0.080	0.64	0.44	0.79
Ceramides	−, −, −	0.72	0.20	0.20	4.41	0.062	0.28
Bile acids	−, −, +	0.97	0.13	0.12	1.37	0.27	0.43

Table S4G. *Permutation p: Freedman–Lane residual permutation of the Hotelling–Lawley trace for the diet term (the reduced model with the two covariates was fitted, its residuals were permuted 10,000 times and added to its fitted values, the full model was refitted, and p = (count ≥ observed + 1)/(10,001); seed 20260924). Class-mean ANCOVA: ordinary least squares of the mean of the three compartment composites on diet with the same two z-scored covariates; F is the square of the t statistic for the diet term; the last column is the largest diet p-value when each pool is omitted in turn. The class mean is a valid one-response summary only where the three compartment composites share a direction (n-3, ceramides); where they do not (branched-chain, glutathione-related, ketone/acylcarnitine, bile acid), the mean cancels and the per-compartment analyses of Table S4D are the one-response checks. Computed by the Supplementary Code from the per-pool composite scores and the two-donor pool phenotype; the traces and F values are those of Table S4B (M4).*

Table S5. KEGG pathways significant in all three matrices

Pathway	Cecal FDR	Impact	Liver FDR	Impact	Plasma FDR	Impact
Ascorbate and aldarate metabolism	6.9×10^{-5}	0.68	3.4×10^{-5}	0.84	5.5×10^{-4}	0.68
Alanine, aspartate and glutamate metabolism	8.1×10^{-8}	0.74	3.2×10^{-6}	0.76	1.7×10^{-4}	0.74
Cysteine and methionine metabolism	2.6×10^{-7}	0.69	9.5×10^{-4}	0.76	3.2×10^{-4}	0.55
Pantothenate and CoA biosynthesis	5.3×10^{-6}	0.14	1.6×10^{-6}	0.75	1.7×10^{-2}	0.12
Pentose and glucuronate interconversions	1.3×10^{-4}	0.40	1.0×10^{-4}	0.71	1.7×10^{-3}	0.51
Glycerophospholipid metabolism	4.3×10^{-3}	0.42	2.7×10^{-7}	0.70	4.2×10^{-3}	0.59
Arginine and proline metabolism	2.5×10^{-8}	0.68	2.2×10^{-5}	0.68	3.1×10^{-4}	0.49
Arginine biosynthesis	2.0×10^{-7}	0.63	1.5×10^{-6}	0.63	1.1×10^{-4}	0.63
Phenylalanine metabolism	8.1×10^{-8}	0.62	2.7×10^{-2}	0.62	4.2×10^{-3}	0.62
Pyrimidine metabolism	1.0×10^{-8}	0.59	2.3×10^{-3}	0.61	1.8×10^{-2}	0.39
Nicotinate and nicotinamide metabolism	1.1×10^{-5}	0.33	1.8×10^{-4}	0.55	5.2×10^{-4}	0.33
Glutathione metabolism	1.7×10^{-4}	0.22	1.8×10^{-3}	0.50	7.2×10^{-3}	0.15
Pyruvate metabolism	8.8×10^{-7}	0.19	1.9×10^{-2}	0.41	3.1×10^{-4}	0.19
Sphingolipid metabolism	1.0×10^{-8}	0.50	1.1×10^{-4}	0.36	9.8×10^{-3}	0.60
Citrate cycle (TCA cycle)	4.1×10^{-7}	0.26	5.0×10^{-5}	0.35	1.7×10^{-4}	0.26
alpha-Linolenic acid metabolism	2.3×10^{-2}	0.33	1.9×10^{-4}	0.33	2.0×10^{-6}	0.33
Lysine degradation	1.6×10^{-4}	0.27	2.7×10^{-7}	0.29	6.8×10^{-12}	0.25
Tyrosine metabolism	8.1×10^{-8}	0.27	1.6×10^{-2}	0.25	6.1×10^{-3}	0.25
Glyoxylate and dicarboxylate metabolism	5.1×10^{-7}	0.19	9.3×10^{-4}	0.24	3.1×10^{-4}	0.25
Inositol phosphate metabolism	1.3×10^{-2}	0.13	4.4×10^{-2}	0.19	8.2×10^{-4}	0.13
Biosynthesis of unsaturated fatty acids	1.6×10^{-2}	0.00	4.2×10^{-3}	0.18	4.9×10^{-8}	0.00
Steroid hormone biosynthesis	1.5×10^{-5}	0.01	2.0×10^{-3}	0.01	3.1×10^{-4}	0.01

Table S5. *The 22 KEGG pathways with FDR < 0.05 in all three matrices (MetaboAnalyst 6.0 procedure and Mus musculus library, implemented in R; global test with relative-betweenness-centrality topology), sorted by liver impact. FDR, the Benjamini–Hochberg-adjusted p-value (q-value) from the global test, is non-directional; impact is the pathway-topology score and is reported as a distinct quantity. All 22 are shown in Figure 3 of the main text. Full per-matrix pathway results, including pathways significant in one or two matrices, are produced by the Supplementary Code; the corresponding MetaboAnalyst web-server results are in Data S2.*

Table S6. Pairwise polyunsaturated fatty-acid ratios

Matrix	Ratio	FC (bean/control)	95% CI	Welch t	df	p	q (within matrix)	Cohen d	Mann–Whitney p
Cecal content	EPA / arachidonate (20:5n3 / 20:4n6)	2.69	1.83–3.93	5.67	11.4	1.2×10^{-4}	3.3×10^{-4}	3.03	5.8×10^{-4}
Cecal content	n3-DPA / n6-DPA (22:5n3 / 22:5n6)	17.40	8.46–35.79	8.77	10.5	3.8×10^{-6}	3.0×10^{-5}	4.69	5.8×10^{-4}
Cecal content	n3-DPA / adrenate (22:5n3 / 22:4n6)	4.98	2.79–8.88	6.06	11.7	6.3×10^{-5}	2.5×10^{-4}	3.24	0.001
Cecal content	DHA / arachidonate (22:6n3 / 20:4n6)	2.29	1.60–3.28	5.37	7.7	7.7×10^{-4}	0.001	2.87	0.002
Cecal content	EPA / linolenate (product / precursor, n-3)	0.60	0.31–1.13	-1.77	11.4	0.104	0.139	-0.94	0.097
Cecal content	arachidonate / linoleate (product / precursor, n-6)	0.81	0.40–1.65	-0.64	12.0	0.531	0.531	-0.34	0.535
Cecal content	stearidonate / linolenate (Δ6 step, n-3)	0.10	0.04–0.27	-5.23	9.5	4.5×10^{-4}	9.0×10^{-4}	-2.79	5.8×10^{-4}
Cecal content	mead / arachidonate (20:3n9 / 20:4n6)	0.61	0.19–1.98	-0.92	11.9	0.376	0.429	-0.49	0.318
Liver	EPA / arachidonate (20:5n3 / 20:4n6)	10.44	7.56–14.40	16.22	10.0	1.7×10^{-8}	6.8×10^{-8}	8.67	5.8×10^{-4}
Liver	n3-DPA / n6-DPA (22:5n3 / 22:5n6)	22.35	10.27–48.63	9.60	6.5	4.6×10^{-5}	5.2×10^{-5}	5.13	5.8×10^{-4}
Liver	n3-DPA / adrenate (22:5n3 / 22:4n6)	8.02	5.70–11.28	14.63	6.6	2.9×10^{-6}	4.7×10^{-6}	7.82	5.8×10^{-4}
Liver	DHA / arachidonate (22:6n3 / 20:4n6)	2.04	1.76–2.36	11.09	8.6	2.1×10^{-6}	4.7×10^{-6}	5.93	5.8×10^{-4}
Liver	EPA / linolenate (product / precursor, n-3)	3.21	2.63–3.92	13.88	6.9	2.6×10^{-6}	4.7×10^{-6}	7.42	5.8×10^{-4}
Liver	arachidonate / linoleate (product / precursor, n-6)	0.55	0.46–0.67	-7.04	11.7	1.5×10^{-5}	2.1×10^{-5}	-3.76	5.8×10^{-4}
Liver	stearidonate / linolenate (Δ6 step, n-3)	3.05	2.74–3.39	23.32	9.9	5.5×10^{-10}	4.4×10^{-9}	12.47	5.8×10^{-4}
Liver	mead / arachidonate (20:3n9 / 20:4n6)	0.51	0.38–0.69	-4.93	10.1	5.9×10^{-4}	5.9×10^{-4}	-2.63	5.8×10^{-4}
Plasma	EPA / arachidonate (20:5n3 / 20:4n6)	10.33	8.07–13.22	21.70	8.2	1.5×10^{-8}	1.2×10^{-7}	11.60	5.8×10^{-4}
Plasma	n3-DPA / n6-DPA (22:5n3 / 22:5n6)	17.62	8.87–35.00	9.82	7.2	1.9×10^{-5}	3.8×10^{-5}	5.25	5.8×10^{-4}
Plasma	n3-DPA / adrenate (22:5n3 / 22:4n6)	7.83	4.06–15.12	7.29	7.5	1.1×10^{-4}	1.8×10^{-4}	3.90	5.8×10^{-4}
Plasma	DHA / arachidonate (22:6n3 / 20:4n6)	3.05	2.50–3.72	13.42	6.8	3.7×10^{-6}	1.5×10^{-5}	7.17	5.8×10^{-4}
Plasma	EPA / linolenate (product / precursor, n-3)	1.12	0.67–1.88	0.54	6.6	0.608	0.608	0.29	0.535
Plasma	arachidonate / linoleate (product / precursor, n-6)	0.32	0.25–0.42	-10.07	7.9	9.1×10^{-6}	2.4×10^{-5}	-5.38	5.8×10^{-4}
Plasma	stearidonate / linolenate (Δ6 step, n-3)	2.14	1.47–3.12	4.42	11.4	9.4×10^{-4}	0.001	2.36	0.002
Plasma	mead / arachidonate (20:3n9 / 20:4n6)	0.65	0.53–0.80	-4.77	8.5	0.001	0.001	-2.55	0.001

Table S6. *For any two metabolites, each scaled to its own median, the ratio of scaled values differs from the molar ratio only by a constant, so the between-group FC in the pairwise ratio equals the FC in the molar ratio. Per-sample $\log_2$ ratios were formed as differences of $\log_2$ abundances (from the batch-normalized, minimum-imputed data in Data S6), compared by Welch's t-test (FC with 95% CI; Cohen d) and by the Mann–Whitney U test (exact without ties), with BH correction across the eight ratios within each matrix. n = 7 pools per group. A summed Σn-3/Σn-6 ratio is not used because a sum of median-scaled abundances is a weighted sum with arbitrary weights.*

Table S7. Hepatic transcripts for five gene sets and the matched fatty-acid indices

RNA-seq of the 20 male liver pools (10 per diet), including the 14 pools profiled by metabolomics with the same two donors per pool for both assays. Part A gives the differential-expression results for the five gene sets, specified from the metabolite results before the per-gene values were extracted but not blind to the genome-wide report (ref. 6 of the main text); part B gives the product-to-precursor fatty-acid indices from the 14 shared pools, each paired with the transcript(s) for the step it interrogates; part C gives the transcript–index correlations. Complete tables, per-pool CPM, and the genome-wide male differential-expression table are in Data S7.

A. Differential expression, bean vs. control (TMM-normalized CPM; empirical-Bayes moderated t with mean–variance trend on $\log_2$(CPM + 1), n = 10 pools per diet, Benjamini–Hochberg FDR across 12,116 retained genes; and Welch's t-test on the 7 + 7 pools shared with the metabolomics)

Gene set	Gene	Mean CPM, control	Mean CPM, bean	$\log_2$FC (n = 10)	FDR (n = 10)	$\log_2$FC (7 shared pools)	Welch p (7 vs. 7)
PUFA desaturation/elongation	*Fads2*	1,585	721	-1.16	2.3×10^{-7}	-1.30	5.1×10^{-8}
PUFA desaturation/elongation	*Fads1*	1,017	629	-0.71	1.8×10^{-5}	-0.78	1.7×10^{-5}
PUFA desaturation/elongation	*Elovl5*	1,778	911	-0.99	2.2×10^{-5}	-1.13	2.7×10^{-4}
PUFA desaturation/elongation	*Elovl2*	800	599	-0.44	0.006	-0.52	0.003
Lipogenesis (SREBP-1c targets)	*Scd1*	25,523	10,725	-1.28	2.2×10^{-5}	-1.46	5.7×10^{-5}
Lipogenesis (SREBP-1c targets)	*Elovl6*	369	187	-0.95	4.0×10^{-4}	-1.19	9.9×10^{-6}
Lipogenesis (SREBP-1c targets)	*Fasn*	2,625	1,563	-0.69	0.005	-0.89	1.6×10^{-4}
Lipogenesis (SREBP-1c targets)	*Acaca*	347	202	-0.74	7.4×10^{-5}	-0.86	3.4×10^{-5}
Fatty-acid oxidation (PPARα axis)	*Ppara*	106	86	-0.28	0.07	-0.41	0.003
Fatty-acid oxidation (PPARα axis)	*Acox1*	2,735	2,538	-0.10	0.51	-0.14	0.07
Fatty-acid oxidation (PPARα axis)	*Cpt1a*	502	347	-0.53	2.3×10^{-6}	-0.60	4.9×10^{-7}
Fatty-acid oxidation (PPARα axis)	*Cyp4a14*	439	530	+0.22	0.53	+0.18	0.61
BCAA catabolism	*Bcat2*	9	12	+0.31	0.01	+0.34	0.007
BCAA catabolism	*Bckdha*	259	266	+0.04	0.72	+0.02	0.76
BCAA catabolism	*Bckdhb*	63	57	-0.14	0.24	-0.18	0.06
BCAA catabolism	*Dbt*	59	68	+0.20	0.04	+0.22	0.04
BCAA catabolism	*Dld*	122	119	-0.04	0.67	-0.05	0.34
BCAA catabolism	*Bckdk*	15	15	+0.06	0.61	+0.02	0.83
BCAA catabolism	*Ppm1k*	62	72	+0.22	0.06	+0.17	0.19
Glutathione synthesis/turnover	*Gclc*	559	587	+0.06	0.66	+0.13	0.28
Glutathione synthesis/turnover	*Gclm*	199	257	+0.37	4.6×10^{-6}	+0.38	1.6×10^{-7}
Glutathione synthesis/turnover	*Gss*	77	81	+0.09	0.40	+0.16	0.10
Glutathione synthesis/turnover	*Chac1*	3	4	+0.17	0.57	+0.57	0.02
Glutathione synthesis/turnover	*Gsr*	89	98	+0.14	0.01	+0.13	0.004

B. Fatty-acid product-to-precursor indices in the 14 shared pools (geometric-mean FC of per-pool ratios; Welch's t-test on log_2 ratios) and the transcript(s) for the corresponding step

Index	Step interrogated	FC, bean/control	Welch p	Matched transcript(s)	Transcript log_2FC
Dihomo-γ-linolenate : linoleate	Δ6 + ELOVL5, n-6	0.71	0.001	*Fads2, Elovl5*	−1.16, −0.99
Arachidonate : dihomo-γ-linolenate	Δ5, n-6	0.78	0.01	*Fads1*	−0.71
Arachidonate : linoleate	n-6 arm, C18 → C20	0.55	1.5×10^{-5}	*Fads2, Elovl5, Fads1*	−1.16, −0.99, −0.71
Adrenate : arachidonate	ELOVL2/5, n-6	0.71	0.002	*Elovl2, Elovl5*	−0.44, −0.99
Stearidonate : α-linolenate	Δ6, n-3	3.05	5.5×10^{-10}	*Fads2*	−1.16
Eicosapentaenoate : α-linolenate	n-3 arm, C18 → C20	3.21	2.6×10^{-6}	*Fads2, Elovl5, Fads1*	−1.16, −0.99, −0.71
n-3 docosapentaenoate : eicosapentaenoate	ELOVL2, n-3	0.55	3.6×10^{-7}	*Elovl2*	−0.44
Docosahexaenoate : eicosapentaenoate	ELOVL2 + Δ6 + β-oxidation, n-3	0.20	7.1×10^{-6}	*Elovl2, Fads2*	−0.44, −1.16
Eicosapentaenoate : arachidonate	end products, n-3 vs. n-6	10.44	1.7×10^{-8}	—	—
Palmitoleate : palmitate	SCD1	0.55	3.7×10^{-5}	*Scd1*	−1.28
Mead acid (20:3n-9), single metabolite	Δ6/Δ5 product of oleate	0.29	2.1×10^{-4} (q)	*Fads2, Fads1*	−1.16, −0.71

C. Transcript–index correlations (log_2 CPM vs. log_2 index)

Gene	Index	Pearson r, 14 pools	Spearman ρ (p), 14 pools	Spearman ρ (p), control, n = 7	Spearman ρ (p), bean, n = 7
Fads2	arachidonate : linoleate	+0.87	+0.71 (6.0×10^{-3})	-0.57 (0.20)	+0.21 (0.66)
Fads2	dihomo-γ-linolenate : linoleate	+0.81	+0.71 (5.6×10^{-3})	-0.04 (0.96)	+0.36 (0.44)
Fads1	arachidonate : dihomo-γ-linolenate	+0.53	+0.45 (1.1×10^{-1})	+0.04 (0.96)	-0.57 (0.20)
Elovl5	arachidonate : linoleate	+0.88	+0.80 (1.0×10^{-3})	-0.07 (0.91)	+0.43 (0.35)
Fads2	stearidonate : α-linolenate	-0.97	-0.77 (1.9×10^{-3})	+0.29 (0.56)	-0.43 (0.35)
Fads2	eicosapentaenoate : α-linolenate	-0.96	-0.80 (1.0×10^{-3})	+0.07 (0.91)	-0.43 (0.35)
Elovl2	n-3 DPA : EPA	+0.69	+0.68 (9.5×10^{-3})	-0.25 (0.59)	+0.00 (1.00)
Elovl2	DHA : EPA	+0.81	+0.78 (1.4×10^{-3})	-0.21 (0.66)	+0.79 (0.05)
Elovl2	adrenate : arachidonate	+0.75	+0.77 (2.1×10^{-3})	+0.04 (0.96)	+0.79 (0.05)
Scd1	palmitoleate : palmitate	+0.85	+0.81 (8.1×10^{-4})	+0.32 (0.50)	+0.32 (0.50)

Table S7. *Transcripts were lower for the three enzymes shared by the n-3 and n-6 pathways (Fads2, Elovl5, Fads1) and for Elovl2. The n-6 indices (part B, first four rows) fell with the transcripts, whereas the n-3 indices for the same steps rose and the ELOVL2-dependent steps beyond eicosapentaenoate fell; the stearoyl-CoA desaturase index and mead acid fell with Scd1 and the desaturase transcripts. In part C the across-pool coefficients are dominated by the between-group difference (the two diets form separate clusters on both axes; Figure S2B–D) and are not evidence of a within-group relation; the within-group coefficients at n = 7 are imprecise and inconsistent in sign. Bcat1 was not retained (CPM < 1); Chac1 is expressed at about 3 CPM and its shared-pool p-value reflects one or two pools. Transcript abundance is not enzyme activity; the branched-chain keto-acid dehydrogenase complex is regulated by phosphorylation, and SREBP-1c activity is set post-translationally. FDR values (Benjamini–Hochberg-adjusted p-values, i.e., q-values) are from limma-trend (R package limma; prior degrees of freedom 4.7, residual degrees of freedom 18); Spearman p-values are exact. The RNA-seq data are those reported in ref. 6 of the main text (GEO accession GSE227120); the gene-set analysis is new. The fatty-acid indices are formed on the free fatty-acid pool measured by the*

platform, not on phospholipid or total fatty acids. Library preparation, sequencing, and alignment details are given in Section 2.8 of the main text and Supplementary Methods SM8.

Table S8. Cage-aware sensitivity analysis

Table S8A. Per-composite multiple-membership mixed model with a cage random effect: diet term under M0 and M4 with 95% CI and leave-one-cage-out (LOCO) sensitivity (n = 14 pools, 8 cages).

Class	Model	Compartment	b diet, OLS	p, OLS	b diet, mixed model	SE	95% CI	p, 6 df	Cage variance	Residual variance	ICC	LOCO max p	LOCO b range	LOCO sign
Branched-chain amino acids	M0	Cecal	2.05	2.7×10^{-8}	2.05	0.21	1.53 to 2.57	6.9×10^{-5}	0.049	0.068	0.42	0.004	1.94 to 2.19	Yes
Branched-chain amino acids	M0	Liver	1.30	4.1×10^{-7}	1.32	0.18	0.88 to 1.76	3.4×10^{-4}	0.041	0.040	0.51	0.007	1.18 to 1.51	Yes
Branched-chain amino acids	M0	Plasma	-0.57	0.037	-0.56	0.25	-1.18 to 0.07	0.072	0.013	0.201	0.06	0.24	-0.70 to -0.35	Yes
Branched-chain amino acids	M4	Cecal	1.52	0.001	1.51	0.40	0.54 to 2.49	0.009	0.064	0.056	0.53	0.12	1.25 to 1.72	Yes
Branched-chain amino acids	M4	Liver	1.42	4.5×10^{-4}	1.39	0.30	0.66 to 2.11	0.003	0.011	0.051	0.18	0.067	1.05 to 1.64	Yes
Branched-chain amino acids	M4	Plasma	-1.20	0.023	-1.20	0.45	-2.30 to -0.10	0.037	0.000	0.148	0.00	0.081	-3.06 to -0.87	Yes
Glutathione-related	M0	Cecal	-0.55	0.15	-0.55	0.35	-1.41 to 0.32	0.17	0.003	0.435	0.01	0.59	-1.04 to -0.23	Yes
Glutathione-related	M0	Liver	0.95	5.0×10^{-6}	0.94	0.16	0.54 to 1.34	0.001	0.033	0.035	0.48	0.019	0.79 to 1.14	Yes
Glutathione-related	M0	Plasma	0.65	0.004	0.65	0.20	0.17 to 1.13	0.016	0.016	0.104	0.13	0.10	0.55 to 0.75	Yes
Glutathione-related	M4	Cecal	-0.56	0.51	-0.57	0.82	-2.58 to 1.45	0.52	0.014	0.479	0.03	0.86	-1.22 to 0.37	No
Glutathione-related	M4	Liver	0.87	0.010	0.77	0.31	0.01 to 1.53	0.047	0.040	0.033	0.55	0.97	-0.02 to 1.32	No
Glutathione-related	M4	Plasma	0.63	0.18	0.63	0.43	-0.43 to 1.68	0.20	0.000	0.135	0.00	0.81	-0.28 to 0.81	No
n-3 polyunsaturated fatty acids	M0	Cecal	1.28	0.036	1.28	0.54	-0.05 to 2.61	0.056	0.000	1.028	0.00	0.33	0.90 to 1.79	Yes
n-3 polyunsaturated fatty acids	M0	Liver	1.45	1.1×10^{-4}	1.45	0.26	0.82 to 2.07	0.001	0.000	0.229	0.00	0.022	1.31 to 1.72	Yes
n-3 polyunsaturated fatty acids	M0	Plasma	1.68	1.6×10^{-6}	1.68	0.26	1.05 to 2.31	6.1×10^{-4}	0.082	0.078	0.51	0.011	1.52 to 2.02	Yes
n-3 polyunsaturated fatty acids	M4	Cecal	1.40	0.25	1.43	1.17	-1.42 to 4.29	0.27	0.080	0.901	0.08	0.85	-0.80 to 2.28	No
n-3 polyunsaturated fatty acids	M4	Liver	1.39	0.036	1.39	0.57	-0.01 to 2.79	0.052	0.000	0.239	0.00	0.64	-0.60 to 2.26	No
n-3 polyunsaturated fatty acids	M4	Plasma	1.60	0.004	1.89	0.51	0.64 to 3.13	0.010	0.114	0.075	0.60	0.079	1.42 to 2.75	Yes
Ketone bodies / acylcarnitines	M0	Cecal	-0.20	0.52	-0.17	0.45	-1.26 to 0.92	0.72	0.270	0.210	0.56	0.97	-0.50 to 0.07	No
Ketone bodies / acylcarnitines	M0	Liver	1.17	0.002	1.17	0.30	0.45 to 1.89	0.008	0.000	0.306	0.00	0.065	0.96 to 1.44	Yes
Ketone bodies / acylcarnitines	M0	Plasma	0.24	0.49	0.06	0.43	-0.98 to 1.11	0.89	0.245	0.189	0.56	0.96	-0.34 to 0.72	No
Ketone bodies / acylcarnitines	M4	Cecal	-0.80	0.26	-0.73	0.80	-2.68 to 1.23	0.40	0.390	0.160	0.71	0.93	-1.36 to 0.10	No

Class	Model	Compartment	b diet, OLS	p, OLS	b diet, mixed model	SE	95% CI	p, 6 df	Cage variance	Residual variance	ICC	LOCO max p	LOCO b range	LOCO sign
Ketone bodies / acylcarnitines	M4	Liver	1.62	0.010	1.62	0.51	0.36 to 2.87	0.020	0.000	0.193	0.00	0.092	1.13 to 3.04	Yes
Ketone bodies / acylcarnitines	M4	Plasma	0.21	0.80	-0.09	0.73	-1.87 to 1.70	0.91	0.437	0.079	0.85	0.94	-0.37 to 0.85	No
Ceramides	M0	Cecal	-0.20	0.24	-0.20	0.16	-0.59 to 0.19	0.26	0.000	0.089	0.00	0.50	-0.32 to -0.12	Yes
Ceramides	M0	Liver	-0.90	1.3×10^{-4}	-0.91	0.20	-1.39 to -0.42	0.004	0.033	0.079	0.30	0.046	-1.02 to -0.62	Yes
Ceramides	M0	Plasma	-0.84	4.2×10^{-4}	-0.83	0.20	-1.31 to -0.34	0.006	0.029	0.083	0.26	0.050	-1.07 to -0.69	Yes
Ceramides	M4	Cecal	-0.10	0.80	-0.10	0.38	-1.03 to 0.83	0.80	0.000	0.106	0.00	0.93	-0.36 to -0.04	Yes
Ceramides	M4	Liver	-0.82	0.063	-0.77	0.44	-1.84 to 0.30	0.13	0.045	0.091	0.33	0.35	-1.11 to -0.51	Yes
Ceramides	M4	Plasma	-0.44	0.26	-0.44	0.38	-1.37 to 0.49	0.29	0.006	0.097	0.06	0.93	-0.91 to 0.97	No
Bile acids	M0	Cecal	-0.73	0.004	-0.73	0.20	-1.23 to -0.23	0.011	0.000	0.146	0.00	0.073	-1.08 to -0.67	Yes
Bile acids	M0	Liver	-0.00	0.99	0.01	0.26	-0.63 to 0.64	0.99	0.058	0.132	0.30	0.97	-0.11 to 0.25	No
Bile acids	M0	Plasma	0.47	0.35	0.31	0.56	-1.06 to 1.67	0.60	0.254	0.606	0.30	0.71	-0.28 to 0.72	No
Bile acids	M4	Cecal	-0.57	0.26	-0.57	0.47	-1.73 to 0.59	0.27	0.000	0.163	0.00	0.61	-0.84 to 0.62	No
Bile acids	M4	Liver	-0.88	0.029	-0.88	0.35	-1.73 to -0.03	0.044	0.000	0.087	0.00	0.78	-1.12 to -0.26	Yes
Bile acids	M4	Plasma	0.05	0.96	0.00	1.24	-3.03 to 3.04	1.00	0.390	0.665	0.37	0.98	-0.98 to 0.78	No

Table S8B. Class-level MANCOVA of Table 1 (pool-level model): leave-one-cage-out sensitivity, every pool holding a donor from the omitted cage removed.

Class	Model	F	p	q (six classes)	LOCO min p	LOCO max p	Fewest pools retained
Branched-chain amino acids	M0	69.5	5.3×10^{-7}	3.2×10^{-6}	1.3×10^{-6}	4.8×10^{-4}	10
Branched-chain amino acids	M4	12.8	0.002	0.012	0.002	0.055	10
Glutathione-related	M0	33.5	1.6×10^{-5}	4.8×10^{-5}	4.0×10^{-5}	0.003	10
Glutathione-related	M4	6.5	0.016	0.047	0.011	0.96	10
n-3 polyunsaturated fatty acids	M0	23.9	7.0×10^{-5}	1.4×10^{-4}	6.8×10^{-5}	0.003	10
n-3 polyunsaturated fatty acids	M4	5.5	0.024	0.049	0.006	0.20	10
Ketone bodies / acylcarnitines	M0	5.0	0.022	0.026	1.7×10^{-4}	0.19	10
Ketone bodies / acylcarnitines	M4	3.4	0.074	0.11	0.006	0.28	10
Ceramides	M0	18.5	2.1×10^{-4}	3.2×10^{-4}	2.3×10^{-4}	0.017	10
Ceramides	M4	1.9	0.20	0.20	0.079	0.60	10
Bile acids	M0	4.0	0.040	0.040	0.017	0.28	10
Bile acids	M4	2.6	0.13	0.15	0.034	0.97	10

Table S8C. Per-metabolite diet comparisons on the host feature set: Welch's t-test against the cage-aware mixed model.

Matrix	Host features	FDR < 0.05, Welch	FDR < 0.05, mixed model (6 df)	Mixed-model set within Welch set	Cage variance estimated at 0	ICC, median	ICC, 90th percentile
Cecal	768	401	262	262 (65%)	35%	0.26	0.75
Liver	948	352	252	252 (72%)	54%	0.00	0.59
Plasma	895	235	133	133 (57%)	35%	0.18	0.67

Table S8D. The 36 concordant host metabolites of Table S3 under the cage-aware mixed model (p on 6 df).

Metabolite	log_2FC cecal	log_2FC liver	log_2FC plasma	p cecal	p liver	p plasma	ICC cecal	ICC liver	ICC plasma	p < 0.05 in all three
fumarate	+4.11	+1.32	+1.35	8.3×10^{-5}	4.2×10^{-4}	4.6×10^{-4}	0.00	0.00	0.00	Yes
gulonate*	+2.21	+0.74	+0.77	2.0×10^{-4}	5.2×10^{-4}	0.005	0.00	0.00	0.00	Yes
alpha-hydroxyisocaproate	+2.95	+1.70	+1.37	5.1×10^{-4}	0.005	0.030	0.40	0.33	0.75	Yes
1-palmitoyl-2-linoleoyl-GPE (16:0/18:2)	+2.22	+1.93	+1.96	5.2×10^{-4}	3.2×10^{-5}	7.7×10^{-5}	0.00	0.00	0.00	Yes

Metabolite	log_2FC cecal	log_2FC liver	log_2FC plasma	p cecal	p liver	p plasma	ICC cecal	ICC liver	ICC plasma	$p < 0.05$ in all three
1-stearoyl-2-arachidonoyl-GPC (18:0/20:4)	-2.61	-0.66	-0.77	0.001	2.7×10^{-4}	0.011	0.19	0.18	0.53	Yes
alpha-tocopherol	-0.37	-0.43	-0.62	0.001	0.013	0.007	0.00	0.08	0.00	Yes
1-palmitoyl-2-arachidonoyl-GPC (16:0/20:4n6)	-2.19	-0.38	-0.58	0.002	9.6×10^{-5}	0.023	0.00	0.00	0.42	Yes
citrate	+3.28	+2.45	+0.42	0.003	0.002	0.012	0.43	0.55	0.54	Yes
N-stearoyl-sphingosine (d18:1/18:0)*	-0.99	-1.69	-2.04	0.004	7.9×10^{-4}	8.6×10^{-4}	0.34	0.00	0.00	Yes
N-glycolylneuraminate	-0.85	-0.23	-0.68	0.005	0.042	0.013	0.13	0.00	0.00	Yes
1-linoleoyl-GPI (18:2)*	+2.34	+0.86	+0.91	0.014	0.003	9.5×10^{-4}	0.69	0.00	0.00	Yes
N1-methyl-2-pyridone-5-carboxamide	-2.65	-0.76	-1.29	0.015	0.004	0.002	0.00	0.41	0.48	Yes
N,N,N-trimethyl-5-aminovalerate	+1.33	+1.65	+1.54	0.018	9.2×10^{-5}	0.003	0.57	0.26	0.56	Yes
docosapentaenoate (n3 DPA; 22:5n3)	+1.15	+1.78	+1.52	0.021	5.7×10^{-4}	0.003	0.00	0.00	0.00	Yes
linolenate [alpha or gamma; (18:3n3 or 6)]	+1.50	+0.98	+2.06	0.023	0.022	9.1×10^{-4}	0.00	0.00	0.66	Yes
glucuronate	+0.49	+0.49	+0.41	0.029	0.011	0.023	0.00	0.44	0.81	Yes
eicosapentaenoate (EPA; 20:5n3)	+1.22	+2.67	+2.22	0.032	7.7×10^{-5}	1.3×10^{-4}	0.16	0.00	0.21	Yes
1-arachidonoyl-GPC (20:4n6)*	-1.38	-0.85	-0.96	0.034	8.2×10^{-5}	3.6×10^{-5}	0.71	0.00	0.00	Yes
lactate	+2.06	+0.31	+0.36	0.035	0.047	0.001	0.38	0.37	0.00	Yes
myo-inositol	+0.37	+0.94	+1.02	0.047	3.4×10^{-4}	0.016	0.00	0.00	0.50	Yes
cholesterol sulfate	-0.84	-0.24	-0.70	4.9×10^{-5}	0.17	8.2×10^{-4}	0.00	0.84	0.15	No
1-(1-enyl-palmitoyl)-GPE (P-16:0)*	-2.17	-0.40	-0.18	0.002	0.075	0.060	0.00	0.56	0.42	No
taurohyodeoxycholic acid	-1.78	-1.01	-0.98	0.005	0.092	0.045	0.00	0.67	0.00	No
pseudouridine	+1.29	+0.26	+0.27	0.018	0.057	0.024	0.36	0.00	0.00	No
trimethylamine N-oxide	+1.52	+0.92	+0.86	0.022	0.065	0.044	0.93	0.48	0.00	No
2-hydroxy-3-methylvalerate	+1.90	+0.80	+0.39	0.023	0.005	0.055	0.85	0.11	0.00	No
1-stearoyl-2-oleoyl-GPC (18:0/18:1)	-1.40	-0.80	-0.51	0.025	1.5×10^{-4}	0.072	0.00	0.00	0.34	No
N2-acetyllysine	+0.50	+0.90	+0.41	0.044	7.6×10^{-4}	0.067	0.00	0.00	0.00	No
indolelactate	-2.31	-0.72	-0.93	0.050	0.005	0.023	0.00	0.00	0.55	No
N6-acetyllysine	+0.59	+0.45	+0.67	0.054	0.004	1.0×10^{-4}	0.57	0.21	0.20	No
adenine	-1.97	-0.38	-1.60	0.058	0.12	0.002	0.84	0.82	0.00	No
1-palmitoyl-2-oleoyl-GPC (16:0/18:1)	-1.19	-0.28	-0.46	0.060	4.0×10^{-4}	0.031	0.00	0.00	0.43	No
2-O-methylascorbic acid	-1.62	-1.01	-0.86	0.086	7.2×10^{-5}	4.8×10^{-4}	0.40	0.00	0.19	No
N-acetylglutamine	+0.74	+0.66	+0.99	0.13	0.033	0.001	0.68	0.21	0.00	No
1,2-dilinoleoyl-GPE (18:2/18:2)*	+0.91	+2.42	+2.59	0.20	1.3×10^{-4}	0.024	0.98	0.00	0.90	No
1-linoleoyl-GPE (18:2)*	+1.47	+1.56	+0.63	0.38	6.4×10^{-5}	0.048	0.91	0.00	0.00	No

Table S8. *Diets were assigned to whole cages (4 cages of 5 mice per diet) with body-weight balancing by exchange of individual animals, and pools were formed by adiposity rank without regard to cage, so the 14 profiled pools draw on 8 cages, 5 as same-cage pairs and 9 spanning two cages, and each cage contributes a donor to 2–4 pools (Data S3, sheet Cage_key); procedures are given in Supplementary Methods SM9. The allocation was balanced rather than an unrestricted randomization of cages, so these are model-based sensitivity analyses; they do not add replication, and the treatment-level replication is 4 cages per diet. A: for each of the 18 composites, the pool-level ordinary least-squares (OLS) diet coefficient and p-value under the same predictors, and the multiple-membership mixed model (cage random effect; pool weight 1/2 on each donor cage, 1 on a single cage; plasma weighted to its contributing donors) with the diet coefficient, standard error (SE), 95% confidence interval and approximate Wald p-value on 6 denominator degrees of freedom (8 cages − 2 diets), the restricted-maximum-likelihood cage and residual variance components (Pareto-scaled units), and the intraclass correlation (ICC) = cage variance/(cage + residual variance). Where the cage variance is estimated at zero the coefficient equals the OLS coefficient and only the degrees of freedom differ; with eight cages a zero estimate means that cage and residual variance are not separated, not that cage effects are absent. LOCO: the model refitted eight times with every pool holding a donor from one cage omitted (10–12 pools remain; Wald test on 5 degrees of freedom), giving the largest diet p-value, the range of the diet coefficient, and whether its sign matched the full-sample estimate in all eight refits. M0, diet only; M4, diet + depot PC1 + hepatic lipid, with the covariates z-scored as in Table S4. B: the parametric Hotelling–Lawley F, p-value, and Benjamini–Hochberg q-value across the six classes of Table S4B, with the smallest and largest diet p-value across the eight leave-one-cage-out refits of the pool-level model and the fewest pools retained (omitting cage 8 or cage 14 removes 4 pools and leaves 4 denominator degrees of freedom for the diet test under M4). The mixed models of part A are fitted to one composite at a time and do not test the joint class hypothesis; part B is the cage-level check on that joint test. C: counts of host features at FDR < 0.05 (Benjamini–Hochberg within matrix across the host feature set) by Welch's t-test and by the mixed model with 6 degrees of freedom; the*

number of the latter that are also in the former; the proportion of features whose cage variance was estimated at zero; and the median and 90th percentile of the ICC across host features. D: for the 36 metabolites of Table S3, log_2FC (bean/control), the mixed-model p-value and ICC in each compartment, and whether $p < 0.05$ in all three; 20 of 36 do. The per-feature results for every host feature are given in Data S9.

Figure S1

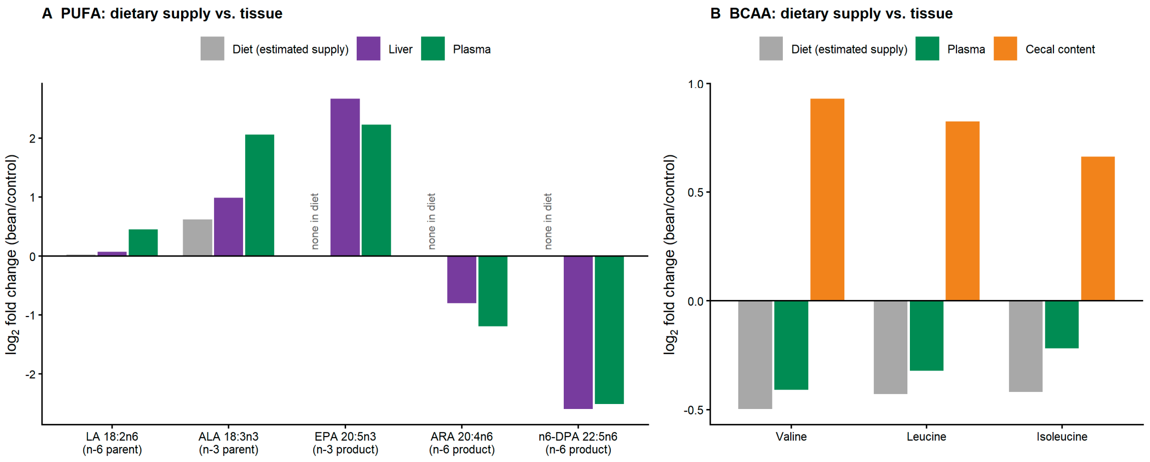

Figure S1. *Dietary supply versus tissue response. (A) For polyunsaturated fatty acids, the ≥20-carbon n-6 products are lower in tissue although their dietary precursor (linoleate) is unchanged, and eicosapentaenoate is higher in tissue although it is absent from both diets and estimated α-linolenate supply is 1.5-fold higher in the bean diet. We interpret the pattern as consistent with competition between the n-3 and n-6 precursor families for the shared Δ6/Δ5 desaturase–elongase pathway under a higher n-3 precursor supply; transcripts of the shared desaturase and elongase enzymes were lower, not higher, with bean feeding (Figure S2; Table S7), enzyme activity was not measured, and the pattern does not demonstrate host enzyme redirection. (B) For BCAA, plasma changes are in the direction of the estimated 27% lower dietary supply of the bean arm, whereas cecal BCAA and their keto and hydroxy acids are higher, opposite to intake; we describe this as cecal accumulation against lower supply, compatible with altered intestinal BCAA handling, and the alternatives listed in Section 3.4 of the main text are not excluded. Dietary values are ingredient-based estimates (Table S1); tissue values are measured log_2FC values (bean/control), $n = 7$ pools per group.*

Figure S2

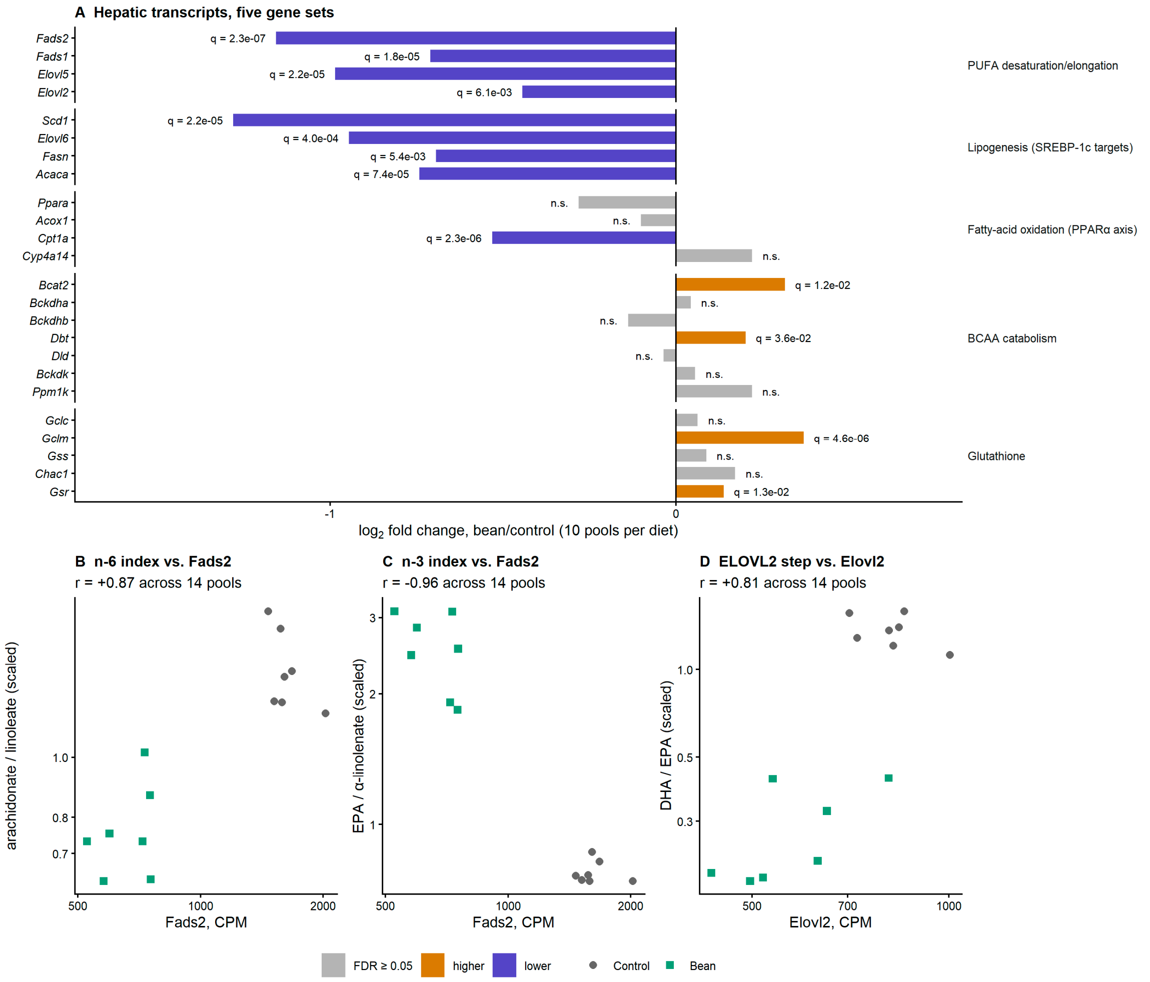

Figure S2. *Hepatic transcripts and their relation to the fatty-acid indices. (A) log₂FC (bean/control) of transcripts in the five candidate gene sets from RNA-seq of the 20 male liver pools (n = 10 per diet); purple, lower at FDR < 0.05; orange, higher at FDR < 0.05; grey, FDR ≥ 0.05; FDR values are printed beside each bar. (B–D) Per-pool transcript abundance (CPM) against the fatty-acid index in the 14 pools shared with the metabolomics (grey circles, control; green squares, bean; both axes logarithmic): (B) Fads2 against arachidonate : linoleate, the n-6 product-to-precursor index; (C) Fads2 against eicosapentaenoate : α-linolenate, the n-3 index; (D) Elovl2 against docosahexaenoate : eicosapentaenoate, the ELOVL2-dependent step. The Pearson r printed in each panel is computed across all 14 pools and is dominated by the separation of the two diet groups; it is not evidence of a within-group relation (Table S7, part C). Fatty-acid values are median-scaled batch-normalized abundances (Data S6) and the indices are ratios of those values, which differ from molar ratios by a constant.*

Supplementary data files

File	Description	Filename
Data S1	Primary analysis workbook: differential abundance per matrix with Welch and Mann–Whitney p and FDR, missing-value counts per feature, exposure-marker flags with the rule applied to each excluded compound (sheet Exposure_marker_list; column Exposure_marker_corrected), PLS-DA summary and VIP for the host set, cross-matrix concordance, pool phenotypes, platform QC summary, and the plasma single-donor sensitivity analysis (sheet PLASMA_single_donor_sens)	COSU-01-23VW_Bean_vs_Control_metabolomics_analysis_R1.xlsx
Data S2	MetaboAnalyst cross-tissue enrichment and pathway-topology results from the MetaboAnalyst web server, with the per-matrix results of the R implementation used for Table S5 (sheets R_Pathway_Cecal, R_Pathway_Liver, R_Pathway_Plasma)	COSU-01-23VW_MetaboAnalyst_crosstissue_summary.xlsx
Data S3	Pooling crosswalk for all 20 pools per group (donors with the cage of each donor, submission status, tissue mass and plasma volume per donor, donor SQ fat/tibia rank), donor-correct phenotypes per sample, composite membership, per-pool scores for all 18 composites, full regression output with coefficients, alternative covariates and sensitivity analyses, and pairwise fatty-acid ratios. Sheet Cage_key lists every cage of the dose-response study with its diet, animals, pools, and profiled pools (GROUP CONTROL or BEAN marks the 8 cages of the two diets analyzed here); sheet Donor_correct_phenotype carries the hepatic-lipid covariate (mg/g dry liver) as used in the reported models.	COSU-01-23VW_perpool_matrix_R2.xlsx
Data S4	Per-sample PC scores and $\log_2$ Pareto-scaled abundance matrices for all three matrices	COSU-01-23VW_PCA_scores_and_abundance_matrix.xlsx
Data S5	Diet formulation workbook (formula-driven): formulation of the male 0% and 70% diets, ingredient protein and fat fractions, amino acid and fatty-acid profiles from published composition data (USDA FoodData Central; Gorissen et al. 2018), the estimated amino acid and fatty-acid composition of the two diets, and the sensitivity of the α-linolenate ratio to the corn-oil specification.	COSU-01-23VW_Diet_workbook_DataS5.xlsx
Data S6	Metabolon data tables for cecal content, liver, and plasma: chemical annotation (HMDB, KEGG, InChIKey), sample metadata, peak areas, batch-normalized data, batch-normalized imputed data, volume-normalized data (plasma), log-transformed data	COSU-01-23VW+ CECAL DATA TABLES.XLSX; COSU-01-23VW+ LIVER DATA TABLES.XLSX; COSU-01-23VW+ PLASMA DATA TABLES.XLSX
Data S7	Hepatic RNA-seq: differential expression for the five candidate gene sets (n = 10 pools per diet), per-pool CPM for the 14 shared pools and all 20 male pools, per-pool fatty-acid indices from Data S6, transcript–index correlations, and the complete male differential-expression table (12,116 genes). Reads: GEO accession GSE227120.	COSU-01-23VW_liver_transcripts_DataS7.xlsx
Data S8	Per-animal source files read by the Supplementary Code: necropsy tissue weights (sheet Tissue Weights), hepatic dry matter and lipid quantification, and the gene-level CPM export for the liver pools from which Data S7 was derived	CSU-236-237-239_DRS_Male_and_Female_Necropsy_Tissue_Weights_5-3-26.xlsx; CSU-236-239 LIVER DRY MATTER and LIPID QUANTIFICATION-MALES SYSTAT.xlsx; Expr_Browser_-_70v0_FM_DRS_-_Liver_GE_condensed.xlsx

File	Description	Filename
Data S9	Per-metabolite results of the cage-aware sensitivity analysis for every host feature in cecal content, liver, and plasma: log2 fold change, Welch p and FDR, the multiple-membership mixed-model diet coefficient, SE, approximate p on 6 df and FDR, cage and residual variance components, and intraclass correlation (sheets Cage_CECAL, Cage_LIVER, and Cage_PLASMA); sheets S8A–S8D reproduce Table S8 as written by the Supplementary Code (Supplementary Methods SM9)	COSU-01-23VW_cage_sensitivity_DataS9.xlsx
Supplementary Code	R Markdown document and its rendered report (R 4.6.1) reproducing all analyses: differential abundance, the cage-aware sensitivity analyses of Table S8, PLS-DA with in-fold scaling and permutation (seed 20260924), fatty-acid ratios, pathway analysis, composites, MANCOVA and univariate regressions, transcript analyses, figures and tables; session information in the report lists exact package versions. Pathway analysis uses the MetaboAnalyst 6.0 Mus musculus pathway library and compound database supplied with the code; the per-animal necropsy and hepatic-lipid files and the RNA-seq Expression Browser export that the code also reads are provided as Data S8	Supplementary_Code/DRS_multicompartment_metabolomics.Rmd; Supplementary_Code/DRS_multicompartment_metabolomics.html; Supplementary_Code/metaboanalyst_lib/compound_db.qs, mmu.qs.
README	Instructions for running the Supplementary Code: the folder layout to keep, the R version, the R packages (CRAN and Bioconductor) with the versions used, pandoc, the run command, the expected run time, and the outputs produced	README_Supplementary_Code.txt

Note: FC values are the ratio of group means of the batch-normalized, minimum-imputed data in Data S6 and are reproduced exactly by the Supplementary Code; the matrices in Data S4 are Pareto-scaled and are not the input for FC. Exposure markers are flagged in Data S1 and were excluded from all host-focused analyses (PLS-DA, VIP, concordance, composites).
