# Supplementary material for: Concordant Metabolite Differences Across Cecal Content, Liver, and Plasma in Mice Fed an Obesogenic Diet Containing Common Bean (Phaseolus vulgaris L.): A Multi-Compartment Metabolomics Study
Source: Nutrients. 2026 Nov 5;18(21):3669. doi: 10.3390/nu18213669 (PMC13765519; doi:10.3390/nu18213669)
Supplement: Supplementary file 1 [file nutrients-18-03669-s001.zip › nutrients-4490632-supplementary/Supplementary_Methods_R2_vs5.pdf]

Supplementary Methods

Concordant Metabolite Differences across Cecal Content, Liver, and Plasma in Mice Fed an Obesogenic Diet Containing Common Bean (*Phaseolus vulgaris* L.): A Multi-Compartment Metabolomics Study

Henry J. Thompson, Vanessa K. Fitzgerald, Tymofiy Lutsiv, John N. McGinley, Elizabeth S. Neil, and Hisham Hussan

These sections provide the procedural detail summarized in Sections 2.1–2.9 of the main text. Reference numbers are those of the main-text reference list.

Table SM1. Location of each reporting item.

Reporting item	Location
Strain, substrain, supplier	Main 2.1; SM1
Animals allocated per diet, animals per cage, cages per treatment	Main 2.1; SM1
Allocation and blinding	Main 2.1 (allocation); SM1
Food and energy intake (not measured)	Main 2.1; SM1
Timing and feeding status at necropsy	Main 2.1; SM1
Diet formulation and estimated composition; finished diets not assayed	Main 2.1; SM2; Table S1; Data S5
Pooling rationale, pool construction, and pool-to-donor crosswalk	Main 2.2; SM3; Data S3
Animals and pools not submitted for profiling; exclusion criteria	SM3; Data S3
Plasma sample composition and single-donor sensitivity analysis	Main 2.2; SM3; Data S1
Phenotype linkage and experimental unit at each stage	SM3; SM7
Run composition, quality-control samples, technical variability	Main 2.3; SM4
Missing-value frequency, inclusion criteria, identification confidence	Main 2.3; SM4; Data S1
Untransformed abundance matrices	Data S6
Exposure-marker rule and list of excluded compounds	Main 2.4; SM5; Data S1
PLS-DA implementation, Q^2, permutation procedure, preprocessing within cross-validation	Main 2.4; SM5; Supplementary Code
Fatty-acid ratio construction	Main 2.5; SM6; Table S6
Pathway-analysis settings	Main 2.6; SM6; Table S5; Data S2
Composite-score construction and membership	Main 2.7; SM7; Data S3
Adiposity profile of the profiled pools: depot comparison, Hotelling's T^2, depot principal components	Main 2.7, 3.9; SM7; Table S4; Figure 7
MANCOVA models, covariate sets, degrees of freedom, multiplicity, leave-one-pool-out sensitivity, permutation test, class-mean analysis of covariance	Main 2.7, 3.9; SM7; Table 1; Table S4
Univariate composite regressions, commonality decomposition, diagnostics, within-group correlations	Main 2.7; SM7; Table S4
RNA-seq procedures and gene sets	Main 2.8; SM8; Table S7; Data S7
Statistical power and multiplicity	Main 2.9
Package versions, random seed, analysis code	Main 2.9; SM5; Supplementary Code
Cage structure, pairing without regard to cage, cage-aware sensitivity analyses (mixed model, leave-one-cage-out)	Main 2.1, 2.2, 2.7, 3.9; SM1; SM3; SM9; Table S8; Data S3; Data S9

Reporting item	Location
Ethics protocol chronology	Main 2.1; Institutional Review Board Statement

SM1. Animals, Husbandry, Allocation, and Necropsy

Male C57BL/6J mice (stock 000664) were obtained from The Jackson Laboratory (Bar Harbor, ME, USA) at 20 days of age. They were housed 5 per cage in solid-bottom polycarbonate cages under a 12 h light–dark cycle at 27.5 ± 2 °C and 30% relative humidity, with ad libitum access to filtered water and diet. From receipt until 8 weeks of age the mice were adapted to the husbandry routine and to a purified powdered diet providing 32.5% of energy from fat (based on formulation D12266B, Research Diets, Inc., New Brunswick, NJ, USA) [6,14].

At 8 weeks of age, 20 mice were allocated to each experimental diet group, with the groups balanced on body weight as previously reported [5,6,14], and fed their diet for 14 weeks. On arrival, 100 male mice were unpacked at random into 20 cages of 5, and body weight was recorded on five occasions over the 4-week adaptation period. At 8 weeks of age the 20 animals with the lowest pre-allocation weight gain (the 19 lowest by rank and one cage-mate) were removed to a companion study, and the remaining 16 cages were assigned to the four diets of the dose-response study as whole cages, 4 cages of 5 mice per diet, with nine animals exchanged between cages so that mean body weight and pre-allocation gain were balanced across groups. In the two groups analyzed here, the four control cages (4, 9, 14, and 17) were intact as unpacked, and three of the four bean cages (1, 6, and 8) each received one animal from a cage that went to the companion study. The cage was therefore the unit of diet assignment and of diet delivery, the allocation was balanced rather than an unrestricted randomization of cages, and the treatment-level replication is 4 cages per diet. Cage was not a factor in the primary analyses; the cage-aware sensitivity analyses are described in SM9, and the cage of every animal is given in Data S3 (sheets Pool_crosswalk and Cage_key).

Investigators were not blinded to diet group during husbandry or necropsy. The contract laboratories that performed sample processing and instrumental analysis received the group and diet assignment of every sample submitted.

Food was replaced daily and monitored to ensure ad libitum access. Food intake was not measured: with group housing only food disappearance per cage can be recorded, and that quantity is dominated by spillage and does not estimate intake with useful precision. Consequently, the energy and nutrient exposure actually received by the two groups cannot be established, and every compositional difference between the diets stated in SM2 is a difference in formulated supply, not in intake.

Necropsy took place over 5 consecutive mornings, 8:00 AM–12:00 PM, at 22–23 weeks of age, in the fed state (animals were not fasted). Mice were anesthetized by isoflurane inhalation to a surgical plane, blood was collected by cardiac puncture into EDTA tubes, and the animals were euthanized by cervical dislocation. Plasma was separated by centrifugation (2000 × g, 10 min). Liver and cecal content were collected immediately, snap-frozen in liquid nitrogen, and stored at −70 °C. Tibia length and wet weights of inguinal subcutaneous (left and right), mesenteric, retroperitoneal, and epididymal fat depots were recorded for every animal; total visceral fat is the sum of the mesenteric, retroperitoneal, and epididymal depots. Whole-liver weight was not recorded.

SM2. Diet Formulation and Estimated Composition

Diets were prepared in our laboratory from purified ingredients, except for the bean. They were formulated, as previously reported [5,6,14–16], to nominally equal macronutrient proportions weight by weight, from ingredient and supplier values (58.6% total carbohydrate, 20% total protein, 15.5% total fat, and 5.9% micronutrients, on the AIN-93G basis [24]), providing 32.5% of energy from fat. The fat was corn oil (11.32 g/100 g) with anhydrous butter fat (4.24 g/100 g) in both diets. Casein, dextrose, sucrose, corn starch, cellulose (Solka-Floc), and the AIN-93 vitamin (Dyets 310025) and AIN-93G mineral (Dyets 210025) premixes made up the remainder (Table S1; Data S5).

The bean ingredient was white kidney bean cooked as a whole food with its leachate, freeze-dried, and milled to a powder (total dietary fiber 23.5 g/100 g powder [6]). It was included at 40.8 g/100 g in the 70% diet in place of the corresponding amounts of casein, corn starch, cellulose, and sucrose, and L-tryptophan (0.06 g/100 g) was added. The bean was entered at 40 g protein/100 g, from the Nutrition Facts label of the canned product expressed on a dry-weight basis. Resistant corn starch was unavailable at the time of formulation, and regular corn starch was used in all diets. Consequently, legume fiber replaced most of the cellulose, bean starch replaced most of the corn starch, and bean minerals supplemented the purified premix, which was held constant between diets.

The formulation describes how the diets were composed. The composition of the finished diets was not assayed, and the supply they delivered, which our previous reports did not address, is estimated here. Published composition data place the protein of cooked white bean at about 26 g/100 g of dry matter [25], below the label-derived value used at formulation, because the label conversion omitted fiber and ash from the dry weight. Both values rest on the general nitrogen-to-protein factor of 6.25, a convention for which food-specific alternatives have been proposed [26], so crude protein is itself an approximation and amino-acid supply is the quantity we compare.

Ingredient-based estimates were made from the formulation and the published amino-acid and fatty-acid profiles of casein [27] and of cooked white bean, corn oil, and milk fat [25] (Table S1; Data S5). They give an implied protein content of 16.6 vs. 20.3 g/100 g (70% vs. 0% diet), a branched-chain amino-acid (BCAA) supply 27% lower, methionine plus cysteine 25% lower, and α-linolenate 1.5-fold higher, with linoleate unchanged. Both diets supply every indispensable amino acid above the National Research Council requirement for the mouse [28]: the 70% diet by 1.4-fold (methionine plus cysteine) to 2.9-fold (lysine), and the 0% diet by 1.9- to 3.9-fold (Table S1). The diets are thus adequate but not identical in amino-acid supply, which is the circumstance that a comparison between a purified protein and a whole food produces.

The diets were therefore formulated to nominally match macronutrient proportions, from ingredient and supplier values, but not in macronutrient source or, by estimate, in protein and amino-acid supply, fiber type, or mineral load; the finished diets were not assayed, and intake was not measured (SM1). These differences are treated as alternative explanations throughout the main text, and the response is attributed to the whole-bean matrix rather than to any constituent.

SM3. Pooling, Sample Selection, and Plasma Sample Composition

Rationale for pooling. Metabolomic profiling was performed on pools of two animals, as in our previous reports from this study [14–16]. Pooling reduces the effective sample size and removes within-pool biological variability; pairing the donors on adiposity controls for that covariate at the pool level. Both points bear on the adiposity analyses of Section 3.9 and are taken up in SM7.

Pool construction. Within each diet group the 20 animals were paired on a similar adiposity and weight-gain pattern (adjacent rank on subcutaneous fat mass normalized to tibia length, the primary adiposity phenotype), so that the two donors to a pool were as similar as possible on the variable most likely to dominate metabolome variance. This yielded 10 pools per group. No animal was excluded from pooling or removed from the study for any reason. Pairs were formed without regard to cage: of the 14 profiled pools, 5 (control pools 6, 11, and 21; bean pools 14 and 34) hold two animals from one cage and 9 span two cages, and all 4 cages of each diet contribute donors (Data S3, sheets Pool_crosswalk and Cage_key). The consequences of this structure for inference are taken up in SM9.

Pools submitted. Seven pools per group were submitted for profiling across the three matrices (42 samples). The 3 control pools not submitted were the pairs at the extremes of the adiposity distribution (pool 1, the 2 lowest ranks; pools 41 and 46, the 4 highest ranks). Of the 3 bean pools not submitted, pool 4 held the two lowest ranks, and pools 39 and 44 were omitted because insufficient sample remained to prepare aliquots for all three matrices. Because the omitted pools include the lowest-ranked pair of each group and the two highest-ranked control pairs, the profiled pools span a narrower adiposity range than the cohort, a point that limits the within-group analyses of Section 3.9.

The 6 animals per group not represented in the metabolomics data remain in the phenotypic dataset, and the pooling crosswalk linking every pool to its donors is provided (Data S3).

Sample size and sex. Seven pools per group is the number recommended by the contract laboratory (Metabolon, Inc., Morrisville, NC, USA) for preclinical designs of this kind, reached in discussions that balanced the number of samples evaluated per group against the cost of analysis. It reflects the per-sample cost of the platform and the difficulty of a formal power analysis for untargeted metabolomics, as discussed previously [14]; the power the design provides for a two-sample comparison is stated in Section 2.9 of the main text. Male mice were the focus, as in those reports, because the prevalence of metabolic dysfunction-associated steatotic liver disease and the dietary fiber gap are both larger in men [14].

Cecal content and liver. Every submitted pool contained approximately equal masses of tissue from both donors (cecal content 43–60 mg and liver 50–62 mg per donor; 92–115 mg and 101–124 mg per pool; Data S3).

Plasma. Plasma volume limited pooling. Of the 14 plasma sample positions, 4 (control pools 6, 16, and 26; bean pool 14) were mixtures of 75 µL from each donor, and 10 (4 control, 6 bean) were 91–150 µL from one donor of the pair. We therefore refer to plasma samples rather than pools. Each plasma sample is one independent observation of its position (an individual animal or a two-animal mixture), and no animal contributed to more than one sample. Single-donor samples carry the between-animal variance that pooling would have halved, so the plasma comparisons are less, not more, powerful than an all-pooled design.

The contract laboratory also provided plasma values normalized to the volume extracted. These differ from the batch-normalized values for one sample (COSU-02065; 90 rather than 100 µL extracted) and shift individual fold changes (FC) by up to 10%. The analyses reported here used the batch-normalized, imputed table, and both tables are provided in Data S6.

Single-donor sensitivity analysis. Every plasma comparison was repeated on the 10 single-donor samples alone (Data S1, sheet PLASMA_single_donor_sens). All 270 features at FDR < 0.05 in the full analysis kept their direction, 244 (90%) remained at $p < 0.05$ with 4 vs. 6 observations, and FC values across all features correlated at Spearman ρ = 0.96. The plasma BCAA-parent decrease, which did not reach FDR < 0.05 in the full analysis (Section 3.4), was the only result in the text that weakened (valine p-value = 0.21).

Phenotype linkage and unit of analysis. Phenotypes were linked to each sample as the mean of its actual donors (both animals for cecal content and liver; the single donor where plasma came from one animal). The cross-compartment analyses use group-level FC values and do not pair samples across matrices. The cage was the unit of diet assignment (SM1), and the animal was the unit of observation for the phenotypic comparisons made on all 40 animals. The sample (n = 7 per group per matrix; pooled for cecal content and liver) was the unit of all metabolomic analyses, and no analysis treated the 14 animals contributing to a compartment as independent observations. The cage, as the unit of diet delivery, is addressed by the sensitivity analyses of SM9.

SM4. Metabolomic Platform, Quality Control, and Data Processing

Samples were profiled on the Metabolon Global Discovery (HD4) platform (Metabolon, Inc., Morrisville, NC, USA) [29]: Waters ACQUITY UPLC coupled to a Thermo Scientific Q-Exactive Orbitrap operated at 35,000 mass resolution, with four chromatographic methods (two reverse-phase positive-ion, one reverse-phase negative-ion, one HILIC negative-ion). The run comprised 16 samples per matrix (the 14 male pools analyzed here and 2 female pools not analyzed here).

Quality control followed the platform's standard design. A technical-replicate pool made from an aliquot of every client sample (CMTRX) and process blanks were injected at intervals through a run in which experimental samples were randomized, and a cocktail of internal standards was added to every sample. Median relative standard deviations were 6%, 6%, and 5% for internal standards and 10%, 9%, and 8% for endogenous biochemicals in the CMTRX replicates for cecal content, plasma, and liver, respectively.

Peak areas were corrected in run-day blocks by registering the per-compound median to 1.0 (batch normalization). Missing values were imputed with the per-compound minimum observed value, and $\log_2$-transformed, Pareto-scaled matrices were derived for parametric and multivariate analyses (Data S4). Compounds were identified by retention index, accurate mass (±10 ppm), and MS/MS match to an authenticated standard in the Metabolon library; names carrying an asterisk indicate identification without a purified standard.

Unnamed features (174, 107, and 105 in cecal content, liver, and plasma) were retained in the differential-abundance tables but excluded from host-focused analyses. Missing values before imputation were present in 320, 228, and 310 features. Of these, 55, 13, and 41 features (50, 12, and 32 of them named) were missing in more than half of the 14 samples in cecal content, liver, and plasma, respectively. These are flagged in Data S1, and where a metabolite discussed in the text is affected (mead acid in plasma, α-hydroxyisocaproate in liver) the number of non-detects is stated. The raw peak-area, batch-normalized, and imputed tables for all three matrices are provided as Data S6.

SM5. Differential Abundance, Exposure-Marker Rule, and PLS-DA

Tests. Welch's *t*-test was applied to $\log_2$-transformed, Pareto-scaled values. Because Pareto scaling is a per-feature affine transform, the test is identical on $\log_2$ values. The two-sided Mann–Whitney U test was exact when the 14 values had no ties (minimum attainable p-value of 5.8×10^{-4} with 7 versus 7 observations) and used a normal approximation with tie and continuity correction otherwise; ties arise mainly from minimum-imputed values.

Exposure-marker rule. Diet-exposure markers were defined as compounds that, in this design, enter the host predominantly from the bean ingredient or from its cooking rather than from endogenous mammalian synthesis. The categories are plant betaines, saponins, isoflavones, plant-specific sugars and shikimate-pathway intermediates, Maillard products, ergothioneine and other fungal or bacterial products of the food, and mercapturic acids of cooking-derived electrophiles. The classification was applied by chemical identifier (HMDB, InChIKey) and synonym rather than by name string, and the complete list of excluded compounds, with the rule applied to each, is given in Data S1. Two compounds on that list, pipecolate and N-formylmethionine, have minor endogenous routes (lysine catabolism; mitochondrial translation) and are annotated as such.

PLS-DA. Two-component PLS-DA was fit in R with a NIPALS implementation of PLS regression (0/1 class response) on the host feature set: named, non-exposure, and not constant across the 14 samples (768, 948, and 895 features in cecal content, liver, and plasma). Performance was evaluated by leave-one-out cross-validation, with Pareto scaling refit inside every fold so that no information from the held-out sample entered preprocessing. Q^2 was computed as 1 − PRESS/SStot. Significance of Q^2 was assessed against 1,000 random permutations of the group labels with the same cross-validation procedure and a fixed random seed (20260924). Features were ranked by VIP from the model fit to all 14 samples. Exact package versions are listed in the session information of the Supplementary Code report.

SM6. Fatty-Acid Ratios and Pathway Analysis

Rationale for pairwise ratios. Because the platform scales each metabolite to its own median, a sum of scaled abundances across different fatty acids is a weighted sum with arbitrary weights and does not represent a molar pool. A summed n-3:n-6 ratio was therefore not used. For any two metabolites, the ratio of median-scaled values differs from the molar ratio only by a constant, so the between-group FC in a pairwise ratio is the FC in the molar ratio.

Ratios examined. Per-sample $\log_2$ ratios were formed as the difference of $\log_2$ abundances, compared between groups by Welch's *t*-test with a 95% confidence interval (CI) on the FC, and checked by Mann–Whitney U. The eight ratios examined (n-3/n-6 pairs at matched chain length, product/precursor pairs on each pathway arm, and mead acid/arachidonate) were FDR-corrected within matrix.

Isomers. The platform reports 18:3 as linolenate (α or γ) and 20:3 as dihomo-linolenate (n-3 or n-6) without resolving the isomers. We refer to them as α-linolenate and dihomo-γ-linolenate because neither diet contained a γ-linolenate source and 20:3n-6 is the predominant 20:3 isomer in mammalian tissue. The ratios that use them carry this uncertainty.

Pathway analysis. The analysis followed the quantitative procedure of MetaboAnalyst 6.0 [33] and was implemented in R with the MetaboAnalyst *Mus musculus* KEGG pathway library and compound database, using the 14 male samples of each matrix grouped by diet. Named compounds with an HMDB identifier were mapped to KEGG identifiers through the compound database; for entries carrying several HMDB identifiers the last was used (matched to the database: 595/635 cecal, 738/793 liver, 694/755 plasma) [34]. Data were not sample-normalized (abundances were already batch-normalized), then $glog_{10}$-transformed as in MetaboAnalyst and Pareto-scaled [35]. Each pathway with at least one matched compound was tested against its KEGG metabolite set [36] by the global test, with Benjamini–Hochberg FDR across tested pathways within each matrix, and pathway impact was the sum of the relative betweenness centrality of the matched compounds [37]. The global test was computed with the R package globaltest [38], and the pathway library and compound database were those distributed with MetaboAnalystR [39]. Identical settings were applied across all three tissues (Table S5). The global test is non-directional; the direction of change for each pathway was taken from the differential-abundance results.

SM7. Composite Scores, Adiposity Covariates, and Multivariate Adjustment

Matching and concordance. Metabolites were matched across compartments by Metabolon compound identifier (CHEM_ID), and log_2FC values of the matched host metabolites were compared by Spearman correlation (script provided as Supplementary Code). Host metabolites differing at $p < 0.05$ and in the same direction in all three compartments were tabulated (Table S3), with those also meeting FDR < 0.05 in all three identified separately.

Composite scores. For each of six metabolite classes (ceramides, bile acids, BCAA parents or catabolites, n-3 polyunsaturated fatty acids, glutathione-related metabolites, and ketone bodies with acylcarnitines), a composite score was computed per sample in each compartment as the mean Pareto-scaled abundance of its directionally coherent member metabolites, giving 18 composites (six classes × three compartments). Membership and per-sample scores are given in Data S3. The classes and their memberships were derived from the data of this study, so the unadjusted diet term for a composite is not an independent test of a diet effect; the acylcarnitine class was included on the basis of the hepatic lipid phenotype rather than the concordance screen (Section 3.3).

Phenotype linkage. For every covariate, phenotypes were linked to each sample as the mean of the animals that contributed to it (both donors for cecal content and liver; the contributing donor or donors for plasma; pooling crosswalk in Data S3). These donor-correct phenotypes are used in the compartment-level and univariate analyses (Table S4C, S4E, S4F); the class-level models, which require one covariate vector per pool across the three compartments, use the mean of the two donors of each pool (Table 1; Table S4B, S4D). Four depot masses were carried per sample: subcutaneous, mesenteric, retroperitoneal, and epididymal fat. Hepatic lipid (mg/g dry weight) was from the previously reported assay [6] and was computed for each animal as extracted lipid mass divided by dry liver mass, from the unrounded masses in Data S8; the per-sample values entered in the models are given in Data S3 (sheet Donor_correct_phenotype, column LIVER_LIPID_mg_g_dry), and the Supplementary Code verifies that they equal the values it recomputes.

Adiposity profile of the profiled pools. Phenotype differences between diets were tested by Welch's t-test on the 14 pools and on all 40 animals (Section 3.9; Figure 7A); total depot mass was not significantly lower in the pools but was lower in the full cohort. The four depot masses were compared between diets as a profile by two-sample Hotelling's T^2 and summarized by principal component analysis of their z-scores (Figure 7B; loadings and scores in Table S4). Body weight was not used as a covariate in the multivariate models because it combines fat and lean mass and was collinear with the first depot component ($r = 0.83$).

MANCOVA. Adjustment for adiposity was tested by multivariate analysis of covariance in two orientations. In the primary, class-level analysis, the response for each class was the vector of its three compartment composites (cecal content, liver, plasma; three responses; n = 14 pools), and the predictors were diet group and, in successive models, no covariate (M0); total depot mass (M6); subcutaneous fat (M5); hepatic lipid (M3); the first depot component (M1); the second depot component (M7); the first two components (M2); the first component with hepatic lipid (M4); and total depot mass with hepatic lipid (M8), all reported in Table S4B; Table 1 reports M0, M6, M1, M4, and M8. In the secondary, compartment-level analysis, the response was the vector of the six composites measured in one compartment with the same predictor sets. The diet term has one degree of freedom, for which the Hotelling–Lawley, Pillai, Wilks, and Roy statistics coincide and yield an exact F; the Hotelling–Lawley trace and its F are reported with (m, n − k − m + 1) degrees of freedom, where m is the number of responses and k the number of model parameters, which gives (3, 10), (3, 9), and (3, 8) for class-level models with zero, one, and two covariates, that is, 8–10 denominator degrees of freedom for the diet test; the compartment-level models have 5–7. The depots were reduced to principal components, and at most two covariates enter any model, to hold the parameter count at 4 against 14 observations. Variance inflation factors were computed for every model from the design matrix: in M4 they were 4.8 for diet, 6.0 for the first depot component, and 11.6 for hepatic lipid (the covariates correlate at r = 0.91 and each with diet), in M8 4.8, 1.7, and 6.1, and in the single-covariate models ≤ 2.4 for the depot covariates and 4.6 for hepatic lipid; collinearity inflates the standard error of the diet term and lowers the power of the adjusted test without changing its nominal error rate. The six classes, their composite memberships, and the covariate sets were fixed, from the concordance screen and the prior predictions of Section 3.3, before any adjustment model was fitted. Because the exact F assumes multivariate normality of the residuals, which cannot be checked at this sample size, the significance of each class-level diet term was also assessed by permutation: the reduced model containing the covariates alone was fitted, its residuals were permuted 10,000 times and added back to its fitted values (Freedman–Lane residual permutation), the full model was refitted to each permuted response, and the permutation p-value is the proportion of permuted Hotelling–Lawley traces at least as large as the observed one, with one added to numerator and denominator, using the fixed seed of Section 2.9. As a one-response check on model stability, the class-mean composite (the mean of the three compartment composites of a class) was analyzed by ordinary analysis of covariance with the same covariate sets, with the same leave-one-pool-out sensitivity (Table S4G); because the mean cancels where the compartments differ in direction, this check is interpreted together with the per-compartment univariate analyses of Table S4D. Each model was refitted with each pool omitted in turn, and the largest resulting diet p-value is reported as the leave-one-pool-out sensitivity (Table 1; all models in Table S4). The six class-level diet terms under a given covariate set are treated as one family, and the Benjamini–Hochberg q-value across the six is reported with each p-value for the conservative model (Table 1; Table S4B). The compartment-level models, with six responses on 14 observations, are near-saturated and are reported in Table S4C as descriptive only (Section 2.9). The depot principal components (stats::prcomp), Hotelling's T^2, and MANCOVA were computed in base R (stats 4.6.1) as general linear hypothesis tests on the multivariate linear model; the diet-term tests were checked against stats::anova.mlm (Supplementary Code).

The analysis is associative. Adiposity is a post-allocation variable that may lie on the pathway from diet to metabolite, and a model containing diet and an adiposity covariate can show only whether the diet association of a class persists, is attenuated, or disappears when that covariate is included; it cannot assign the variance the two predictors share, and a non-significant adjusted diet term is a failure to separate them, not evidence of no effect.

Univariate follow-up. As descriptive follow-up (Table S4F; Data S3), each of the 18 composites was regressed by ordinary least squares on diet group and one adiposity covariate: subcutaneous fat mass, mesenteric fat, total visceral fat, epididymal fat, body weight, or hepatic lipid. Because incremental R^2 depends on predictor order, the variance explained is reported as a commonality decomposition [40]: the component unique to diet, the component unique to the covariate, and the component shared between them. Table S4 also gives the coefficient, standard error, and 95% CI of each term, the variance inflation factor, and Shapiro–Wilk and Breusch–Pagan residual diagnostics, with FDR across the 18 composites. Within-group correlations between composites and adiposity are reported with CIs and with a sensitivity analysis omitting the highest-adiposity bean pool; with 7 observations per group these intervals are wide, and no claim is made from them (Section 3.9). The univariate models contain one covariate at a time. They give, for each composite, the fraction of explained variance that diet and a covariate share and the diet term under

covariates not used in the multivariate models, including body weight. A nominal diet term for one composite in a single-covariate model is not interpreted as retention of its class (Section 3.9).

SM8. Liver RNA Sequencing and Transcript Analysis

RNA extraction and sequencing. Sequencing was as described [6,14]. Total RNA was extracted from the 20 male liver pools of the design described in SM3 (10 per diet), which include the 14 pools profiled by metabolomics with the same two donor animals per pool for both assays. Frozen liver was ground under liquid nitrogen, RNA was isolated with the RNeasy mini-kit (cat. 74104, QIAGEN, Germantown, MD, USA), and integrity was assessed on an Experion automated electrophoresis station (Bio-Rad Laboratories, Hercules, CA, USA). cDNA libraries were constructed with the Nugen Universal mRNA kit (Tecan Genomics, Redwood City, CA, USA) and sequenced as paired-end 2 × 150 bp reads on a NovaSeq 6000 (Illumina, San Diego, CA, USA) at the Genomics and Microarray Core, University of Colorado Anschutz Medical Campus (Aurora, CO, USA). The sequencing data are deposited in the Gene Expression Omnibus under accession GSE227120.

Mapping and normalization. Reads were trimmed (quality limit 0.05) and mapped to the *Mus musculus* reference genome GRCm38 in CLC Genomics Workbench (version 21.0.3; QIAGEN, Redwood City, CA, USA). Gene-level counts were normalized by the trimmed mean of M-values [56] and exported as counts per million (CPM). Genes with CPM ≥ 1 in at least 5 of the 20 pools were retained (12,116 genes).

Gene sets. The analysis was restricted to five gene sets specified from the metabolite results before the per-gene values were extracted (Table SM2). Because genome-wide results from these data were reported previously [6], the selection was not blind to that report. The genome-wide table is provided in Data S7 for completeness and is not interpreted.

Table SM2. Gene sets examined.

Gene set	Genes
Polyunsaturated fatty-acid desaturase–elongase module	*Fads2*, *Fads1*, *Elovl5*, *Elovl2*
Lipogenic targets of SREBP-1c	*Scd1*, *Elovl6*, *Fasn*, *Acaca*
PPARα axis	*Ppara*, *Acox1*, *Cpt1a*, *Cyp4a14*
BCAA catabolism	*Bcat2*, *Bckdha*, *Bckdhb*, *Dbt*, *Dld*, *Bckdk*, *Ppm1k*
Glutathione synthesis and turnover	*Gclc*, *Gclm*, *Gss*, *Chac1*, *Gsr*

Differential expression. Because TMM-normalized CPM rather than raw counts were the exported quantity, differential expression between diets was assessed on $\log_2$(CPM + 1) with an empirical-Bayes moderated t statistic incorporating a mean–variance trend, the limma-trend estimator [57]. It was computed with the R package limma (lmFit and eBayes with a mean–variance trend; prior degrees of freedom 4.7, residual degrees of freedom 18), with the code provided as Supplementary Code, and with Benjamini–Hochberg control of the FDR across all retained genes. This differs from the negative-binomial generalized linear model with Wald tests applied to the raw counts in [6]; a count-based model was not applied here. For the 14 pools shared with the metabolomics, $\log_2$(CPM + 1) was also compared by Welch's *t*-test and the Mann–Whitney U test, the tests applied to the metabolite data.

Transcript–index relations. Product-to-precursor fatty-acid indices were formed per pool from the batch-normalized liver data as in SM6 and related to transcript abundance by Pearson and Spearman correlation across the 14 shared pools and within each diet group. Because the two diets form separate clusters on both axes, the across-pool

coefficients restate the group difference and are not interpreted as within-group relations. Transcript abundance was not confirmed by quantitative PCR, and protein abundance and enzyme activity were not measured.

SM9. Cage-Aware Sensitivity Analyses

Cage structure. Diets were assigned to whole cages, 4 cages of 5 mice per diet, with body-weight balancing by exchange of individual animals (SM1), so the cage was the unit of diet assignment and delivery. Pools were formed by adjacent rank of tibia-normalized subcutaneous fat within diet group without regard to cage (SM3), so a pool holds two animals from one cage or from two cages. The 14 profiled pools draw on all 8 cages of the two diets: control cages 4, 9, 14, and 17 and bean cages 1, 6, 8, and 18; 5 pools are same-cage pairs and 9 span two cages; each cage contributes a donor to 2–4 pools (Data S3, sheet Cage_key). The dependence therefore has a multiple-membership structure: a pool belongs to one or two cages and a cage to two to four pools. The leave-one-pool-out and Freedman–Lane residual-permutation procedures of SM7 operate on pools and do not address this structure. Because the cage allocation was balanced on body weight rather than an unrestricted randomization, and because the pools were formed within the realized diet groups, a design-based (randomization) test at the cage level is not available, and the analyses below are model-based sensitivity analyses of the principal diet comparisons and of the class-level models. They do not add replication: the treatment-level replication is 4 cages per diet.

Multiple-membership linear mixed model. For a response vector y over the 14 pools (a composite score, or the $\log_2$ abundance of a host metabolite), the model is $y = Xb + Wu + e$, with $u \sim N(0, \sigma_{cage}^2 I_8)$ over the 8 cages, $e \sim N(0, \sigma_{res}^2 I_{14})$, X the design matrix of the corresponding fixed-effects model (diet alone, M0; or diet with depot PC1 and hepatic lipid, M4; SM7), and W the 14 × 8 membership matrix carrying weight 1/2 on each donor cage of a pool and weight 1 on the single cage of a same-cage pool. For plasma, the membership of a sample is that of its contributing donor or donors (SM3). The variance ratio $\lambda = \sigma_{cage}^2/\sigma_{res}^2$ was estimated by profile restricted maximum likelihood (REML) over $\log \lambda$ on the interval [−12, 8], with the boundary $\lambda = 0$ retained when its restricted likelihood was not exceeded; at the estimate, the fixed effects were obtained by generalized least squares and the residual variance from the weighted residual sum of squares on $14 - k$ degrees of freedom, k being the number of fixed-effect parameters. The diet term was tested by a Wald t statistic (coefficient divided by its standard error) on 6 denominator degrees of freedom, the between-cage degrees of freedom (8 cages − 2 diets), and a 95% confidence interval was formed on the same t distribution. This is the containment choice for a factor applied at the cage level; it is conservative relative to the 10–12 residual degrees of freedom of the corresponding pool-level ordinary least-squares models but does not make the test exact, because the variance components are estimated from eight cages and are imprecise, and the Wald p-values are reported as approximate. A cage variance estimated at zero (the REML boundary) means that the data do not separate cage variance from residual variance; it does not establish that cage effects are absent. The intraclass correlation is $\sigma_{cage}^2/(\sigma_{cage}^2 + \sigma_{res}^2)$. The implementation was checked against a standard mixed-model fit on a single-membership design before use. Per-metabolite tests were corrected within matrix by the Benjamini–Hochberg procedure across the host feature set (768, 948, and 895 features).

Leave-one-cage-out. Each composite model (M0 and M4) was refitted eight times, omitting every pool that holds a donor from one cage (2–4 pools; 10–12 pools and 7 cages remain, and the Wald test then uses 5 degrees of freedom), with the covariates held at their full-sample z-scores as in the leave-one-pool-out analysis of SM7. The largest diet p-value, the range of the diet coefficient, and whether the coefficient kept the sign of the full-sample estimate in all eight refits are reported. The class-level MANCOVA of Table 1 (pool-level multivariate linear model; SM7) was refitted the same way, and the largest diet p-value across the eight omissions is reported; omitting either cage 8 or cage 14 removes 4 of the 14 pools and leaves 4 denominator degrees of freedom for the diet test under M4. The mixed models are fitted to one composite at a time, so they do not test the joint, three-compartment class hypothesis; the joint test remains the pool-level MANCOVA, and the leave-one-cage-out refit of that test is the cage-level check on it.

Reporting. Table S8 gives, for the 18 composites under M0 and M4, the diet coefficient and p-value of the pool-level ordinary least-squares model alongside the mixed-model coefficient, standard error, 95% confidence interval, Wald p-value on 6 degrees of freedom, cage and residual variance components, intraclass correlation, and the leave-one-cage-out maximum p-value, coefficient range, and sign consistency (part A); for the six classes under M0 and M4, the parametric Hotelling–Lawley F, p-value, and Benjamini–Hochberg q-value across the six classes of Table S4B, with the leave-one-cage-out maximum and minimum p-value and the smallest number of pools retained (part B); for each

matrix, the number of host features at FDR < 0.05 by Welch's test and by the mixed model, the number of the latter within the former, the proportion of features whose cage variance was estimated at zero, and the median and 90th percentile of the intraclass correlation (part C); and for the 36 concordant metabolites of Table S3, the mixed-model p-value and intraclass correlation in each compartment and whether the metabolite kept $p < 0.05$ in all three (part D). The per-metabolite results for every host feature are provided as Data S9. The analyses were implemented in base R in the Supplementary Code, which reads the cage of each donor from Data S3.
